# A coordinated network of MYB regulators orchestrates anthocyanin biosynthesis in banana

**DOI:** 10.1093/hr/uhaf361

**Published:** 2026-01-13

**Authors:** Nandita Thakur, Rajni Kanwar, Akhil Singh Karchuli, Sanjana Negi

**Affiliations:** BRIC-National Agri-Food and Biomanufacturing Institute, Main Campus, Sector 81, Sahibzada Ajit Singh Nagar, Punjab 140306, India; BRIC-National Agri-Food and Biomanufacturing Institute, Main Campus, Sector 81, Sahibzada Ajit Singh Nagar, Punjab 140306, India; BRIC-National Agri-Food and Biomanufacturing Institute, Main Campus, Sector 81, Sahibzada Ajit Singh Nagar, Punjab 140306, India; BRIC-National Agri-Food and Biomanufacturing Institute, Main Campus, Sector 81, Sahibzada Ajit Singh Nagar, Punjab 140306, India

## Abstract

Banana, a globally important staple fruit, is naturally deficient in anthocyanins; however, successful engineering of anthocyanin-enriched banana has not been reported to date. Herein, a regulatory network of five R2R3-MYBs (*MusaUP1*, *MusaUP2*, *MbaMA2*, *MusaMA4*, and *MusaMA8*) differentially synchronizing anthocyanin biosynthesis in banana bract is reported. RNA-seq data of red bract revealed a web of regulatory and structural genes fine-tuning anthocyanin accumulation through amalgamation of MYBs and bHLHs activities. Yeast one-hybrid (Y1H) demonstrated differential affinities of these MusaMYBs to banana TT8, CHS, ANR, UFGT, FLS, ANS, and LAR, revealing an intricate pattern of layered regulation in bract pigmentation. Functional competence of this MYB network resulted in intense anthocyanin accumulation in whitish onion and restoration of pigmentation in *myb90/tt8 Arabidopsis* seedlings. Hierarchical regulation in this MYB network stemmed from contrasting control over early and late flavonoid structural genes as revealed by disparate orange fluorescence of *myb90/tt8 Arabidopsis* seedlings after DPBA staining. In banana, a distinctive requirement of *TT8* for pigmentation was observed for *MbaMA2* and *MusaMA8*, while *MusaUP1*, *MusaUP2*, and *MusaMA4* were self-competent, although co-expression of *MusaTT8* augmented the ectopic pigmentation effect. Transcript abundance of flavonoid structural genes in transgenic banana is in coherence with Y1H data, thus catalysing pigmentation up to 500-fold over control. This regulatory MYBs hierarchical framework manifested flux in a spectrum of distinct pigment metabolites, viz peonidin-3,5-diglucoside in *MusaUP1* and *MusaUP2*, dalbergiodin in *MbaMA2*/TT8 lines (FLS-mediated pathway), leucodelphinidin and leucopelargonidin in *MusaMA4* lines (DFR to ANS flux), and prodelphinidin B4 in *MusaMA8* lines. This study will be a step forward towards metabolic engineering for bio-fortification of banana and development of functional foods, as evident by strong antioxidant activities of these MYB lines.

## Introduction

Anthocyanins are excellent dietary supplements with antioxidant properties and health-promoting effects. Historically, they are well known as attractants and defence molecules and chemically are a subclass of water-soluble flavonoids responsible for imparting differential reddish, pinkish, and bluish-purple colouration to flowers, fruits, or leaves [1]. Though the anthocyanin biosynthesis pathway is highly conserved, its regulation is tightly controlled at various transcriptional levels via MYB, bHLH, and WD40 (MBW) transcription factors (TFs) through MBW complex formation. MYB-TF family is one of the largest families, and among them, R2R3-MYBs play a central role in determining the spatial, temporal, and quantitative expression of anthocyanin biosynthetic pathway genes [2]. In banana (*Musa* spp.), Pucker *et al.* [3] identified 285 *R2R3-MYB* transcription factors, and 81 were suggested as potential regulators of flavonoid accumulation. However, recent studies have suggested a two-tier regulation, i.e., through individual TFs and a more integrated regulation as in the MBW ternary complex formation [4–6]. In *Arabidopsis*, distinct sets of MYBs regulate the early (EBGs) and late biosynthetic genes (LBGs) in the flavonoid pathway. General flavonoid biosynthesis pathway genes are categorized into two types as early biosynthetic genes (*CHS*, *CHI*, *F3H*, *F3′H*, *FLS*) and late biosynthetic genes (*DFR*, *ANS*, *UFGT*, *LAR*, *ANR*). In *Arabidopsis* (a dicot plant), R2R3-MYB TFs regulate the early biosynthetic genes independently (e.g., *MYB11*, *MYB12*), while late biosynthetic genes require a ternary MBW complex (MYB-bHLH-WD40). In contrast, *Zea mays* (monocot plant) activates both early and late genes through a single MBW complex (e.g., *C1/Pl1* with *R1/B1* and *PAC1*). This shows a more integrated regulated system in monocots as compared to the stepwise control seen in dicots [7–9]. Functional characterization of MYBs in model plants has been enabled by technological innovations such as transcriptomics or metabolite profiling, laying the foundation for synthetic biology approaches to engineer flavonoid-enriched plants.

In banana, two *MYBs* (MaMYBPA1 and MaMYBPA2) are associated with proanthocyanidin biosynthesis [10], while three other *MYBs* (*MusaMYBA1*, *MusaMYBA2*, and *MusaMYBPA2*) have been shown to activate *AtANS* and *AtDFR* genes [11]. However, neither of these studies has demonstrated visible accumulation of ectopic anthocyanin deposition, indicating that knowledge on transcriptional regulation of the anthocyanin biosynthesis pathway in banana remains incompletely understood. Moreover, information on the global regulation of anthocyanin biosynthesis and related DEGs in banana through RNAseq analysis of red bract in banana is warranted.

In the current study, we demonstrate, to our knowledge, for the first time, the visible accumulation of ectopic anthocyanin deposition in banana tissues after identifying the global regulators as DEGs in anthocyanin-rich red bract tissue of banana through RNAseq analysis. In parallel, we also aimed to identify and characterize functional orthologues or homeologues of MYBs from previously reported and functionally characterized MYBs in *Arabidopsis* and other plant species. Herein, we report the effects of overexpressing these five differentially expressed *MYBs* alone or in combination with bHLH TF and the transcription regulation governed by them. Thereafter, all five identified *MaMYB* TFs were overexpressed alone as well as in combination with the identified *MabHLH* TF in the banana cultivar Rasthali (AAB), which is a premium dessert variety widely known in the global market, comparable to Cavendish [12]. Anthocyanin accumulation is considered a premium quality in fruit valuation; our study can lead to red-pulped banana fruits, besides improving the pathogen resistance, thus potentially offering significant benefits to farmers [13]. This work has demonstrated the functional conservation of these identified MYBs and bHLH in inducing the anthocyanin pathway across onion and anthocyanin-deficient *myb90/tt8 Arabidopsis* mutants. Transactivation of the anthocyanin pathway genes was confirmed for these MYBs and bHLH and corroborated with enhanced levels of flavonoids, anthocyanins, and significantly improved ROS scavenging in banana lines.

Our findings reveal not only the regulatory architecture of anthocyanin biosynthesis in banana but also its potential for bioengineering and metabolic engineering. By leveraging synthetic biology tools and technological innovations, this work provides a framework to generate anthocyanin-enriched cultivars with improved antioxidant potential, pathogen resistance, and market value. Such outcomes underscore wide-ranging applications in agriculture, functional foods, and health-oriented crops.

## Results

### Identification of key transcriptional regulators in banana anthocyanin biosynthesis

**Figure 1:**
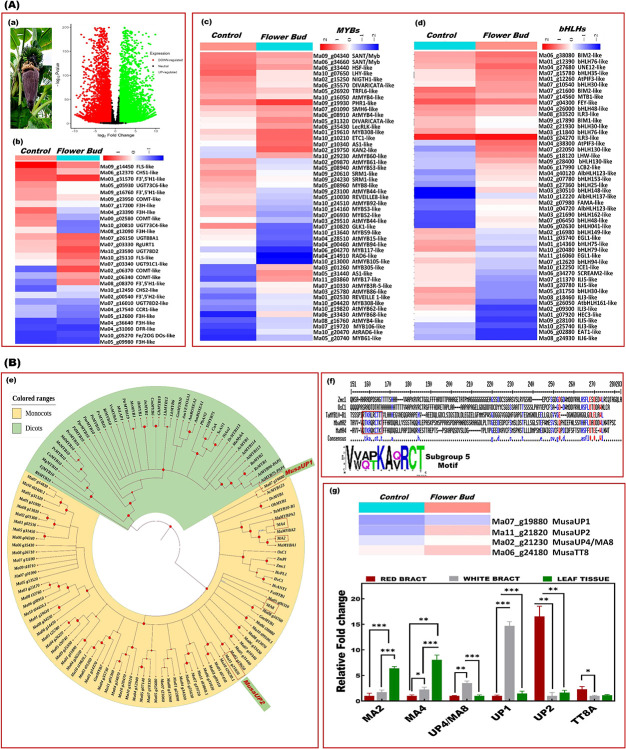
(a) Volcano plot showing differentially expressed genes (DEGs) in red bract tissue when compared to leaf tissue. Red dots indicate significantly upregulated genes (*P* < 0.05 and log₂ fold change ≥1.5), while blue dots indicate significantly downregulated genes (*P* < 0.05 and log₂ fold change ≤1.5). Heat maps of significant DEGs associated with (b) flavonoid biosynthesis pathway, (c) MYB genes, and (d) bHLH transcription factors in red bracts as compared to control. The scale bar represents the log₂ fold change in gene expression in red bracts compared with the control. Upregulated genes are shown in red and downregulated genes in blue, and the colour intensity corresponds to the magnitude of the log₂ fold change. (e) Phylogenetic tree of functionally characterized MYB TF genes in various plant species with the identified and upregulated *MusaMYBs*. The tree was constructed using the neighbour-joining method with 1000 bootstrap replicates. Bootstrap values are denoted by red circles at the nodes, ranging from 0.5 to 1.0. The protein accession numbers used to construct the phylogenetic tree are given in Table S2. (f) Presence of conserved motif related to subgroup 5 family in *MbaMA2* and *MusaMA4* MYB TF selected via homology-based BLAST search. (g) Differential expression of selected MYBs and bHLH TFs from red bract tissue, along with expression profiling of selected genes for *in vivo* validation of transcriptomic data. Statistical analysis was performed using GraphPad version 9.0.3. Statistical significance was checked at *P* ≤ 0.05–0.001.

In the current study, using two complementary strategies, comparative transcriptomic analyses and a homology-based approach, we identified five *MusaMYBs* potentially involved in regulating the anthocyanin biosynthesis pathway in banana. RNA-seq analysis revealed 25 320 differentially expressed genes, among which 19 087 showed significant expression changes (*P* < 0.05), as illustrated in the volcano plot (Fig. 1a). From this set, the 4741 upregulated and 5084 suppressed genes with |log2(FC)| cut-off of ≥1.5 were screened for further downstream analysis, excluding 9262 DEGs with log2FC values between −1.5 and 1.5. DEGs obtained from anthocyanin-rich red coloured flower bracts divulged a plethora of genes providing a deeper insight into the regulation of anthocyanin biosynthesis in banana. A Significant upregulation in flavonoid biosynthesis pathway genes (Fig. 1b) was observed in two *COMT*-like (Ma02_g06370 and Ma02_g06340), one *CHS2*-like (Ma10_g12450), two *F3′,5′H1*-like (Ma08_g08370 and Ma03_g31570), one *FLS*-like (Ma10_g25110), one *RgURT1* (Ma07_g03330), and three *UDP-glycosyltransferase* (UGT78D2: Ma10_g23590; UGT88A1: Ma07_g26150; and UGT91C1-like: Ma07_g03340). Flavonoid biosynthesis pathway genes with strong decline in transcript abundance were one *CCR1*-like (Ma04_g17540), one *CHS1*-like (Ma06_g12370), 2 *COMT*-like (Ma09_g23950 and Ma00_g02580), one *DFR*-like (Ma04_g31060), 2 *F3′,5′H*-like (Ma09_g16760 and Ma02_g00540), 6 *F3H*-like (Ma07_g17200, Ma05_g09980, Ma04_g36640, Ma04_g23390, Ma05_g12600, and Ma08_g12090), one *Fe/2OG Dos*-like (Ma10_g05270), one *FLS*-like (Ma09_g14450), and three *UDP-glycosyltransferase* (Ma10_g20810, Ma05_g05930, and Ma07_g16010). Furthermore, on transcriptional regulators, we have specifically focused on *MYB*s and *bHLH*s in DEGs observed from red bract tissue. Among the top altered MYBs (Fig. 1c) with functions reported in orthologues are *AtMYB68*-like (Ma06_g33430; suberin deposition), *MYB17*-like (Ma11_g03860; flowering and anthocyanin regulation), *AS1*-like (ASYMMETRIC LEAVES1; Ma05_g31440 and Ma07_g10340; leaf polarity), *MYB305*-like (Ma03_g01260; nectar production and flavonoid metabolism), *AtRAD6*-like (RADIALIS; Ma10_g20470; flower symmetry), *KAN2*-like (KANADI-like; Ma02_g19750; organ polarity and anthocyanin regulation), *MYB61*-like (Ma05_g20740; stomatal aperture and mucilage production), *MYB106-*like (Ma07_g19720; trichome development), *REVEILLE* 1-like (Ma01_g02530; circadian clock), *AtMYB4-*like (Ma08_g16760 and Ma06_g08910; repressor of phenylpropanoid pathway), *AtMYB60*-like (Ma10_g29230; stomatal opening and anthocyanin regulation), *AtMYB62-*like (Ma10_g19820; phosphate starvation), *ETC1-*like (Ma10_g10210; anthocyanin regulation), *DIVARICATA*-like (Ma05_g31320; flowering time regulation), *MYB308*-like (Ma01_g19610 and Ma10_g04420; repression of flavonoids), *AtMYB3R-5*-like (Ma07_g10330; cell cycle), and *PHR1*-LIKE (PHOSPHATE STARVATION RESPONSE; Ma09_g19930). The top suppressed MYBs in red bract were *RAD6*-like (Ma04_g14910; flower symmetry), *GLK1-*like (Ma07_g10820; anthocyanin regulation), *AtMYB105*-like (Ma10_g13000; organ patterning), *MYB117*-like (Ma06_g04270; organ patterning and anthocyanin regulation), *MYB59-*like (Ma10_g13640; salicylic induced senescence), *AtMYB94-*like (Ma04_g00460; cuticular wax biosynthesis), *AtMYB15*-like (Ma04_g28510; shikimate pathway regulation), *MYBS3*-like (Ma10_g14160; stress responses), *AtMYB44*-like (Ma09_g23100 and Ma03_g2951; stress responses), *LHY-*like (Late Elongated Hypocotyl; Ma10_g07650; flowering time), and *MYB8*-like (Ma05_g08960 and iron deficiency). In case of bHLHs (Fig. 1d), several DEGs elevated functions in brassinosteroid signalling, such as *ILI3*-like (Ma02_g09300, Ma08_g18460, and Ma10_g25740), *ILI5*-like (Ma09_g28100, Ma07_g11370, and Ma03_g20780), and *bHLH149*-like (Ma02_g16980). Other induced genes of bHLH type belong to *EAT1*-like (Ma06_g02880; pollen development), *ILI6-*like (Ma08_g24930; leaf inclination), *AtbHLH161*-like (Ma05_g26050; flowering time), *HEC3*-like (Ma01_g07920; gynoecium development), *bHLH79-*like (Ma10_g20480; flowering), *SCREAM2*-like (Ma06_g34270; stomatal differentiation), *EGL1*-like (Ma11_g03740; anthocyanin regulation), *BIM1*-like (Ma09_g17890; embryonic patterning), and *ICE1-*like (Ma10_g12250; cold stress response).

Some of the suppressed bHLHs in the DEGs list are *bHLH148*-like (Ma03_g30510; brassinosteroid signalling), *bHLH48-*like (Ma07_g06450; flowering time), *bHLH041*-like (Ma06_g02630; auxin-induced cellular reprogramming), *AtPIF3*-like (Ma04_g38300 and Ma01_g12260; anthocyanin regulation), *LHW*-like (Ma05_g18120; vascular development), and *FAMA*-like (Ma02_g07980; stomatal development).

To further narrow down the candidates and identify potential regulators of anthocyanin biosynthesis, a neighbour-joining phylogenetic tree was constructed using 60 upregulated MYBs, along with previously characterized MYBs from other plant species (Fig. 1e). Genes identified through transcriptomic analysis were coded as *upregulated MusaMYB1* (*UP1*), *upregulated MusaMYB2* (*UP2*), and *upregulated MusaMYB4* (*UP4*). Simultaneously, a homology-based BLAST analysis was performed, and three *Musa MYBs* were identified as *Musa anthocyanin gene 2* (*MbaMA2*), *Musa anthocyanin gene 4* (*MA4*), and *Musa anthocyanin gene 8* (*MA8*), which were also included in the phylogenetic analysis (Fig. 1f). *UP4* and *MA8* were determined to be the same gene, and are referred to as *MusaMA8* in this study. The expression of selected *MusaMYBs* was validated through qRT-PCR in three different tissues, i.e., flower red bract, flower white bract, and leaf tissue. *MusaUP1*, *MusaUP2*, and *MusaMA8* showed the highest expression in flower bracts in comparison to leaf tissue, which validates the transcriptomic data (Fig. 1g). Similarly, the *MusaMA4* and *MbaMA2* genes had considerably higher transcript levels in leaf tissue than flower bracts, suggesting potential hitherto unknown functions. Also, *MusaTT8*, initially identified through homology-based blast studies using well-characterized bHLH in other plant species, was found to be upregulated in transcriptomic data and further validated through qRT-PCR (Fig. 1g).

### Conserved motif analysis of *MusaMYBs* implicated in anthocyanin biosynthesis

The gene structure was visualized using GSDS to analyse the distribution of exons and introns, highlighting structural variations among the *MusaMYB* genes (Fig. S2a). Also, conserved motifs were initially identified through the MEME suite. Domain 1 and domain 2 showed the conserved R2R3-MYB domain across all *MusaMYBs* (Fig. S2b). All the selected R2R3-*MusaMYBs* (*MbaMA2*, *MusaMA4*, *MusaMA8*, *MusaUP1*, and *MusaUP2*) were further analysed for conserved motifs by comparing them with well-characterized anthocyanin-related genes. All MYBs showed the presence of bHLH-binding motif [D/E]Lx2[R/K]x3Lx6Lx3R in their protein sequences (Fig. S2c). This motif is highly conserved within the C-terminal region of many MYB proteins, particularly those belonging to subgroup 2. It plays a critical role in mediating protein–protein interactions, especially with bHLH TF and WD40 repeat proteins, facilitating the formation of the MYB-bHLH-WD40 (MBW) complex. In MusaUP1 and MusaMA8, subgroup 6 motif [K/R]P[R/Q][PR]R[R/S/T]F was present, which is a signature feature of R2R3-MYB TFs that regulate anthocyanin and flavonoid biosynthesis (Fig. S2d). This motif is thought to influence the transcriptional activation capacity of MYBs by modulating their interaction [14, 15]. In MbaMA2 and MusaMA4, another motif was found, which is generally present in subgroup 5 [V/L][W/I]xxKAxRCT [16]. The RCT motif present in these two MYBs aligned with the same motif present in *Triticum aestivum* MYB10, *Z. mays* C1, and *Oryza sativa* C1 protein, already well characterized for anthocyanin biosynthesis regulation (Fig. 1f). Similarly, the MusaUP2 protein sequence had shown the presence of the subgroup 6 motif [A/S/G/NDV]. The NDV motif is a conserved amino acid sequence found in the C-terminal region of R2R3-MYB TFs, especially involved in regulating anthocyanin biosynthesis and floral pigmentation [17].

### Transient overexpression in onion-based monocot system reveals *MusaMYB*s-driven activation of pathway genes

**Figure 2:**
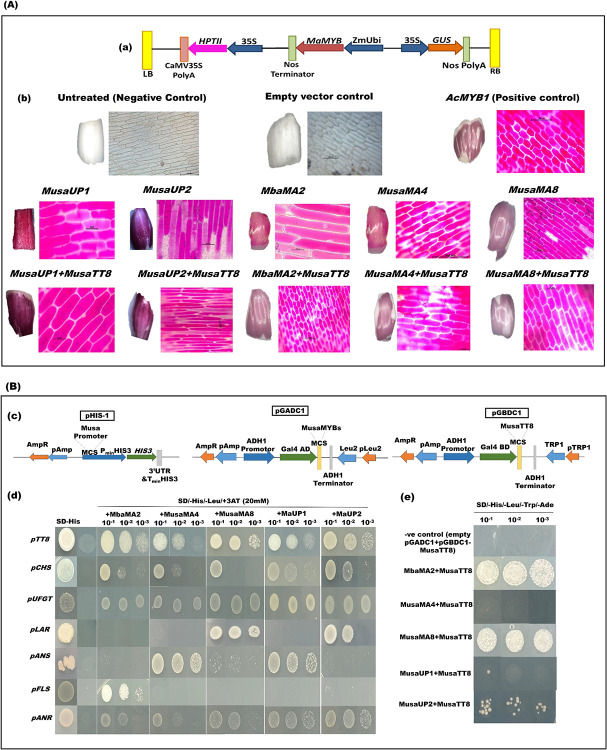
(a) Schematic representation of the pCAMBIA 1301 overexpression cassette for plant transformation. The construct contains the full-length coding sequence of the selected *MusaMYBs* (*MbaMA2*, *MA4*, *MA8*, *UP1*, *UP2*) and *MusabHLH TT8* cloned under the *Z. mays* ubiquitin promoter and followed by NOS terminator. (b) Transient overexpression assay in *A. cepa* (onion) epidermal cells to assess the transcriptional activation potential *of MusaMYBs* (*MbaMA2*, *MA4*, *MA8*, *UP1*, *UP2*) alone as well as in combination with *Musa*bHLH TF *TT8*. *Allium cepa AcMYB1*, functionally well characterized, was used as a positive control for the experiment, and the empty vector served as a negative control. (c) Y1H assay showing interaction of MusaMYB TFs with flavonoid and anthocyanin pathway gene promoters. Promoters (*pMusaCHS*, *pMusaANS*, *pMusaANR*, *pMusaUFGT*, *pMusaLAR*, *pMusaFLS*, *pMusaTT8*) are represented vertically, and MbaMA2, MusaMA4, MusaMA8, MusaUP1, and MusaUP2 are shown horizontally. (d) Y2H assay demonstrating direct interaction between *MusaMYB* transcription factors and *MusaTT8*. Serial dilutions of saturated cultures were spotted to evaluate interaction strength in both Y1H and Y2H assays. Yeast growth on selective media indicates positive interaction between *MusaMYBs* and *MusaTT8* as well as pathway promoters, respectively, whereas negative controls (empty vectors) show no growth in both experiments.

To assess the transcriptional activation potential of selected *MusaMYBs* (*MbaMA2*, *MA4*, *MA8*, *UP1*, *UP2*) and *MusabHLH TT8*, their coding regions were placed under the control of *Z. mays* polyubiquitin promoter (Fig. 2a). Sequencing results revealed that *MbaMA2* was cloned from the B-genome of the Rasthali cultivar, while the other *MYBs* showed more sequence similarity with the A-genome, displaying 2%–3% varietal differences. These binary vectors were then mobilized to the *Agrobacterium tumefaciens* EHA105 strain and subsequently used to infect the whitish bulb onion as a monocot model system. After the third day of infection, whitish onion cuttings developed pink–purple pigmentation similar to the positive control *AcMYB1*, proving the activation of the anthocyanin pathway by our identified genes (Fig. 2b). However, no pigmentation developed in onion cuttings infected with the control *Agrobacterium*.

### Yeast one-hybrid assay confirms direct binding of *MusaMYBs* to anthocyanin-related gene promoters

To validate the direct regulatory involvement of MusaMYBs in the anthocyanin biosynthetic pathway, yeast one-hybrid (Y1H) assays were performed to evaluate their binding affinity to the promoter regions of several key structural genes involved in flavonoid biosynthesis (Fig. S4). All MusaMYBs demonstrated robust binding activity to the *TT8 promoter* (*pTT8*) of MusaTT8 used throughout this study, as indicated by yeast growth up to a 10^−3^ dilution on SD/−His +3-AT (20 mM) medium, proving strong transcriptional activation potential. In contrast, moderate binding [10-2] was observed between MusaMYBs and *Chalcone synthase promoter* (*pCHS*), while faint growth with weak interactions [10-3] was recorded with the promoters of *anthocyanidin reductase* (*pANR*) and *pUFGT*, indicating limited or gene-specific regulatory influence on these targets. However, *MbaMA2* displayed robust growth [10-3], pointing towards strong and specific interaction with the *flavonol synthase promoter* (*pFLS*), whereas no other MYBs exhibited detectable binding to this promoter, suggesting a unique regulatory role in flavonol biosynthesis. The *anthocyanidin synthase promoter* (*pANS*) showed strong binding [10-3] with MusaUP1 and MusaMA4, whereas MusaMA8 and MusaUP2 showed very weak interactions [10-1], implying differential regulatory capacities among selected MusaMYBs (Fig. 2d). Further, the *leucoanthocyanidin reductase pLAR* demonstrated strong binding with MusaUP2 [10-2] and MusaMA8 [10-3], while the remaining MYBs did not show any detectable interaction, highlighting promoter-specific MYB-DNA binding dynamics that may contribute to fine-tuned regulation within the anthocyanin branch of the flavonoid pathway. Quantitative measurement of yeast growth was not feasible in our system; therefore, interaction strength was inferred from dilution-dependent growth patterns.

### Yeast two-hybrid assay confirms direct interaction of *MbaMA2* and *MusaMA8* with *MusaTT8*

To determine whether the MYB proteins directly interact with the bHLH factor TT8, we performed a yeast two-hybrid (Y2H) assay for all five genes. The *MusaMYB*s were fused to the GAL4 activation domain (AD), and *MusaTT8* was fused to the GAL4 DNA-binding domain (BD) (Fig. 2c). Co-expression of these constructs supported growth on selective SD/−Leu/−Trp/−His/−Ade medium, whereas all negative controls showed no growth. Among the tested *MYB*s, *MusaUP1* showed no detectable interaction, while *MusaUP2* showed less growth (10^−3^ dilution). In contrast, *MbaMA2* and *MusaMa8* exhibited strong and consistent interaction signals, with visible dense growth sustained up to the 10^−3^ dilution. However, *MusaMA4* showed negligible growth, indicating minimal or no interaction with *MusaTT8*. The serial dilution assay, therefore, highlights *MbaMA2* and *MusaMA8* as the primary MYBs that physically associate with *MusaTT8*, supporting their TT8-dependent role in activating the anthocyanin pathway (Fig. 2e).

### Anthocyanin-deficient *myb90/tt8 Arabidopsis* complementation confirms the role of banana MYBs in anthocyanin and flavonol biosynthesis

**Figure 3:**
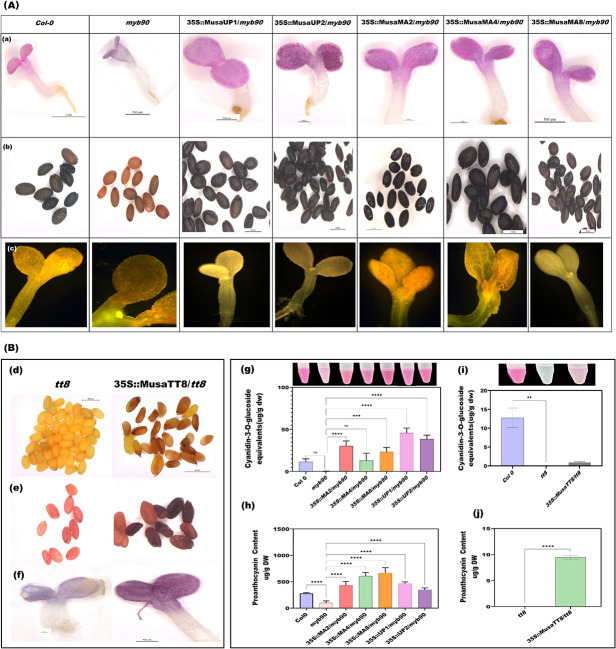
Functional characterization of 35S::MusaMYBs in *Arabidopsis myb90* mutant background. (a) Phenotypic comparison of 4-day-old *Arabidopsis* seedlings grown on ½ MS medium supplemented with 3 ppm norflurazon. From left to right: wild-type (*Col-0*), *myb90* mutant, and T1 generation transgenic lines overexpressing 35S::MusaMYBs (*MusaUP1*, *MusaUP2*, *MbaMA2*, *MusaMA4*, *MusaMA8*). Enhanced anthocyanin accumulation in complemented lines, suggesting restoration of anthocyanin biosynthesis. (b) DMACA staining of T_1_ seeds showing flavonoid accumulation. Darker staining intensity indicates high proanthocyanidin content in the seed coat. (c) DPBA staining of 4-day-old seedlings for visualizing flavonol accumulation. Orange fluorescence under UV light reflects the differential accumulation of flavonols. (d) Functional characterization of *35S::MusaTT8* in *Arabidopsis tt8* mutant background. Phenotypic comparison of T_1_ seeds. (e) DMACA staining of T_1_ seeds showing proanthocyanidin accumulation. (f) Four-day-old *Arabidopsis* seedlings grown on ½ MS medium supplemented with 3 ppm norflurazon: *tt8* mutant and 35S::MusaTT8/*tt8*. (g) Anthocyanin content in 4-day-old seedlings overexpressing *MusaMYBs* in the *myb90* mutant background. (h) Proanthocyanidin content in T1 complementation seeds expressing *MusaMYB* genes in the *myb90* mutant. (i) Anthocyanin content in T1 seedlings expressing *MusaTT8* in the *tt8* mutant background. (j) Proanthocyanidin content in T1 seeds expressing *MusaTT8* in the *tt8* mutant background. Values represent mean ± SE.

Complementation assays in *Arabidopsis thaliana myb90* and *tt8* mutant backgrounds revealed that *MbaMA2*, *MusaMA4*, *MusaMA8*, *MusaUP1*, *MusaUP2*, and *MusaTT8* retained conserved functionality capable of restoring anthocyanin and flavonoid biosynthesis. In *myb90* mutant, which exhibits reduced anthocyanin pigmentation, overexpression of *MusaMYBs* restored anthocyanin accumulation in mutant seeds to levels comparable to wild type (*Col-0*) when grown on norflurazon-supplemented ½ MS medium, indicating successful partial complementation (Fig. 3a and g). In contrast, the *myb90* mutant showed significantly diminished pigmentation (Fig. S6a).

Further validation using DMACA staining confirmed enhanced proanthocyanidin accumulation in complemented lines relative to the mutant control (Fig. 3b and h). Additionally, DPBA staining revealed that *MbaMA2* and, to some extent, *MusaMA4* induced orange fluorescence under UV light, indicative of flavonol accumulation and suggesting their role in regulating EBGs. However, *MusaMA8*, *MusaUP1*, and *MusaUP2* did not show orange fluorescence, supporting their involvement in the regulation of LBGs (Fig. 3c). Furthermore, complementation assays in the *tt8* mutant background were performed using mutant seeds expressing *MusaTT8*. Phenotypic assessment based on seed coat pigmentation, norflurazon response, and DMACA staining indicated partial functional complementation (Fig. 3e–g, i–j). This partial complementation is consistent with previous findings highlighting the limited functional compatibility of monocot *TT8*-like genes in dicot systems [11]. Similarly, *MusaMYB*s were also overexpressed in the *tt8* mutant background to assess whether they could activate the anthocyanin biosynthesis pathway in the absence of TT8. Among the five *MusaMYB* genes, *MusaMA4* and *MusaUP1* exhibited clear complementation, showing a significant increase in anthocyanin accumulation in 4-day-old T1 seedlings despite the loss of endogenous TT8 (Fig. S6b). Both MYBs partially rescued the pigmentation defect of the *tt8* mutant, demonstrating their strong transactivation capacity. In contrast, the remaining *MYBs* displayed only mild or negligible restoration of anthocyanin levels. *MusaMA4* showed the most pronounced complementation activity in the tt8 background. However, when overexpressed with *MusaTT8*, it was able to restore anthocyanin accumulation (Fig. S6c).

### Stable overexpression of *MusaMYBs* in banana reveals distinct roles in anthocyanin biosynthesis

**Figure 4:**
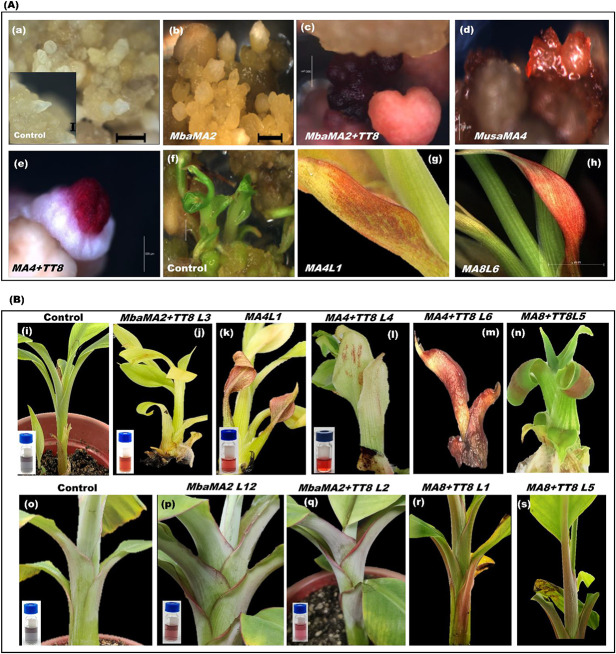
Developmental stages of banana somatic embryos, from left to right: (a) control (*Musa* sp. cv. Rasthali; AAB); (b) overexpression of *MbaMA2*; (c) *MbaMA2* + *MusaTT8*; (d) *MusaMA4*; (e) *MusaMA4 + MusaTT8*; (f) control (*Musa* sp. cv. Rasthali; AAB); (g) *MusaMA4 L1*; (h) *MusaMA8 L6*, transcription factors. These *MusaMYB*s showed normal embryogenesis and successful regeneration into healthy plantlets, indicating their controlled regulation of anthocyanin biosynthesis conducive to plant development. Anthocyanin accumulation in transgenic overexpression lines of young banana plantlets: (i) control (*Musa* sp. cv. Rasthali; AAB); (j) *MbaMA2 + TT8*; (k) *MusaMA4*; (l-m) *MusaMA4 + TT8*; (n) *MusaMA8 + TT8*; (o) *Control*; (p) tissue-specific anthocyanin accumulation in mature banana plants. Overexpression lines of *MbaMA2* showing accumulation in axial parts of the pseudostem leaf sheaths: (q) *MbaMA2 + MusaTT8*; (r–s) overexpression lines of *MusaMA8 + MusaTT8* showed accumulation in the pseudostem leaf sheaths.

Overexpression of individual *MusaMYB* was carried out in embryogenic cell suspension (ECS) of banana, while additional co-transformations were performed to co-express individual *MusaMYB* with *MusabHLH*. Thereafter, anthocyanin accumulation was monitored during various developmental stages, from somatic embryo formation to shoot emergence, and was compared with the control (Figs 4a, f, and S8). In *MbaMA2*, no anthocyanin pigmentation was detected in embryos when overexpressed alone (Fig. 4b). However, co-transformation with *MusaTT8* led to visible anthocyanin accumulation, indicating that *MbaMA2* requires *MusaTT8* for transactivation of anthocyanin biosynthesis genes (Fig. 4c and j). However, expression of *MusaMA4* (Fig. 4d and k) and *MusaMA8* (Fig. 4h) alone could induce pronounced anthocyanin accumulation during early plantlet stages. Moreover, their co-expression with *MusaTT8* resulted in intense pigmentation in somatic embryos, particularly with *MusaMA4* (Fig. 4e, l–m) and leaves of *MusaMA8* (Fig. 4n). At maturity, as compared with the control (Fig. 4o), *MbaMA2* and *MbaMA2 + MusaTT8* plants exhibited tissue-specific anthocyanin accumulation along the axial region of pseudostem sheaths (Fig. 4p and q), whereas *MusaMA8 + MusaTT8* co-transformed lines displayed pigmentation in the basal region of the pseudostem (Fig. 4r–s).

In contrast, *MusaUP1* (Fig. S8a–d) and *MusaUP2* (Fig. S8e–h), when overexpressed alone or with *MusaTT8* (Fig. S8i–l, m–p, respectively), induced strong anthocyanin production during somatic embryogenesis. However, the anthocyanin accumulation was so excessive that it probably resulted in phytotoxic effects, ultimately hindering the regeneration of transgenic lines (Figs S9 and S10).

### Gene-specific and combinatorial effects on anthocyanin pathway activation in overexpression lines

The expression profile of eight key genes involved in the anthocyanin biosynthesis pathway (*CHS*, *ANS*, *UFGT*, *DFR*, *LAR*, *ANR*, *CHI2*, and *FLS*) was analysed in transgenic overexpression (OE) lines of *MbaMA2*, *MusaMA4*, and *MusaMA8*, both independently and in combination with *MusaTT8*. These genes represent critical steps from the early to the late stages of the flavonoid pathway. All the pathway genes were transcriptionally activated in the overexpression lines, consistent with the Y1H results, where strong binding of the *MusaMYBs* with *pCHS* was observed. As the first committed step in the pathway, *CHS* serves as a key regulatory gene for directing metabolic flux towards anthocyanin biosynthesis. In *MbaMA2* OE lines, a moderate increase in expression was observed. However, when *MbaMA2* was co-overexpressed with *MusaTT8*, a notable increase in expression was observed, particularly in LBGs such as *ANS*, *UFGT*, and *DFR* (Fig. 5a and b), indicating a synergistic regulatory effect. Interestingly, *MusaMA4* independently induced a dramatic upregulation of *ANS* expression, with up to a 200-fold increase compared to the control (Fig. 5c). Co-overexpression of *MusaMA4* with *MusaTT8* further amplified the activation of the LBGs, again reaching a 200-fold increase (Fig. 5d), suggesting that *MusaMA4* is a potent activator of LBGs. In *MusaMA8*, its independent overexpression led to the selective and prominent upregulation of *LAR*, along with moderate activation of other genes (Fig. 5e). However, co-overexpression with *MusaTT8* resulted in significant expression of LBGs; still, *MusaMA8* alone showed an independent regulatory mechanism for *LAR* expression in banana (Fig. 5f). The expression of *MbaMA2*, *MusaMA4*, and *MusaMA8* was significantly elevated in their respective overexpression lines, as shown in Fig. S11. Expression profiling of *MusaUP1* and *MusaUP2* in nonregenerating callus tissues demonstrated a marked upregulation of several structural genes associated with the flavonoid biosynthetic pathway. In particular, both genes strongly enhanced the expression of *CHS*, a key enzyme that channels phenylpropanoid pathway intermediates towards anthocyanin biosynthesis. MusaUP1 led to an approximately 200-fold increase in *CHS* expression, while MusaUP2 induced a dramatic 1000-fold increase, suggesting potential phytotoxic effects that may hinder regeneration into whole plants (Fig. S12a). Furthermore, gene expression analysis revealed downregulation of chlorophyll biosynthesis genes alongside upregulation of chlorophyll degradation genes in the overexpressing callus tissue of both *MYB*s (Fig. S12b and c), indicating a disruption in chlorophyll homeostasis potentially linked to the observed developmental inference.

**Figure 5:**
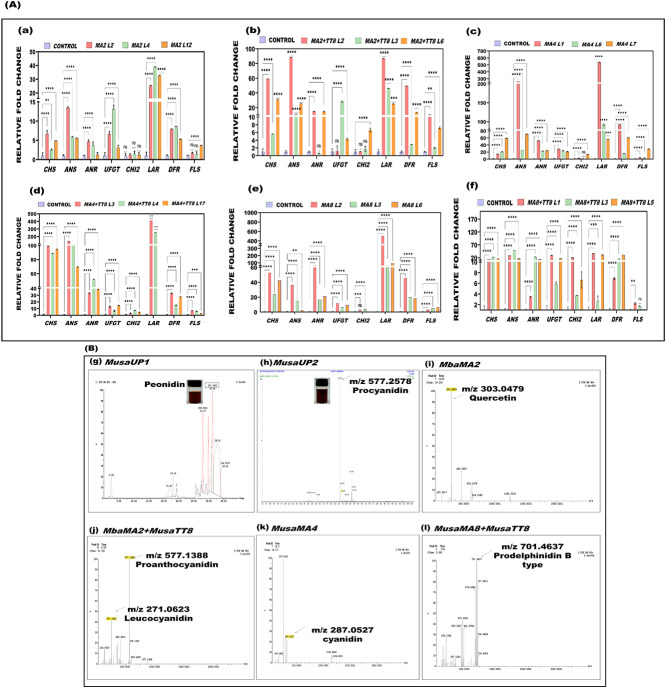
Continues.

**Figure 5:**
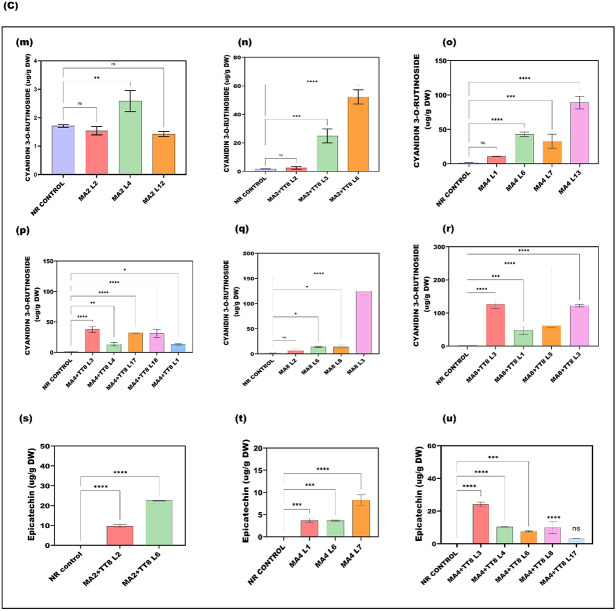
Expression profile of anthocyanin biosynthesis pathway genes in different transgenic overexpression lines of MusaMYB TFs (*MbaMA2*, *MusaMA4*, *MusaMA8*) alone as well as in combination with bHLH TF (*MusaTT8*); (a) relative fold change value in *MbaMA2* OE lines showing the transcriptional activation of the eight pathway genes, i.e., *MaCHS*, *MaANS*, *MaANR*, *MaUFGT*, *MaCHI2*, *MaLAR*, *MaDFR*, and *MaFLS*, respectively; (b) relative fold change value in *MbaMA2* in combination with *MusaTT8* OE lines; (c) relative fold change value in *MusaMA4* in OE lines; (d) relative fold change value in *MusaMA4* in combination with *MusaTT8* OE lines; (e) relative fold change value in *MusaMA8* OE lines; (f) relative fold change value in *MusaMA8* in combination with *MusaTT8* OE lines. LC–MS library screening of major metabolites in overexpression lines and nonregenerative tissues of selected *MusaMYB* genes. (g) Spectra depicting peonidin-3,5-diglucoside from nonregenerative tissue of overexpressing *MusaUP1* and *MusaUP2*; (h) spectra depicting proanthocyanidin accumulation in nonregenerative tissue overexpressing MusaUP2; (i–l) representative MS spectra of major identified compounds in methanolic extracts from *MbaMA2*, *MusaMA4*, and *MusaMA8* co-overexpressed lines with *Musa TT8*. Peaks are annotated with observed *m/z* values, and compound identities were assigned based on library matching with reference spectra. Variations in peak profiles and intensities indicate differential accumulation of flavonoids and anthocyanins. LC–MS-based quantification of cyanidin-3-*O*-rutinoside and epicatechin in MusaMYBs overexpressing lines using standards. Quantification was performed using calibration curves of the above-mentioned standards. (m) Cyanidin-3-*O*-rutinoside content in *MbaMA2* overexpressing lines; (n) cyanidin-3-*O*-rutinoside in *MbaMA2* overexpressing lines co-transformed with MusaTT8; (o) cyanidin-3-*O*-rutinoside content in *MusaMA4* overexpressing lines; (p) cyanidin-3-*O*-rutinoside in *MusaMA4* overexpressing lines co-transformed with *MusaTT8*; (q) cyanidin-3-*O*-rutinoside content in *MusaMA8* overexpressing lines; (r) cyanidin-3-*O*-rutinoside in *MusaMA8* overexpressing lines co-transformed with *MusaTT8*. (s) Epicatechin content in *MbaMA2* overexpressing lines co-transformed with *MusaTT8*; (t) epicatechin content in *MusaMA4* overexpressing lines; (u) epicatechin in *MusaMA4* overexpressing lines co-transformed with *MusaTT8.* Data represent mean ± SD of three independent experiments. Asterisks denote statistically significant differences at *P* ≤ 0.05–0.00.

### Enhanced secondary metabolite accumulation correlates with *MusaMYBs* overexpression

To assess the metabolic impact of *MusaMYB* overexpression, biochemical analyses were performed using methanolic extracts from transgenic overexpression lines of *MbaMA2*, *MusaMA4*, and *MusaMA8*, and from nonregenerating callus tissues of *MusaUP1* and *MusaUP2*. A significant increase in flavonoid content in *MbaMA2* (*~*1800–2800 μg CE/g FW), *MusaMA4* (~900–1000 μg CE/g FW), and *MusaMA8* (*1805–2431* μg/g FW) overexpression lines as compared to the control (~670–790 μg CE/g FW) was observed (Fig. S13a). Likewise, *MusaUP1* (~742 μg CE/g FW) and *MusaUP2* (~1202 μg CE/g FW) showed elevated flavonoid levels, while co-overexpression of *MusaTT8* led to a slight further increase (Fig. S13b). In contrast, total anthocyanin content varied among the transgenic lines: *MbaMA2* showed pronounced tissue-specific anthocyanin accumulation in the midrib region (Fig. S15), while *MusaMA4* and *MusaMA8* showed comparatively higher anthocyanin content when co-expressed with *MusaTT8* (Fig. S13c). Notably, *MusaUP1* (~95.49–150 μg/g DW) and *MusaUP2* (~40–65 μg/g DW) overexpression led to increased anthocyanin content compared to the negligible levels in control tissues (~1.3–11.21 μg/g DW) cyanidin-3-*O*-glucoside equivalents, indicating a potential phytotoxic effect that could explain their failure to regenerate (Fig. S13d). Additionally, extracts from transgenic lines and nonregenerative callus showed significantly higher antioxidant activity as compared to control (Fig. S13e and f), supporting the role of candidate *MusaMYB* TFs in regulating secondary metabolite biosynthesis and oxidative stress response. Also, MYB-overexpressing lines exhibited reduced chlorophyll a and b and markedly lower total carotenoids compared with the control (Fig. S14).

### LC–MS-based metabolite profiling reveals gene-specific regulation of flavonoid and anthocyanin biosynthesis in banana

We performed LC–MS analysis to profile metabolites associated with the flavonoid biosynthetic pathway relative to the control. Detected features were subsequently identified and annotated through screening against the KEGG metabolite library (Supplementary File S2; Table S4). Nonregenerative *MusaUP1* and *MusaUP2* overexpressing callus showed elevated levels of flavanols, proanthocyanidins, and anthocyanin (peonidin), otherwise absent at this stage (Fig. 5g and h). *MbaMA2* overexpression led exclusively to flavanol and flavanone accumulation, whereas it produced the pigmented metabolite dalbergiodin via the FLS-mediated pathway when co-overexpressed with *MusaTT8* (Fig. 5i and j). Similarly, *MusaMA4* overexpressing lines resulted in leucodelphinidin and leucopelargonidin accumulation, reflecting DFR to ANS flux towards anthocyanin biosynthesis and LBGs regulation. *MusaMA8* overexpression increased flavanols, while co-overexpression with *MusaTT8* enabled prodelphinidin B4 accumulation (Fig. 5k and l). Collectively, these results suggest that *MbaMA2*, *MusaMA4*, and *MusaMA8* regulate LBGs with *MusaTT8* via MBW complex formation, whereas *MusaUP1* and *MusaUP2* could be strong regulators of EBGs and LBGs to promote anthocyanin biosynthesis in banana.

### LC–MS-based evidence for divergent pathway control among the overexpression lines

Multiple studies have shown that cyanidin-3-rutinoside is the predominant anthocyanin in banana tissues. Pazmiño-Durán *et al.* and Kitdamrongsont *et al.* identified it as the major pigment in *Musa paradisiaca* bracts and wild *Musa* sp., while Fu *et al.* and other analyses of red banana cultivars reported rutinoside derivatives as key contributors to peel colouration. Recent LC–MS-based profiling further confirms the abundance of cyanidin-3-rutinoside in banana inflorescences and coloured varieties [18–20].

Therefore, we performed targeted LC–MS analysis of leaf tissues from the overexpression lines, which revealed a clear differential requirement for MusaTT8 in regulating cyanidin-3-rutinoside biosynthesis. Overexpression of *MbaMA2* alone (Fig. 5m) produced only a modest increase (~2–15 μg/g DW), whereas *MbaMA2 + MusaTT8* co-overexpression enhanced cyanidin-3-rutinoside levels up to ~30–50 μg/g DW (Fig. 5n), indicating that *MbaMA2* requires *MusaTT8* for efficient flux enhancement. In contrast, *MusaMA4* overexpression alone accumulated ~30–80 μg/g DW of cyanidin-3-rutinoside, comparable to *MusaTT8* co-overexpression lines (Fig. 5o–p), suggesting that *MusaMA4* functions independently of *MusaTT8*. A similar dependency pattern to *MbaMa2* was observed for *MusaMa8*, which accumulated only 3–20 μg/g DW of cyanidin-3-rutinoside when expressed alone but exceeded 100–120 μg/g DW in *MusaMA8 + MusaTT8* co-overexpression lines, consistent with the stronger visible pigmentation observed in these plants (Fig. 5q–r). Epicatechin quantification further supported these regulatory relationships. Epicatechin was detected predominantly in *MbaMA2 + MusaTT8* (Fig. 5s) and *MusaMA4* overexpression lines, aligning with prior evidence that *MbaMA2* regulates late-pathway genes through the MBW complex, whereas *MusaMA4* can directly activate *pANS* and redirect metabolic flux towards epicatechin production (Fig. 5t–u). Targeted LC–MS also confirmed enhanced accumulation in the midrib of *MbaMA2* overexpression lines and leaf tissue of co-overexpressed *MbaMA2* and *MusaTT8* transgenic lines (Fig. S15).

## Discussion

The present study was focused on functionally characterizing *Musa* R2R3-MYB transcription factors (*MusaUP1*, *MusaUP2*, *MbaMA2*, *MusaMA4*, *MusaMA8*) involved in anthocyanin biosynthesis using transcriptomic profiling, overexpression (transient and stable), *Arabidopsis* complementation, promoter binding, and stable transformation in banana. These approaches revealed their conserved but functionally diverse roles in flavonoid pathway regulation [21, 22]. *MusaUP1*, identified through comparative transcriptomics, was strongly upregulated in red bracts and showed progressive induction during the white-to-red transition, supporting its role as a positive regulator of anthocyanin biosynthesis [23, 24]. Phylogenetic placement with SG6 MYBs such as *AtPAP1* and *AcMYB123* [25] further supported this function. Consistently, transient and stable overexpression produced visible pigmentation, and Y1H showed promoter binding to both CHS and ANS, indicating activation of early (EBGs) and late (LBGs) pathway genes. *MusaUP1* induced strong CHS (~200-fold), UFGT, and LAR expression while repressing FLS, suggesting redirection of metabolic flux from flavonols to anthocyanins. Targeted LC–MS profiling, with metabolite identities confirmed via KEGG library screening, revealed broad accumulation of flavanols, proanthocyanidins, and anthocyanins (including peonidin-3,5-diglucoside) in *MusaUP1* overexpressing callus, supporting this flux shift. Growth retardation and partial necrosis reflected phytotoxic consequences of excessive pathway activation, consistent with metabolic imbalance and ROS-linked stress [9). *Arabidopsis myb90* complementation restored anthocyanin and PA levels while reducing DPBA-detectable flavanols, aligning with FLS repression. Y2H assays showed no detectable interaction with *MusaTT8*, and *MusaUP1* displayed pigmentation even in *tt8* mutant seedlings, confirming TT8-independent activation of the anthocyanin pathway.

*MusaUP2*, also highly expressed in red bracts, carries the conserved SG6 [A/S/G/NDV] motif characteristic of *Arabidopsis MYB90/75/113/114* [14]*.* Functional assays showed strong activation of CHS (~1000-fold), DFR, and ANR, and Y1H demonstrated LAR promoter binding, supporting LBG activation. Overexpression across onion, banana, and *Arabidopsis myb90* mutants generated pigmentation and redirected flux towards anthocyanins and PAs. LC–MS profiling similarly revealed flavanol, PA, and peonidin-3,5-diglucoside accumulation. Y2H detected only weak interaction with *MusaTT8*, and complementation in *tt8* was minimal, indicating that, like *MusaUP1*, *MusaUP2* functions largely independent of TT8 but with a narrower LBG activation profile.

*MusaMA8*, [K/R]P[R/Q][PR]R[R/S/T]F motif typical of SG6 [26], showed visible pigmentation upon transient and stable overexpression. Y1H revealed strong binding to the LAR promoter and weaker ANS binding. LC–MS demonstrated that *MusaMA8* alone produced low cyanidin-3-rutinoside levels (3–20 μg/g DW) and accumulated primarily flavanols; however, co-overexpression with *MusaTT8* substantially enhanced cyanidin-3-rutinoside production (100–120 μg/g DW) and enabled prodelphinidin B4 accumulation, indicating MBW-dependent flux enhancement. Y2H confirmed a strong physical interaction between *MusaMA8* and *MusaTT8*, and its inability to complement the *tt8* mutant further established *MusaMA8* as a TT8-dependent LBG activator.

*MusaMA4*, which shares 97% identity with MaMYBA2 (Grand Naine [11]) and possesses the SG5 motif ([V/L][W/I]xxKAxRCT), induced pigmentation in transient and stable assays and upregulated ANS by ~100-fold. Y1H confirmed ANS promoter binding. Notably, *MusaMA4* accumulated high cyanidin-3-rutinoside levels (30–80 μg/g DW) and epicatechin even when overexpressed alone, and LC–MS identified leucodelphinidin and leucopelargonidin, indicating strong TT8-independent activation of LBGs. This was reinforced by strong complementation in *Arabidopsis tt8* seedlings and by Y2H, which showed negligible interaction with *MusaTT8*. These results identify *MusaMA4* as a potent TT8-independent activator of the anthocyanin pathway, particularly in vegetative tissues.

*MbaMA2*, cloned from Rasthali (AAB) and exhibiting high expression in leaves, showed strong FLS promoter binding in Y1H assays and preferentially induced flavonol biosynthesis in transient assays. Stable overexpression of *MbaMA2* alone produced modest cyanidin-3-rutinoside levels (2–15 μg/g DW), accumulated flavanones/flavanols, and supported production of the FLS-derived metabolite dalbergiodin. However, co-overexpression with *MusaTT8* significantly enhanced cyanidin-3-rutinoside (30–50 μg/g DW), supporting TT8-dependent pathway activation. Y2H confirmed strong physical interaction with *MusaTT8*, and a lack of complementation in *tt8* seedlings indicated strict reliance on the MBW complex for anthocyanin induction. These properties resemble the bHLH-dependent activation of *C1* in maize and *AcMYB123* in kiwifruit [25, 27]. Finally, reduced chlorophyll a/b and carotenoid content in MYB overexpression lines likely reflect metabolic reallocation towards flavonoid biosynthesis or transcriptional cross-regulation of plastidial pathways.

Previous studies, such as Busche *et al.* [11], identified key R2R3-MYB activators acting within the MBW complex to regulate late anthocyanin genes, primarily using transient assays. In light of these findings, our study adopts a broader validation strategy, including transcriptomic profiling, promoter binding (Y1H), stable transformation in banana, LC–MS/KEGG-based metabolite identification, and *Arabidopsis* complementation, to clarify both shared and distinct functions of *MusaMYBs* across the flavonoid biosynthetic pathway. While Busche *et al.* reported overlapping activation, the current manuscript indicates more defined roles for anthocyanin- and flavonol-associated MYBs, along with differences in bHLH dependency. In contrast, Rajput *et al.* [10] emphasized activator–repressor interactions influencing proanthocyanidin levels; this study provides complementary promoter-level and metabolite-level evidence specifically supporting anthocyanin biosynthesis. Overall, these findings refine current knowledge and offer a more nuanced view of *MusaMYB* functional diversification.

## Conclusion

This comprehensive analysis of five *Musa* R2R3-MYB TFs reveals their distinct yet overlapping regulatory roles in the flavonoid biosynthetic pathway. *MusaMA8*, *MusaUP1*, and *MusaUP2*, belonging to SG6-type MYBs, primarily activate anthocyanin and proanthocyanidin branches, while *MbaMA2* appears specialized for flavonol biosynthesis. *MbaMA2* shares functional similarity with monocot MYBs that require bHLH co-factors for full activation. Functional validation through overexpression, promoter binding, and *Arabidopsis myb90* complementation supports the conserved roles of these *MusaMYB*s, while also highlighting species-specific differences in their regulatory behaviour in banana.

**Figure 6:**
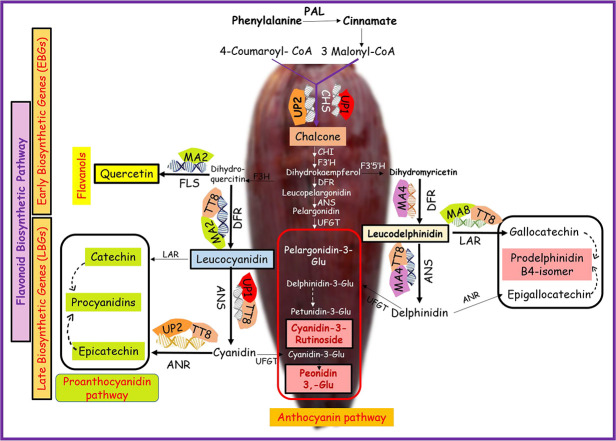
Proposed regulatory roles of five MusaMYB TFs in flavonoid and anthocyanin biosynthesis. Diagram showing the putative positions, target genes, and metabolite branch points regulated by *MusaMYB*s, based on molecular, metabolite, and functional analyses.

This study provides a valuable framework for targeted metabolic engineering to enhance anthocyanin accumulation and antioxidant capacity in *Musa* spp. In the future, it will be particularly interesting to explore how individual MYBs respond to different abiotic or biotic stresses, and whether stress-specific MYB activation contributes to tailored flavonoid accumulation and stress tolerance (Fig. 6).

## Experimental procedures

### Plant materials and growth conditions

*Musa* sp. cv. Rasthali (AAB) was cultivated at the BRIC–National Agri-Food and Biomanufacturing Institute (BRIC-NABI), Mohali, and used for collecting various plant tissues. The harvested samples were immediately frozen in liquid nitrogen and stored at −80°C until further use. *Arabidopsis thaliana* mutant lines were obtained from the *Arabidopsis* Biological Resource Centre (ABRC, USA) with the following SALK accession numbers: *AtMYB90* mutant (SALK_058359C) and *AtTT8* mutant (SALK_030966). *Nicotiana benthamiana*, *A. thaliana* Col-0 (wild-type), and mutant lines were grown under controlled conditions in growth chambers. *Allium cepa* (white onion) bulbs were sourced from the local market for transient overexpression assays. Phenotypic observations for each construct were confirmed across at least three independent transgenic events, which consistently exhibited comparable pigmentation intensity and transcript levels, indicating reproducible effects of transgene expression (Supplementary File S3).

### RNA-Seq analysis and gene identification

Transcriptome analysis was performed using the leaf and flower bract tissue samples of Rasthali (AAB) in two and three biological replicates, respectively. Principal component analysis revealed the clustering among biological replicates with minor intragroup variation (PC2: 0.63% variance) and strong tissue-specific variance in the transcriptomic data (PC1: 98.59% variance) (Fig. S1). Total RNA was isolated from leaf tissue and anthocyanin-rich flower bract (red-purple coloured) of banana using the Concert™ Plant RNA Reagent (Invitrogen, USA). The extracted RNA was cleaned up using RNeasy Plant Mini Kit (Qiagen, Germany), and gDNA contamination was removed using on-column DNase digestion (Qiagen, Cat. No. 79254). Before preparing the RNA library, total RNA was subjected to ribosomal RNA depletion. At every step, messenger RNA (mRNA) integrity, enrichment, fragmentation, and library size distribution were analysed using a bioanalyser profile. After confirmation of the library size by gel electrophoresis, sequencing was carried out using paired-end reads on an Illumina HiSeq 2500 platform. Approximately 5 GB of raw data per sample was generated with a minimum of 50 million reads per sample. Further, raw reads were subjected to adapter trimming after quality control using the fastp tool [28]. The clean reads were aligned to the banana reference genome using the HISAT2 programme [29]. Gene expression levels were quantified, and differentially expressed genes were determined using HTSeq-count [30] and the DESeq2 [31] programmes, respectively. Results were visualized through heat maps, and downstream analysis, including pathway enrichment and gene ontology (GO) classification, was performed. Upregulated MYB TFs were identified in the coloured bract in comparison to the leaf in banana.

In parallel, homology-based BLAST search was also performed using annotated MYB sequences from maize (*Z. mays*), rice (*O. sativa*), and *Arabidopsis* (*A. thaliana*) as queries against the banana genome hub (https://banana-genome-hub.southgreen.fr/) and Ensembl Plants (https://plants.ensembl.org/index.html) to further identify anthocyanin biosynthesis-regulating MYBs. All three previously reported positive regulators of the anthocyanin biosynthesis pathway from *Musa acuminata* cultivar Grand Naine were also considered [11].

### Phylogeny, motif analysis, and gene architecture

Protein sequences of 60 upregulated *MaMYB*s identified through transcriptomic data, three identified through BLAST search, and 60 previously annotated R2R3 MYBs of dicots and monocots (Table S2) were considered (https://web.expasy.org/translate/). Phylogenetic analysis was conducted using MEGA version 12 [32] to construct a neighbour-joining tree based on 123 aligned MYB protein sequences, with 1000 bootstrap replicates to assess the reliability of tree topology. Based on phylogenetic analyses, five putative anthocyanin biosynthesis regulators—*MbaMA2*, *MusaMA4*, *MusaMA8*, *MusaUP1*, and *MusaUP2* (Accession IDs provided in Table S1)—were selected for further analyses. Protein sequences of selected R2R3-MYBs were aligned. The tree was visualized with ITOL v7 (https://itol.embl.de/). Multiple sequence alignment was performed using MultAlin [33] to identify the conserved domains and motifs (http://multalin.toulouse.inra.fr/multalin/). Further, conserved motifs were predicted using MEME suite Version 5.5.8 [34] using default parameters, and their distribution across MYB proteins was visualized (https://meme-suite.org/meme/tools/meme). To further represent conserved amino acids within key motifs, WebLogo was employed to generate a sequence logo (https://weblogo.berkeley.edu/logo.cgi). Also, gene structure analysis carried out using Gene Structure Display Server (GSDS) revealed the exon–intron organization of selected *MaMYB* genes, providing insights into their structural variation and evolutionary conservation (Fig. S2a).

### Confirmation of transcriptome data

To validate the differential expression of the selected five *MYB* genes (*MbaMA2*, *M usaMA4*, *MusaMA8*, *MusaUP1*, *MusaUP2*, and *MusaTT8*) identified from the transcriptomic data, quantitative real-time PCR (qRT-PCR) was performed. Total RNA from three tissues, i.e., leaf, white bract, and red bract of Rasthali (AAB), was reverse transcribed into first-strand cDNA using Revert Aid cDNA Synthesis Kit (Thermo Fisher Scientific). qRT-PCR was performed using diluted cDNA and 2× SYBR Green Supermix (SYBR Green PCR kit, BioRad) on a real-time PCR system (BioRad, USA). Each reaction mixture contained 1:10 diluted cDNA, 2 pmol of each gene-specific forward and reverse primer, and 2× SYBR green PCR master mix. Ct values were normalized against the expression of the reference gene. Expression of MaEF1α (GSMUA_AchrT02020_001) served as the internal reference gene, and relative transcript levels were calculated using the comparative Ct (2^−ΔΔCt^) method [35].

### Molecular cloning and vector construction for plant transformation

Full-length coding sequences of five *Musa MYBs* (*MbaMA2*, *MusaMA4*, *MusaMA8*, *MusaUP1*, *MusaUP2*) and *MusabHLH* (*MusaTT8*) were amplified from red bract and leaf cDNA of the banana cv. Rasthali (AAB) using gene-specific primers (Table S3). *AcMYB1* was also cloned from onion bulb cDNA as a positive control for transient assays. PCR amplification was performed with Phire Hot Start II DNA Polymerase (Thermo Scientific), and purified products (QIAquick Gel Extraction Kit, Qiagen) were ligated into the pJET 1.2 vector (CloneJET PCR cloning Kit, Thermo Fisher). Recombinant plasmids were transformed into *Escherichia coli* DH5α, screened, and validated by sequencing (Applied Biosystems, 3730xl). Sequences were confirmed using NCBI-BLAST. For functional assays, MusaMYBs and MusaTT8 were cloned into a modified pCambia 1301 vector [36], with MYBs under the *Z. mays* polyubiquitin promoter and *GUS* under the CaMV 35S promoter (Fig. 2a). Transient overexpression was tested in onion bulb epidermis with/without *MusaTT8* to evaluate anthocyanin pathway activation before stable transformation in banana ECS.

### Transient overexpression of *MusaMYBs* in monocot *A. cepa*

Transient expression of MusaMYB TFs was performed using white onion bulbs (local market). *Agrobacterium tumefaciens* harbouring overexpression MYB-constructs (as mentioned above) and *MusaTT8* (for co-transformation) were grown to OD_600_ 0.6–0.8, resuspended in infiltration buffer (10 mM MES, pH 5.6, 10 mM MgCl_2_, 100 μM acetosyringone), and used to infect square-shaped onion slices. For co-expression, equal volumes of MusaMYBs and MusaTT8 *Agrobacterium* cultures were mixed before infection. Infected slices were dried on sterile filter papers and incubated on MS medium supplemented with acetosyringone (100 μM) plates at 28°C for 48–72 h. For each construct, five to eight slices from the same bulb lot were used, with two to three replicate plates per treatment; representative replicates are shown in Supplementary Fig. S3.

### Y1H and Y2H assay for interaction studies

To confirm the binding of *MusaMYBs* (MbaMA2, MusaMA4, MusaMA8, MusaUP1, MusaUP2) to the promoter region of anthocyanin biosynthesis genes, a Y1H assay was performed. Promoter regions of *CHS*, *ANS*, *ANR*, *UFGT*, *LAR*, *FLS*, and *TT8* were amplified from the banana cv. Rasthali (AAB) and cloned upstream of the *HIS3* reporter gene in the pHIS-1 vector (Supplementary File S4). Sequences were verified by Sanger sequencing and analysed for MYB cis-elements using PlantCARE [37]. *MusaMYB*s were cloned into pGAD-C1 as effectors fused with the GAL4 activation domain. Reporter plasmids were linearized with NcoI or AflII and transformed into *Saccharomyces cerevisiae* Y1HGold using the PEG–lithium acetate method (Clontech). To minimize leaky *HIS3* expression, the optimal 3-amino-1,2,4-triazole (3-AT) concentration (0 mM–30 mM) was determined for each reporter strain (Supplementary Fig. S5). Subsequently, effector plasmids were introduced into seven promoter–reporter strains, and interactions were evaluated by yeast growth on SD–His medium. Binding specificity was confirmed using double dropout (SD/−His/−Leu) medium, with empty pGAD-C1 serving as a negative control. Protein–protein interactions between *MusaMYB* transcription factors and *MusaTT8* were examined using the GAL4-based Matchmaker Yeast-two Hybrid system (Clontech). Full-length *MusaTT8* was cloned into the pGBDC1 vector as bait (BD-*MusaTT8*), and *MusaMYBs* were cloned into pGADC1 [38] as prey (AD-MusaMYBs). Constructs were co-transformed into *S. cerevisiae* strain Y2H Gold using the lithium acetate/PEG method, and transformants were first selected on double dropout medium (SD/−Leu/−Trp; DDO). Well-grown colonies were inoculated in liquid DDO, adjusted to an OD_600_ of 0.8, and subjected to serial 10-fold dilutions. Ten microliters of each dilution were spotted onto quadruple dropout (−Leu/−Trp/−His/−Ade; QDO) medium to assess protein–protein interactions. Plates were incubated at 28°C for 3–4 days. Growth on QDO plates indicates a positive interaction between MusaMYBs and MusaTT8, while no growth on QDO was observed for negative controls (AD-empty/BD-*MusaTT8*).

### Complementation and histochemical analysis in *Arabidopsis*

Functional complementation of *Musa*MYBs and *Musa*TT8 was performed in *Arabidopsis myb90/tt8* mutants. Full-length CDS of banana genes were cloned into Pri101-AN under the CaMV 35S promoter and introduced into *A. tumefaciens* (EHA105) for floral dip transformation [39]. T_1_ seeds were surface sterilized (2% sodium hypochlorite), stratified at 4°C for 2 days, and germinated in growth chambers. Seedlings were analysed 4–5 days postgermination. For anthocyanin induction, Col-0, *Atmyb90*, *Attt8*, and complemented lines were germinated on ½ MS medium containing 4% sucrose and 3 ppm norflurazon. Anthocyanin pigmentation was examined under a stereomicroscope (Leica). Proanthocyanidin accumulation was assessed by DMACA staining and quantified against a catechin standard curve, expressed as catechin equivalents (μg g^−1^ DW) [10,40]. Flavanols were detected by DPBA staining with Triton X-100, and fluorescence was visualized using UV microscopy [41,42].

### Generation of transgenic banana plants

All the recombinant vectors confirmed via Sanger sequencing were then transformed into the *Agrobacterium* strain EHA105. For the generation of transgenic lines, banana cultivar Rasthali (AAB) ECS was transformed using *A. tumefaciens* strain EHA105 harbouring a desired combination of *MusaMYBs* (*MbaMA2*, *MusaMA4*, *MusaMA8*, *MusaUP1*, *MusaUP2*) with and without *MusaTT8* TF as per the previously published protocol [43]. Generated shoots were stained with GUS histochemical stain to confirm the integration of T-DNA in genomic DNA. In the case of co-transformation of *MYB*s and *bHLH* overexpression cassette, T-DNA insertion of both was further confirmed by PCR amplification using gene-specific and one vector-specific primer (Supplementary Fig. S7). Confirmed plants were shifted to MS medium tubes supplemented with 2 mg/l 6-benzylaminopurine and further multiplied, followed by rooting on MS supplemented with 1 mg/l naphthalene acetic acid under controlled conditions [44].

### Expression profiling of transgenic banana plants

To further validate the transactivation of anthocyanin biosynthesis pathway genes, total RNA was isolated from the confirmed transgenic lines. cDNA was prepared from the DNase-treated purified RNA using the revert aid cDNA synthesis kit (Thermo Fisher Scientific). Prepared cDNA was diluted, and qRT-PCR was performed using SYBR green chemistry. Primers used for expression profiling of pathway genes are listed in Table S3. *MaEF1α* was used as an internal control to normalize qRT-PCR data, as mentioned in the above section. The data were expressed as a relative fold change value in comparison to control Rasthali (AAB) plants and displayed using GraphPad Prism 9.0.3 software. Phenotypic observations for each construct were confirmed across at least three independent transgenic events, which consistently exhibited comparable pigmentation intensity and transcript levels, indicating reproducible effects of transgene expression.

### Biochemical analysis of banana transgenic plants

Leaf tissues exhibiting visible anthocyanin accumulation were harvested and immediately flash frozen in liquid nitrogen. The samples were subsequently lyophilized, and 10 mg/ml of the lyophilized tissue (dried leaves) was used for biochemical analyses. Methanolic extracts were prepared by homogenising the dried tissue in 1% acidified 90% methanol, followed by overnight incubation in the dark at room temperature. The extracts were then centrifuged at 12000 rpm for 5 min. Total anthocyanin content was estimated following the method of Urbstaite *et al.* [45], while total flavonoid content and DPPH free radical scavenging activity were determined according to reference [46]. Absorbance was measured at respective wavelengths of each assay using a UV–VIS spectrophotometer (Spectramax i3x, Molecular Devices, USA). Chlorophyll a, chlorophyll b, and total chlorophyll were quantified following the extraction and spectrophotometric procedures described by Kaur *et al.* [47]. The estimation was performed using their standardized protocol with absorbance readings at appropriate wavelengths.

### LC–MS/MS analysis

Untargeted metabolite profiling was performed using a Waters SYNAPT-XS HDMS system (Mode: DBA064, UK) with a UPLC ACQUITY H-class separation module, controlled by MassLynx software (version 4.2). Chromatographic separation was employed on a Waters Acquity BEH C18 column (2.1 × 100 mm, 1.7 μm). For each run, 5 μl of the sample was injected. The mobile phase comprised solvent A (0.1% formic acid in LC–MS grade water) and solvent B (0.1% formic acid in acetonitrile). The gradient programme was as follows: 95% A/5% B for 0–5 min; linear change to 10% A/90% B from 5 to 30 min, held until 35 min; reequilibration to 95% A/5% B at 36 min, maintained until 45 min. Flow rate was 0.2 ml/min with curve type 6 transitions. Data were acquired in positive ESI mode using multiple reaction monitoring (MRM) at unit resolution. Source parameters included desolvation gas flow 950 l/h, cone gas flow 50 l/h, desolvation temperature 550°C, source temperature 120°C, capillary voltage 3.22 kV, cone voltage 50 V, collision energy 4 eV, and source offset 80 V. LC–MS/MS library screening was conducted at SAIF, Panjab University, Chandigarh.

### Targeted quantification of key flavonoids

Targeted LC–MS/MS analysis of cyanidin-3-ritinoside, epicatechin, and kaempferol was conducted using an ACQUITY UPLC system (Waters) connected to a triple quadrupole mass spectrometer equipped with a turbo spray ion source. Data were acquired in positive-ion MRM mode. Chromatographic separation was achieved under a 15.015-min gradient at a flow rate of 0.2 ml/min using mobile phase A (water + 0.1% formic acid) and mobile phase B (acetonitrile). The gradient schedule was 0–1.0 min, 92%–90% A; 1.0–4.5 min, 90%–85% A; 4.5–8.8 min, 85%–75% A; 8.8–12.5 min, 75%–50% A; and 12.5–15.0 min, 50%–5% A. Ionization and acquisition parameters were optimized for maximum sensitivity and precision. The curtain gas was maintained at 35 psi, with the collision gas set to medium. Ion source gases GS1 and GS2 were both kept at 55 psi, and the ion spray voltage was 5000 V. The source temperature was 550°C. Additional MS settings included declustering potential (DP) 82 V, entrance potential (EP) 6 V, collision energy (CE) 47 V, and collision cell exit potential (CXP) 15 V. The monitored MRM transitions were m/z 595 → 287 for *cyanidin-3-rutinoside*, m/z 287 → 269 for *kaempferol*, and m/z 289 → 245 for *epicatechin*, each with a dwell time of 10 ms. The full 15.015-min run generated 8576 acquisition cycles per sample, with a total scan cycle time of 0.105 s.

## Supplementary Material

Web_Material_uhaf361

## Data Availability

Data underlying this article are available in the article and in its online supplementary material.
