# Supplementary material for: A coordinated network of MYB regulators orchestrates anthocyanin biosynthesis in banana
Source: Hortic Res. 2026 Jan 13;13(6):uhaf361. doi: 10.1093/hr/uhaf361 (PMC13273576; doi:10.1093/hr/uhaf361)

## Slide 1
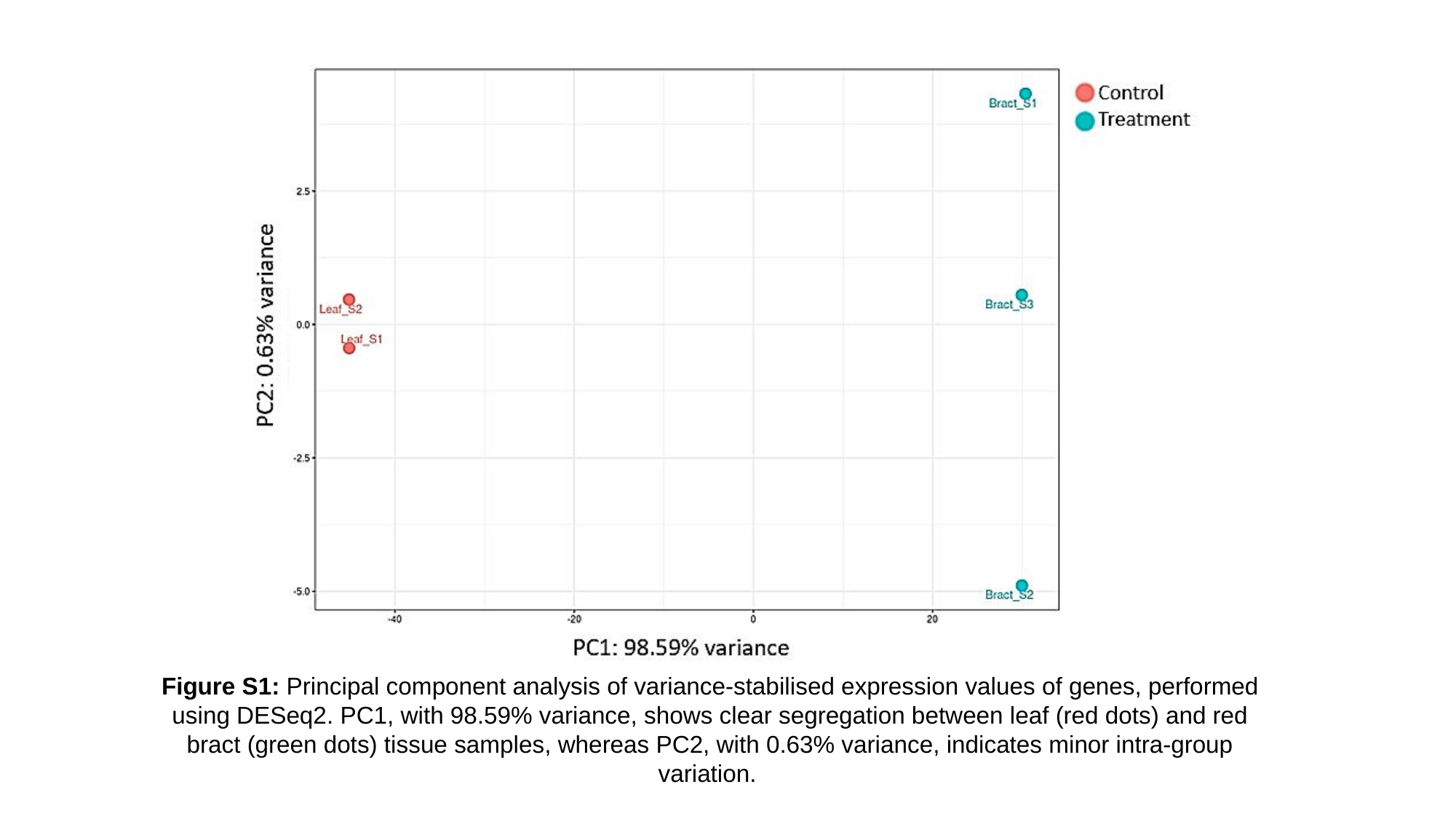

Figure S1: Principal component analysis of variance-stabilised expression values of genes, performed using DESeq2. PC1, with 98.59% variance, shows clear segregation between leaf (red dots) and red bract (green dots) tissue samples, whereas PC2, with 0.63% variance, indicates minor intra-group variation.

## Slide 2
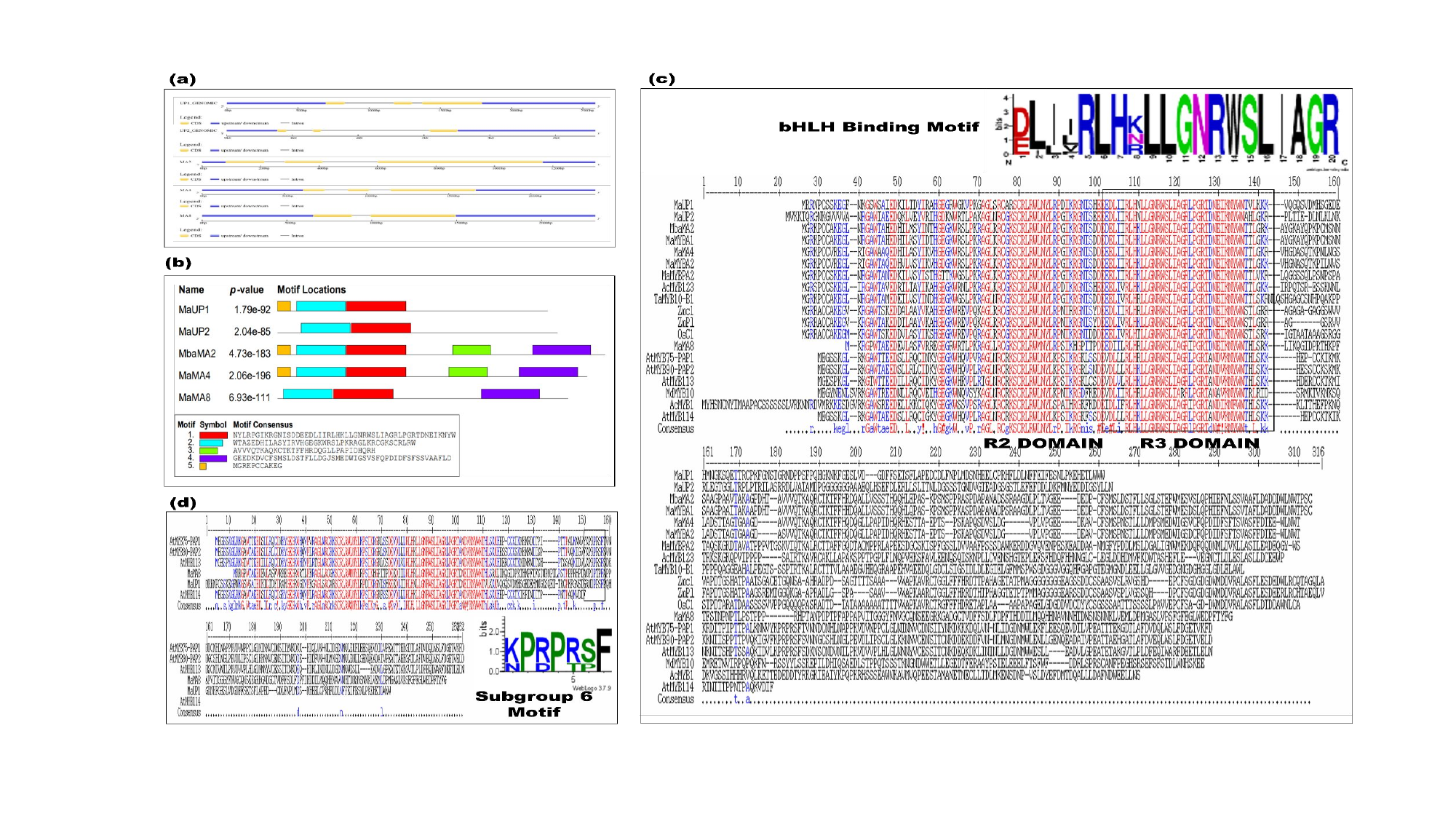

## Slide 3
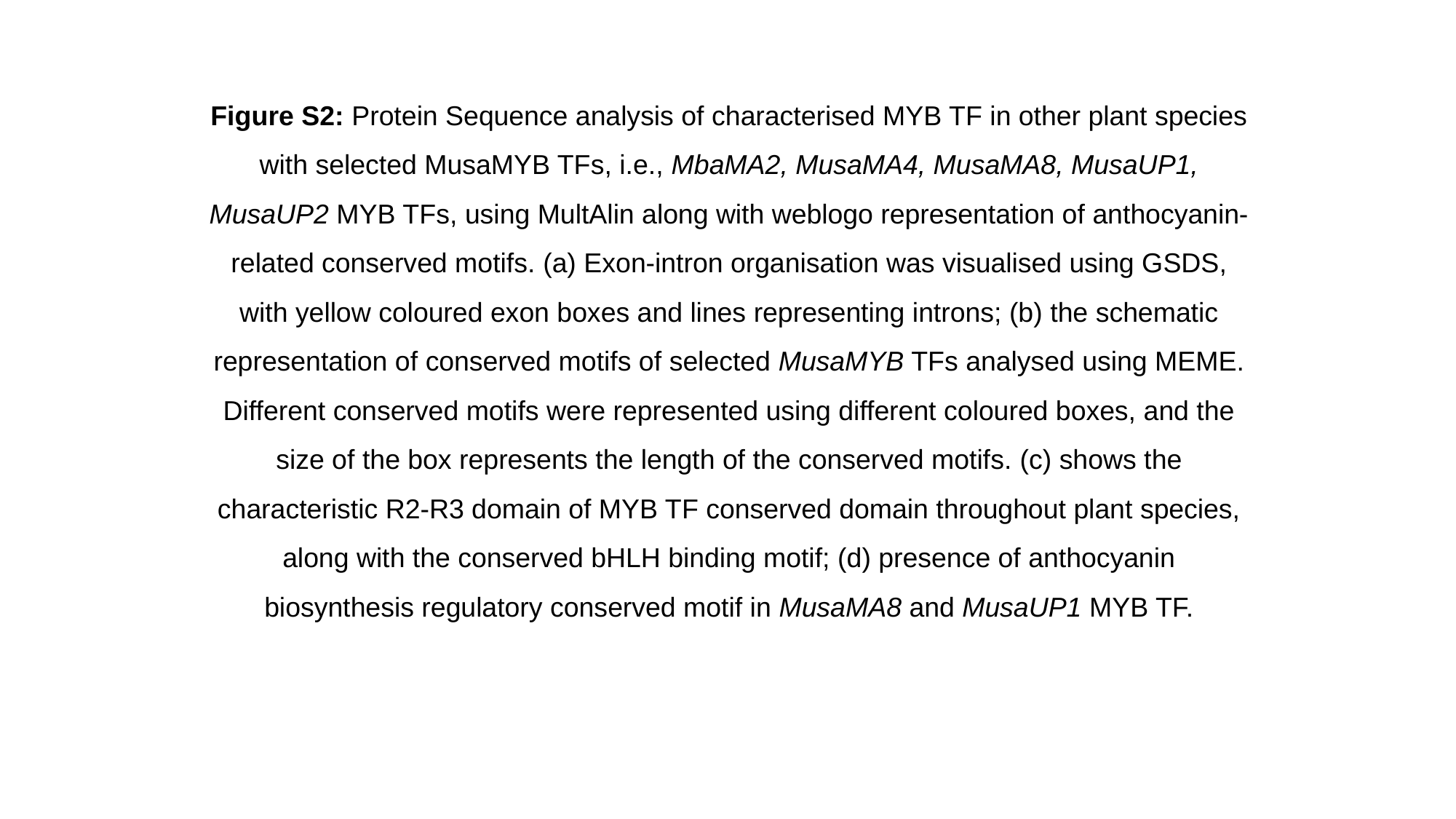

Figure S2: Protein Sequence analysis of characterised MYB TF in other plant species with selected MusaMYB TFs, i.e., MbaMA2, MusaMA4, MusaMA8, MusaUP1, MusaUP2 MYB TFs, using MultAlin along with weblogo representation of anthocyanin-related conserved motifs. (a) Exon-intron organisation was visualised using GSDS, with yellow coloured exon boxes and lines representing introns; (b) the schematic representation of conserved motifs of selected MusaMYB TFs analysed using MEME. Different conserved motifs were represented using different coloured boxes, and the size of the box represents the length of the conserved motifs. (c) shows the characteristic R2-R3 domain of MYB TF conserved domain throughout plant species, along with the conserved bHLH binding motif; (d) presence of anthocyanin biosynthesis regulatory conserved motif in MusaMA8 and MusaUP1 MYB TF.

## Slide 4
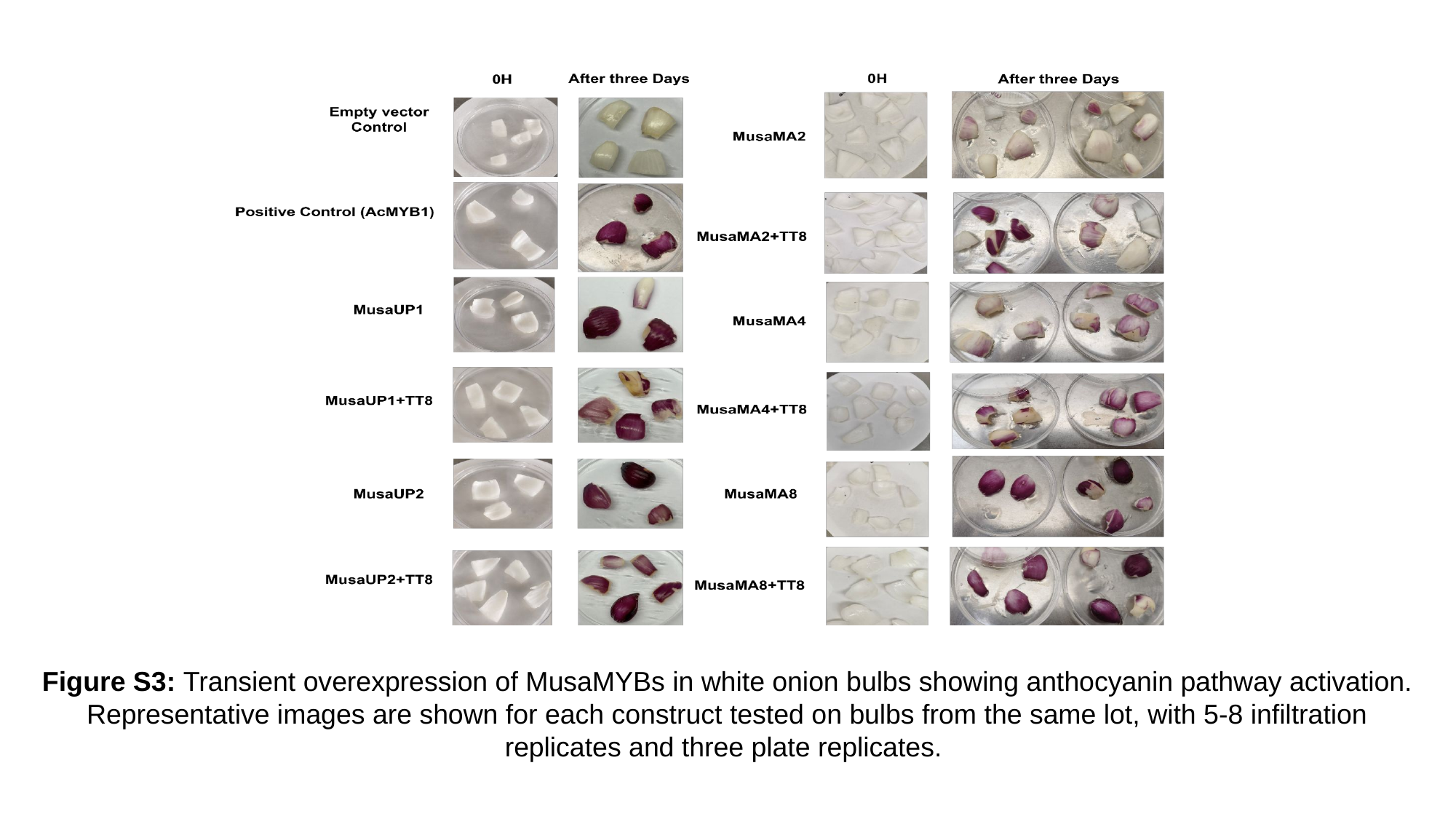

Figure S3: Transient overexpression of MusaMYBs in white onion bulbs showing anthocyanin pathway activation. Representative images are shown for each construct tested on bulbs from the same lot, with 5-8 infiltration replicates and three plate replicates.

## Slide 5
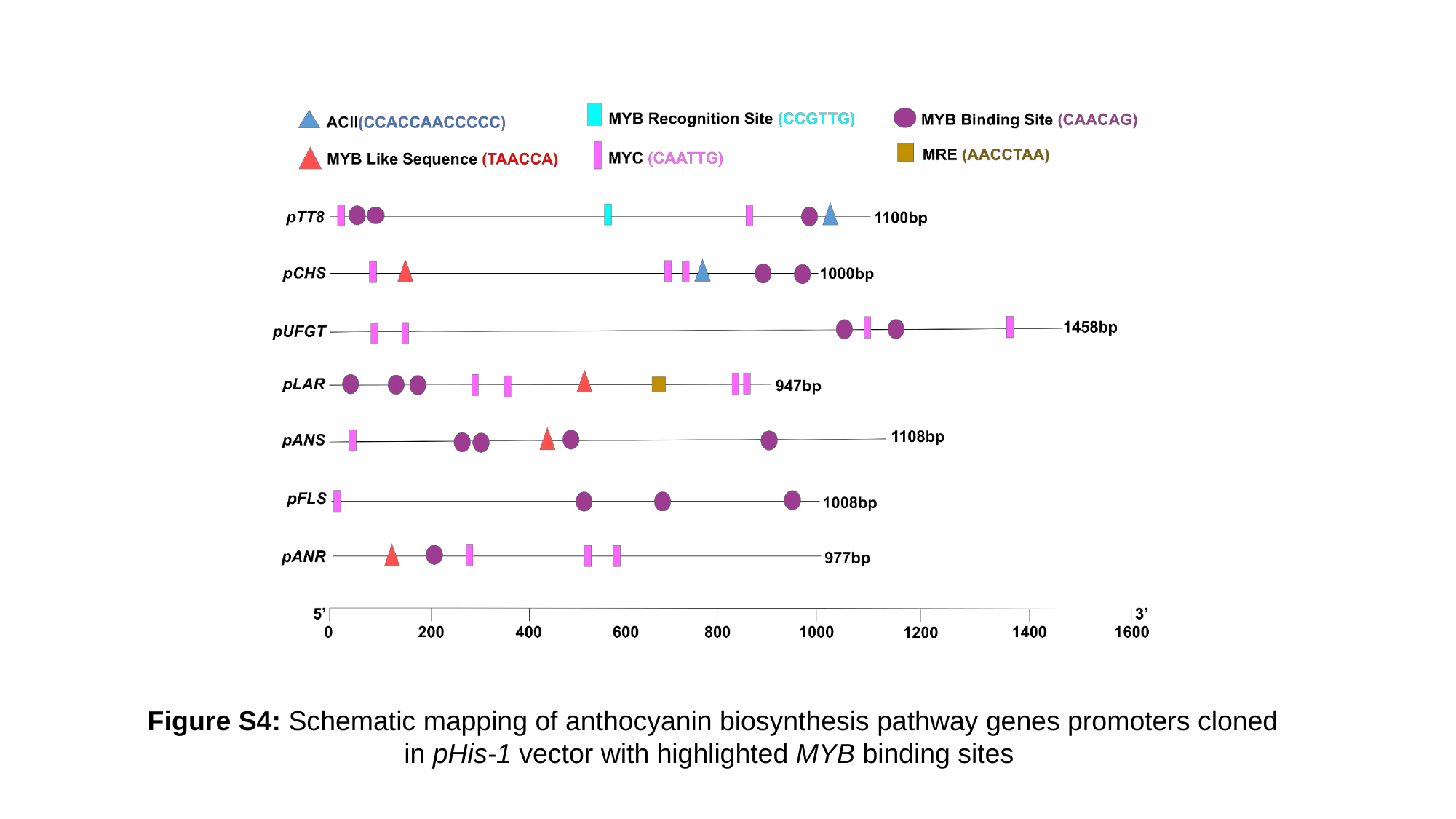

Figure S4: Schematic mapping of anthocyanin biosynthesis pathway genes promoters cloned in pHis-1 vector with highlighted MYB binding sites

## Slide 6
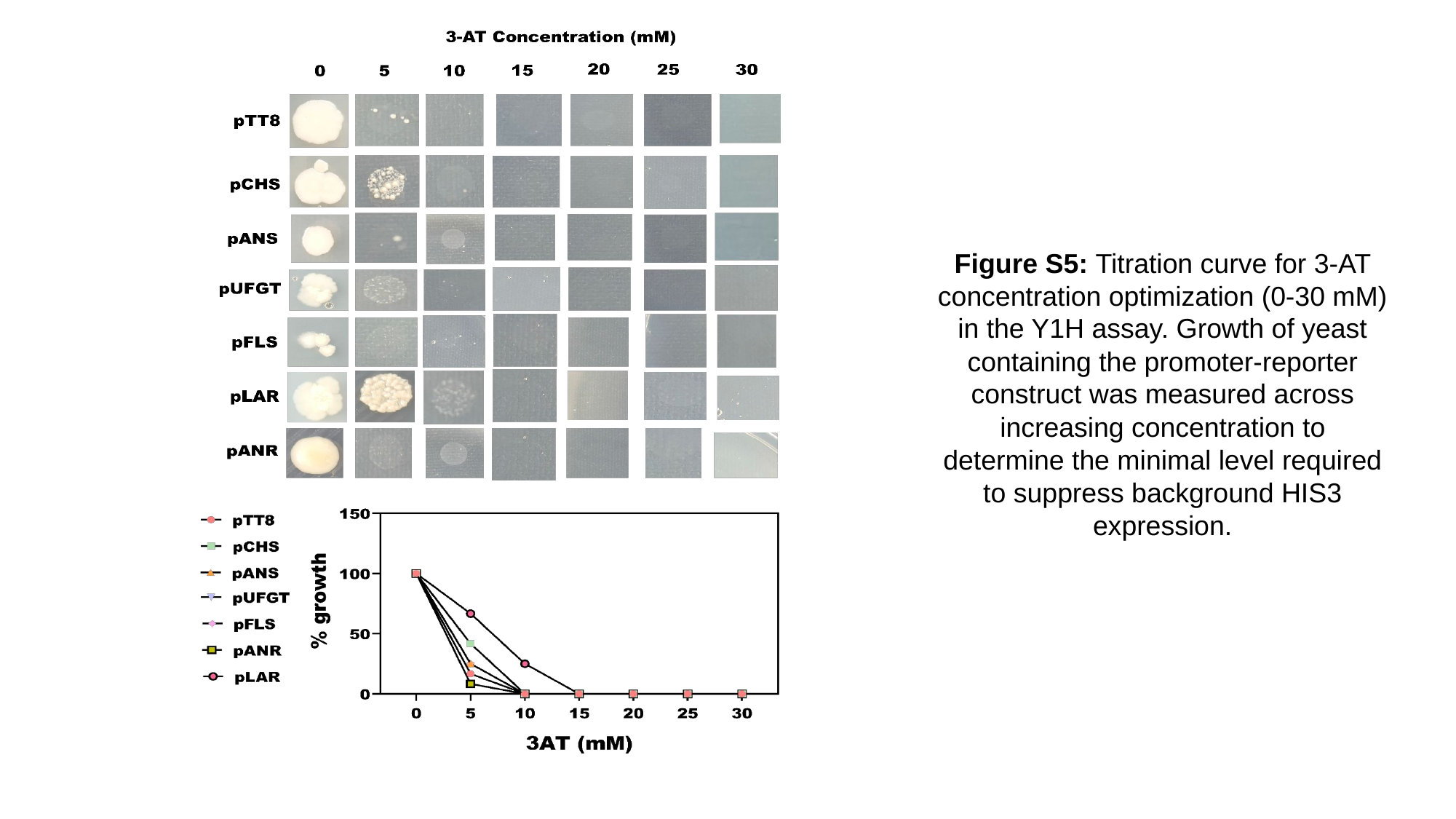

Figure S5: Titration curve for 3-AT concentration optimization (0-30 mM) in the Y1H assay. Growth of yeast containing the promoter-reporter construct was measured across increasing concentration to determine the minimal level required to suppress background HIS3 expression.

## Slide 7
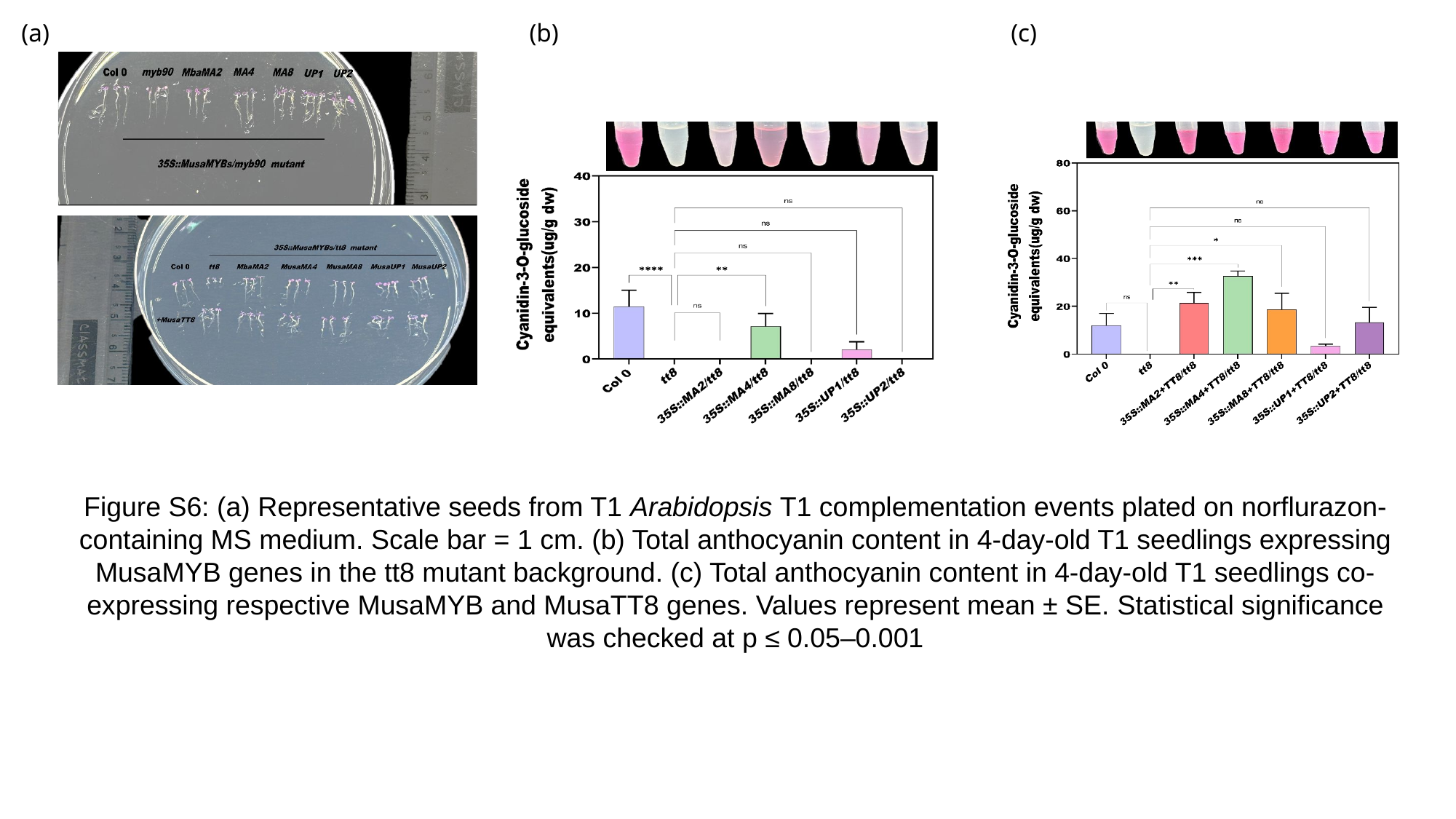

(a)
(b)
(c)
Figure S6: (a) Representative seeds from T1 Arabidopsis T1 complementation events plated on norflurazon-containing MS medium. Scale bar = 1 cm. (b) Total anthocyanin content in 4-day-old T1 seedlings expressing MusaMYB genes in the tt8 mutant background. (c) Total anthocyanin content in 4-day-old T1 seedlings co-expressing respective MusaMYB and MusaTT8 genes. Values represent mean ± SE. Statistical significance was checked at p ≤ 0.05–0.001

## Slide 8
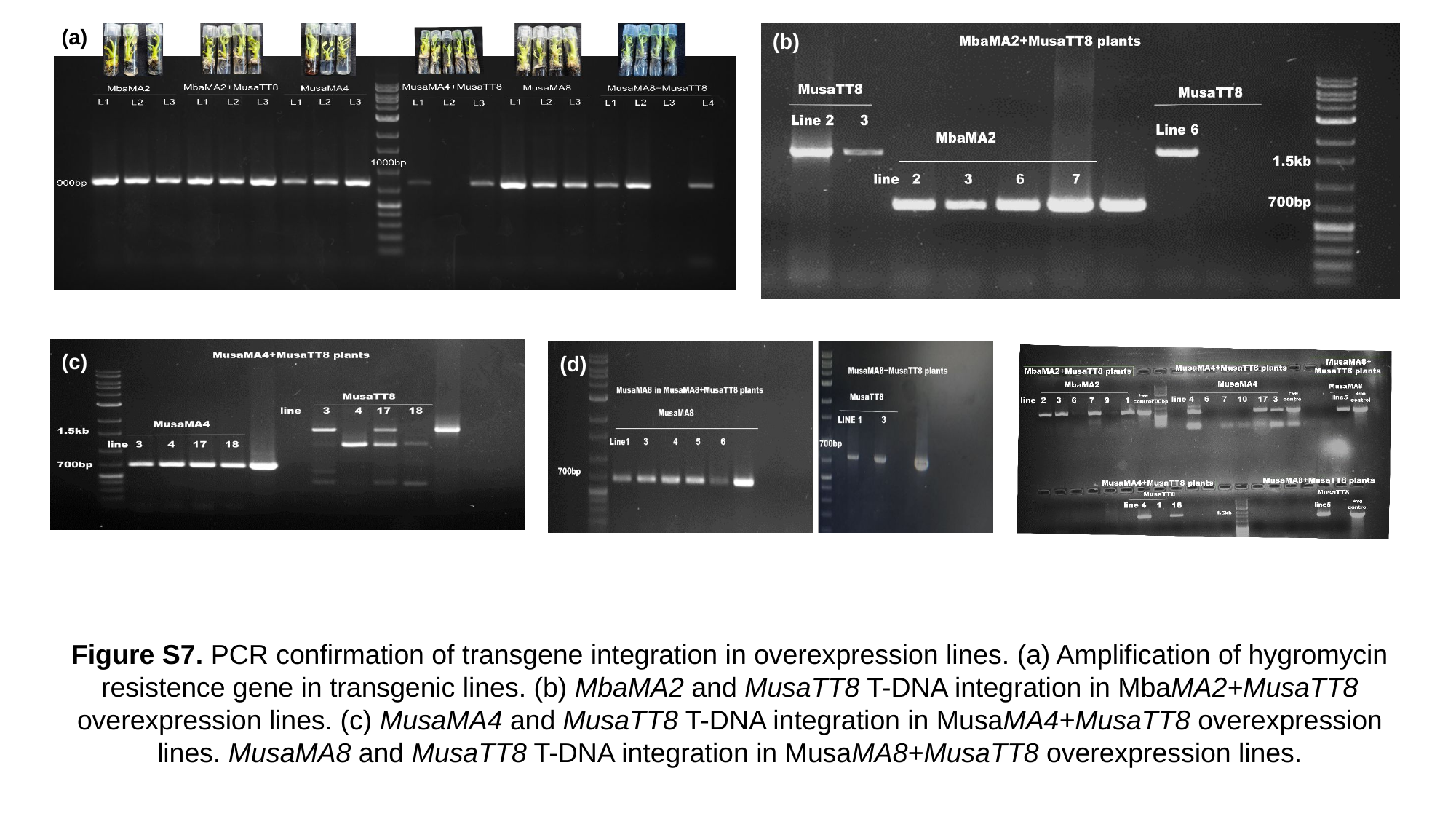

(a)
(b)
(c)
(d)
Figure S7. PCR confirmation of transgene integration in overexpression lines. (a) Amplification of hygromycin resistence gene in transgenic lines. (b) MbaMA2 and MusaTT8 T-DNA integration in MbaMA2+MusaTT8 overexpression lines. (c) MusaMA4 and MusaTT8 T-DNA integration in MusaMA4+MusaTT8 overexpression lines. MusaMA8 and MusaTT8 T-DNA integration in MusaMA8+MusaTT8 overexpression lines.

## Slide 9
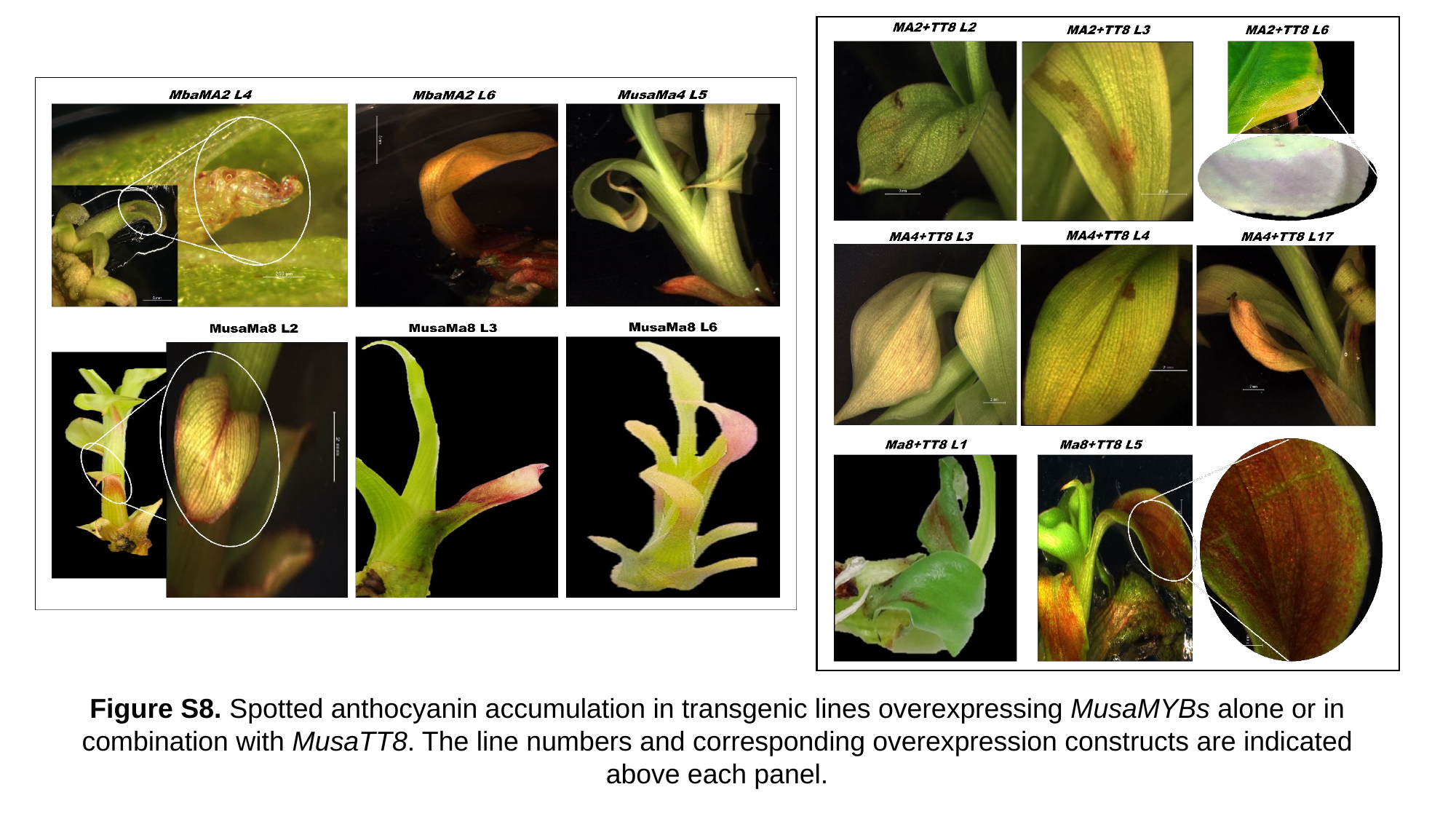

Figure S8. Spotted anthocyanin accumulation in transgenic lines overexpressing MusaMYBs alone or in combination with MusaTT8. The line numbers and corresponding overexpression constructs are indicated above each panel.

## Slide 10
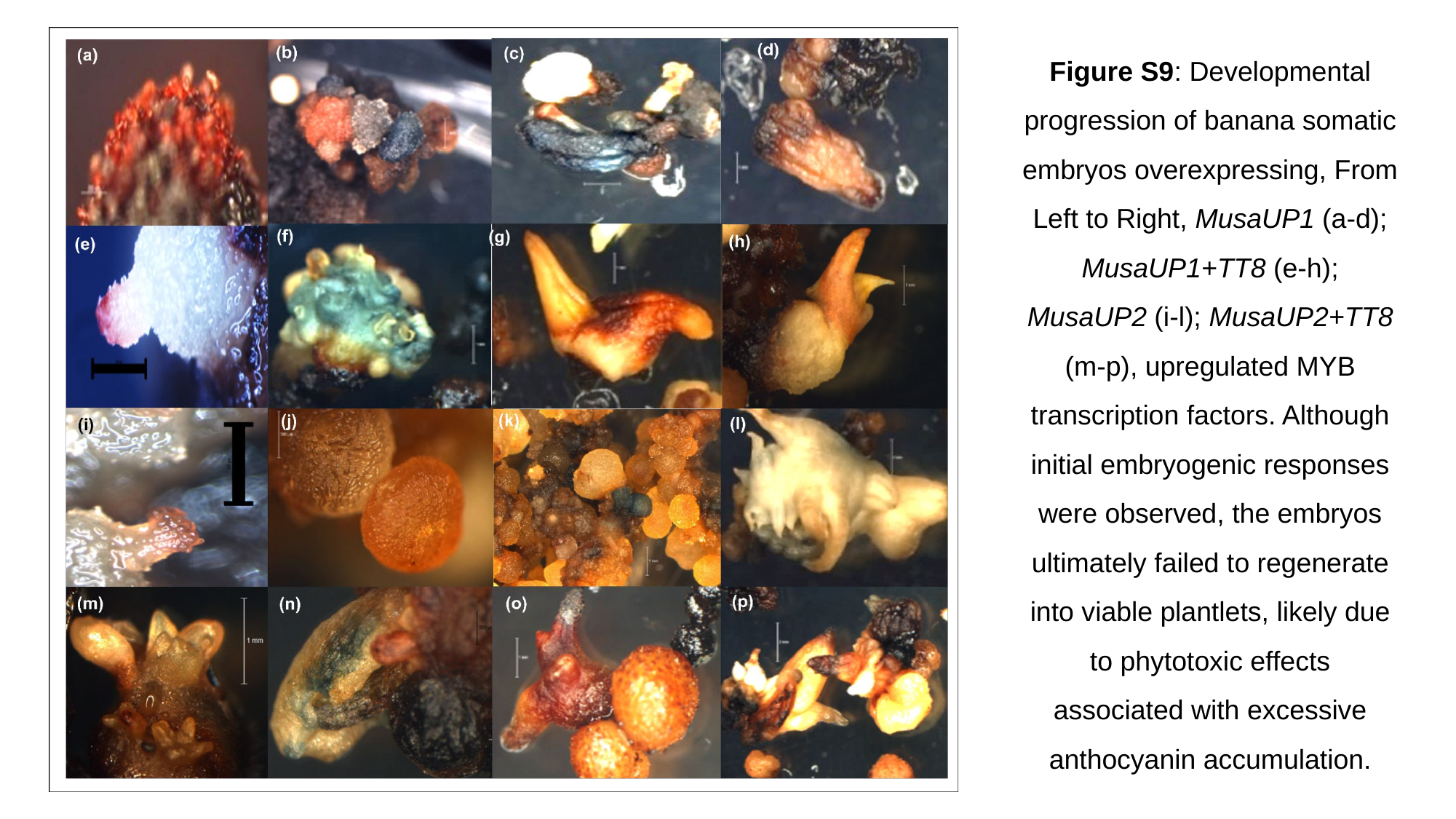

Figure S9: Developmental progression of banana somatic embryos overexpressing, From Left to Right, MusaUP1 (a-d); MusaUP1+TT8 (e-h); MusaUP2 (i-l); MusaUP2+TT8 (m-p), upregulated MYB transcription factors. Although initial embryogenic responses were observed, the embryos ultimately failed to regenerate into viable plantlets, likely due to phytotoxic effects associated with excessive anthocyanin accumulation.

## Slide 11
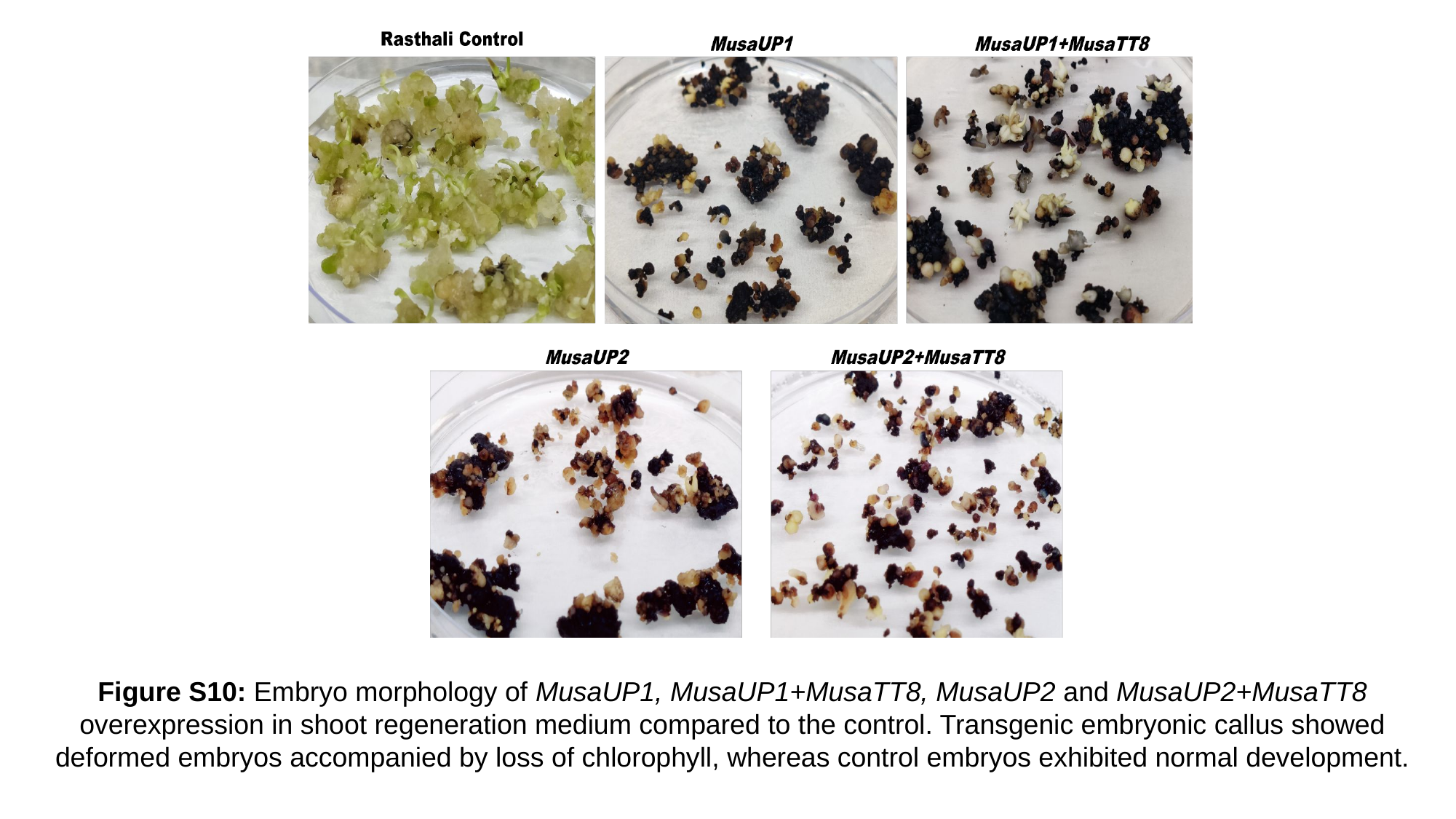

Figure S10: Embryo morphology of MusaUP1, MusaUP1+MusaTT8, MusaUP2 and MusaUP2+MusaTT8 overexpression in shoot regeneration medium compared to the control. Transgenic embryonic callus showed deformed embryos accompanied by loss of chlorophyll, whereas control embryos exhibited normal development.

## Slide 12
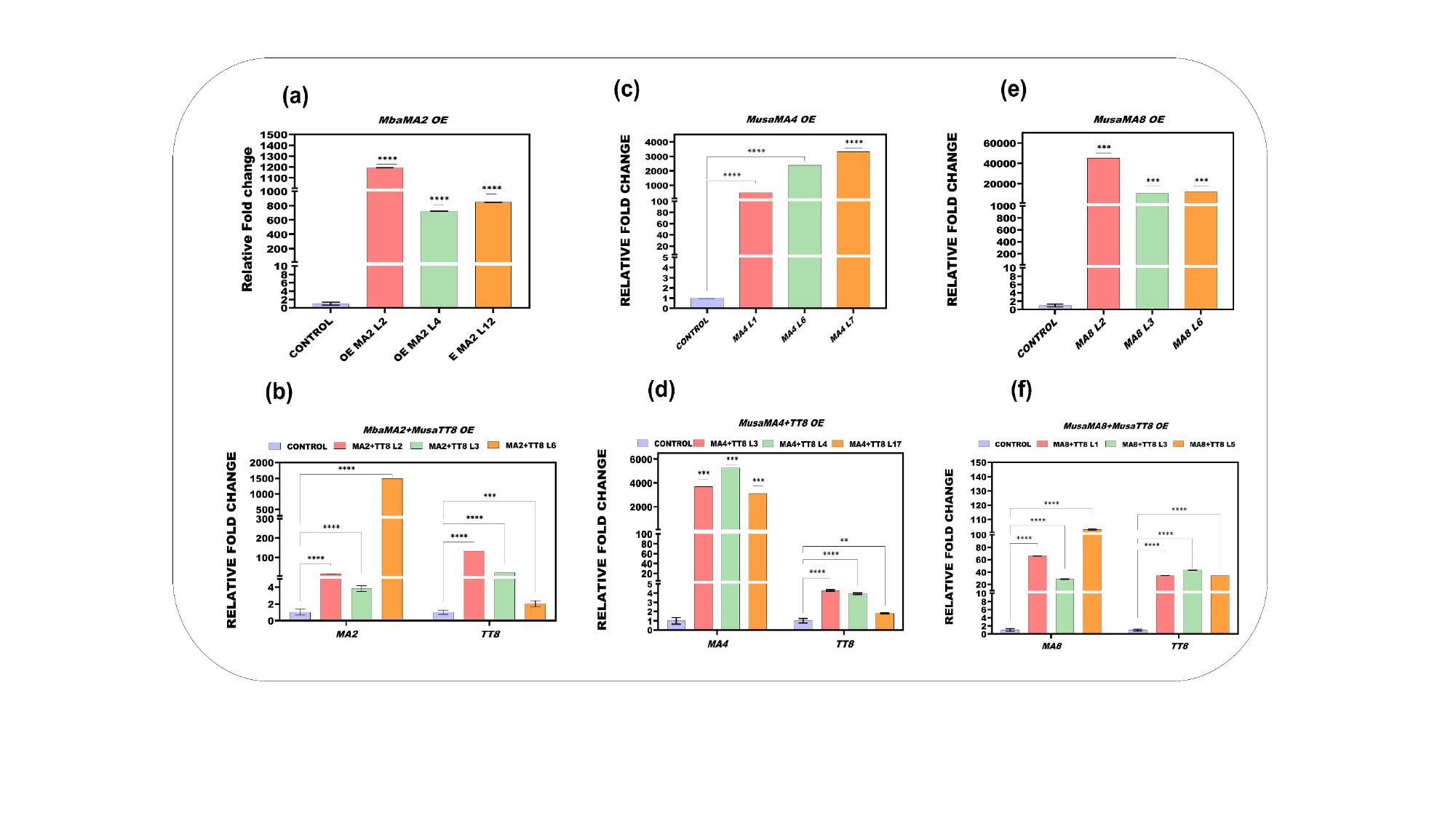

## Slide 13
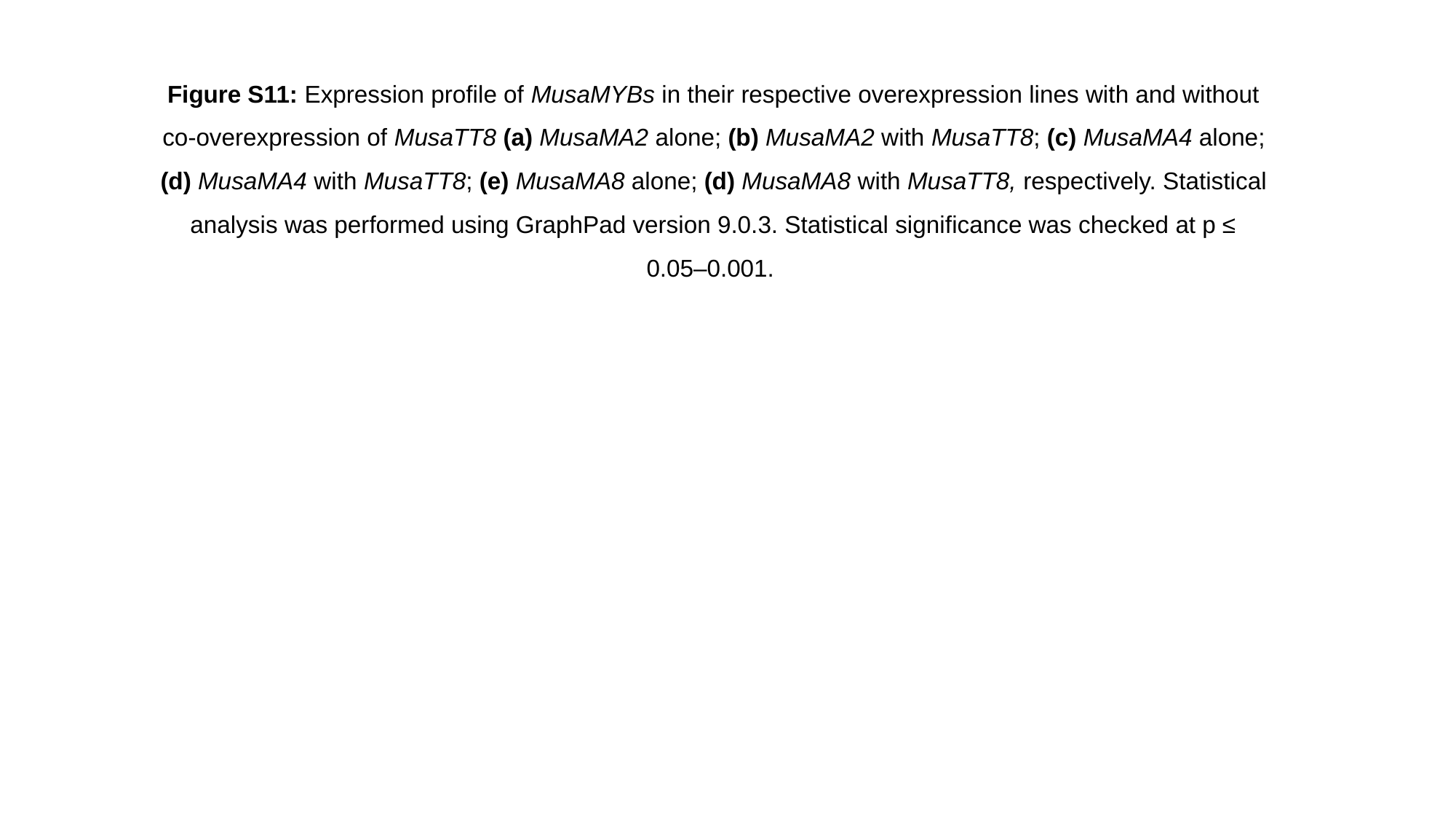

Figure S11: Expression profile of MusaMYBs in their respective overexpression lines with and without co-overexpression of MusaTT8 (a) MusaMA2 alone; (b) MusaMA2 with MusaTT8; (c) MusaMA4 alone; (d) MusaMA4 with MusaTT8; (e) MusaMA8 alone; (d) MusaMA8 with MusaTT8, respectively. Statistical analysis was performed using GraphPad version 9.0.3. Statistical significance was checked at p ≤ 0.05–0.001.

## Slide 14
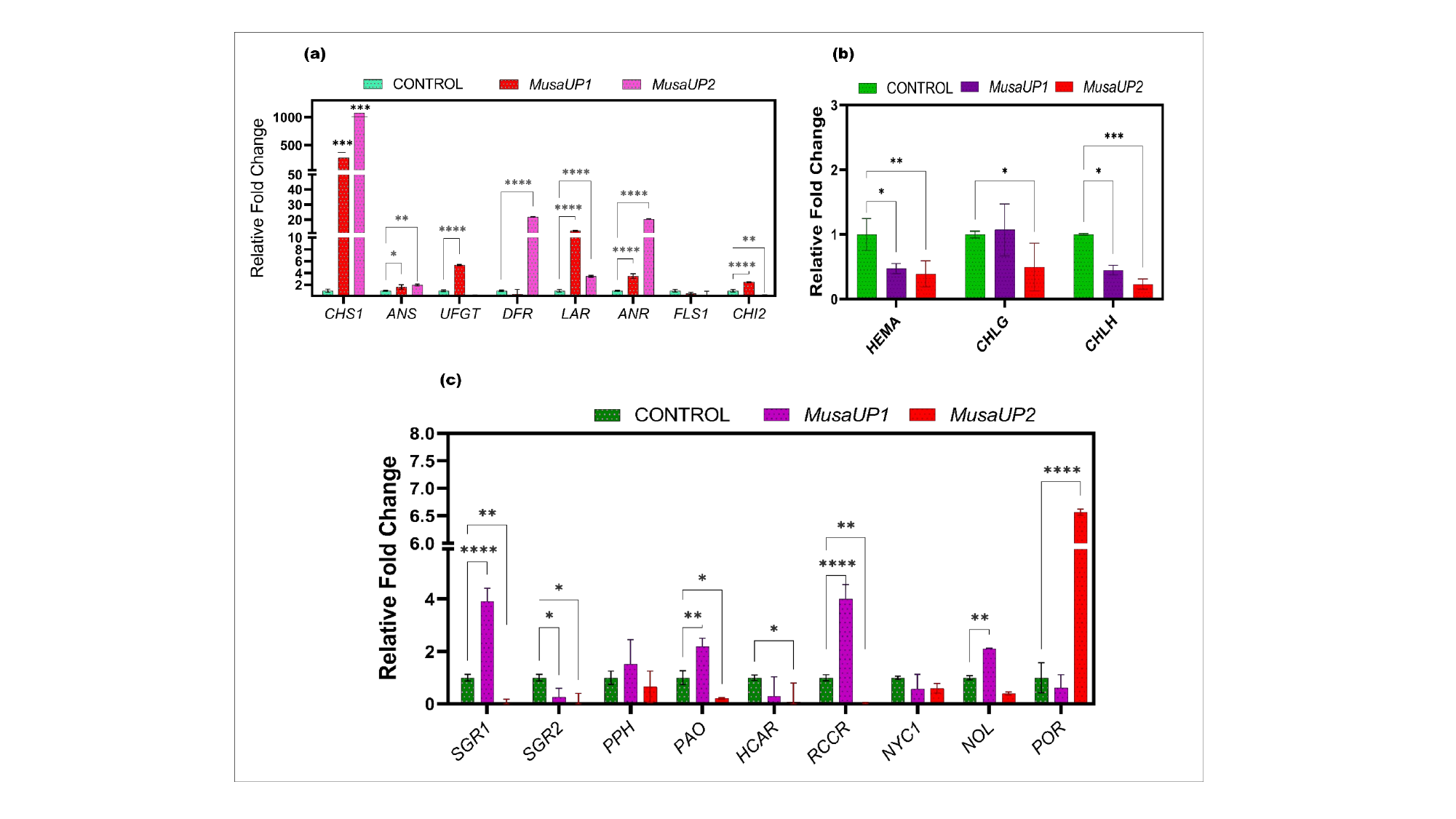

## Slide 15
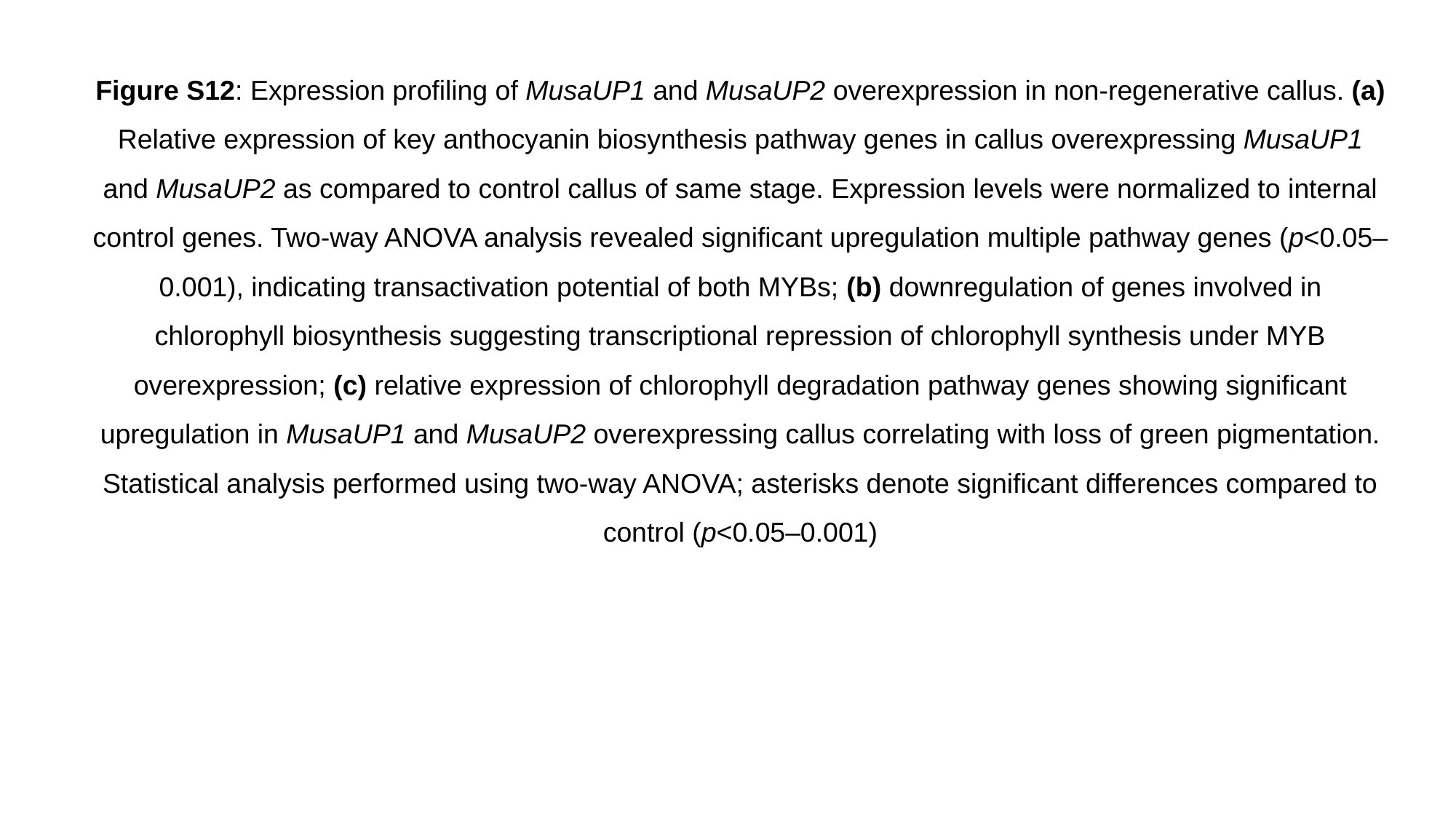

Figure S12: Expression profiling of MusaUP1 and MusaUP2 overexpression in non-regenerative callus. (a) Relative expression of key anthocyanin biosynthesis pathway genes in callus overexpressing MusaUP1 and MusaUP2 as compared to control callus of same stage. Expression levels were normalized to internal control genes. Two-way ANOVA analysis revealed significant upregulation multiple pathway genes (p<0.05–0.001), indicating transactivation potential of both MYBs; (b) downregulation of genes involved in chlorophyll biosynthesis suggesting transcriptional repression of chlorophyll synthesis under MYB overexpression; (c) relative expression of chlorophyll degradation pathway genes showing significant upregulation in MusaUP1 and MusaUP2 overexpressing callus correlating with loss of green pigmentation. Statistical analysis performed using two-way ANOVA; asterisks denote significant differences compared to control (p<0.05–0.001)

## Slide 16
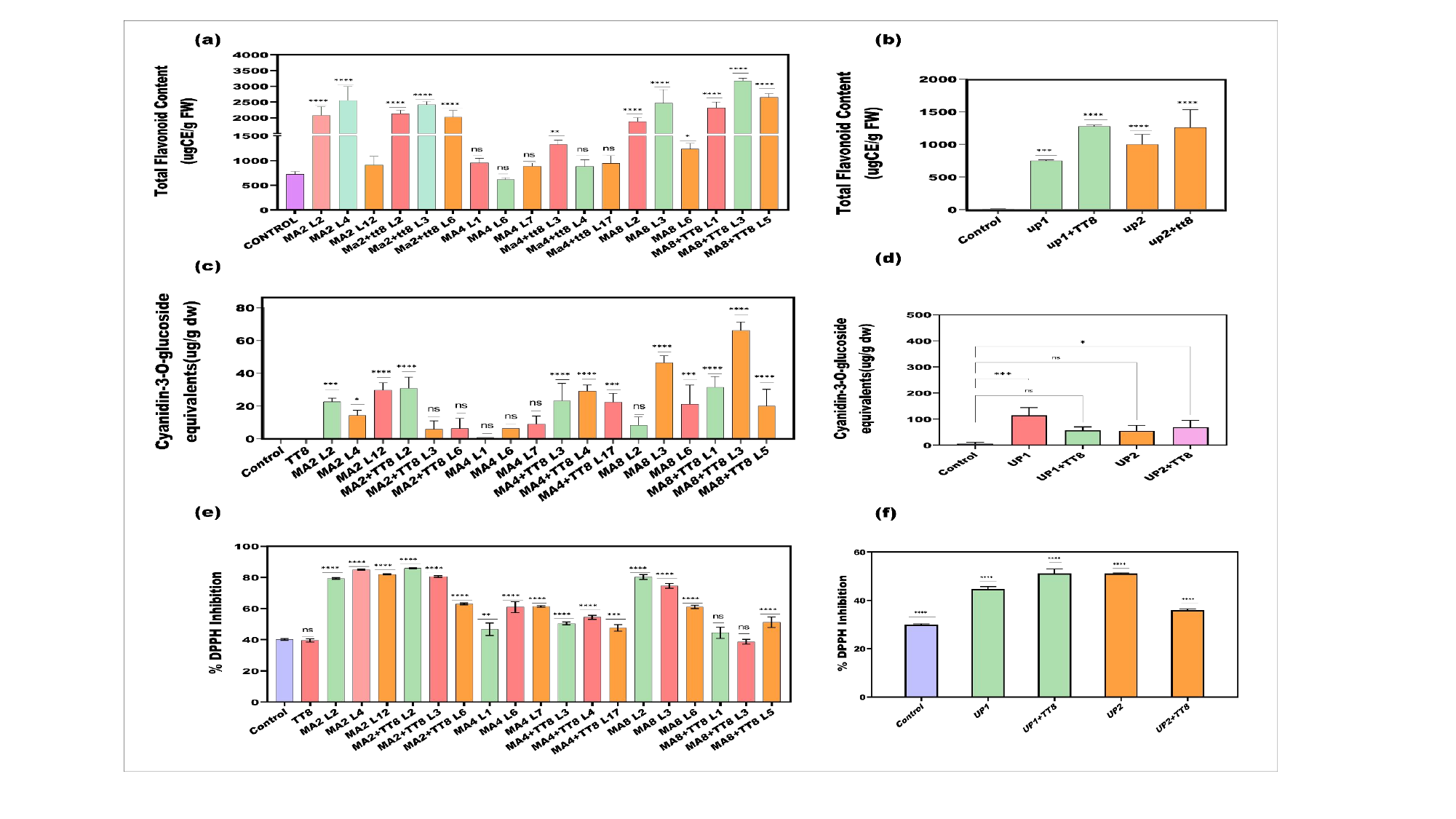

## Slide 17
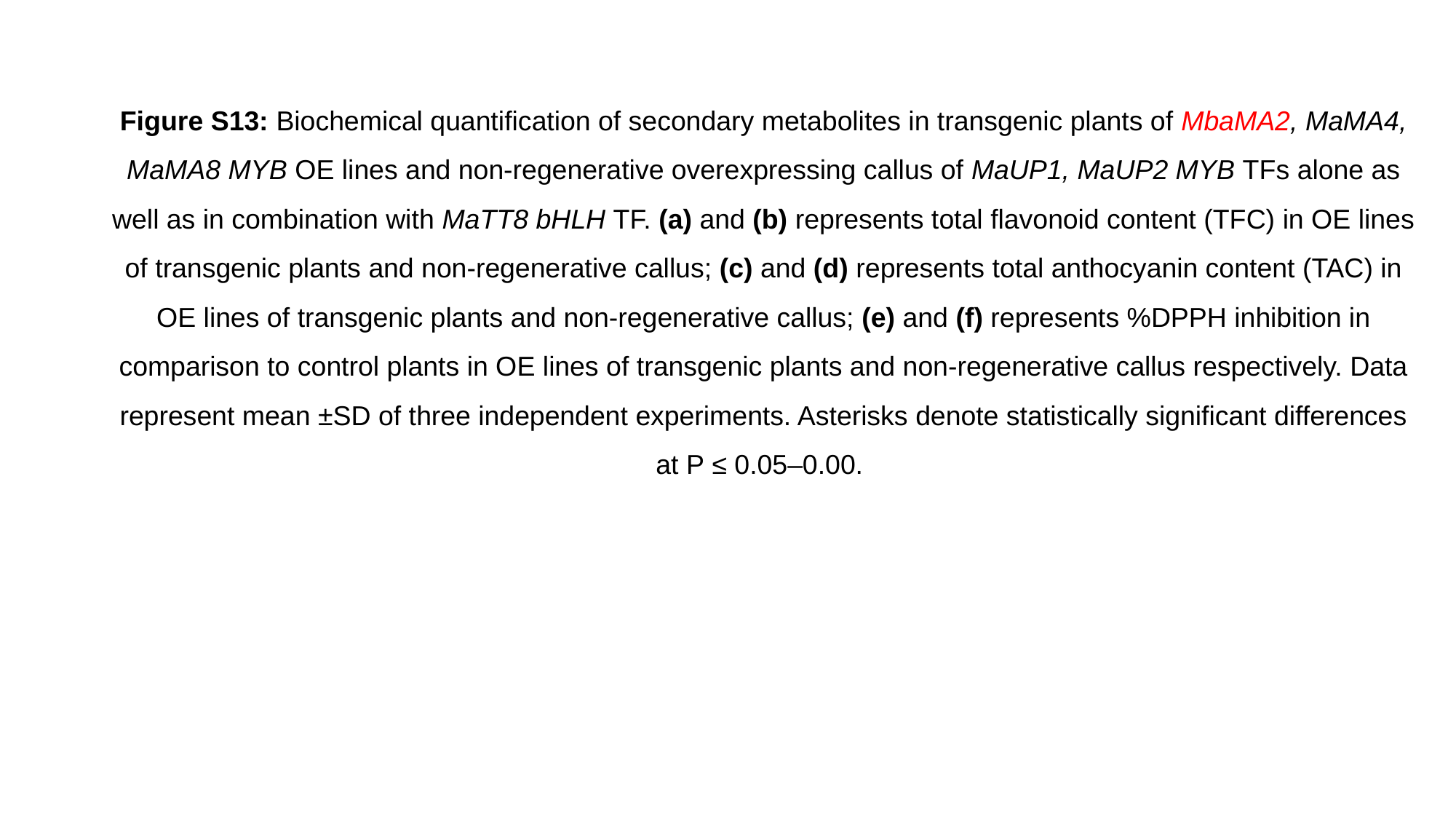

Figure S13: Biochemical quantification of secondary metabolites in transgenic plants of MbaMA2, MaMA4, MaMA8 MYB OE lines and non-regenerative overexpressing callus of MaUP1, MaUP2 MYB TFs alone as well as in combination with MaTT8 bHLH TF. (a) and (b) represents total flavonoid content (TFC) in OE lines of transgenic plants and non-regenerative callus; (c) and (d) represents total anthocyanin content (TAC) in OE lines of transgenic plants and non-regenerative callus; (e) and (f) represents %DPPH inhibition in comparison to control plants in OE lines of transgenic plants and non-regenerative callus respectively. Data represent mean ±SD of three independent experiments. Asterisks denote statistically significant differences at P ≤ 0.05–0.00.

## Slide 18
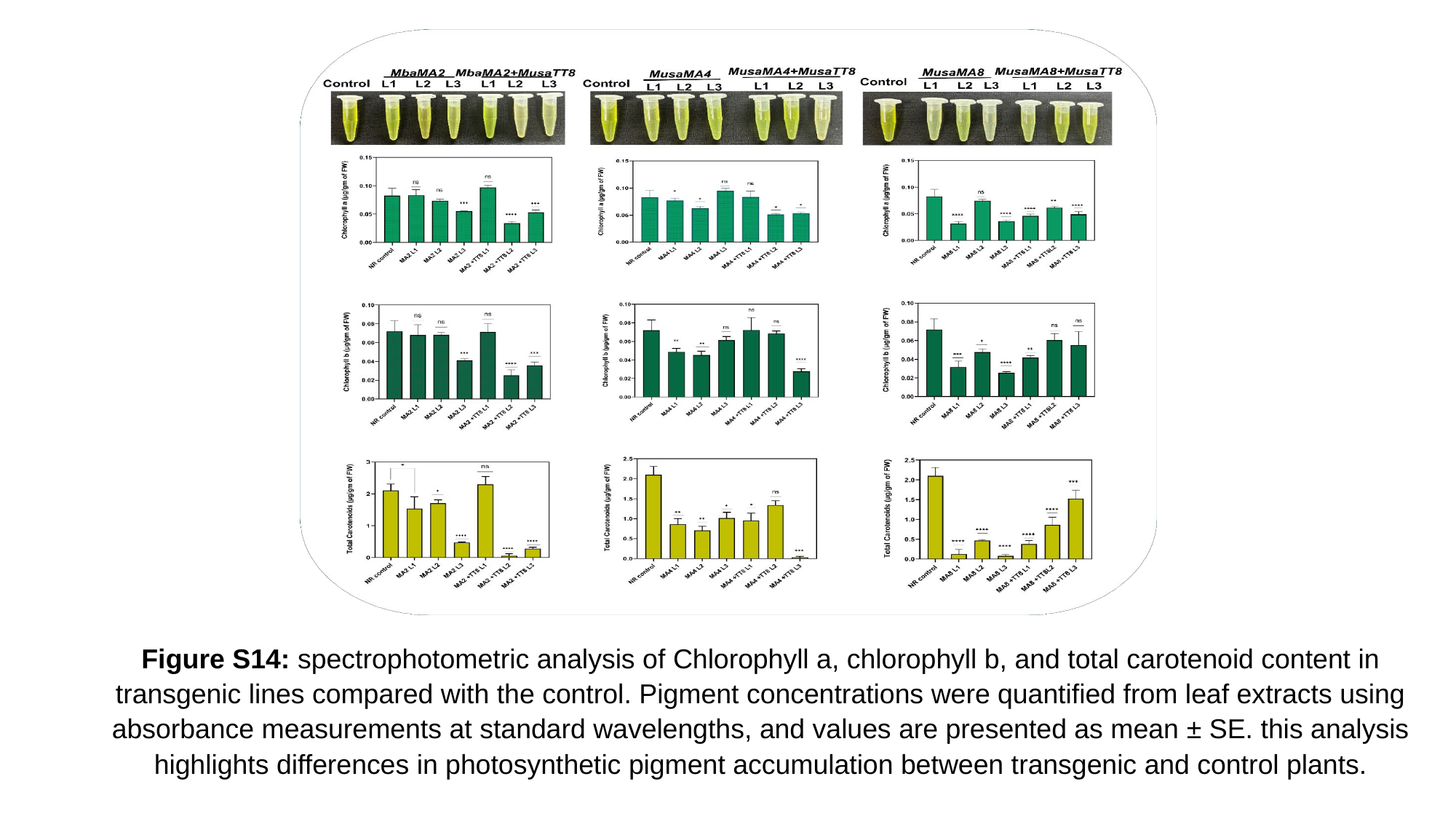

Figure S14: spectrophotometric analysis of Chlorophyll a, chlorophyll b, and total carotenoid content in transgenic lines compared with the control. Pigment concentrations were quantified from leaf extracts using absorbance measurements at standard wavelengths, and values are presented as mean ± SE. this analysis highlights differences in photosynthetic pigment accumulation between transgenic and control plants.

## Slide 19
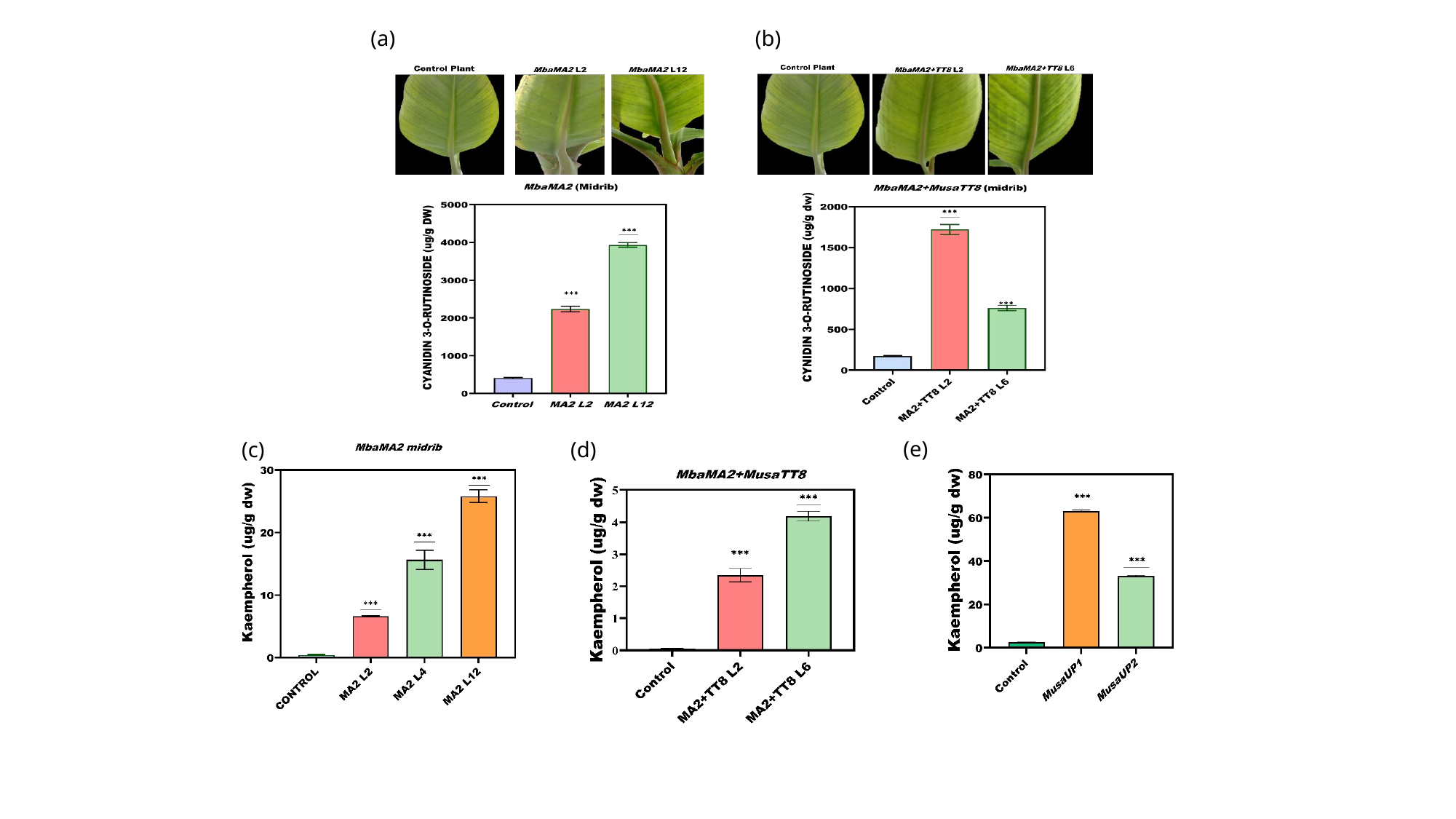

(a)
(b)
(e)
(c)
(d)

## Slide 20
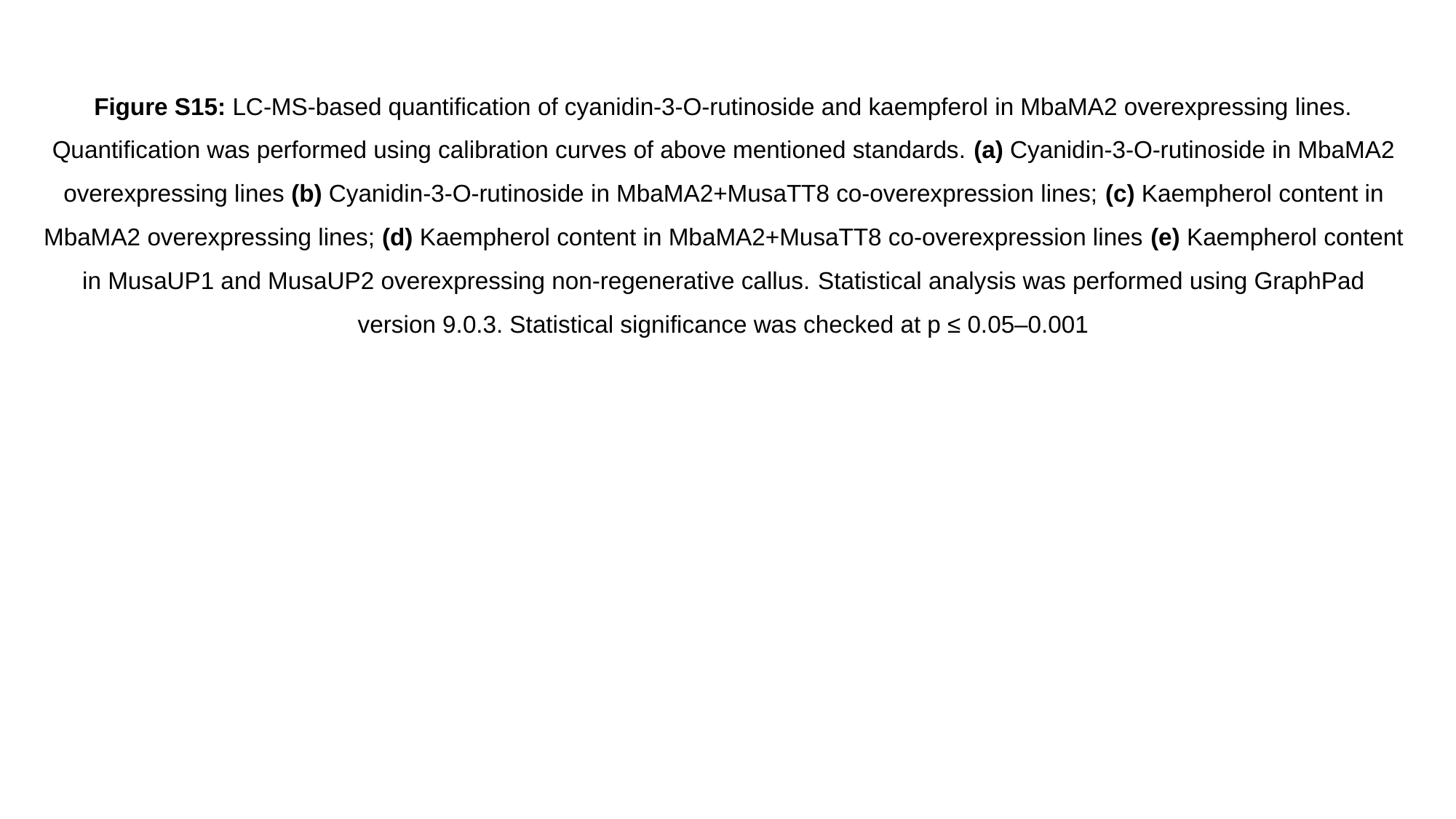

Figure S15: LC-MS-based quantification of cyanidin-3-O-rutinoside and kaempferol in MbaMA2 overexpressing lines. Quantification was performed using calibration curves of above mentioned standards. (a) Cyanidin-3-O-rutinoside in MbaMA2 overexpressing lines (b) Cyanidin-3-O-rutinoside in MbaMA2+MusaTT8 co-overexpression lines; (c) Kaempherol content in MbaMA2 overexpressing lines; (d) Kaempherol content in MbaMA2+MusaTT8 co-overexpression lines (e) Kaempherol content in MusaUP1 and MusaUP2 overexpressing non-regenerative callus. Statistical analysis was performed using GraphPad version 9.0.3. Statistical significance was checked at p ≤ 0.05–0.001

## Slide 21
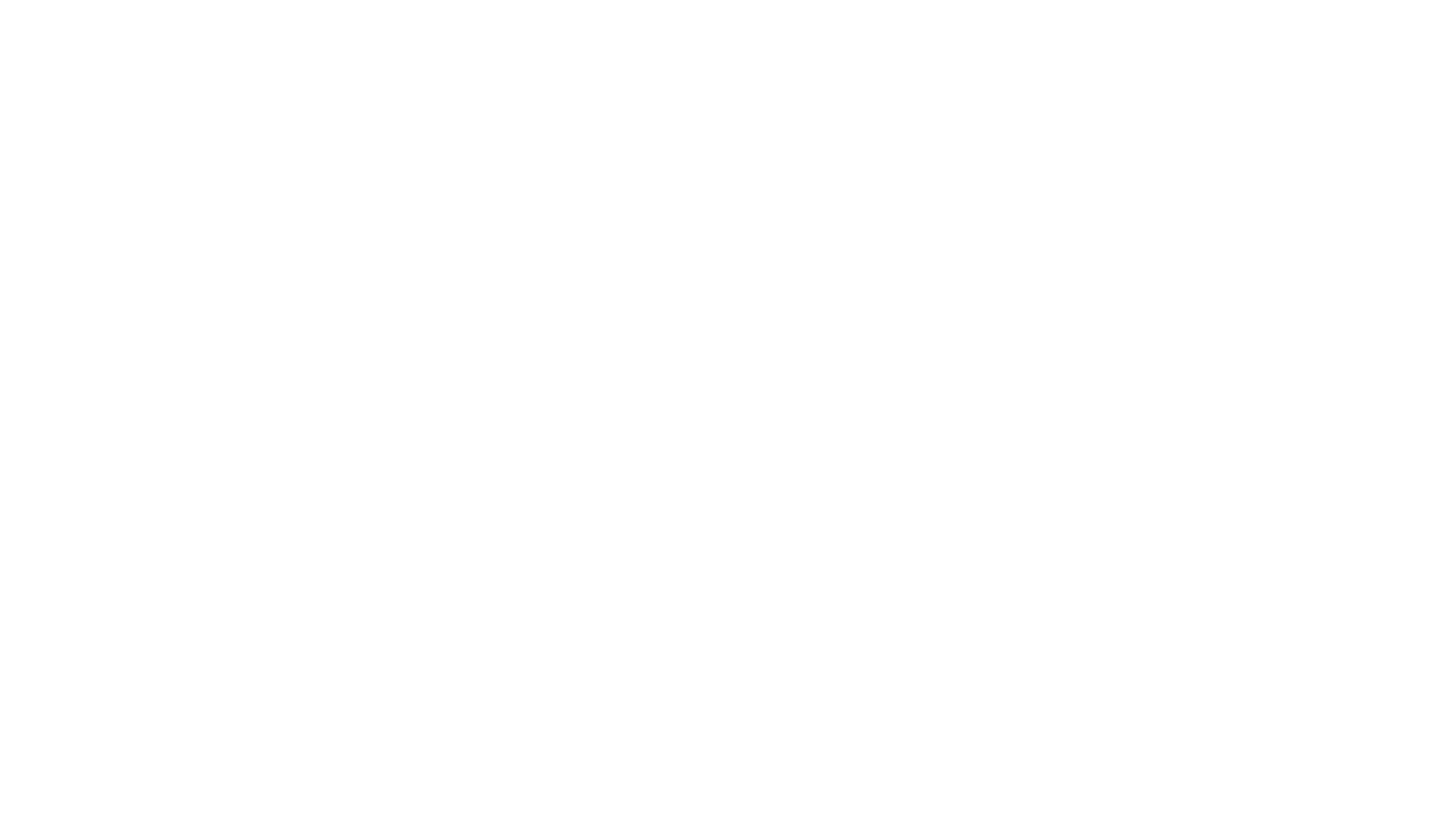

Supplement: Web_Material_uhaf361 [file Web_Material_uhaf361.zip › Supplementary figures S5.pptx]
