# Supplementary material for: A coordinated network of MYB regulators orchestrates anthocyanin biosynthesis in banana
Source: Hortic Res. 2026 Jan 13;13(6):uhaf361. doi: 10.1093/hr/uhaf361 (PMC13273576; doi:10.1093/hr/uhaf361)
Supplement: Web_Material_uhaf361 [file Web_Material_uhaf361.zip › Supplementary file S2.pptx]

## Slide 1
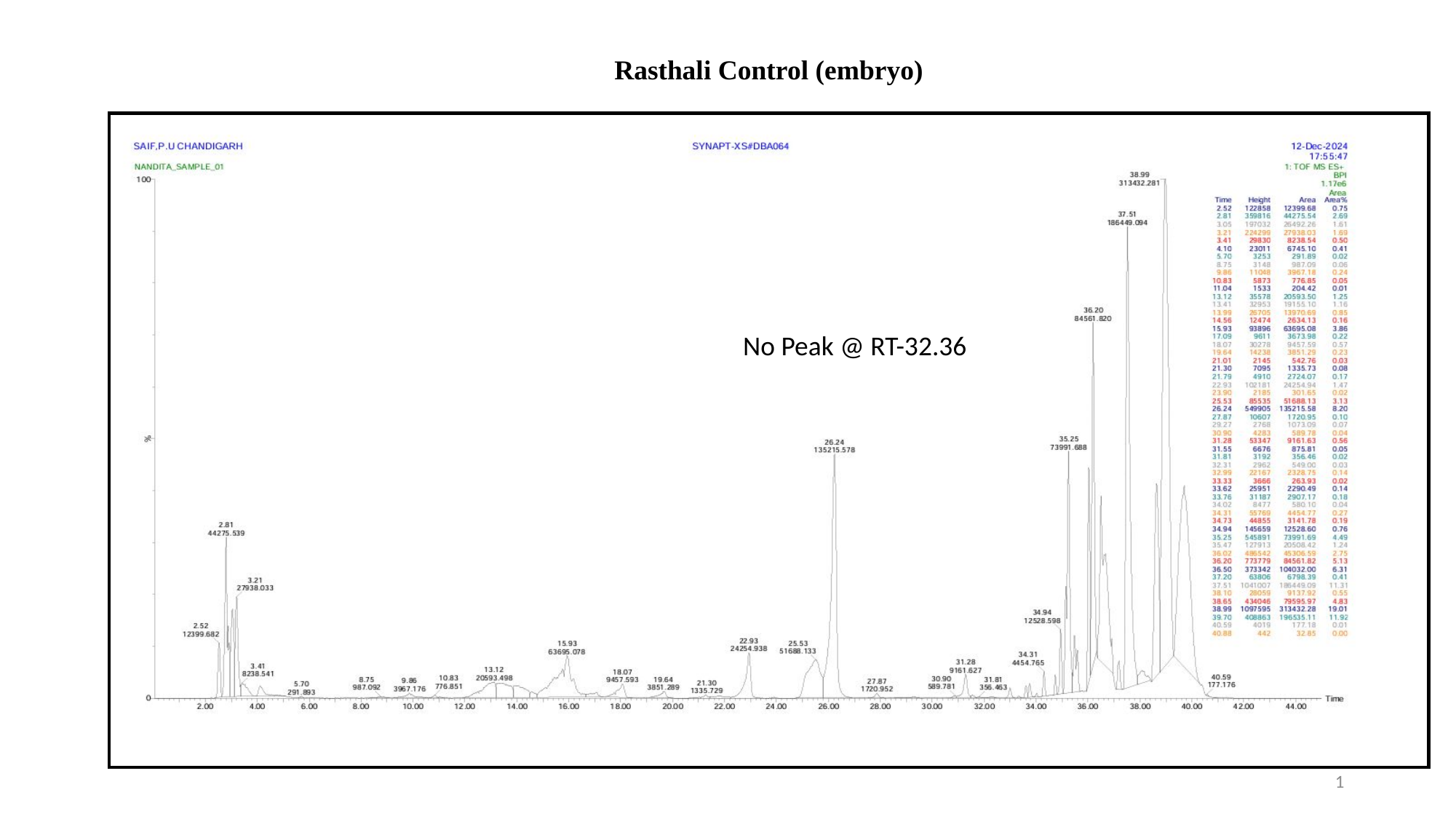

Rasthali Control (embryo)
No Peak @ RT-32.36
1

## Slide 2
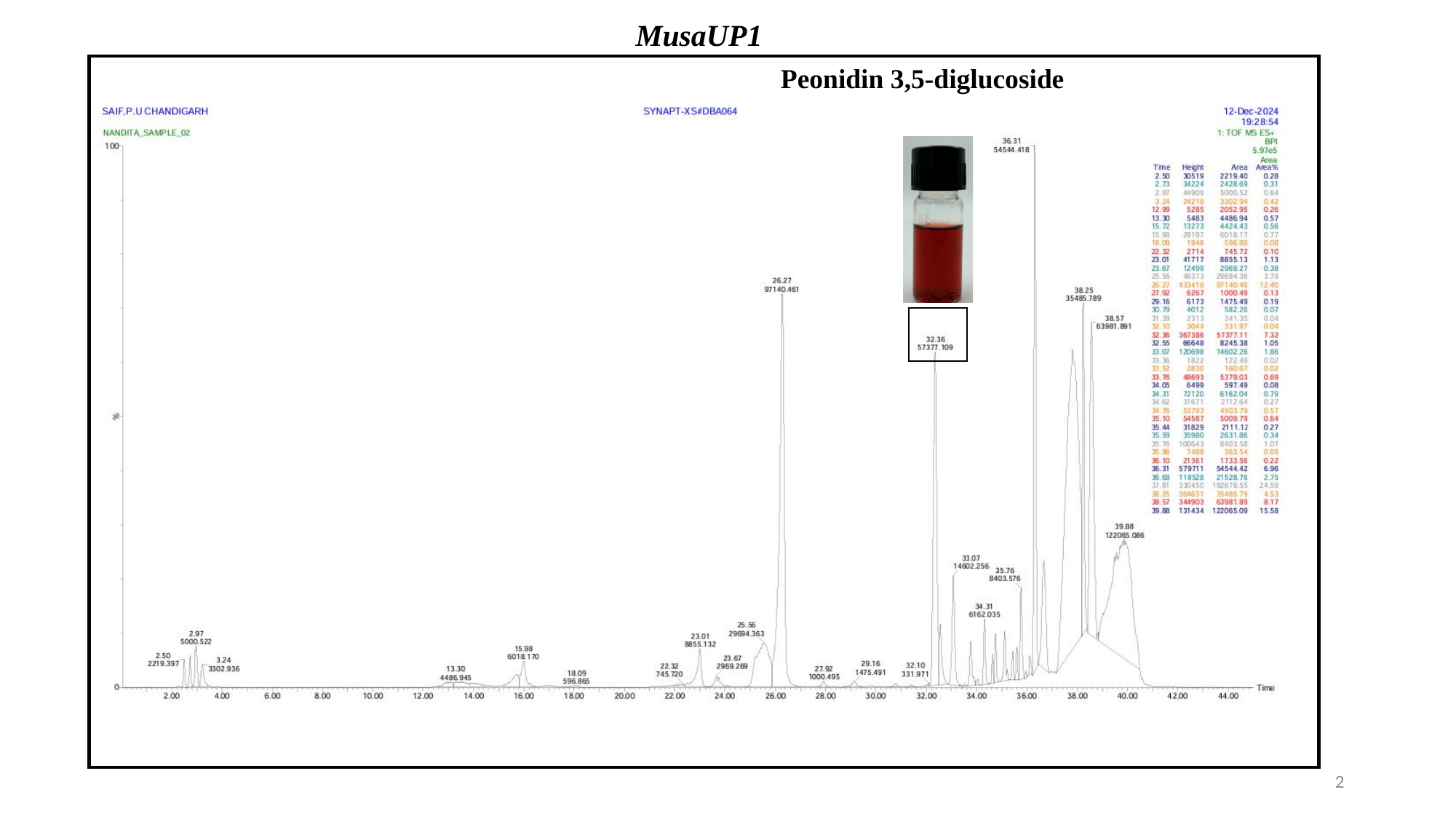

MusaUP1
Peonidin 3,5-diglucoside
2

## Slide 3
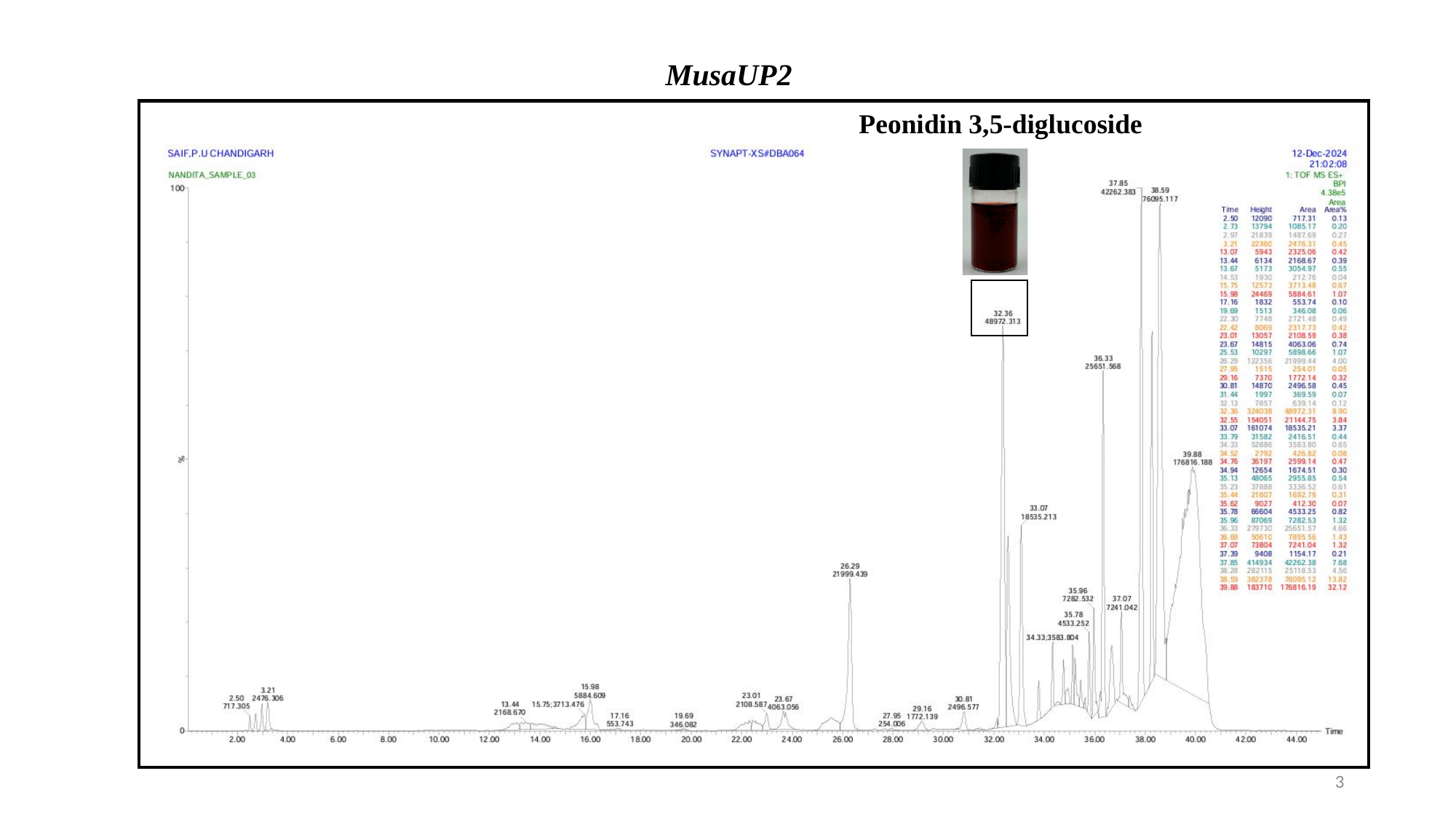

MusaUP2
Peonidin 3,5-diglucoside
3

## Slide 4
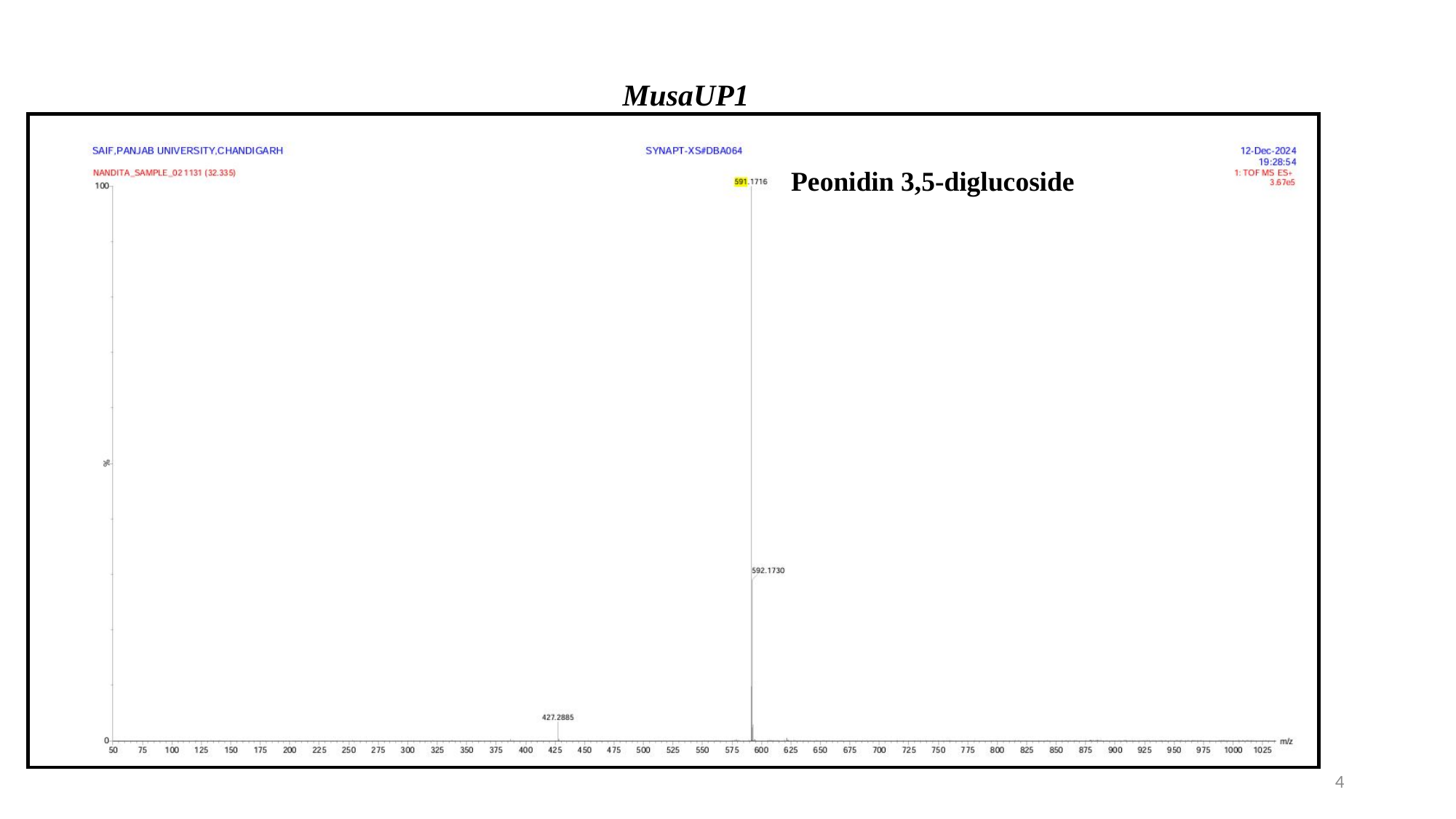

MusaUP1
Peonidin 3,5-diglucoside
4

## Slide 5
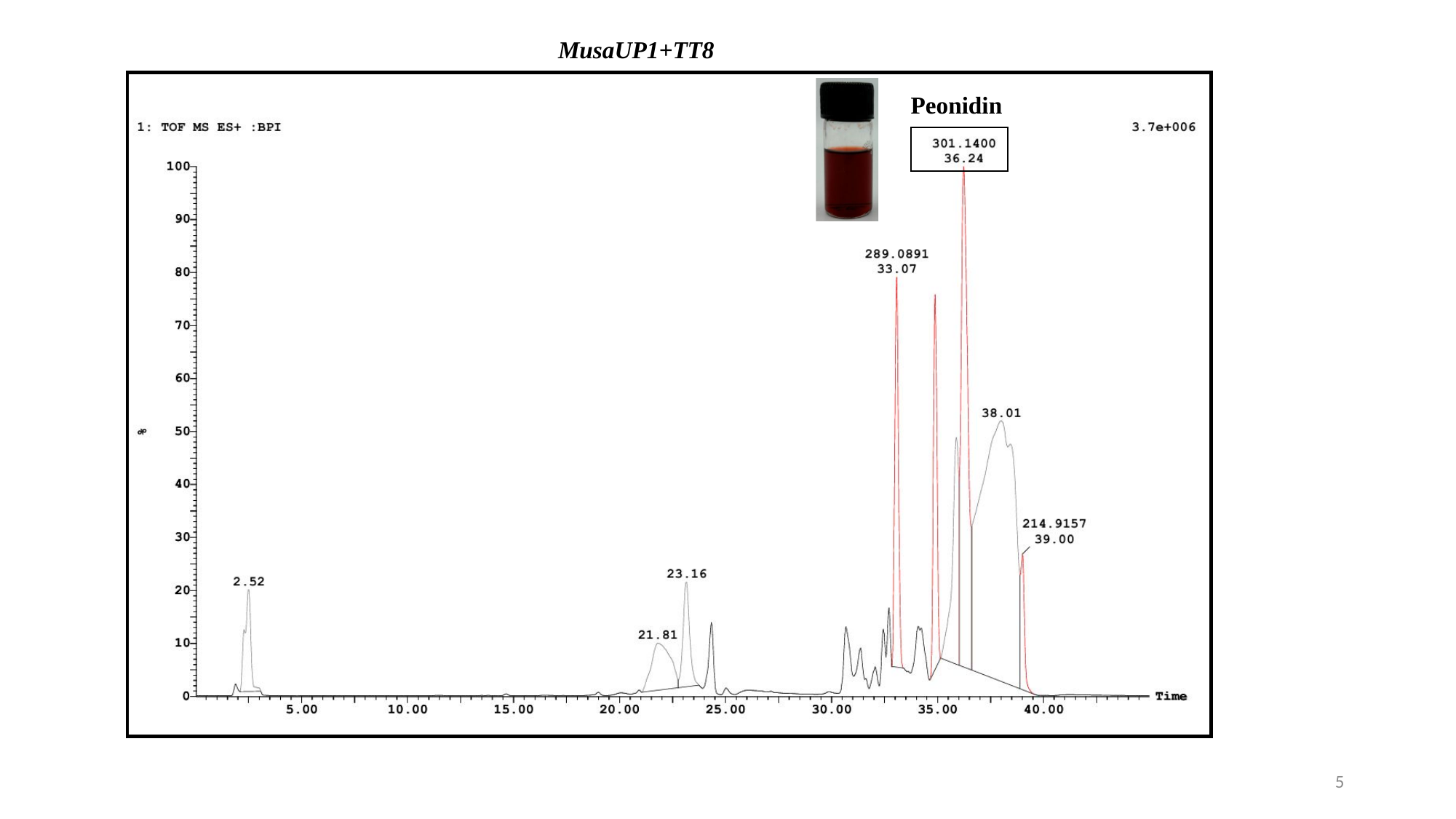

MusaUP1+TT8
Peonidin
5

## Slide 6
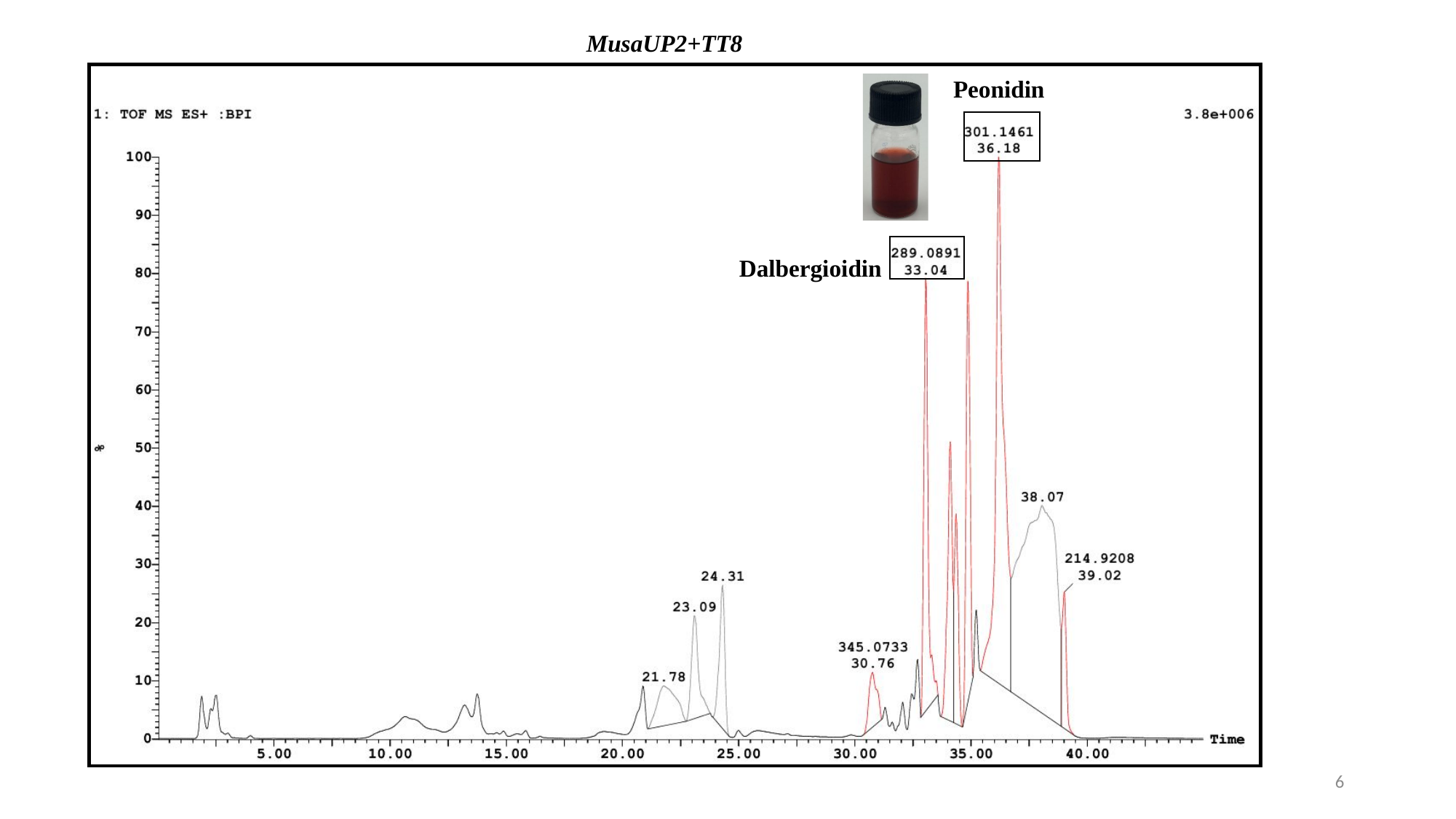

MusaUP2+TT8
Peonidin
Dalbergioidin
6

## Slide 7
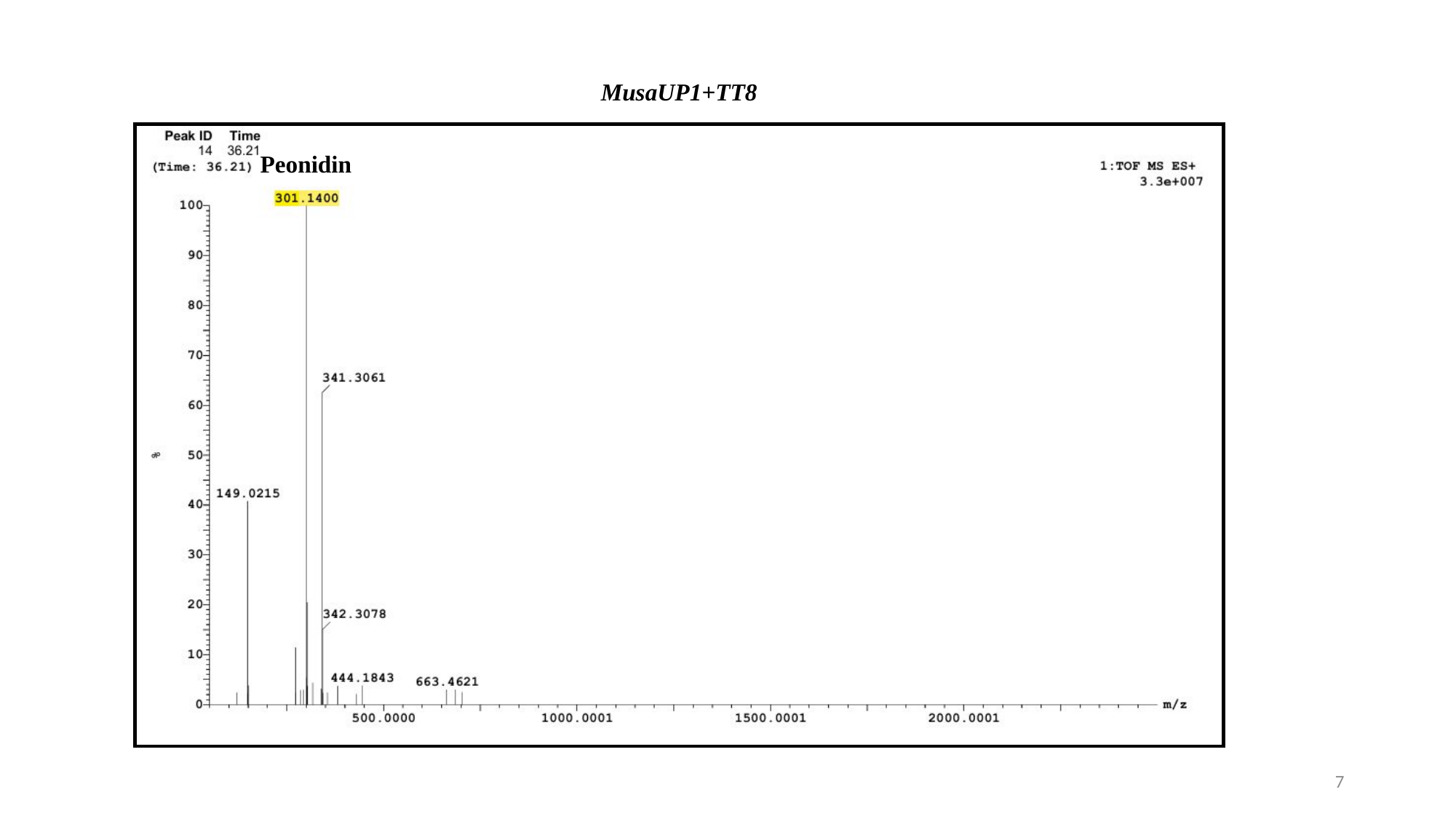

MusaUP1+TT8
Peonidin
7

## Slide 8
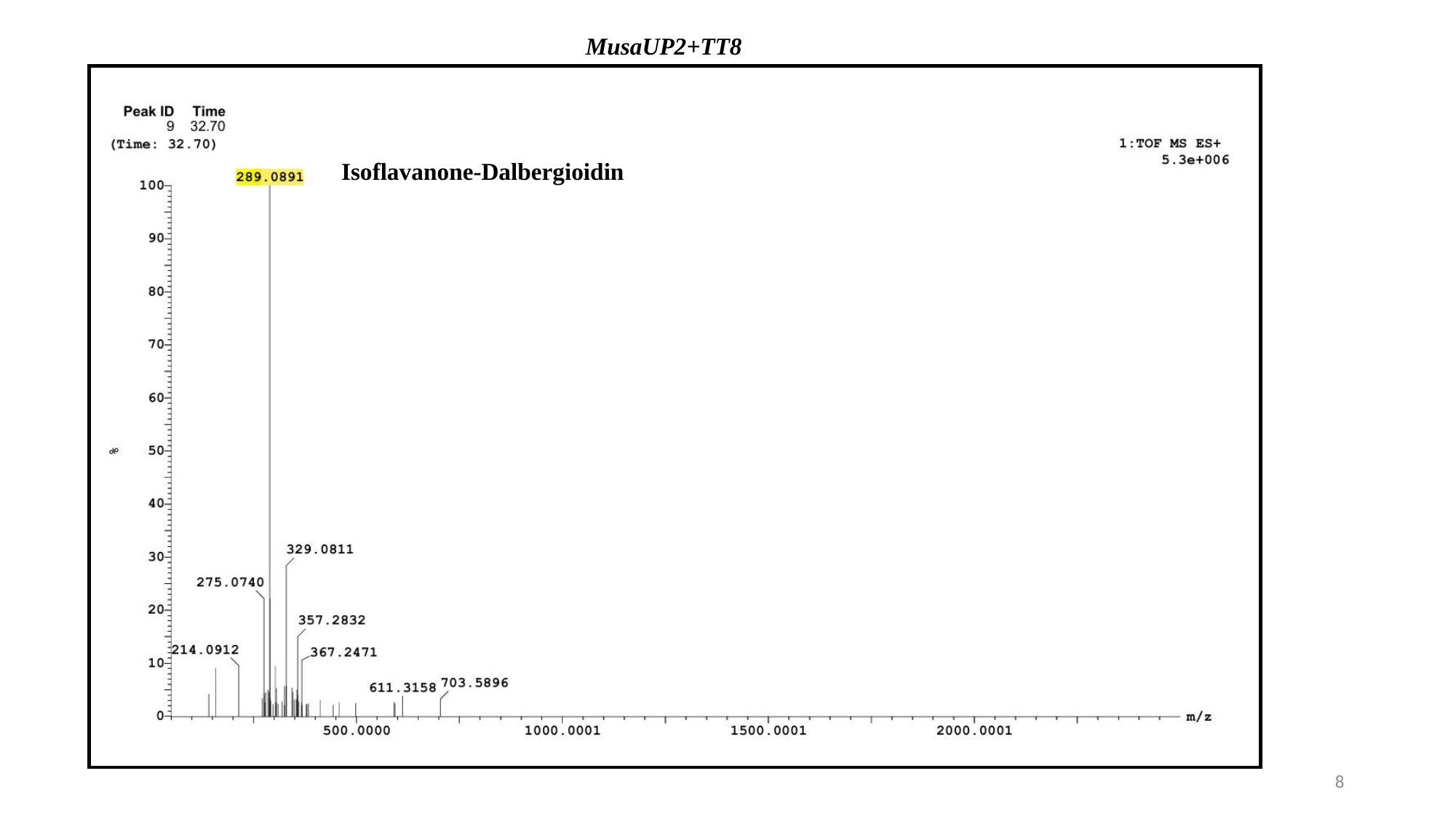

MusaUP2+TT8
Isoflavanone-Dalbergioidin
8

## Slide 9
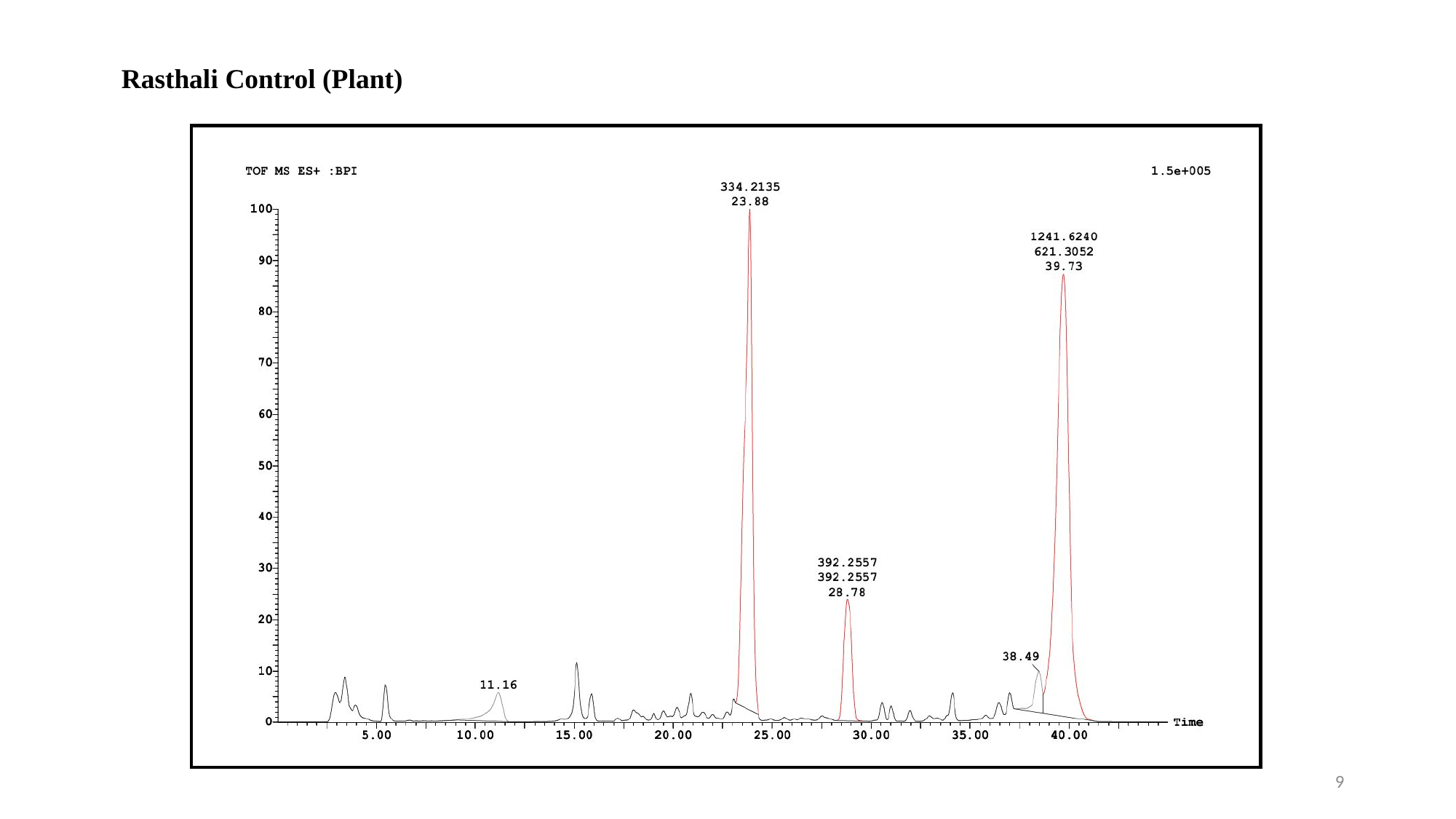

Rasthali Control (Plant)
9

## Slide 10
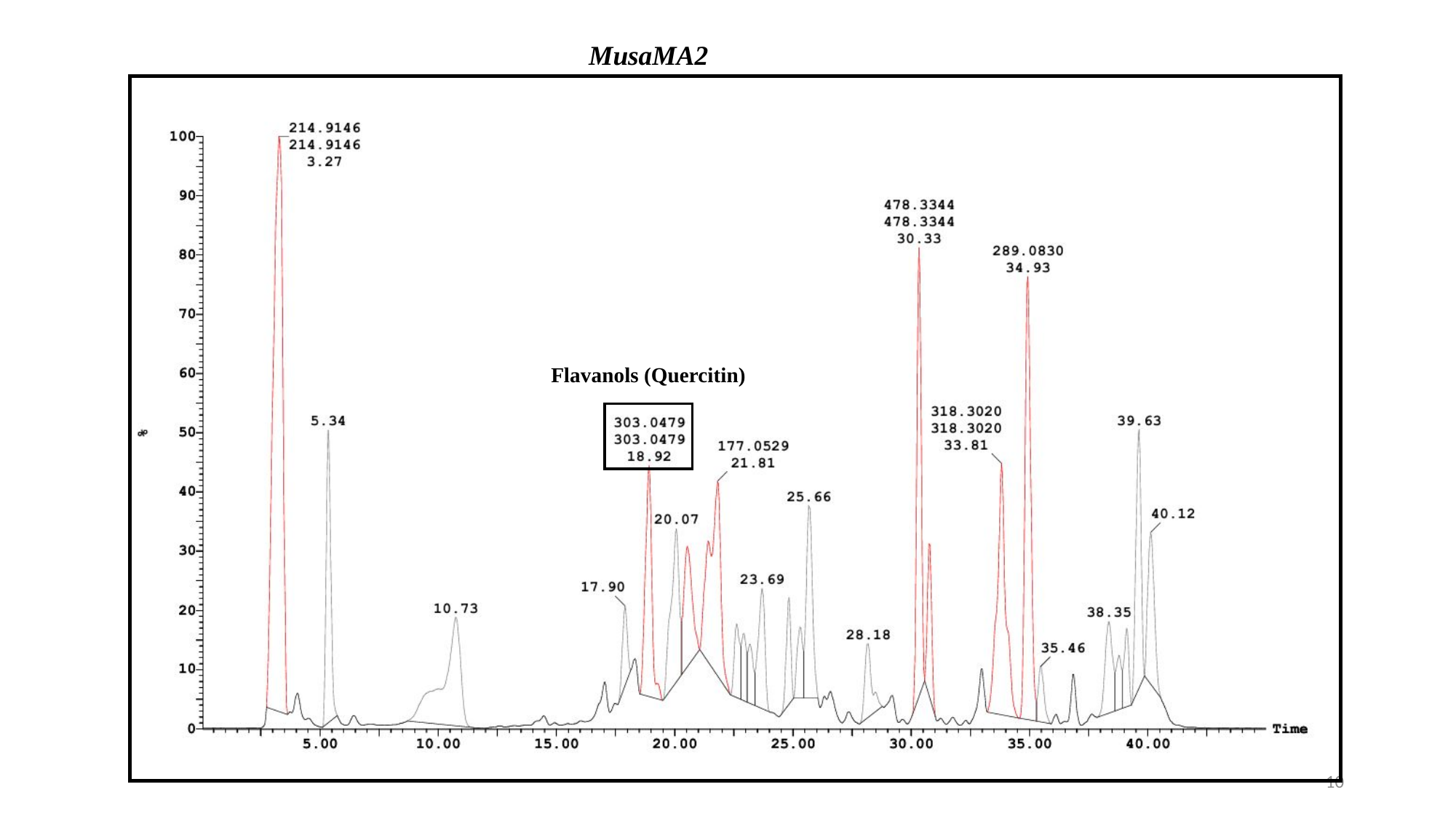

MusaMA2
Flavanols (Quercitin)
10

## Slide 11
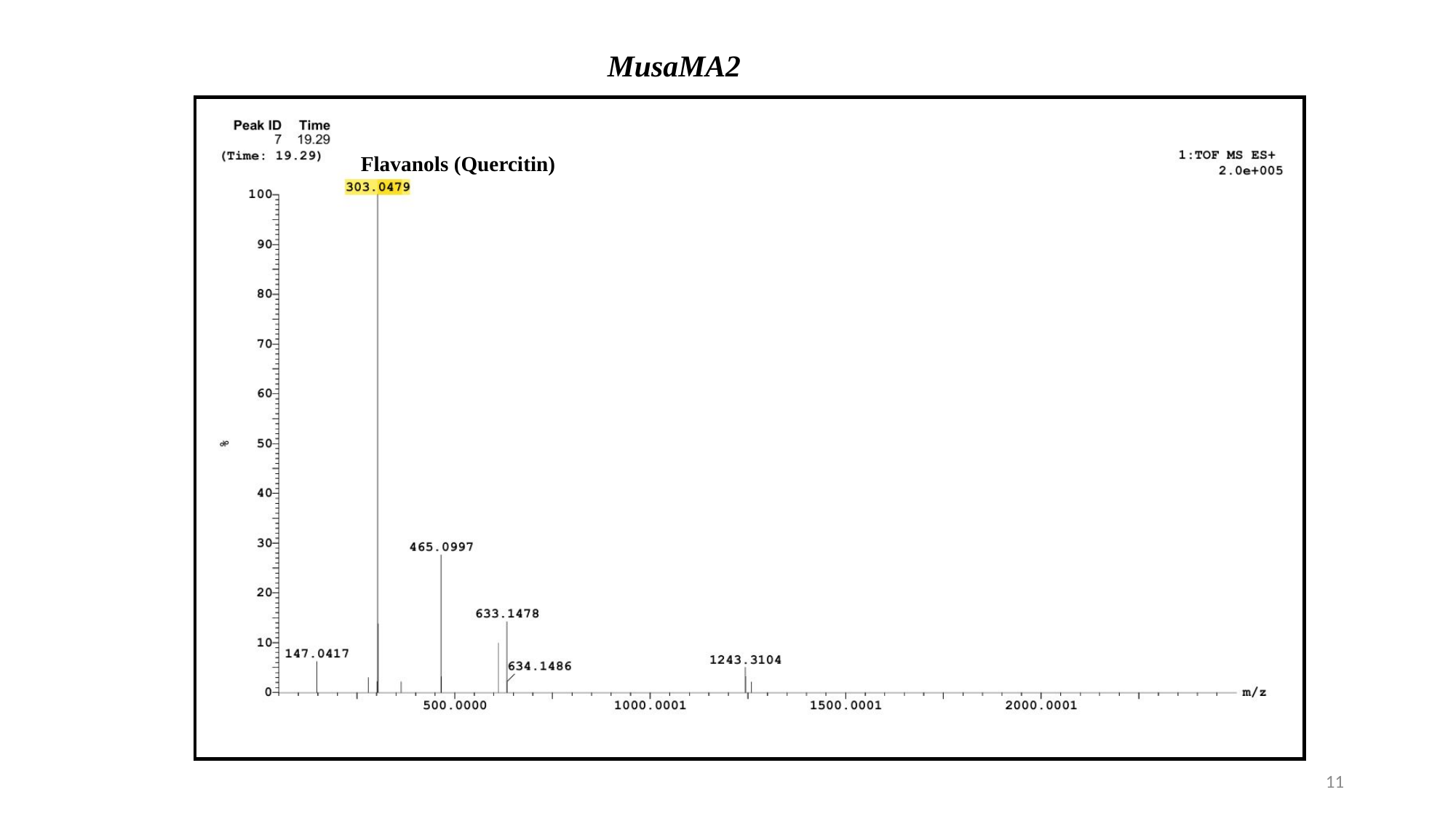

MusaMA2
Flavanols (Quercitin)
11

## Slide 12
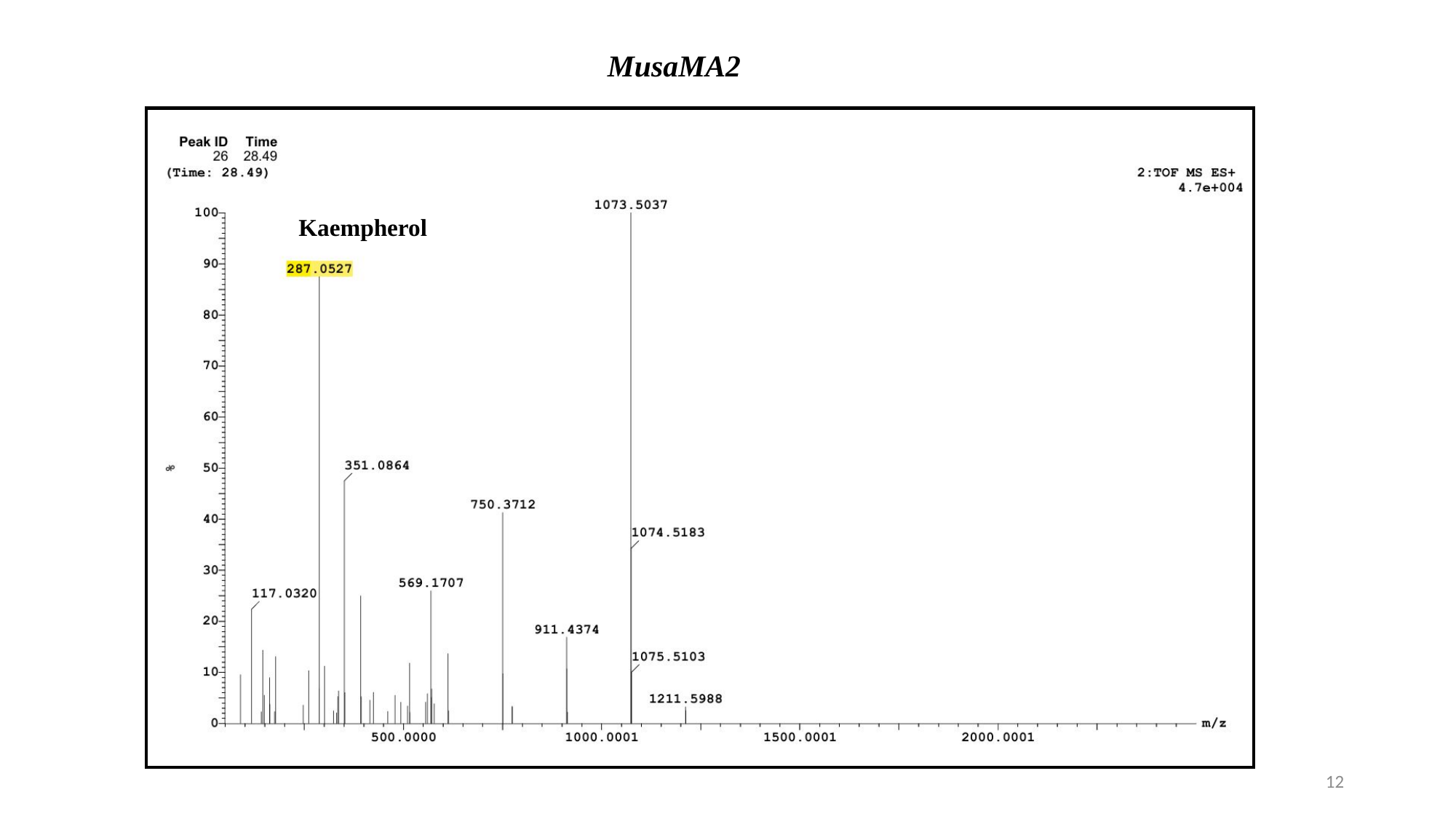

MusaMA2
Kaempherol
12

## Slide 13
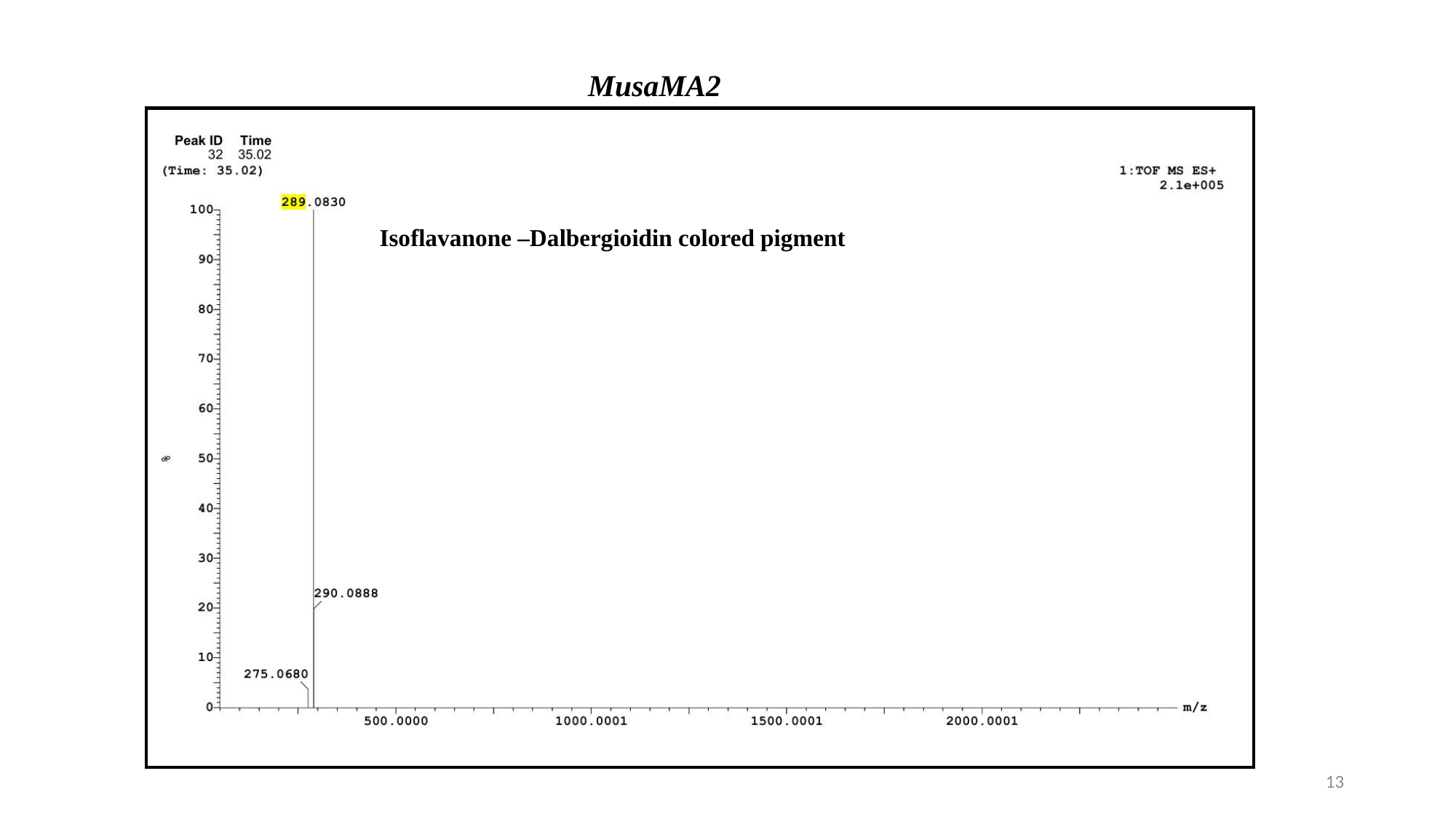

MusaMA2
Isoflavanone –Dalbergioidin colored pigment
13

## Slide 14
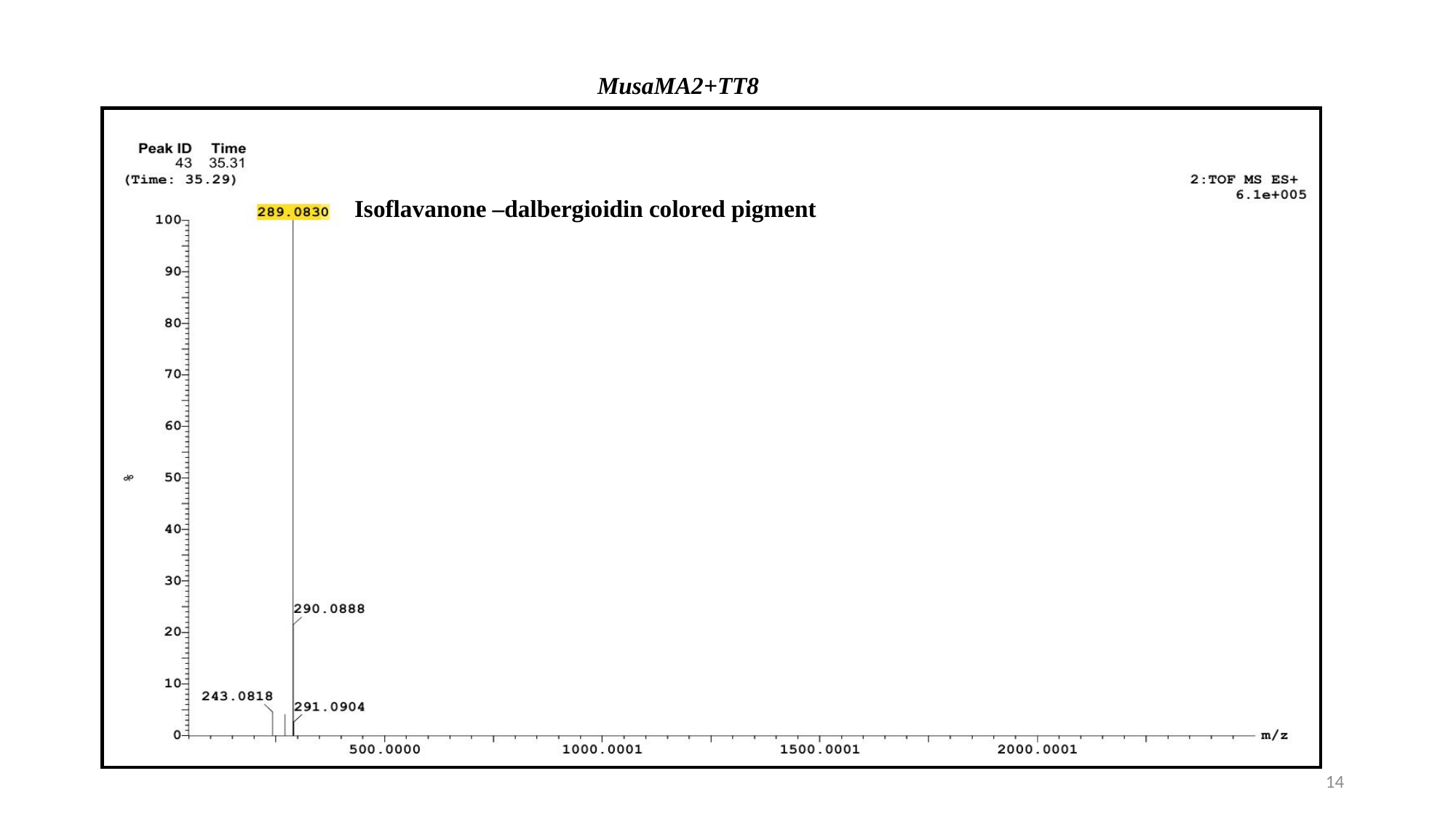

MusaMA2+TT8
Isoflavanone –dalbergioidin colored pigment
14

## Slide 15
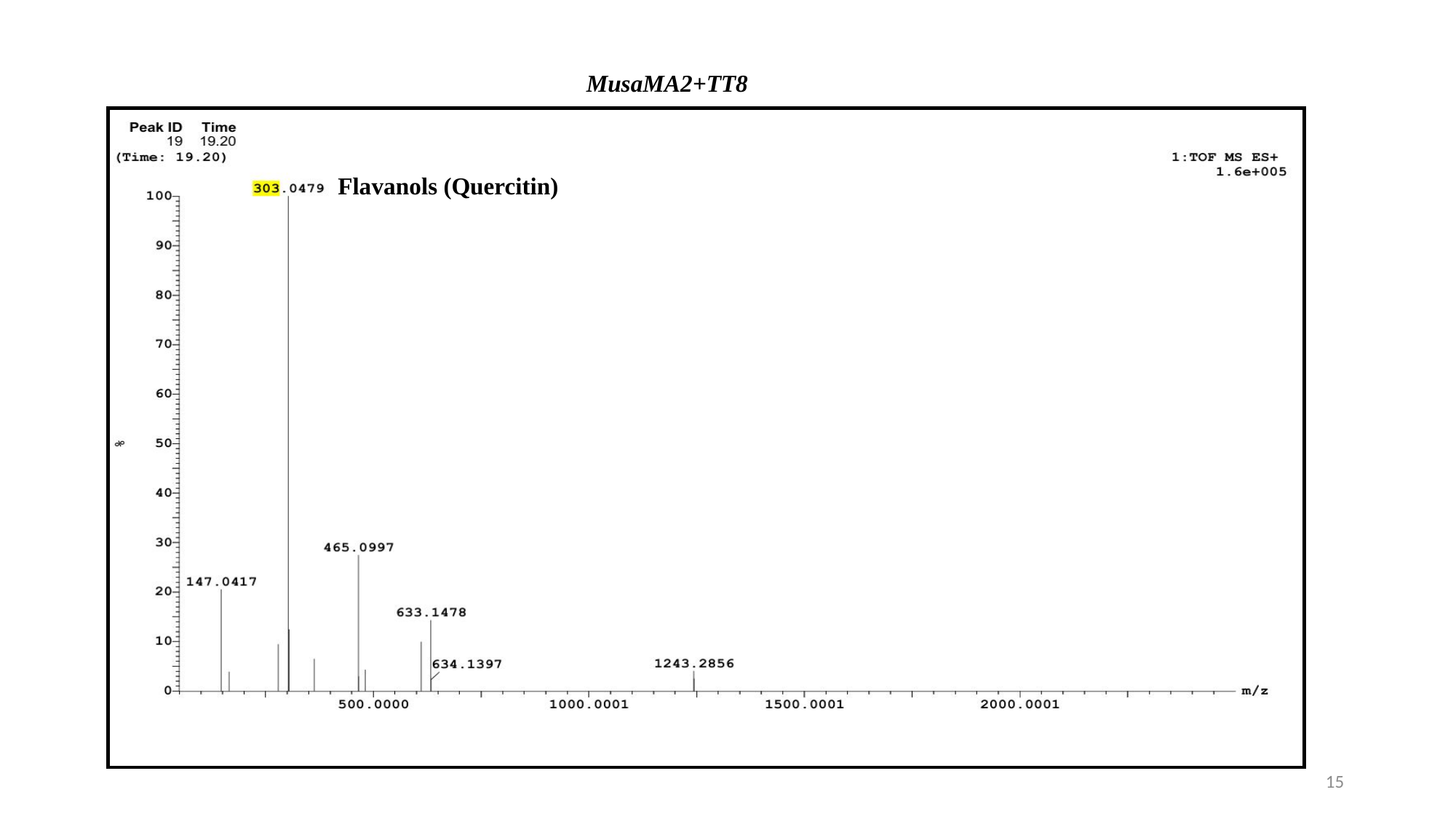

MusaMA2+TT8
Flavanols (Quercitin)
15

## Slide 16
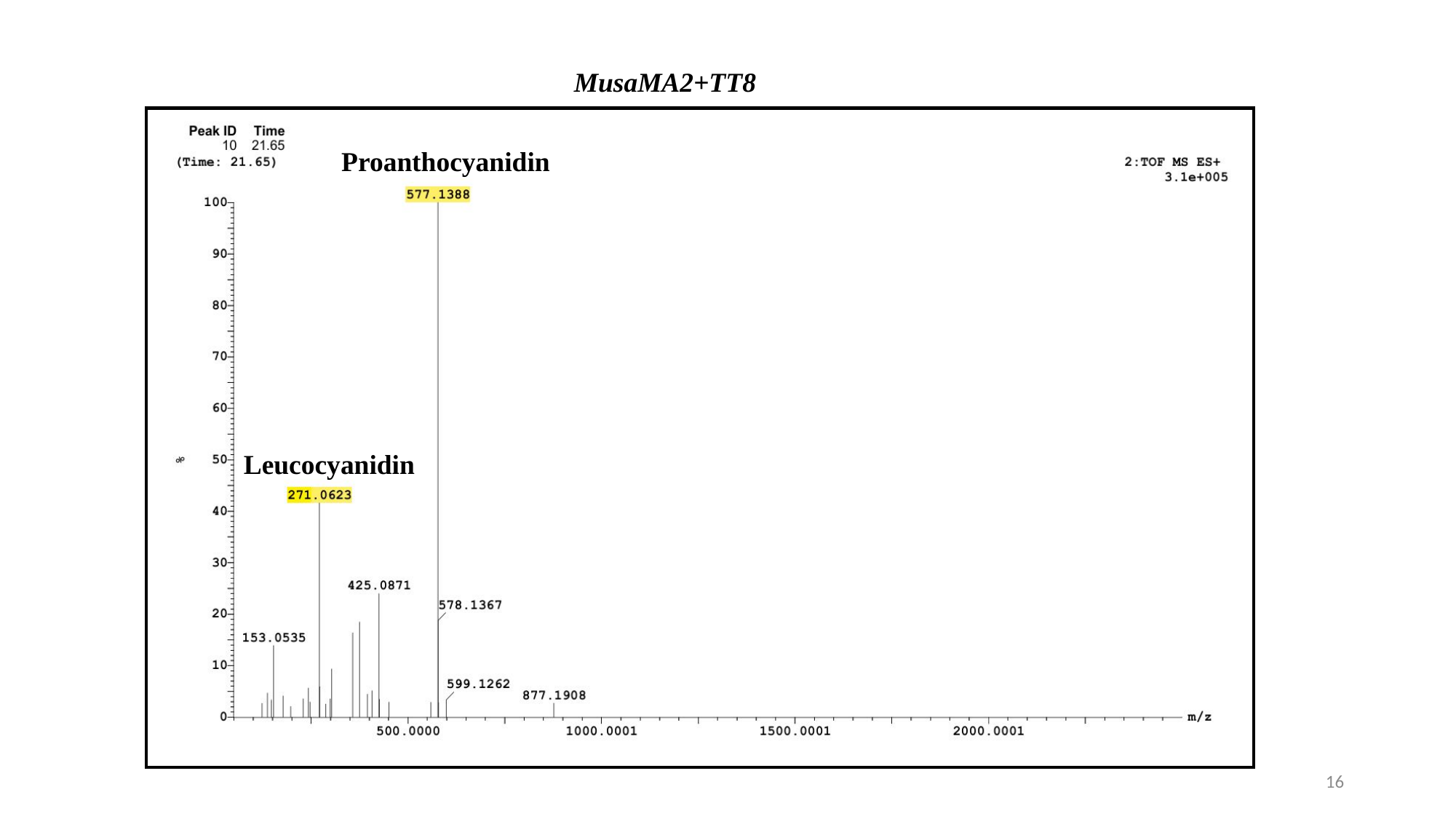

MusaMA2+TT8
Proanthocyanidin
Leucocyanidin
16

## Slide 17
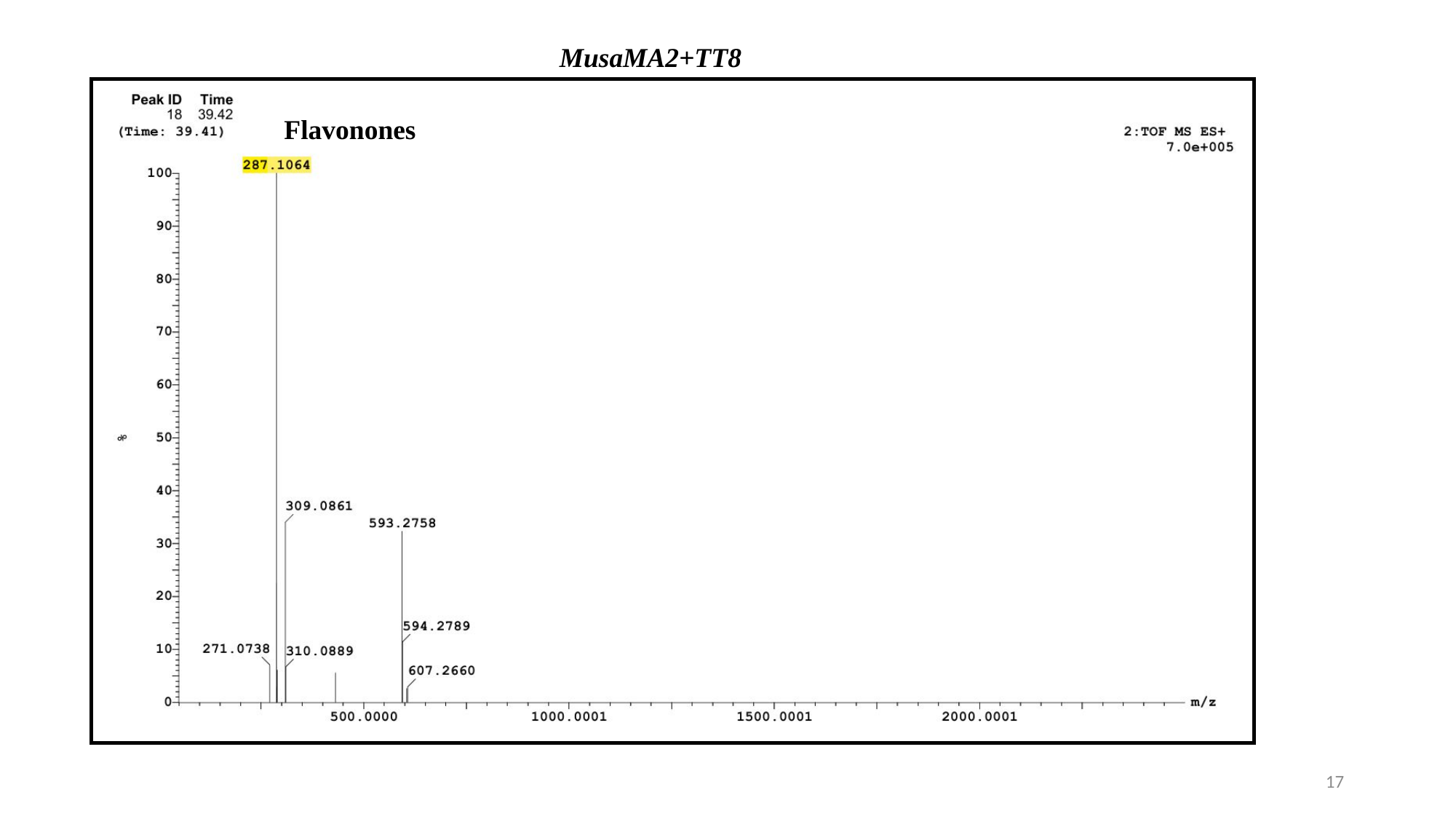

MusaMA2+TT8
Flavonones
17

## Slide 18
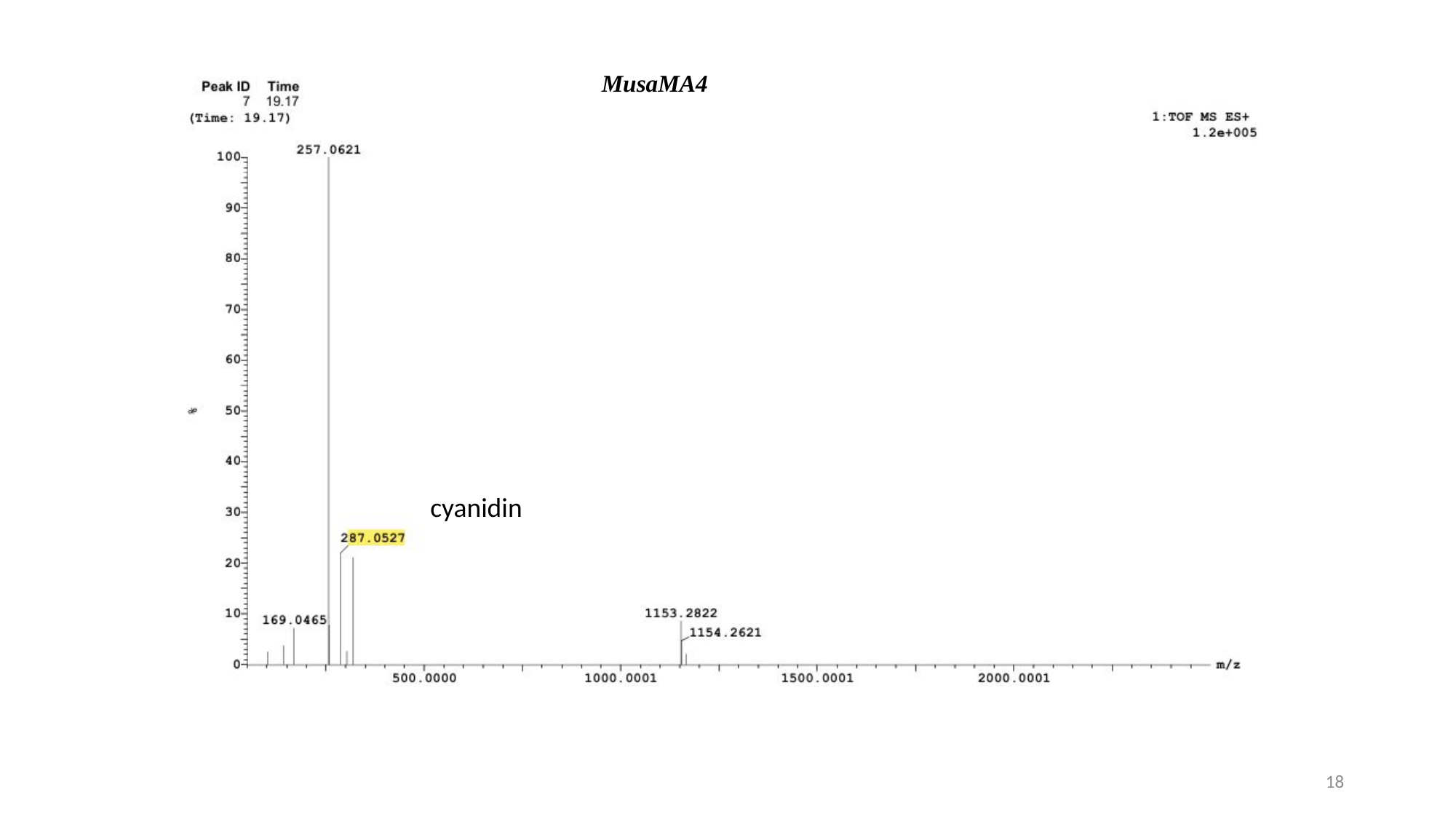

MusaMA4
cyanidin
18

## Slide 19
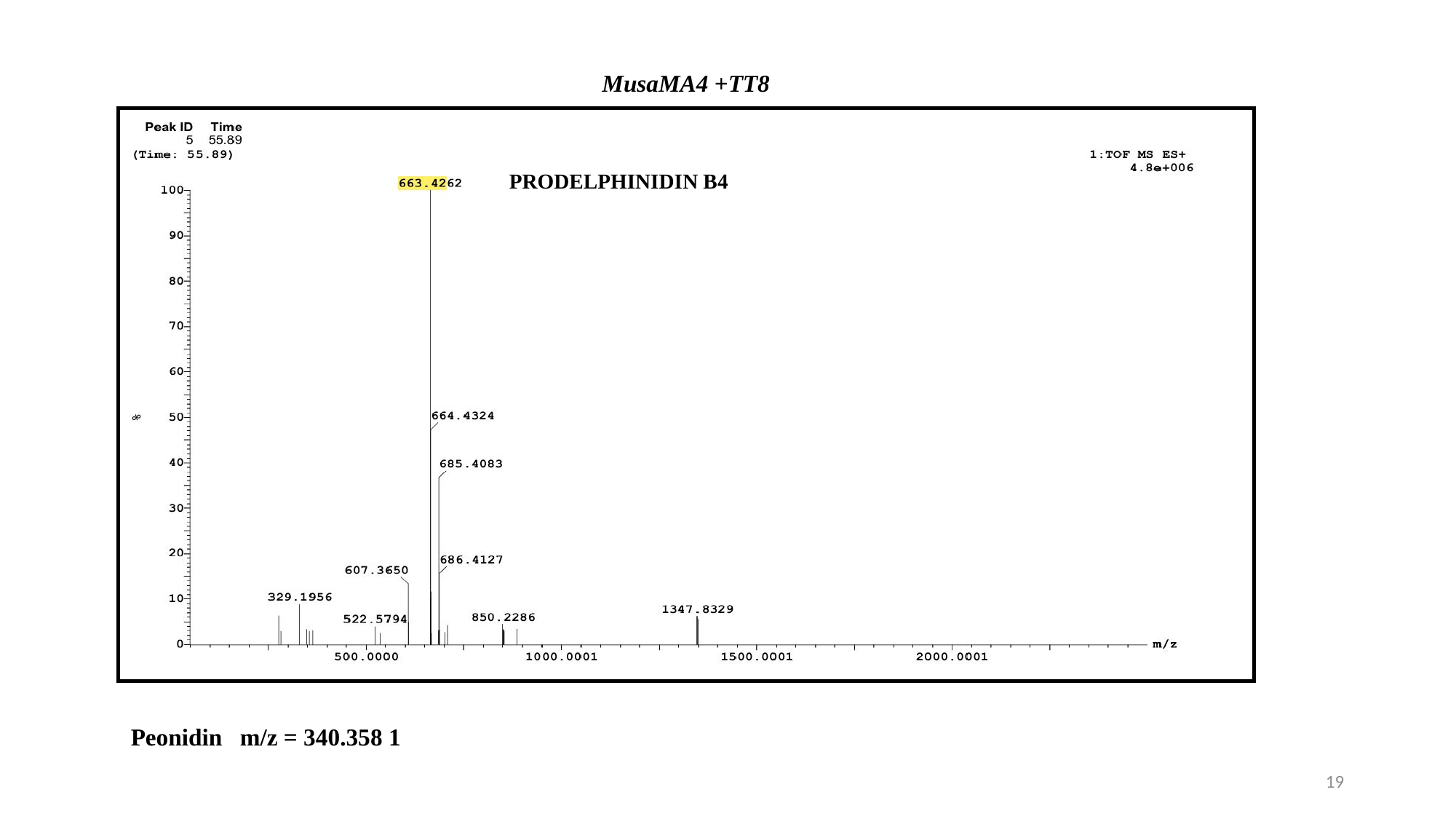

MusaMA4 +TT8
PRODELPHINIDIN B4
Peonidin   m/z = 340.358 1
19

## Slide 20
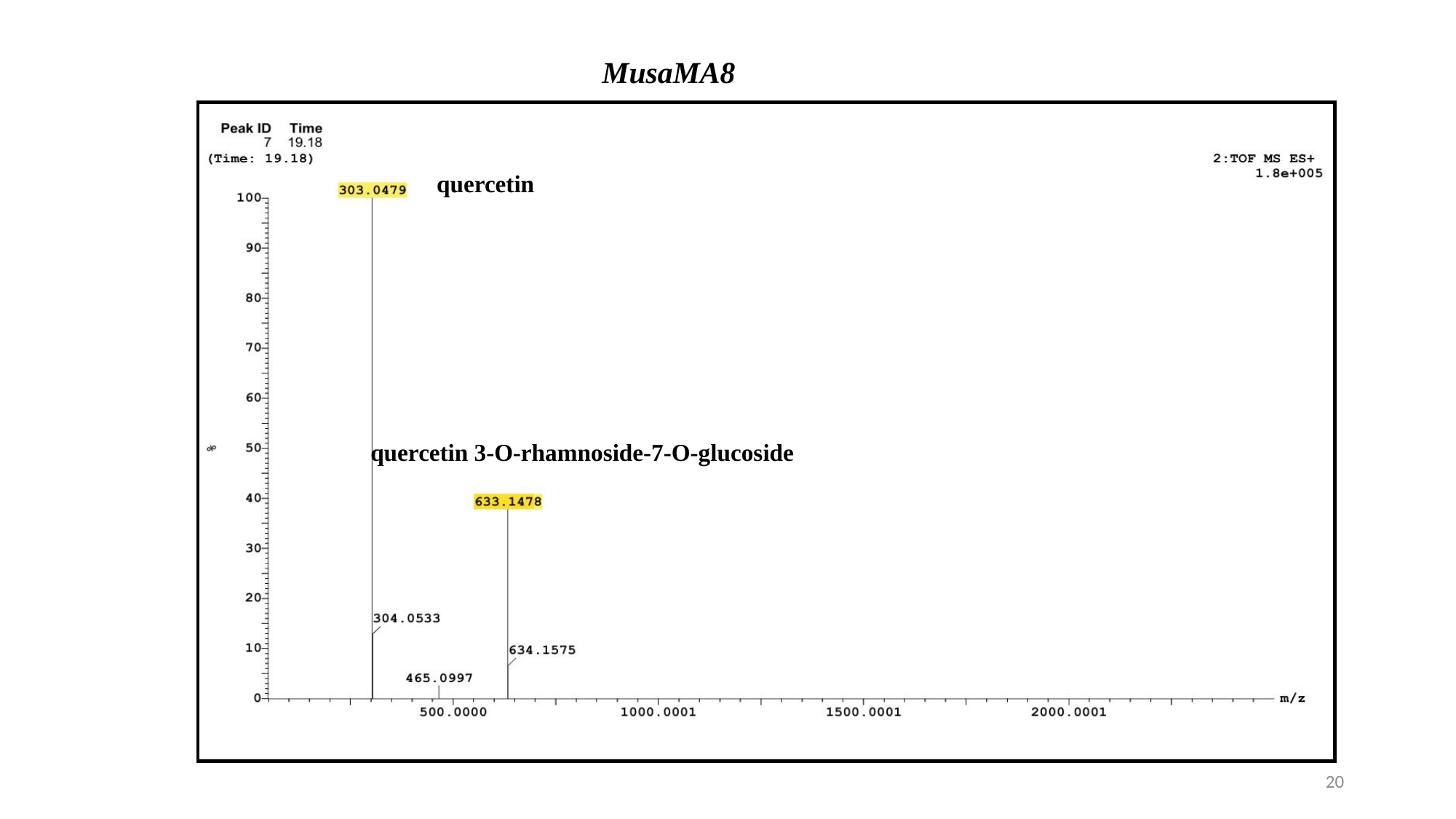

MusaMA8
quercetin
quercetin 3-O-rhamnoside-7-O-glucoside
20

## Slide 21
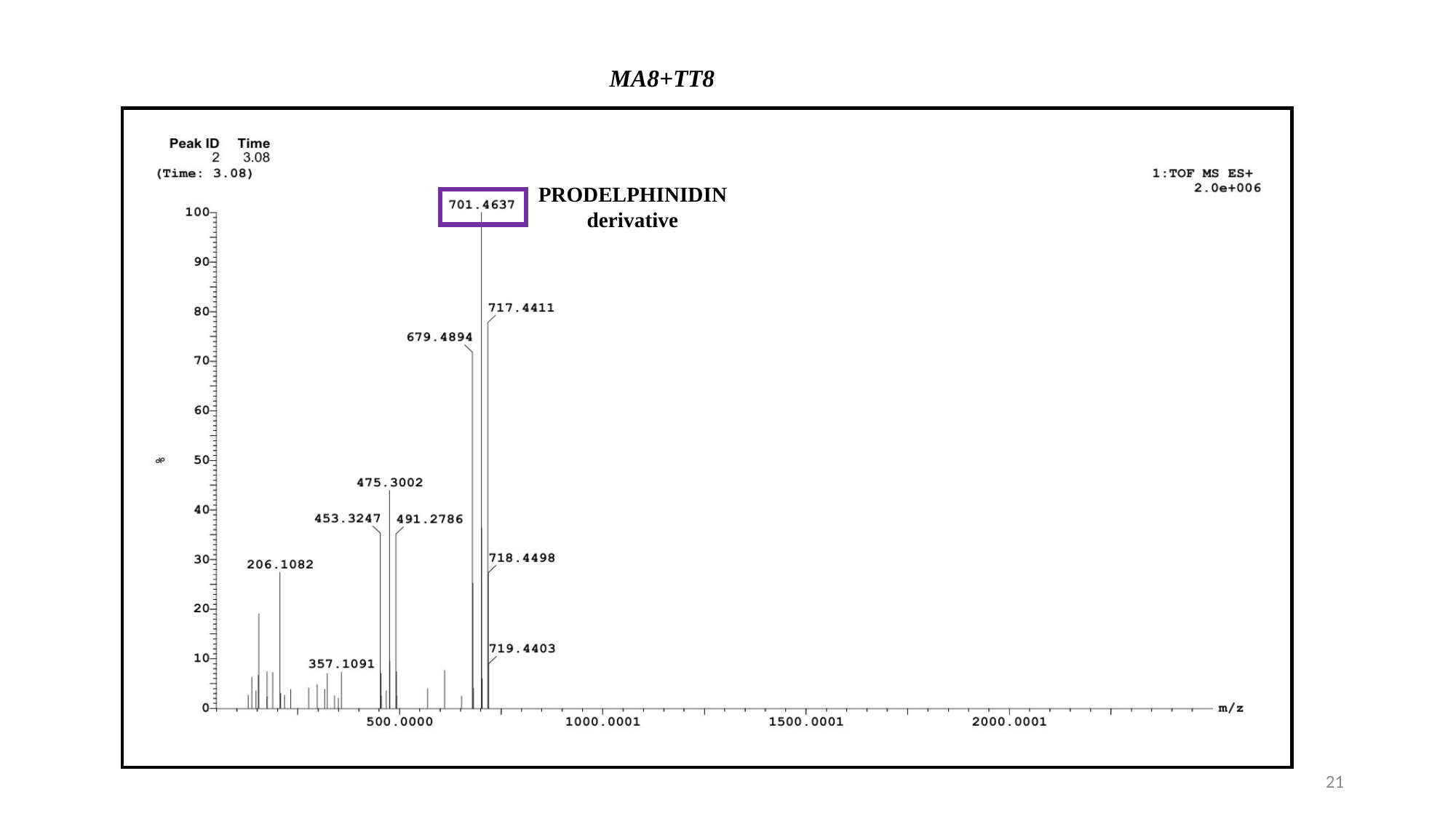

MA8+TT8
PRODELPHINIDIN
 derivative
21
