# Supplementary material for: A coordinated network of MYB regulators orchestrates anthocyanin biosynthesis in banana
Source: Hortic Res. 2026 Jan 13;13(6):uhaf361. doi: 10.1093/hr/uhaf361 (PMC13273576; doi:10.1093/hr/uhaf361)
Supplement: Web_Material_uhaf361 [file Web_Material_uhaf361.zip › Supplementary file S4.docx]

| Gene Code | Accession Number | Genomic Location |
| --- | --- | --- |
| *MaCHS1* | Ma06_t09770 | CAJGYN010000006.1:6790203:6792768:-1 |
| *MaANR* | Ma08_t01380 | CAJGYN010000008.1:1243657:1246442:-1 |
| *MaLAR* | Ma05_t16480 | CAJGYN010000005.1:16725706:16729540:-1 |
| *MaDFR* | Ma03_t32330 | CAJGYN010000003.1:34166830:34169662:-1 |
| *MaFLS1* | Ma03_t32330 | CAJGYN010000003.1:34167430:34170062:-1 |
| *MaCHI2* | Ma11_t21950 | CAJGYN010000011.1:25907361:25909877:1 |
| *MaANS* | Ma05_t03850 | CAJGYN010000005.1:2867005:2870583:-1 |
| *MaUFGT* | Ma01_t07150 | CAJGYN010000001.1:5151297:5154450:-1 |
| *MaTT8A* | Ma06_t24180 | CAJGYN010000006.1:22641183:22646554:-1 |

>*pCHS1*

GTGGAGGGTATGATATTCTGGGTGGTGTAATTTCTTAATCAATATTTGTAAAAACATTCCTAATTTAGACTTTTCTAGATACTTTCTTTATATATATATATATATATATATAATTTCGAATATATCTTAACCACATACTTCTTTTCTCACGAGAAAAATGGATAAAAATATGCTAAAACAATTTGGTACTTTCTGTCTTTTTCAACAACACTAACAAATGCAATAGCAATCACTCACCAAATGGCAAGCTTAAGGTCAACATTTTTACAACTTAAATTTTACATAATTTTTTTCTTCTTATCTTTTTTATATTTTTTTTAGGATTATCATTTTTACTTCGAAACTACACAAGAATGTCTACCATAAAGCAAAATTAAAAAAAGGTGTTCGAGTTGTGAGGGAAAAAATAAAAAAATAATTATATGATCAAAATAACTATTAATTATGCGATCAAAAACTATTTTAACTAAAACTTGATTCAATTAGACAGATCGATTTACCATATAGAAAAATAAGAAAAAATAGATAGGTGACTTACTATCAAATCGAAGTTATATTTGGAATTAGACAAAGAAAATTATAGAGAATTTTGAAAAAGGTATCTCTCGGAAAAGATCGAAAAAATATTTTTTTCCCAGGGAAATCACCCTAATTTCTTTACTTGTTAATAAACACTTCATGAAACACGTGGCATATCATCTAGGTTTTCCTTTGAAAAGTATTGTCTTTCTTGATAGGACACATTACACAACTCCATAACGACGTCAGCCATCACGTTTAATGAAGATGACGAGGCACTCACCAAACCCCTCGTGGCCTCACGTACTGCCTGTCCCTTCCACTTGTCATCTACTACAAATGTCCAAACTCCACGACATCCAAAGCAAGCCATCAACTGTTCGAGCTGCAATACTCGGCTTGTGGCAGTCGTTGGTGAAGATTAGAAGAGCCAGGAGTC

*>pANR*

GGTCGGATTCGGGTAGAGAAGTGGGACTTCACATAGCCTTTTCACGTCTTCTTCCTTCGTCACATCTTCTCCTCTCTATCTTCTGTCGTTCCTCCAAAGCTCCATTTATATGAGCGAGGGAAGGCGGAGGGCTCACTGCAGCATCGCGGGCGACGGCACGTAGGTTGAATCGGTTCAGTGAAGAGCAGCCCGTCCGATCGACCGAGCATGACGGATCGGTCCCACTCCACCAAAATTTGAAAGCTTGACTGGTAGCTCAGTCTACCGATGGGGAATCCAAACTATTTGAGTACCCCAAAGGAGCTTTTGTAGGTTAATTGATGAGATTAAAAGTGGTGTTCTGGAAGAGATCAAAAGAAAAAAGATGATGTCTTCCCCTTACCGAACATCTTTTTGATAGGTTCTTTGAGGAAAGTTCGATAGAGGTCCATCTTGTATTGCATATCATAGAGCATCCTCTTTCTTCGTATCTCATCATTTCTCATATCATAGACCATACTTTCCCAGACTCTCTTAAGATCTTAATAAACTAAATTCAATAGAACCTCAATATCCCATCAAAGTGATGAATTAAACACTTGTCTAAGAATTCATGTGGATGTCCAGTGTACCAGACAAATTTGTCTGACAGTAGCCTGGACAAGGGGCCATTTCTCCGGCTAACGTTCCTGCATAGTGTTACATTTTCACGTTGTTACCTGCCACCGAGTTCATCAAATGACTGGATTCATGGATTATGACCGGAGCAATATGGATAAGAGTTCCAATTTAGAGGCCCTGTTGAGTCGGAAATAACTCTTGGTGTTGTGGGGGTGACGAAGAAGCAGCCTTTGAACGGTGGTTAAGCTTTACTGTGGCTTCGTTGGTGAACACCCAACGTGATGATCTACCTGCACTTTCTGCGTCGTATTTAACGCCGTGCGAGTGGCCGAGAGGTGCACTGTGAGCCAGGATACAGCACTCGAAGAAGCGATCGAG

*>pANS*

TCTGCGTGTAATCTTCTCTCTTGCGTGCGTTAAGAACTTTTCTTATCGGTCTTCAAATCCCAAATCAGGTTTCATGTCAGTATGTTAGTTTGATCTCAACCCAATCCAAATCCAAAATTCTTTAATGGGAGTGCAAGCTCAAAGAATTTAGCCAACATTTATTATATGTATGTAATAAAAATATAAATCAATATATATATATCTATATTTTTTTTCCAGAAAAACATTGTCATTGTATTACTTAAAATCAGGGAATACAATCAACAGAATCAACAGTAAAAAAAAAGGAGGATCCCAAAATAATAATAATAATAATAATATAGTTGACGTGGCAAAGCACAATGGTTTCCTTCCATGAATACATGACAAACATTAACATCATCAAAAGAGCACATCAATGCTTTACTGAGATGGCCACATATCAGGGGAAGTGACGATGCGTAGGTAGTCCAAGTTAGCTCAATTTGCGTTGCGACCATCTCGACATGGTTTAGTCCAGACGTTGGTGTATGGTCTCTACTATGGGACCCACAGCGGATCCGATCCTTTACGTACTTGGACTAATCCGACGAATAGCCAACCCCATATGTCTACTTCAACGCATTCTCGCAGTTGCCGCATGCGAGAACTACCAACGTCGCTCCACGAAGACGGTTGGTTACACTGCGTTCGCATCAGGGCGGTTGGTGCGCACGTGCTTCCCTTTCTGGATTCACGTGCTTAATATCCCGTCAACGCCTCCTTCCGTGGCAGATCAAACCCACCGAATCGGATGGAGGGCCGAGATTGAAGGCTGGATTTGTTTTGTAGAGCGTGGCCCCACGTGTCACCTTAATTCCTTCTCTCAACCATGCCCTCGTTTTCATTGTCGTGGCTGTGACCACCGGCGCCATTCATACTGTAGTACCATGATGCCCCCCTGCACACCGGTTAGGTGAAGAAGTTAGCTGTAGCAGGTGCGGGGCATCCGCCCCTGGTTTCTCCGCTCACGTTTGCTGCCAGTAGCGGAAGCTTCTCCTATATATGTACTGCAGCCTTCCTCGTTGGGACAGCAGCAGCAAGCAGCAGCCCTCTTGGAATTGTTCCGACGAGGACCGAGAGAGACCAG

>pUFGT

GTGGGAGACAACAAACAATCTAAAGTATAAAAAAATTGTTTTGTTAATAAAGATCTAAGGTGCAAAGAATTGATCTAAAGCACATGATATCATGTGTCATAAGATTGAAAGTAATATTTGTTGGAAGGTAAAAGGGCTCGTCATCATCATCTCGCATTTGTATATGATTCGACTTAGCTATGTTTAGCTATCGTGTTGCTGTCAATTTAGGGATTTCCGTTTAGTGACTGGTTTTATCTTCATCATTGTTAGGAGAATTTTTATGTAAGATATGAAAGTGGAAGGAGGATCAAAAGAATAGTTTCATATTGTTAATGATCGAAAGAATCAAATGGCATATATTGAGTTTAATATATAATTTTAATAAACTTATTTTTTATATTTAAATTATTGAATTGGTCCACATCATTATGTTAATTTTAAATAGTTTGATTGATATAGTTGAGTTTTTATAATTTGATTGATCAATCCATATATCTTATATTAAAAAGGGATAGGGCATAATCTTAAAAGACTTTAACGAGTGTTAGTAGAAAATATTGTTAATATCTTTATGAACCCGATGTGGTTCAGACTCATATATACCGGAATGTTCGAGATATTTCTGAGGTAAAAATATTGTGCTCCCTCCAATGTGGAAATTATATGCTCTCGAGATAAAAAAATATTATATTTTCGAGATATCACATACACGAAGACTAGATCGAAAGATATTTTTCAACTTGATCTCTCCAATGTATAAGTTAGTAATTCGAGATTCCTAGATATGGGTTAGAATAGTTTCAAAAAGGAAAGAGCGAGAGCCTTTTTTTTATTATTTAAAAAATATATTTTATACCTTAAAGATAAGAATAAAGTTTATAATTATCTTCCTTGTCATAAAAAAAATAAGTTTTAGATTGTCTTTATCGTTAAAAATAATAACAAAATTTATGATTATATTTTTTATGTTTCCTTAGATTTTTATCAAGGATGATTTAGATCTTATATGGCAATCACGTGTTGACTAACCTTGTATCTCGCTTCCAAGTTAAAGAGACAAATCTACCTACGCAACATCTAGAAACTTTGAGATCTAAATCATTCTTCCATCAACCAAGTAATATTTATCTTCGCCCCCACCACAATATATATCCACCCAACATCTAGAAACTTCGAGATCCAAATCATTCTTCGATCAACCAATTAATATATATCTTCGGTCAATGTTTCTTCCAATTTTCGTTGTCATCAGACGAACTCGATGACATCTTCGCTGACGTGGCCACGTGTTGACTGATAGGTGATTGAATGATGTGGGTGTGATCGACTTCAAATCAAGTAGCAGTATTTACCTCTGCACTCCAACGCAGTATTTACCACTGCGGTCGGTGTCGCCACCCTGTGCTATAAAAGTGGCCTTATATAATTCGAAGATGTCGCTTCGGACCAGAGAGAGAGAGAGAGAGAGAGAGAGGAC

>pLAR

ACTGTGTTAGTCAGACTAAAGCTGAAGGAGACGTCTAAGCCGTACCTAACGGCCTTCCCAAAGACCTTCAACAGTTCCCGGTCACCATACTCTTCTTCTTCTTCTACCAGGTGATCCTGAAGCAGCAATGGGATACAACAGCAGGTTTTTCTTCTTCCAGGTGATCCAGAAGCAGCGATGGGATACAACAGATTGAGATTGGAAGAGAGGGAGGCGGAGAAAGAAGAAGACGAGAGGAAACACTCGTTGACTTAGTGTAGTATCTTATTATAGTCAATGGTAATAAAAGTGGCAAAACGTGCGACATGTTGGCAGTCGCCATCGTCCCTTCCCACGTCCGATGACTTATTCCCTGCCCCAAAGATATTTGCATGCAATCTTGTCTTACCATGTGGAAATTACGAATGGGATCCACAGCTCGCGTCGTATCCTACCCGATGTTCCACGGCACCGCTCCTCGTGCCTCGTGAGCCCAACTTCTTTTCCTGGTTAGAAAAGTCTTGCAATCAATAGTAATAATGATATGATAACATCAGGATGACCGGTCAATCAATCGCGGTATAACGCGTGTGCATGAAGATGTTATTCTCATCACCGGTAGCCTCGACACGGTCTGCATCGTAGCAATGCTTCCAATTCTATAACTTCAACCTAACACTTCCTCTCCCTGGAAATTTACTATTCGGCAAATGCAGATACTGCTCAAGTATGAACATGTACTGTTCATCATACATTACATGATGAAGGCTGTACTGTACGAGGTAATGCAAAGCAAAGTGCACGATAGGCGATCCTTGACGGTTAGGTGCAATCAAGACCGTCGGAGGGATGGATCCTGTACGTCTCCCCATGTGCATGTGGCTTTATATGATCAGCCCGGGCGGCCGCTTCGCTTTGCCCACTGCTCCTTCCCCACCCTCTAATTCTTTCAGCATATTCTCC

>pTT8

ATCATAAATGGCATCTATTAGCAATGTCTACTCCAATTGGTGATGACGTGGACTTGAGAATAAGCAGGTAGGGAAAATGACAACAGTGACTTTGAAGAAGACGGCTAACGGAAGGCAATATTTTAGCCTATTTAAATTAAATGGACAGCTAAAGCTGGTGTAGTTTTAGTAGGTGATACCACGAATATTCTGTACCAATCGGATAACGACAGGTGGACCTTAAGATGATCATTCTAGACTCAGGCCTTGTCAAAATGGATCGACGCCAAATGTCATCAGACAATATTTTTTTGCATGGATGTCGAGAATGGTTCGACCTGATTCAGACATGAAACCAAAGCAGGTCAATCTCGTCTAATGTGGCAAGATGCCGAACTAGAAGAACTGAGTGCTAGAGTCGAAGCGTCGAGGTTATTCTTTCCGAAAACCATAAAGGTTTCTTAGATAGTTGAGCTAATGCAGGAGAAGACTGATGTTAGATTTTGCTGGAGTCTCTGGTCCTTCCATGTTCATGTAAATATATATATTTTTTAAGATGACCGTTGACATGAGAAATTTGACATAATAGATTTTATCACTTTCGTGTACACGTGTCAAATATTTATTAAAAGCATTCTTTATCTCGACGCTAAAAGAGAAATTTAAAGAAATGATAAAAAATTGACGCGAAGATTTACGTGATTCGATAACGAACAAACAATGTTGAGTTGGGCCGGAGATGAACTGAGACTGAAGCTATAGCGTTGTTGTCACCGTGGATTCGGCAATGGCGTCGTTACGTGGGACACCAGCACTATGCCCACCAAGTGGTCGCTATAAGACCGAGCAGGTGAGAGCCGACTGGTAGAGCGACCAGTCAAAACCAAACCCAACGTGGAATCCCCCCTCCCCCCCGCCTCCCACCACCCGGGGCAAGTCACTTCCCAATTCTTCGCACTTCCACTTCCCTTTCTTCCCCCTTCCTCAAATCCACCACCAAACAGTCCGCCTGCACCTCTCAACCACCACCACCCCCCCTCTCTGTCTCTCTCTCTCACCCTCTTTTATTTTAATCCCCCTCCTTGTAGCAAGCAAGCGGTTCCTTACGCCGGAGGCCGCC

>pFLS

TAAAGAGAGTGAGTGATTACATATGCCAATTATTTTTTTCGCACACATTTATATAAAGCATGTGACATGATAATGATTTTGGCCAACACAAATTTATATCTGACTTATGTCATCATATATGAAGGAAATTTGTTGATCGTATGTTTGTGTTACACTGAATTGATGTAATATACGTTTAACGGTATATTAGAATTGTAATTCTTAAATAATATGATATATTTTGAATGATGTTTTCTTGGCACTCTACTTGGAAACTAAGTGCATCTTTCCTTCTCATTGATCATCATTTCTCTATGAATTCTCCATCATTATGCTCTTCAATCTTGCGATCGTTTCTCGATTTCATGATTCTAAATTTCTTGTTGACTCTGTGAATCTAATCGCTATTTCACTCTATTATTTGTCTGTGTGGATAGGAGTAATCGATCATAGAGAAATGGATGTTCTTCTACGTAGTATTATTTAGTAGACACTGTCAGAAACCTAGCAGAGATATCACTGCACTTGGTTGCAATGAATGCCACCTGTTTACATGTATGTCACGAGGGACATGAGCAATCTGGATCGCCCGTATCAACTACCTCTCTAAATCCAGCCTCGAATCTGATCCAATTTCTAGAAGTGAAAGCTGCCGTTCATCCACACAAATTAAATTTCCATGATTTAGCTGGTGGACACGACAGCAAATCCAACACACAACTGCTAACATTGATGGTAATGCAAAATACTCCAGATAGAGGATGCTAAGAGTTATCGCCAGACTTTGTATAGTCTCAAAGTCTCTCTCTCTCTCTCTCTCTCTCTAAGTCTCAGGTTGGTAGGGATAACAAGTGAATAGCGTGAATGCACGTCAGAAGAGAAAACATGGAGGATAAGTACTGCTACCAACAACAAAGGTTGAAACTAACCTCCTCTCCCGTATTTATAACACCCTCCGCCGCTTCTCAAGTCCGACCACCACAACCACTTTCTTTGCTGCTTCCTCAGCAAAGACCGCAGG
