# Supplementary material for: A coordinated network of MYB regulators orchestrates anthocyanin biosynthesis in banana
Source: Hortic Res. 2026 Jan 13;13(6):uhaf361. doi: 10.1093/hr/uhaf361 (PMC13273576; doi:10.1093/hr/uhaf361)
Supplement: Web_Material_uhaf361 [file Web_Material_uhaf361.zip › supplementary file S6 (1).pdf]

	Sample Name	Sample ID	Sample Type	File Name	Analyte Peak Area (counts)
163	Jayram_09092025_38		Unknown	28072025_Akhil_Dr	0.00e+000
164	Jayram_09092025_39		Unknown	28072025_Akhil_Dr	0.00e+000
165	Jayram_09092025_4		Unknown	28072025_Akhil_Dr	2.46e+003
166	Jayram_09092025_5		Unknown	28072025_Akhil_Dr	0.00e+000
167	Jayram_09092025_6		Unknown	28072025_Akhil_Dr	0.00e+000
168	Jayram_09092025_7		Unknown	28072025_Akhil_Dr	0.00e+000
169	Jayram_09092025_8		Unknown	28072025_Akhil_Dr	8.36e+002
170	Jayram_09092025_9		Unknown	28072025_Akhil_Dr	2.45e+003

Sample Name: "08092025_Sample_34_TR3" Sample ID: "" File: "08092025_Sample_34_TR3.wiff"
Peak Name: "cynidin3retinoside" Mass(es): "595.000/287.000 Da"
Comment: "" Annotation: ""

```
Sample Index:        1
Sample Type:      Unknown
Concentration:       N/A
Calculated Conc:     5.77     ng/mL
Acq. Date:         9/9/2025
Acq. Time:         3:36:31 PM

Modified:            Yes
Proc. Algorithm: Analyst Classic
Bunching Factor:     1
Noise Threshold:   13.19    cps
Area Threshold:   65.95    cps
,Num. Smooths:        0
Sep. Width:          0.20
Sep. Height:         0.01
Exp. Peak Ratio:     5.00
Exp. Adj. Ratio:     4.00
Exp. Val. Ratio:     3.00
RT Window:          30.0     sec
Expected RT:         7.31     min
Use Relative RT:     No

Int. Type:           Manual
Retention Time:      7.42     min
Area:            3.44e+002    counts
Height:            2.06e+001  cps
Start Time:          6.74     min
End Time:            7.58     min
```

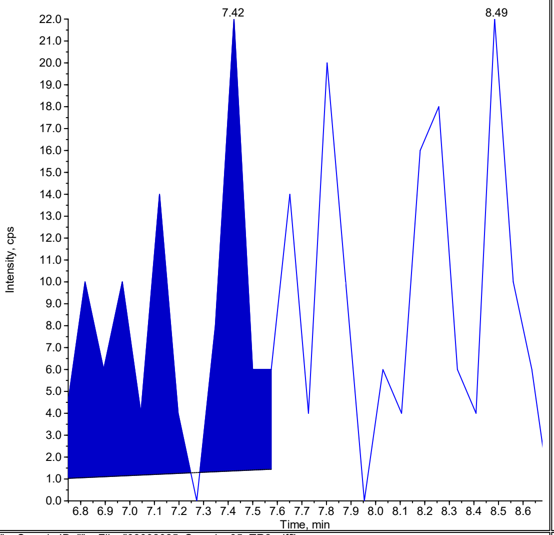

Sample Name: "08092025_Sample_35_TR1" Sample ID: "" File: "08092025_Sample_35_TR1.wiff"
Peak Name: "cynidin3retinoside" Mass(es): "595.000/287.000 Da"
Comment: "" Annotation: ""

```
Sample Index:        1
Sample Type:      Unknown
Concentration:       N/A
Calculated Conc:     56.3     ng/mL
Acq. Date:         9/9/2025
Acq. Time:         3:52:41 PM

Modified:            No
Proc. Algorithm: Analyst Classic
Bunching Factor:     1
Noise Threshold:   13.19    cps
Area Threshold:   65.95    cps
,Num. Smooths:        0
Sep. Width:          0.20
Sep. Height:         0.01
Exp. Peak Ratio:     5.00
Exp. Adj. Ratio:     4.00
Exp. Val. Ratio:     3.00
RT Window:          30.0     sec
Expected RT:         7.31     min
Use Relative RT:     No

Int. Type:           Valley
Retention Time:      7.25     min
Area:            6.45e+003    counts
Height:            6.25e+002  cps
Start Time:          6.74     min
End Time:            7.58     min
```

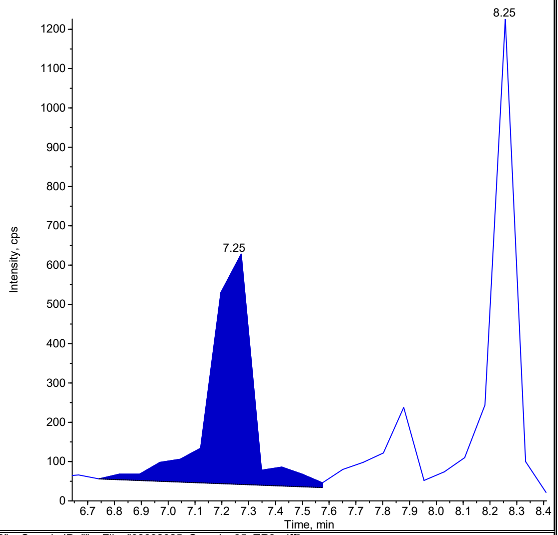

Sample Name: "08092025_Sample_35_TR2" Sample ID: "" File: "08092025_Sample_35_TR2.wiff"
Peak Name: "cynidin3retinoside" Mass(es): "595.000/287.000 Da"
Comment: "" Annotation: ""

```
Sample Index:        1
Sample Type:      Unknown
Concentration:       N/A
Calculated Conc:     69.1     ng/mL
Acq. Date:         9/9/2025
Acq. Time:         4:08:51 PM

Modified:            Yes
Proc. Algorithm: Analyst Classic
Bunching Factor:     1
Noise Threshold:   13.19    cps
Area Threshold:   65.95    cps
,Num. Smooths:        0
Sep. Width:          0.20
Sep. Height:         0.01
Exp. Peak Ratio:     5.00
Exp. Adj. Ratio:     4.00
Exp. Val. Ratio:     3.00
RT Window:          30.0     sec
Expected RT:         7.31     min
Use Relative RT:     No

Int. Type:           Manual
Retention Time:      7.20     min
Area:            8.00e+003    counts
Height:            7.68e+002  cps
Start Time:          6.59     min
End Time:            7.50     min
```

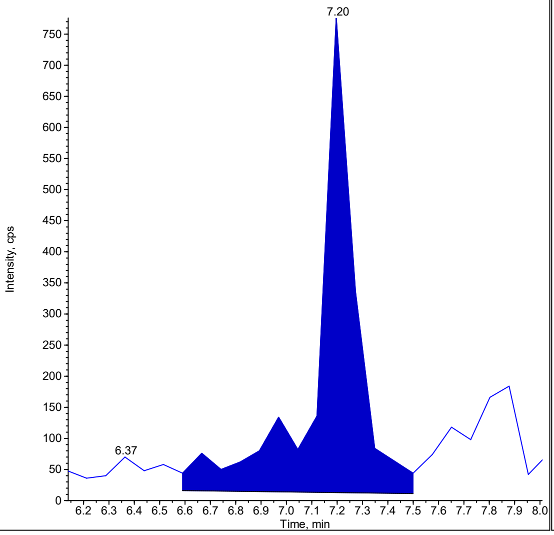

Sample Name: "08092025_Sample_35_TR3" Sample ID: "" File: "08092025_Sample_35_TR3.wiff"
Peak Name: "cynidin3retinoside" Mass(es): "595.000/287.000 Da"
Comment: "" Annotation: ""

```
Sample Index:        1
Sample Type:      Unknown
Concentration:       N/A
Calculated Conc:     78.1     ng/mL
Acq. Date:         9/9/2025
Acq. Time:         4:25:02 PM

Modified:            Yes
Proc. Algorithm: Analyst Classic
Bunching Factor:     1
Noise Threshold:   13.19    cps
Area Threshold:   65.95    cps
,Num. Smooths:        0
Sep. Width:          0.20
Sep. Height:         0.01
Exp. Peak Ratio:     5.00
Exp. Adj. Ratio:     4.00
Exp. Val. Ratio:     3.00
RT Window:          30.0     sec
Expected RT:         7.31     min
Use Relative RT:     No

Int. Type:           Manual
Retention Time:      7.27     min
Area:            9.10e+003    counts
Height:            1.32e+003  cps
Start Time:          6.89     min
End Time:            7.58     min
```

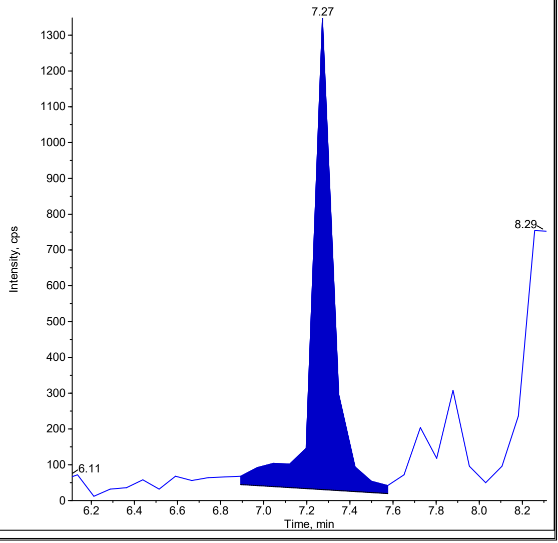

	Sample Name	Sample ID	Sample Type	File Name	Analyte Peak Area (counts)
1	Std_4_Dil_1		Standard	28072025_Akhil_Dr	3.03e+004
2	Std_4_Dil_2		Standard	28072025_Akhil_Dr	1.36e+004
3	Std_4_Dil_3		Standard	28072025_Akhil_Dr	7.74e+003
4	Std_4_Dil_4		Standard	28072025_Akhil_Dr	3.98e+003
5	Std_4_Dil_5		Standard	28072025_Akhil_Dr	1.19e+003
6	Sample_1		Unknown	28072025_Akhil_Dr	4.94e+005
7	Sample_4		Unknown	28072025_Akhil_Dr	4.78e+006
8	Sample_5		Unknown	28072025_Akhil_Dr	2.04e+006
9	Sample_6		Unknown	28072025_Akhil_Dr	8.91e+005
10	Sample_7		Unknown	28072025_Akhil_Dr	2.19e+004
11	Sample_8		Unknown	28072025_Akhil_Dr	2.74e+004
12	Sample_9		Unknown	28072025_Akhil_Dr	0.00e+000
13	Sample_10		Unknown	28072025_Akhil_Dr	6.01e+004
14	Sample_11		Unknown	28072025_Akhil_Dr	3.90e+004
15	Sample_12		Unknown	28072025_Akhil_Dr	1.31e+005
16	Sample_13		Unknown	28072025_Akhil_Dr	0.00e+000
17	Sample_14		Unknown	28072025_Akhil_Dr	1.45e+005
18	Sample_15		Unknown	28072025_Akhil_Dr	8.97e+005
19	Sample_16		Unknown	28072025_Akhil_Dr	7.19e+004
20	Sample_17		Unknown	28072025_Akhil_Dr	1.44e+005
21	Sample_18		Unknown	28072025_Akhil_Dr	6.38e+005
22	Sample_19		Unknown	28072025_Akhil_Dr	2.11e+005
23	Sample_20		Unknown	28072025_Akhil_Dr	3.90e+004
24	Sample_21		Unknown	28072025_Akhil_Dr	0.00e+000
25	Sample_22		Unknown	28072025_Akhil_Dr	0.00e+000
26	Sample_23		Unknown	28072025_Akhil_Dr	0.00e+000
27	Sample_24		Unknown	28072025_Akhil_Dr	0.00e+000
28	Sample_25		Unknown	28072025_Akhil_Dr	1.54e+004
29	Sample_3		Unknown	28072025_Akhil_Dr	2.72e+006
30	Sample_2		Unknown	28072025_Akhil_Dr	7.13e+005
31	08092025_Sample_1_TR1		Unknown	28072025_Akhil_Dr	1.72e+003
32	08092025_Sample_1_TR2		Unknown	28072025_Akhil_Dr	1.65e+003
33	08092025_Sample_1_TR3		Unknown	28072025_Akhil_Dr	1.77e+003
34	08092025_Sample_2_TR1		Unknown	28072025_Akhil_Dr	1.45e+003
35	08092025_Sample_2_TR2		Unknown	28072025_Akhil_Dr	1.72e+003
36	08092025_Sample_2_TR3		Unknown	28072025_Akhil_Dr	1.37e+003
37	08092025_Sample_3_TR1		Unknown	28072025_Akhil_Dr	3.62e+003
38	08092025_Sample_3_TR2		Unknown	28072025_Akhil_Dr	2.49e+003
39	08092025_Sample_3_TR3		Unknown	28072025_Akhil_Dr	2.62e+003
40	08092025_Sample_4_TR1		Unknown	28072025_Akhil_Dr	3.87e+003
41	08092025_Sample_4_TR2		Unknown	28072025_Akhil_Dr	1.41e+003
42	08092025_Sample_4_TR3		Unknown	28072025_Akhil_Dr	1.24e+003
43	08092025_Sample_5_TR1		Unknown	28072025_Akhil_Dr	1.88e+004
44	08092025_Sample_5_TR2		Unknown	28072025_Akhil_Dr	2.42e+003
45	08092025_Sample_5_TR3		Unknown	28072025_Akhil_Dr	1.84e+003

	Sample Name	Sample ID	Sample Type	File Name	Analyte Peak Area (counts)
46	08092025_Sample_6_TR1		Unknown	28072025_Akhil_Dr	2.65e+003
47	08092025_Sample_6_TR2		Unknown	28072025_Akhil_Dr	1.57e+003
48	08092025_Sample_6_TR3		Unknown	28072025_Akhil_Dr [illegible]	3.74e+003
49	08092025_Sample_7_TR1		Unknown	28072025_Akhil_Dr	2.28e+004
50	08092025_Sample_7_TR2		Unknown	28072025_Akhil_Dr	3.33e+004
51	08092025_Sample_7_TR3		Unknown	28072025_Akhil_Dr	3.31e+004
52	08092025_Sample_8_TR1		Unknown	28072025_Akhil_Dr	5.81e+004
53	08092025_Sample_8_TR2		Unknown	28072025_Akhil_Dr	6.07e+004
54	08092025_Sample_8_TR3		Unknown	28072025_Akhil_Dr	6.96e+004
55	08092025_Sample_9_TR1		Unknown	28072025_Akhil_Dr	1.28e+004
56	08092025_Sample_9_TR2		Unknown	28072025_Akhil_Dr	1.20e+004
57	08092025_Sample_9_TR3		Unknown	28072025_Akhil_Dr	1.27e+004
58	08092025_Sample_10_TR1		Unknown	28072025_Akhil_Dr	5.29e+004
59	08092025_Sample_10_TR2		Unknown	28072025_Akhil_Dr	5.41e+004
60	08092025_Sample_10_TR3		Unknown	28072025_Akhil_Dr	4.65e+004
61	08092025_Sample_11_TR1		Unknown	28072025_Akhil_Dr	2.52e+004
62	08092025_Sample_11_TR2		Unknown	28072025_Akhil_Dr	4.41e+004
63	08092025_Sample_11_TR3		Unknown	28072025_Akhil_Dr	4.79e+004
64	08092025_Sample_12_TR1		Unknown	28072025_Akhil_Dr	5.13e+004
65	08092025_Sample_12_TR2		Unknown	28072025_Akhil_Dr	4.42e+004
66	08092025_Sample_12_TR3		Unknown	28072025_Akhil_Dr	3.91e+004
67	08092025_Sample_13_TR1		Unknown	28072025_Akhil_Dr	1.20e+004
68	08092025_Sample_13_TR2		Unknown	28072025_Akhil_Dr	1.83e+004
69	08092025_Sample_13_TR3		Unknown	28072025_Akhil_Dr	1.73e+004
70	08092025_Sample_14_TR1		Unknown	28072025_Akhil_Dr	3.77e+004
71	08092025_Sample_14_TR2		Unknown	28072025_Akhil_Dr	3.87e+004
72	08092025_Sample_14_TR3		Unknown	28072025_Akhil_Dr	4.01e+004
73	08092025_Sample_15_TR1		Unknown	28072025_Akhil_Dr	3.40e+004
74	08092025_Sample_15_TR2		Unknown	28072025_Akhil_Dr	4.59e+004
75	08092025_Sample_15_TR3		Unknown	28072025_Akhil_Dr	3.05e+004
76	08092025_Sample_16_TR1		Unknown	28072025_Akhil_Dr	1.40e+004
77	08092025_Sample_16_TR2		Unknown	28072025_Akhil_Dr	1.71e+004
78	08092025_Sample_16_TR3		Unknown	28072025_Akhil_Dr	1.56e+004
79	08092025_Sample_17_TR1		Unknown	28072025_Akhil_Dr	4.11e+003
80	08092025_Sample_17_TR2		Unknown	28072025_Akhil_Dr	1.16e+004
81	08092025_Sample_17_TR3		Unknown	28072025_Akhil_Dr	7.49e+003
82	08092025_Sample_18_TR1		Unknown	28072025_Akhil_Dr	1.79e+004
83	08092025_Sample_18_TR2		Unknown	28072025_Akhil_Dr	1.65e+004
84	08092025_Sample_18_TR3		Unknown	28072025_Akhil_Dr	1.53e+004
85	08092025_Sample_19_TR1		Unknown	28072025_Akhil_Dr	1.26e+004
86	08092025_Sample_19_TR2		Unknown	28072025_Akhil_Dr	2.16e+004
87	08092025_Sample_19_TR3		Unknown	28072025_Akhil_Dr	1.45e+004
88	08092025_Sample_20_TR1		Unknown	28072025_Akhil_Dr	1.51e+005
89	08092025_Sample_20_TR2		Unknown	28072025_Akhil_Dr	1.41e+005
90	08092025_Sample_20_TR3		Unknown	28072025_Akhil_Dr	1.61e+005

	Sample Name	Sample ID	Sample Type	File Name	Analyte Peak Area (counts)
91	08092025_Sample_21_TR1		Unknown	28072025_Akhil_Dr	1.55e+005
92	08092025_Sample_21_TR2		Unknown	28072025_Akhil_Dr	1.65e+005
93	08092025_Sample_21_TR3		Unknown	28072025_Akhil_Dr	1.35e+005
94	08092025_Sample_22_TR1		Unknown	28072025_Akhil_Dr	4.48e+004
95	08092025_Sample_22_TR2		Unknown	28072025_Akhil_Dr	5.24e+004
96	08092025_Sample_22_TR3		Unknown	28072025_Akhil_Dr	6.67e+004
97	08092025_Sample_23_TR1		Unknown	28072025_Akhil_Dr	8.32e+004
98	08092025_Sample_23_TR2		Unknown	28072025_Akhil_Dr	6.78e+004
99	08092025_Sample_23_TR3		Unknown	28072025_Akhil_Dr	7.37e+004
100	08092025_Sample_24_TR1		Unknown	28072025_Akhil_Dr	1.53e+005
101	08092025_Sample_24_TR2		Unknown	28072025_Akhil_Dr	1.45e+005
102	08092025_Sample_24_TR3		Unknown	28072025_Akhil_Dr	1.46e+005
103	08092025_Sample_25_TR1		Unknown	28072025_Akhil_Dr	9.35e+003
104	08092025_Sample_25_TR2		Unknown	28072025_Akhil_Dr	9.08e+003
105	08092025_Sample_25_TR3		Unknown	28072025_Akhil_Dr	7.65e+003
106	08092025_Sample_26_TR1		Unknown	28072025_Akhil_Dr	1.71e+003
107	08092025_Sample_26_TR2		Unknown	28072025_Akhil_Dr	1.76e+003
108	08092025_Sample_26_TR3		Unknown	28072025_Akhil_Dr	2.12e+003
109	08092025_Sample_27_TR1		Unknown	28072025_Akhil_Dr	1.71e+003
110	08092025_Sample_27_TR2		Unknown	28072025_Akhil_Dr	1.41e+003
111	08092025_Sample_27_TR3		Unknown	28072025_Akhil_Dr	1.26e+003
112	08092025_Sample_28_TR1		Unknown	28072025_Akhil_Dr	2.20e+003
113	08092025_Sample_28_TR2		Unknown	28072025_Akhil_Dr	1.60e+003
114	08092025_Sample_28_TR3		Unknown	28072025_Akhil_Dr	1.97e+003
115	08092025_Sample_29_TR1		Unknown	28072025_Akhil_Dr	9.50e+004
116	08092025_Sample_29_TR2		Unknown	28072025_Akhil_Dr	1.09e+005
117	08092025_Sample_29_TR3		Unknown	28072025_Akhil_Dr	1.17e+005
118	08092025_Sample_30_TR1		Unknown	28072025_Akhil_Dr	8.01e+004
119	08092025_Sample_30_TR2		Unknown	28072025_Akhil_Dr	7.84e+004
120	08092025_Sample_30_TR3		Unknown	28072025_Akhil_Dr	6.81e+004
121	08092025_Sample_31_TR1		Unknown	28072025_Akhil_Dr	6.39e+002
122	08092025_Sample_31_TR2		Unknown	28072025_Akhil_Dr	4.30e+002
123	08092025_Sample_31_TR3		Unknown	28072025_Akhil_Dr	4.27e+002
124	08092025_Sample_32_TR1		Unknown	28072025_Akhil_Dr	4.61e+002
125	08092025_Sample_32_TR2		Unknown	28072025_Akhil_Dr	5.04e+002
126	08092025_Sample_32_TR3		Unknown	28072025_Akhil_Dr	5.26e+002
127	08092025_Sample_33_TR1		Unknown	28072025_Akhil_Dr	8.99e+002
128	08092025_Sample_33_TR2		Unknown	28072025_Akhil_Dr	9.12e+002
129	08092025_Sample_33_TR3		Unknown	28072025_Akhil_Dr	1.03e+003
130	08092025_Sample_34_TR1		Unknown	28072025_Akhil_Dr	2.50e+002
131	08092025_Sample_34_TR2		Unknown	28072025_Akhil_Dr	3.09e+002
132	08092025_Sample_34_TR3		Unknown	28072025_Akhil_Dr	3.44e+002
133	08092025_Sample_35_TR1		Unknown	28072025_Akhil_Dr	6.45e+003
134	08092025_Sample_35_TR2		Unknown	28072025_Akhil_Dr	8.00e+003
135	08092025_Sample_35_TR3		Unknown	28072025_Akhil_Dr	9.10e+003

	Sample Name	Sample ID	Sample Type	File Name	Analyte Peak Area (counts)
136	Jayram_09092025_1		Unknown	28072025_Akhil_Dr	0.00e+000
137	Jayram_09092025_10		Unknown	28072025_Akhil_Dr	5.91e+002
138	Jayram_09092025_11		Unknown	28072025_Akhil_Dr	1.71e+003
139	Jayram_09092025_12		Unknown	28072025_Akhil_Dr	2.50e+003
140	Jayram_09092025_13		Unknown	28072025_Akhil_Dr	2.07e+003
141	Jayram_09092025_15		Unknown	28072025_Akhil_Dr	0.00e+000
142	Jayram_09092025_16		Unknown	28072025_Akhil_Dr	0.00e+000
143	Jayram_09092025_17		Unknown	28072025_Akhil_Dr	0.00e+000
144	Jayram_09092025_18		Unknown	28072025_Akhil_Dr	1.17e+003
145	Jayram_09092025_19		Unknown	28072025_Akhil_Dr	0.00e+000
146	Jayram_09092025_2		Unknown	28072025_Akhil_Dr	0.00e+000
147	Jayram_09092025_21		Unknown	28072025_Akhil_Dr	2.23e+003
148	Jayram_09092025_22		Unknown	28072025_Akhil_Dr	8.23e+002
149	Jayram_09092025_23		Unknown	28072025_Akhil_Dr	0.00e+000
150	Jayram_09092025_24		Unknown	28072025_Akhil_Dr	0.00e+000
151	Jayram_09092025_26		Unknown	28072025_Akhil_Dr	5.14e+004
152	Jayram_09092025_27		Unknown	28072025_Akhil_Dr	0.00e+000
153	Jayram_09092025_28		Unknown	28072025_Akhil_Dr	0.00e+000
154	Jayram_09092025_29		Unknown	28072025_Akhil_Dr	0.00e+000
155	Jayram_09092025_3		Unknown	28072025_Akhil_Dr	0.00e+000
156	Jayram_09092025_30		Unknown	28072025_Akhil_Dr	0.00e+000
157	Jayram_09092025_31		Unknown	28072025_Akhil_Dr	0.00e+000
158	Jayram_09092025_32		Unknown	28072025_Akhil_Dr	0.00e+000
159	Jayram_09092025_33		Unknown	28072025_Akhil_Dr	1.79e+003
160	Jayram_09092025_35		Unknown	28072025_Akhil_Dr	1.60e+003
161	Jayram_09092025_36		Unknown	28072025_Akhil_Dr	0.00e+000
162	Jayram_09092025_37		Unknown	28072025_Akhil_Dr	0.00e+000
163	Jayram_09092025_38		Unknown	28072025_Akhil_Dr	0.00e+000
164	Jayram_09092025_39		Unknown	28072025_Akhil_Dr	0.00e+000
165	Jayram_09092025_4		Unknown	28072025_Akhil_Dr	2.46e+003
166	Jayram_09092025_5		Unknown	28072025_Akhil_Dr	0.00e+000
167	Jayram_09092025_6		Unknown	28072025_Akhil_Dr	0.00e+000
168	Jayram_09092025_7		Unknown	28072025_Akhil_Dr	0.00e+000
169	Jayram_09092025_8		Unknown	28072025_Akhil_Dr	8.36e+002
170	Jayram_09092025_9		Unknown	28072025_Akhil_Dr	2.45e+003

	Sample Name	Analyte Peak Height (cps)	Analyte Concentration (ng/mL)	Standard Query Status	Use Record	Record Modified
1	Std_4_Dil_1	3.85e+003	250.	Pass	☑	☐
2	Std_4_Dil_2	1.83e+003	125.	Pass	☑	☐
3	Std_4_Dil_3	1.07e+003	62.5	Pass	☑	☐
4	Std_4_Dil_4	4.80e+002	31.3	Pass	☑	☐
5	Std_4_Dil_5	1.82e+002	15.6	Pass	☑	☑
6	Sample_1	8.65e+004	N/A	N/A		☐
7	Sample_4	7.85e+005	N/A	N/A		☐
8	Sample_5	3.28e+005	N/A	N/A		☐
9	Sample_6	1.06e+005	N/A	N/A		☐
10	Sample_7	3.77e+003	N/A	N/A		☐
11	Sample_8	5.26e+003	N/A	N/A		☐
12	Sample_9	0.00e+000	N/A	N/A		☐
13	Sample_10	1.13e+004	N/A	N/A		☐
14	Sample_11	6.48e+003	N/A	N/A		☐
15	Sample_12	1.76e+004	N/A	N/A		☐
16	Sample_13	0.00e+000	N/A	N/A		☐
17	Sample_14	1.75e+004	N/A	N/A		☐
18	Sample_15	1.14e+005	N/A	N/A		☐
19	Sample_16	1.21e+004	N/A	N/A		☐
20	Sample_17	2.63e+004	N/A	N/A		☐
21	Sample_18	9.23e+004	N/A	N/A		☐
22	Sample_19	2.51e+004	N/A	N/A		☐
23	Sample_20	7.37e+003	N/A	N/A		☐
24	Sample_21	0.00e+000	N/A	N/A		☐
25	Sample_22	0.00e+000	N/A	N/A		☐
26	Sample_23	0.00e+000	N/A	N/A		☐
27	Sample_24	0.00e+000	N/A	N/A		☐
28	Sample_25	2.33e+003	N/A	N/A		☐
29	Sample_3	4.20e+005	N/A	N/A		☐
30	Sample_2	9.65e+004	N/A	N/A		☐
31	08092025_Sample_1_TR1	2.16e+002	N/A	N/A		☑
32	08092025_Sample_1_TR2	1.81e+002	N/A	N/A		☑
33	08092025_Sample_1_TR3	2.27e+002	N/A	N/A		☑
34	08092025_Sample_2_TR1	2.11e+002	N/A	N/A		☑
35	08092025_Sample_2_TR2	8.00e+002	N/A	N/A		☑
36	08092025_Sample_2_TR3	9.24e+002	N/A	N/A		☑
37	08092025_Sample_3_TR1	2.78e+002	N/A	N/A		☑
38	08092025_Sample_3_TR2	2.76e+002	N/A	N/A		☑
39	08092025_Sample_3_TR3	7.40e+002	N/A	N/A		☑
40	08092025_Sample_4_TR1	5.94e+002	N/A	N/A		☑
41	08092025_Sample_4_TR2	1.85e+003	N/A	N/A		☑
42	08092025_Sample_4_TR3	2.27e+003	N/A	N/A		☑
43	08092025_Sample_5_TR1	1.53e+003	N/A	N/A		☐
44	08092025_Sample_5_TR2	2.73e+003	N/A	N/A		☑
45	08092025_Sample_5_TR3	1.98e+003	N/A	N/A		☑

	Sample Name	Analyte Peak Height (cps)	Analyte Concentration (ng/mL)	Standard Query Status	Use Record	Record Modified
46	08092025_Sample_6_TR1	3.79e+002	N/A	N/A		☑
47	08092025_Sample_6_TR2	1.34e+003	N/A	N/A		☑
48	08092025_Sample_6_TR3	6.03e+002	N/A	N/A		☑
49	08092025_Sample_7_TR1	3.58e+003	N/A	N/A		☑
50	08092025_Sample_7_TR2	2.77e+003	N/A	N/A		☑
51	08092025_Sample_7_TR3	3.09e+003	N/A	N/A		☑
52	08092025_Sample_8_TR1	2.94e+003	N/A	N/A		☑
53	08092025_Sample_8_TR2	3.51e+003	N/A	N/A		☑
54	08092025_Sample_8_TR3	4.07e+003	N/A	N/A		☑
55	08092025_Sample_9_TR1	1.26e+003	N/A	N/A		☑
56	08092025_Sample_9_TR2	8.10e+002	N/A	N/A		☑
57	08092025_Sample_9_TR3	1.07e+003	N/A	N/A		☑
58	08092025_Sample_10_TR1	3.00e+003	N/A	N/A		☑
59	08092025_Sample_10_TR2	3.08e+003	N/A	N/A		☑
60	08092025_Sample_10_TR3	3.03e+003	N/A	N/A		☑
61	08092025_Sample_11_TR1	2.40e+003	N/A	N/A		☑
62	08092025_Sample_11_TR2	2.42e+003	N/A	N/A		☑
63	08092025_Sample_11_TR3	2.57e+003	N/A	N/A		☑
64	08092025_Sample_12_TR1	3.14e+003	N/A	N/A		☑
65	08092025_Sample_12_TR2	2.84e+003	N/A	N/A		☑
66	08092025_Sample_12_TR3	2.99e+003	N/A	N/A		☑
67	08092025_Sample_13_TR1	7.19e+002	N/A	N/A		☑
68	08092025_Sample_13_TR2	1.19e+003	N/A	N/A		☑
69	08092025_Sample_13_TR3	1.37e+003	N/A	N/A		☑
70	08092025_Sample_14_TR1	2.54e+003	N/A	N/A		☑
71	08092025_Sample_14_TR2	2.87e+003	N/A	N/A		☑
72	08092025_Sample_14_TR3	2.64e+003	N/A	N/A		☑
73	08092025_Sample_15_TR1	2.46e+003	N/A	N/A		☑
74	08092025_Sample_15_TR2	2.56e+003	N/A	N/A		☑
75	08092025_Sample_15_TR3	2.65e+003	N/A	N/A		☑
76	08092025_Sample_16_TR1	7.03e+002	N/A	N/A		☑
77	08092025_Sample_16_TR2	1.04e+003	N/A	N/A		☑
78	08092025_Sample_16_TR3	1.30e+003	N/A	N/A		☑
79	08092025_Sample_17_TR1	3.12e+002	N/A	N/A		☑
80	08092025_Sample_17_TR2	4.40e+002	N/A	N/A		☑
81	08092025_Sample_17_TR3	4.56e+002	N/A	N/A		☑
82	08092025_Sample_18_TR1	3.11e+003	N/A	N/A		☑
83	08092025_Sample_18_TR2	1.82e+003	N/A	N/A		☑
84	08092025_Sample_18_TR3	2.99e+003	N/A	N/A		☑
85	08092025_Sample_19_TR1	7.43e+002	N/A	N/A		☑
86	08092025_Sample_19_TR2	3.56e+003	N/A	N/A		☑
87	08092025_Sample_19_TR3	2.59e+003	N/A	N/A		☑
88	08092025_Sample_20_TR1	1.26e+004	N/A	N/A		☑
89	08092025_Sample_20_TR2	1.43e+004	N/A	N/A		☑
90	08092025_Sample_20_TR3	1.55e+004	N/A	N/A		☑

	Sample Name	Analyte Peak Height (cps)	Analyte Concentration (ng/mL)	Standard Query Status	Use Record	Record Modified
91	08092025_Sample_21_TR1	1.42e+004	N/A	N/A		☑
92	08092025_Sample_21_TR2	1.20e+004	N/A	N/A		☑
93	08092025_Sample_21_TR3	1.29e+004	N/A	N/A		☑
94	08092025_Sample_22_TR1	6.10e+003	N/A	N/A		☑
95	08092025_Sample_22_TR2	6.59e+003	N/A	N/A		☑
96	08092025_Sample_22_TR3	6.27e+003	N/A	N/A		☑
97	08092025_Sample_23_TR1	9.15e+003	N/A	N/A		☐
98	08092025_Sample_23_TR2	8.14e+003	N/A	N/A		☑
99	08092025_Sample_23_TR3	8.91e+003	N/A	N/A		☑
100	08092025_Sample_24_TR1	1.36e+004	N/A	N/A		☐
101	08092025_Sample_24_TR2	1.46e+004	N/A	N/A		☐
102	08092025_Sample_24_TR3	1.27e+004	N/A	N/A		☑
103	08092025_Sample_25_TR1	8.56e+002	N/A	N/A		☑
104	08092025_Sample_25_TR2	6.76e+002	N/A	N/A		☑
105	08092025_Sample_25_TR3	1.00e+003	N/A	N/A		☑
106	08092025_Sample_26_TR1	1.93e+002	N/A	N/A		☑
107	08092025_Sample_26_TR2	2.53e+002	N/A	N/A		☐
108	08092025_Sample_26_TR3	1.57e+002	N/A	N/A		☑
109	08092025_Sample_27_TR1	9.32e+001	N/A	N/A		☑
110	08092025_Sample_27_TR2	9.45e+001	N/A	N/A		☑
111	08092025_Sample_27_TR3	8.68e+001	N/A	N/A		☑
112	08092025_Sample_28_TR1	2.94e+002	N/A	N/A		☑
113	08092025_Sample_28_TR2	1.58e+002	N/A	N/A		☑
114	08092025_Sample_28_TR3	3.04e+002	N/A	N/A		☑
115	08092025_Sample_29_TR1	9.08e+003	N/A	N/A		☐
116	08092025_Sample_29_TR2	9.16e+003	N/A	N/A		☐
117	08092025_Sample_29_TR3	6.62e+003	N/A	N/A		☑
118	08092025_Sample_30_TR1	7.35e+003	N/A	N/A		☑
119	08092025_Sample_30_TR2	4.80e+003	N/A	N/A		☑
120	08092025_Sample_30_TR3	4.90e+003	N/A	N/A		☑
121	08092025_Sample_31_TR1	2.36e+001	N/A	N/A		☑
122	08092025_Sample_31_TR2	2.50e+001	N/A	N/A		☑
123	08092025_Sample_31_TR3	2.45e+001	N/A	N/A		☑
124	08092025_Sample_32_TR1	2.88e+001	N/A	N/A		☑
125	08092025_Sample_32_TR2	3.10e+001	N/A	N/A		☑
126	08092025_Sample_32_TR3	2.38e+001	N/A	N/A		☑
127	08092025_Sample_33_TR1	6.69e+001	N/A	N/A		☑
128	08092025_Sample_33_TR2	4.98e+001	N/A	N/A		☑
129	08092025_Sample_33_TR3	8.27e+001	N/A	N/A		☐
130	08092025_Sample_34_TR1	1.74e+001	N/A	N/A		☑
131	08092025_Sample_34_TR2	1.71e+001	N/A	N/A		☑
132	08092025_Sample_34_TR3	2.06e+001	N/A	N/A		☑
133	08092025_Sample_35_TR1	6.25e+002	N/A	N/A		☐
134	08092025_Sample_35_TR2	7.68e+002	N/A	N/A		☑
135	08092025_Sample_35_TR3	1.32e+003	N/A	N/A		☑

	Sample Name	Analyte Peak Height (cps)	Analyte Concentration (ng/mL)	Standard Query Status	Use Record	Record Modified
136	Jayram_09092025_1	0.00e+000	N/A	N/A		☐
137	Jayram_09092025_10	7.13e+001	N/A	N/A		☐
138	Jayram_09092025_11	8.53e+001	N/A	N/A		☐
139	Jayram_09092025_12	1.05e+002	N/A	N/A		☐
140	Jayram_09092025_13	1.46e+002	N/A	N/A		☐
141	Jayram_09092025_15	0.00e+000	N/A	N/A		☐
142	Jayram_09092025_16	0.00e+000	N/A	N/A		☐
143	Jayram_09092025_17	0.00e+000	N/A	N/A		☐
144	Jayram_09092025_18	1.14e+002	N/A	N/A		☐
145	Jayram_09092025_19	0.00e+000	N/A	N/A		☐
146	Jayram_09092025_2	0.00e+000	N/A	N/A		☐
147	Jayram_09092025_21	2.14e+002	N/A	N/A		☐
148	Jayram_09092025_22	6.33e+001	N/A	N/A		☐
149	Jayram_09092025_23	0.00e+000	N/A	N/A		☐
150	Jayram_09092025_24	0.00e+000	N/A	N/A		☐
151	Jayram_09092025_26	2.94e+003	N/A	N/A		☐
152	Jayram_09092025_27	0.00e+000	N/A	N/A		☐
153	Jayram_09092025_28	0.00e+000	N/A	N/A		☐
154	Jayram_09092025_29	0.00e+000	N/A	N/A		☐
155	Jayram_09092025_3	0.00e+000	N/A	N/A		☐
156	Jayram_09092025_30	0.00e+000	N/A	N/A		☐
157	Jayram_09092025_31	0.00e+000	N/A	N/A		☐
158	Jayram_09092025_32	0.00e+000	N/A	N/A		☐
159	Jayram_09092025_33	5.97e+001	N/A	N/A		☐
160	Jayram_09092025_35	1.01e+002	N/A	N/A		☐
161	Jayram_09092025_36	0.00e+000	N/A	N/A		☐
162	Jayram_09092025_37	0.00e+000	N/A	N/A		☐
163	Jayram_09092025_38	0.00e+000	N/A	N/A		☐
164	Jayram_09092025_39	0.00e+000	N/A	N/A		☐
165	Jayram_09092025_4	1.28e+002	N/A	N/A		☐
166	Jayram_09092025_5	0.00e+000	N/A	N/A		☐
167	Jayram_09092025_6	0.00e+000	N/A	N/A		☐
168	Jayram_09092025_7	0.00e+000	N/A	N/A		☐
169	Jayram_09092025_8	1.16e+002	N/A	N/A		☐
170	Jayram_09092025_9	1.16e+002	N/A	N/A		☐

	Sample Name	Calculated Concentration (ng/mL)	Accuracy (%)
1	Std_4_Dil_1	253.	101.
2	Std_4_Dil_2	116.	92.5
3	Std_4_Dil_3	67.0	107.
4	Std_4_Dil_4	35.8	115.
5	Std_4_Dil_5	12.8	81.8
6	Sample_1	4090.	N/A
7	Sample_4	39600.	N/A
8	Sample_5	16900.	N/A
9	Sample_6	7370.	N/A
10	Sample_7	184.	N/A
11	Sample_8	229.	N/A
12	Sample_9	No Peak	N/A
13	Sample_10	500.	N/A
14	Sample_11	326.	N/A
15	Sample_12	1080.	N/A
16	Sample_13	No Peak	N/A
17	Sample_14	1210.	N/A
18	Sample_15	7420.	N/A
19	Sample_16	598.	N/A
20	Sample_17	1200.	N/A
21	Sample_18	5280.	N/A
22	Sample_19	1750.	N/A
23	Sample_20	326.	N/A
24	Sample_21	No Peak	N/A
25	Sample_22	No Peak	N/A
26	Sample_23	No Peak	N/A
27	Sample_24	No Peak	N/A
28	Sample_25	131.	N/A
29	Sample_3	22500.	N/A
30	Sample_2	5900.	N/A
31	08092025_Sample_1_TR1 NR Control	17.1	N/A
32	08092025_Sample_1_TR2	16.6	N/A
33	08092025_Sample_1_TR3	17.6	N/A
34	08092025_Sample_2_TR1 MA2L2	14.9	N/A
35	08092025_Sample_2_TR2	17.1	N/A
36	08092025_Sample_2_TR3	14.3	N/A
37	08092025_Sample_3_TR1 MA2L4	32.9	N/A
38	08092025_Sample_3_TR2	23.5	N/A
39	08092025_Sample_3_TR3	24.6	N/A
40	08092025_Sample_4_TR1 MA2L12	34.9	N/A
41	08092025_Sample_4_TR2	14.6	N/A
42	08092025_Sample_4_TR3	13.2	N/A
43	08092025_Sample_5_TR1 MA2L5	158.	N/A
44	08092025_Sample_5_TR2	22.9	N/A
45	08092025_Sample_5_TR3	18.2	N/A

	Sample Name	Calculated Concentration (ng/mL)	Accuracy (%)
46	08092025_Sample_6_TR1 MA2+TT8l[illegible]	24.8	N/A
47	08092025_Sample_6_TR2	15.9	N/A
48	08092025_Sample_6_TR3	33.8	N/A
49	08092025_Sample_7_TR1 MA2+TT8l[illegible]	192.	N/A
50	08092025_Sample_7_TR2	278.	N/A
51	08092025_Sample_7_TR3	276.	N/A
52	08092025_Sample_8_TR1 MA2+TT8l[illegible]	484.	N/A
53	08092025_Sample_8_TR2	505.	N/A
54	08092025_Sample_8_TR3	579.	N/A
55	08092025_Sample_9_TR1 MA4l1	109.	N/A
56	08092025_Sample_9_TR2	102.	N/A
57	08092025_Sample_9_TR3	108.	N/A
58	08092025_Sample_10_TR1 MA4L6	440.	N/A
59	08092025_Sample_10_TR2	450.	N/A
60	08092025_Sample_10_TR3	387.	N/A
61	08092025_Sample_11_TR1 MA4L7	212.	N/A
62	08092025_Sample_11_TR2	368.	N/A
63	08092025_Sample_11_TR3	399.	N/A
64	08092025_Sample_12_TR1 MA4+TT8L3	428.	N/A
65	08092025_Sample_12_TR2	369.	N/A
66	08092025_Sample_12_TR3	326.	N/A
67	08092025_Sample_13_TR1 MA4+TT8L4	102.	N/A
68	08092025_Sample_13_TR2	154.	N/A
69	08092025_Sample_13_TR3	146.	N/A
70	08092025_Sample_14_TR1 MA4+TT8L17	315.	N/A
71	08092025_Sample_14_TR2	323.	N/A
72	08092025_Sample_14_TR3	335.	N/A
73	08092025_Sample_15_TR1 MA4+TT8L18	284.	N/A
74	08092025_Sample_15_TR2	382.	N/A
75	08092025_Sample_15_TR3	255.	N/A
76	08092025_Sample_16_TR1 MA4+TT8L1	118.	N/A
77	08092025_Sample_16_TR2	144.	N/A
78	08092025_Sample_16_TR3	132.	N/A
79	08092025_Sample_17_TR1 MA8L2	36.9	N/A
80	08092025_Sample_17_TR2	98.7	N/A
81	08092025_Sample_17_TR3	64.8	N/A
82	08092025_Sample_18_TR1 MA8L6	151.	N/A
83	08092025_Sample_18_TR2	139.	N/A
84	08092025_Sample_18_TR3	130.	N/A
85	08092025_Sample_19_TR1 MA8L8	107.	N/A
86	08092025_Sample_19_TR2	182.	N/A
87	08092025_Sample_19_TR3	123.	N/A
88	08092025_Sample_20_TR1 MA8L3	1250.	N/A
89	08092025_Sample_20_TR2	1170.	N/A
90	08092025_Sample_20_TR3	1330.	N/A

	Sample Name	Calculated Concentration (ng/mL)	Accuracy (%)
91	08092025_Sample_21_TR1 MA8+TT8L3	1280.	N/A
92	08092025_Sample_21_TR2	1370.	N/A
93	08092025_Sample_21_TR3	1120.	N/A
94	08092025_Sample_22_TR1 MA8+TT8L1	374.	N/A
95	08092025_Sample_22_TR2	437.	N/A
96	08092025_Sample_22_TR3	554.	N/A
97	08092025_Sample_23_TR1 MA8+TT8L5	691.	N/A
98	08092025_Sample_23_TR2	563.	N/A
99	08092025_Sample_23_TR3	612.	N/A
100	08092025_Sample_24_TR1 MA8+TT8L3	1270.	N/A
101	08092025_Sample_24_TR2	1200.	N/A
102	08092025_Sample_24_TR3	1210.	N/A
103	08092025_Sample_25_TR1	80.3	N/A
104	08092025_Sample_25_TR2	78.0	N/A
105	08092025_Sample_25_TR3	66.2	N/A
106	08092025_Sample_26_TR1	17.1	N/A
107	08092025_Sample_26_TR2	17.5	N/A
108	08092025_Sample_26_TR3	20.5	N/A
109	08092025_Sample_27_TR1	17.1	N/A
110	08092025_Sample_27_TR2	14.6	N/A
111	08092025_Sample_27_TR3	13.4	N/A
112	08092025_Sample_28_TR1	21.2	N/A
113	08092025_Sample_28_TR2	16.2	N/A
114	08092025_Sample_28_TR3	19.2	N/A
115	08092025_Sample_29_TR1 MA4L13	789.	N/A
116	08092025_Sample_29_TR2	906.	N/A
117	08092025_Sample_29_TR3	967.	N/A
118	08092025_Sample_30_TR1	665.	N/A
119	08092025_Sample_30_TR2	651.	N/A
120	08092025_Sample_30_TR3	567.	N/A
121	08092025_Sample_31_TR1	8.21	N/A
122	08092025_Sample_31_TR2	6.47	N/A
123	08092025_Sample_31_TR3	6.45	N/A
124	08092025_Sample_32_TR1	6.73	N/A
125	08092025_Sample_32_TR2	7.09	N/A
126	08092025_Sample_32_TR3	7.27	N/A
127	08092025_Sample_33_TR1	10.4	N/A
128	08092025_Sample_33_TR2	10.5	N/A
129	08092025_Sample_33_TR3	11.4	N/A
130	08092025_Sample_34_TR1	4.99	N/A
131	08092025_Sample_34_TR2	5.48	N/A
132	08092025_Sample_34_TR3	5.77	N/A
133	08092025_Sample_35_TR1	56.3	N/A
134	08092025_Sample_35_TR2	69.1	N/A
135	08092025_Sample_35_TR3	78.1	N/A

	Sample Name	Calculated Concentration (ng/mL)	Accuracy (%)
136	Jayram_09092025_1	No Peak	N/A
137	Jayram_09092025_10	7.81	N/A
138	Jayram_09092025_11	17.1	N/A
139	Jayram_09092025_12	23.6	N/A
140	Jayram_09092025_13	20.0	N/A
141	Jayram_09092025_15	No Peak	N/A
142	Jayram_09092025_16	No Peak	N/A
143	Jayram_09092025_17	No Peak	N/A
144	Jayram_09092025_18	12.6	N/A
145	Jayram_09092025_19	No Peak	N/A
146	Jayram_09092025_2	No Peak	N/A
147	Jayram_09092025_21	21.4	N/A
148	Jayram_09092025_22	9.72	N/A
149	Jayram_09092025_23	No Peak	N/A
150	Jayram_09092025_24	No Peak	N/A
151	Jayram_09092025_26	428.	N/A
152	Jayram_09092025_27	No Peak	N/A
153	Jayram_09092025_28	No Peak	N/A
154	Jayram_09092025_29	No Peak	N/A
155	Jayram_09092025_3	No Peak	N/A
156	Jayram_09092025_30	No Peak	N/A
157	Jayram_09092025_31	No Peak	N/A
158	Jayram_09092025_32	No Peak	N/A
159	Jayram_09092025_33	17.7	N/A
160	Jayram_09092025_35	16.2	N/A
161	Jayram_09092025_36	No Peak	N/A
162	Jayram_09092025_37	No Peak	N/A
163	Jayram_09092025_38	No Peak	N/A
164	Jayram_09092025_39	No Peak	N/A
165	Jayram_09092025_4	23.3	N/A
166	Jayram_09092025_5	No Peak	N/A
167	Jayram_09092025_6	No Peak	N/A
168	Jayram_09092025_7	No Peak	N/A
169	Jayram_09092025_8	9.84	N/A
170	Jayram_09092025_9	23.1	N/A

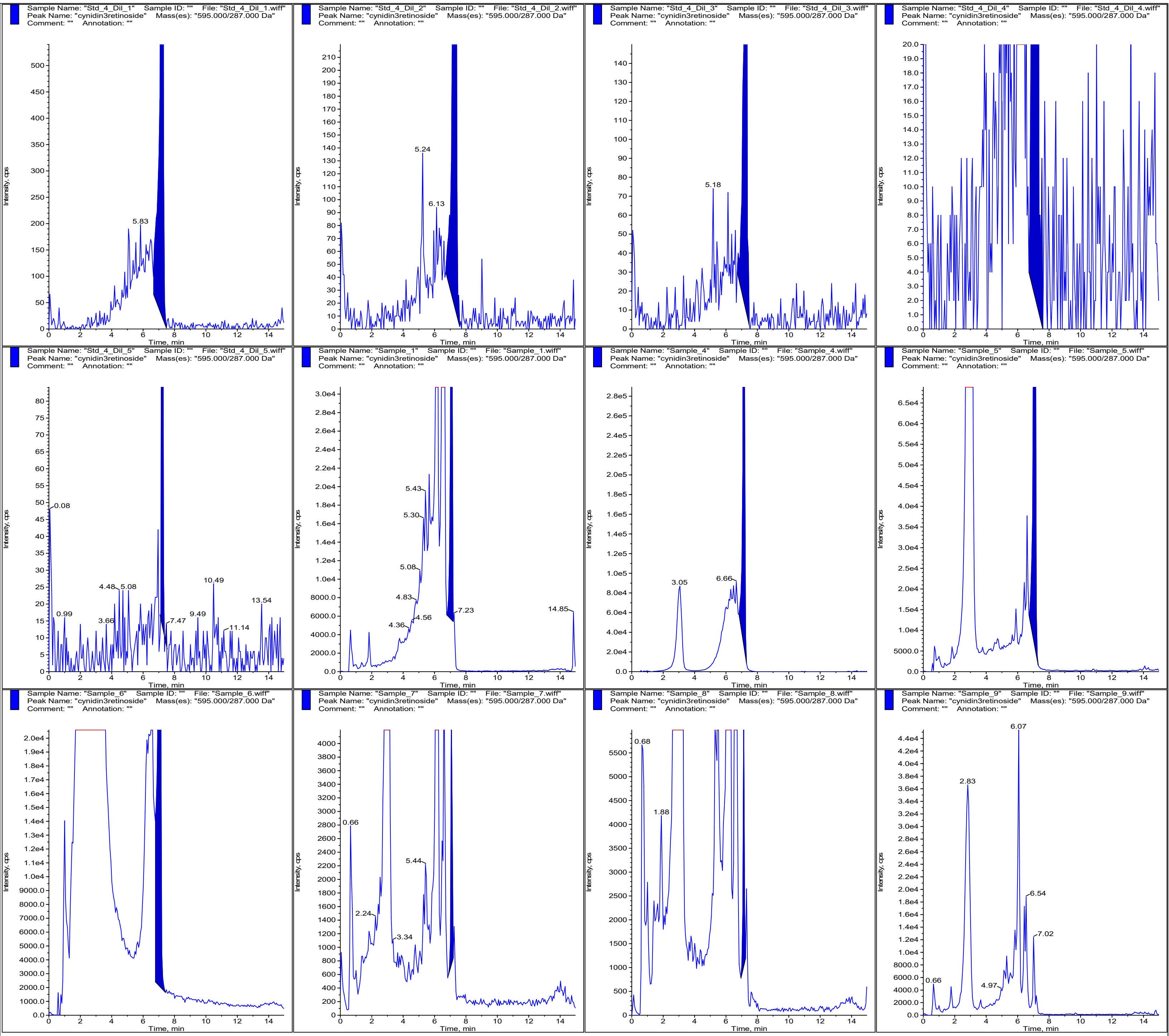

	Sample Name	Sample ID	Sample Type	File Name	Analyte Peak Area (counts)
1	Std_4_Dil_1		Standard	28072025_Akhil_Dr	3.03e+004
2	Std_4_Dil_2		Standard	28072025_Akhil_Dr	1.36e+004
3	Std_4_Dil_3		Standard	28072025_Akhil_Dr	7.74e+003
4	Std_4_Dil_4		Standard	28072025_Akhil_Dr	3.98e+003
5	Std_4_Dil_5		Standard	28072025_Akhil_Dr	1.19e+003
6	Sample_1		Unknown	28072025_Akhil_Dr	4.94e+005
7	Sample_4		Unknown	28072025_Akhil_Dr	4.78e+006

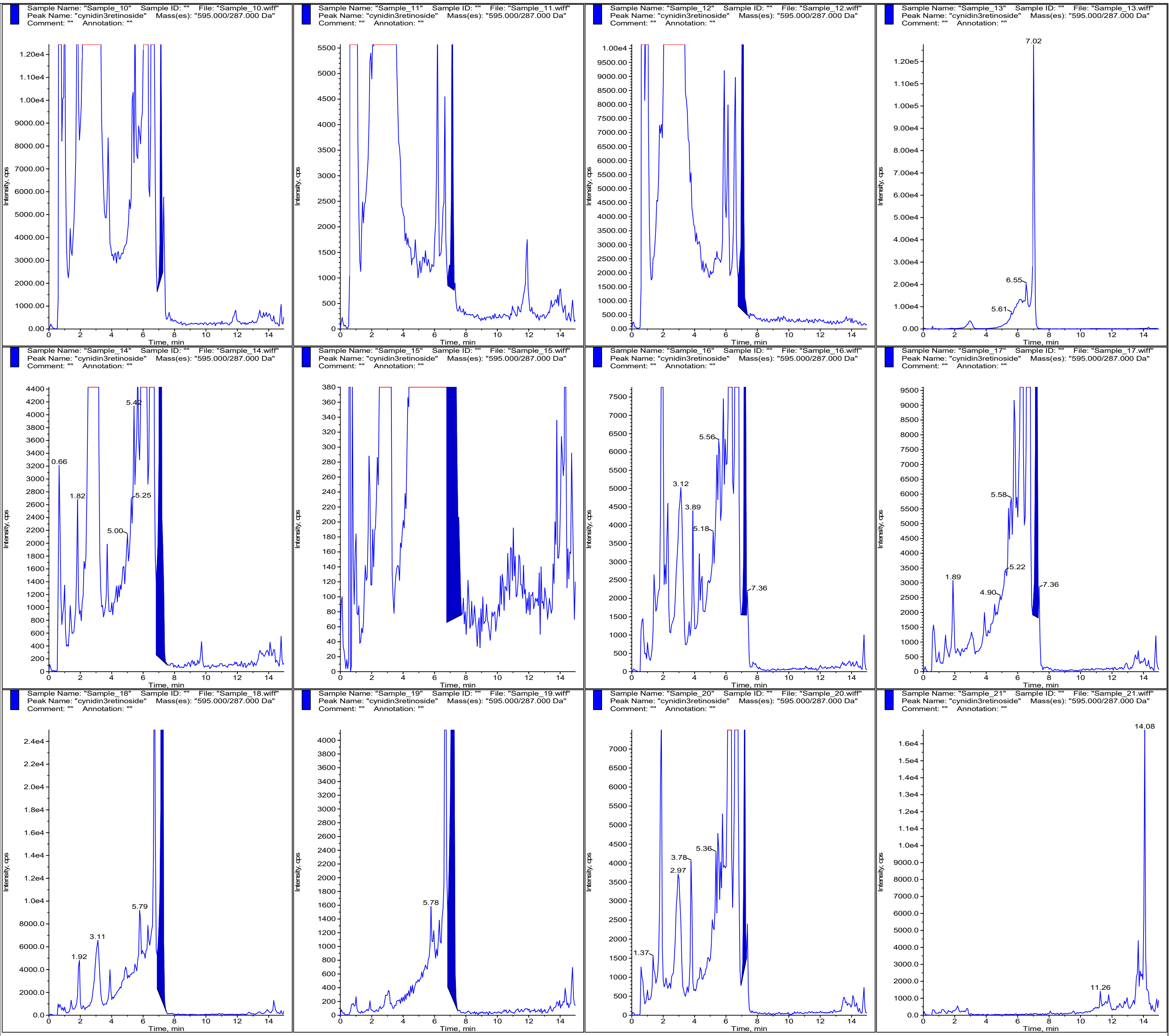

	Sample Name	Sample ID	Sample Type	File Name	Analyte Peak Area (counts)
13	Sample_10		Unknown	28072025_Akhil_Dr	6.01e+004
14	Sample_11		Unknown	28072025_Akhil_Dr	3.90e+004
15	Sample_12		Unknown	28072025_Akhil_Dr	1.31e+005
16	Sample_13		Unknown	28072025_Akhil_Dr	0.00e+000
17	Sample_14		Unknown	28072025_Akhil_Dr	1.45e+005
18	Sample_15		Unknown	28072025_Akhil_Dr	8.97e+005
19	Sample_16		Unknown	28072025_Akhil_Dr	7.19e+004

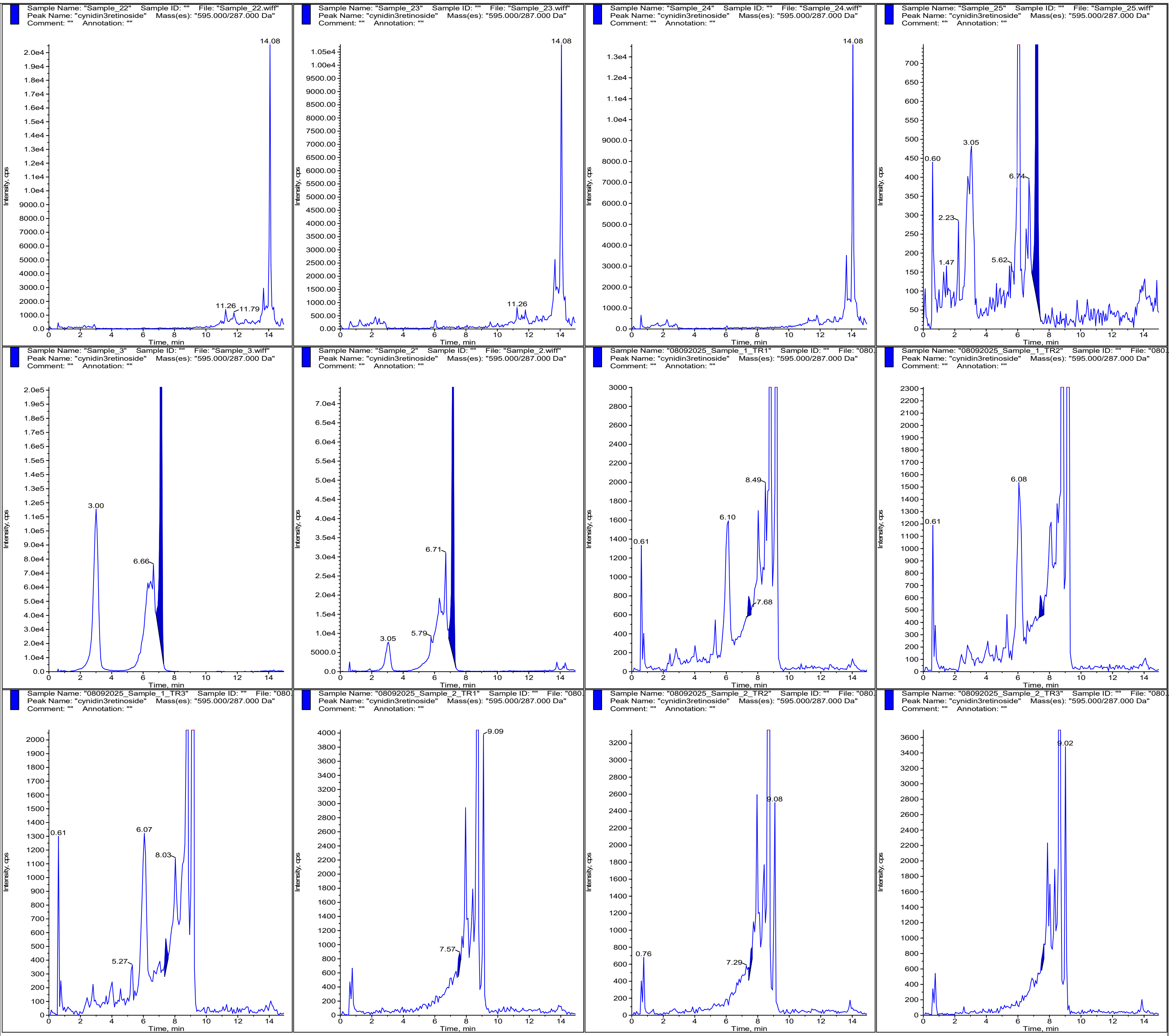

	Sample Name	Sample ID	Sample Type	File Name	Analyte Peak Area (counts)
25	Sample_22		Unknown	28072025_Akhil_Dr	0.00e+000
26	Sample_23		Unknown	28072025_Akhil_Dr	0.00e+000
27	Sample_24		Unknown	28072025_Akhil_Dr	0.00e+000
28	Sample_25		Unknown	28072025_Akhil_Dr	1.54e+004
29	Sample_3		Unknown	28072025_Akhil_Dr	2.72e+006
30	Sample_2		Unknown	28072025_Akhil_Dr	7.13e+005
31	08092025_Sample_1_TR1		Unknown	28072025_Akhil_Dr	1.72e+003

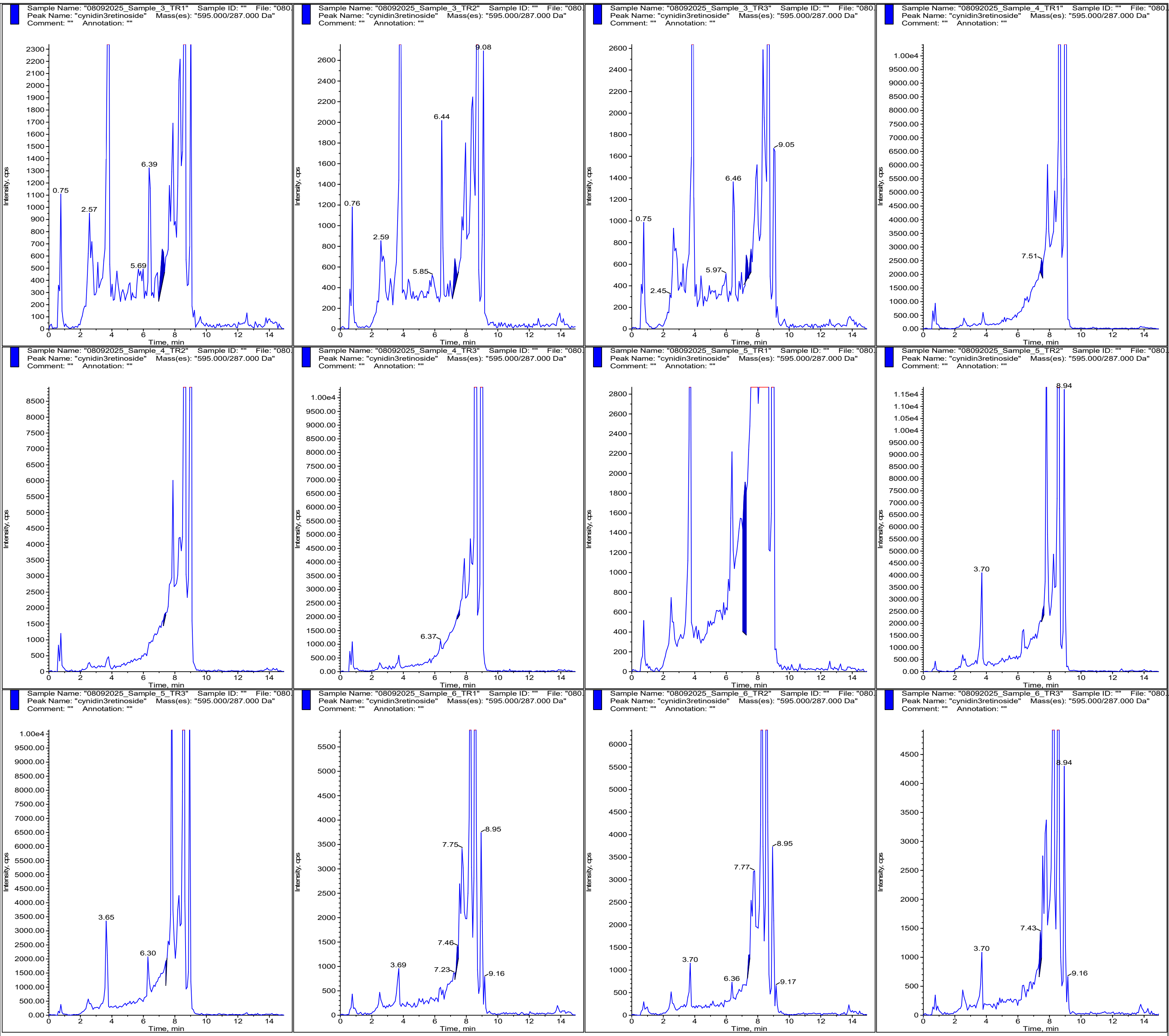

	Sample Name	Sample ID	Sample Type	File Name	Analyte Peak Area (counts)
37	08092025_Sample_3_TR1		Unknown	28072025_Akhil_Dr	3.62e+003
38	08092025_Sample_3_TR2		Unknown	28072025_Akhil_Dr	2.49e+003
39	08092025_Sample_3_TR3		Unknown	28072025_Akhil_Dr	2.62e+003
40	08092025_Sample_4_TR1		Unknown	28072025_Akhil_Dr	3.87e+003
41	08092025_Sample_4_TR2		Unknown	28072025_Akhil_Dr	1.41e+003
42	08092025_Sample_4_TR3		Unknown	28072025_Akhil_Dr	1.24e+003
43	08092025_Sample_5_TR1		Unknown	28072025_Akhil_Dr	1.88e+004

Acq. File: 28072025_Akhil_DrNegi_Pos_Stds.dam,..

Sample Name: Std_4_Dil_1
Sample Number: Sample 1 of 170

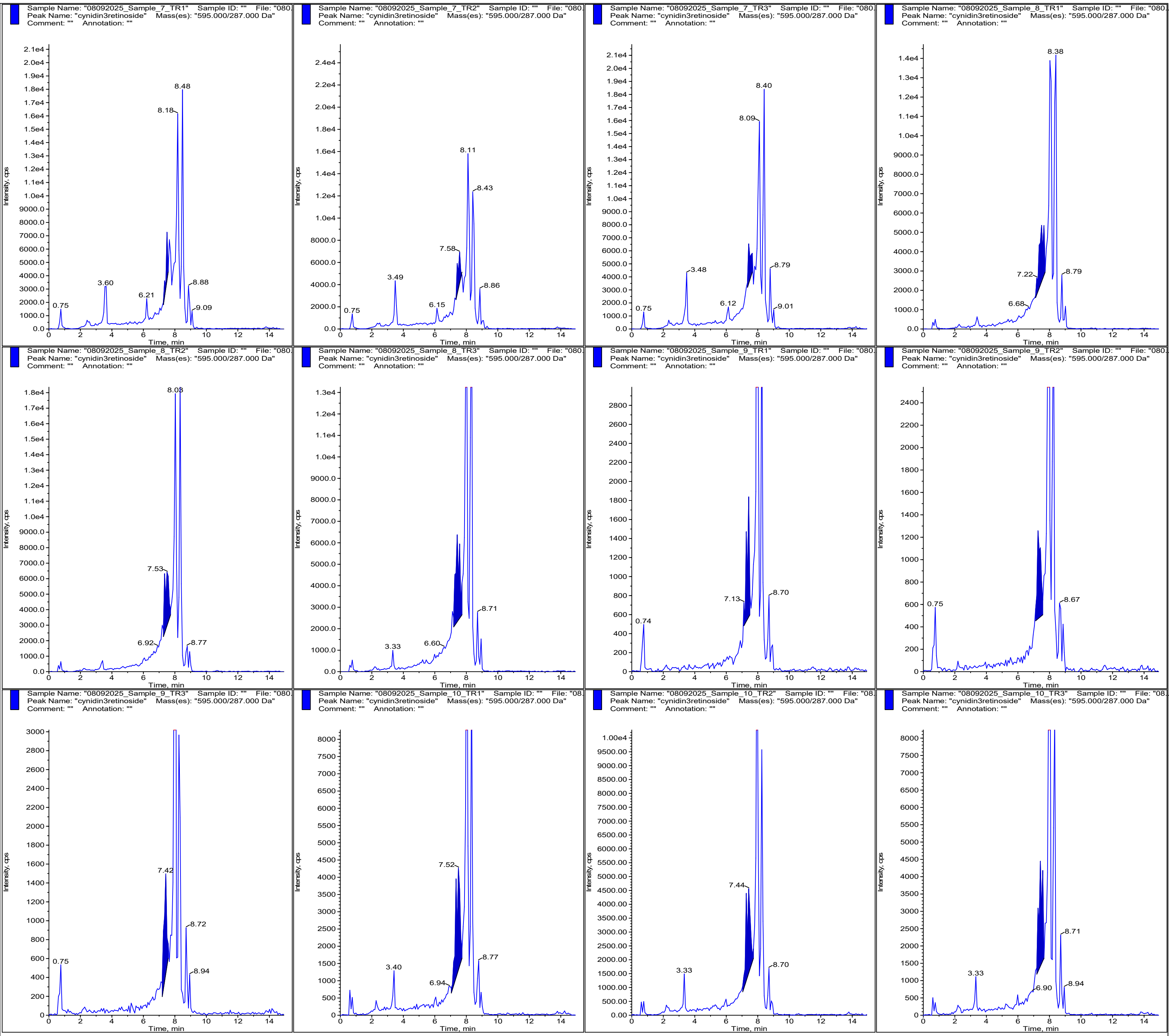

	Sample Name	Sample ID	Sample Type	File Name	Analyte Peak Area (counts)
49	08092025_Sample_7_TR1		Unknown	28072025_Akhil_Dr	2.28e+004
50	08092025_Sample_7_TR2		Unknown	28072025_Akhil_Dr	3.33e+004
51	08092025_Sample_7_TR3		Unknown	28072025_Akhil_Dr	3.31e+004
52	08092025_Sample_8_TR1		Unknown	28072025_Akhil_Dr	5.81e+004
53	08092025_Sample_8_TR2		Unknown	28072025_Akhil_Dr	6.07e+004
54	08092025_Sample_8_TR3		Unknown	28072025_Akhil_Dr	6.96e+004
55	08092025_Sample_9_TR1		Unknown	28072025_Akhil_Dr	1.28e+004

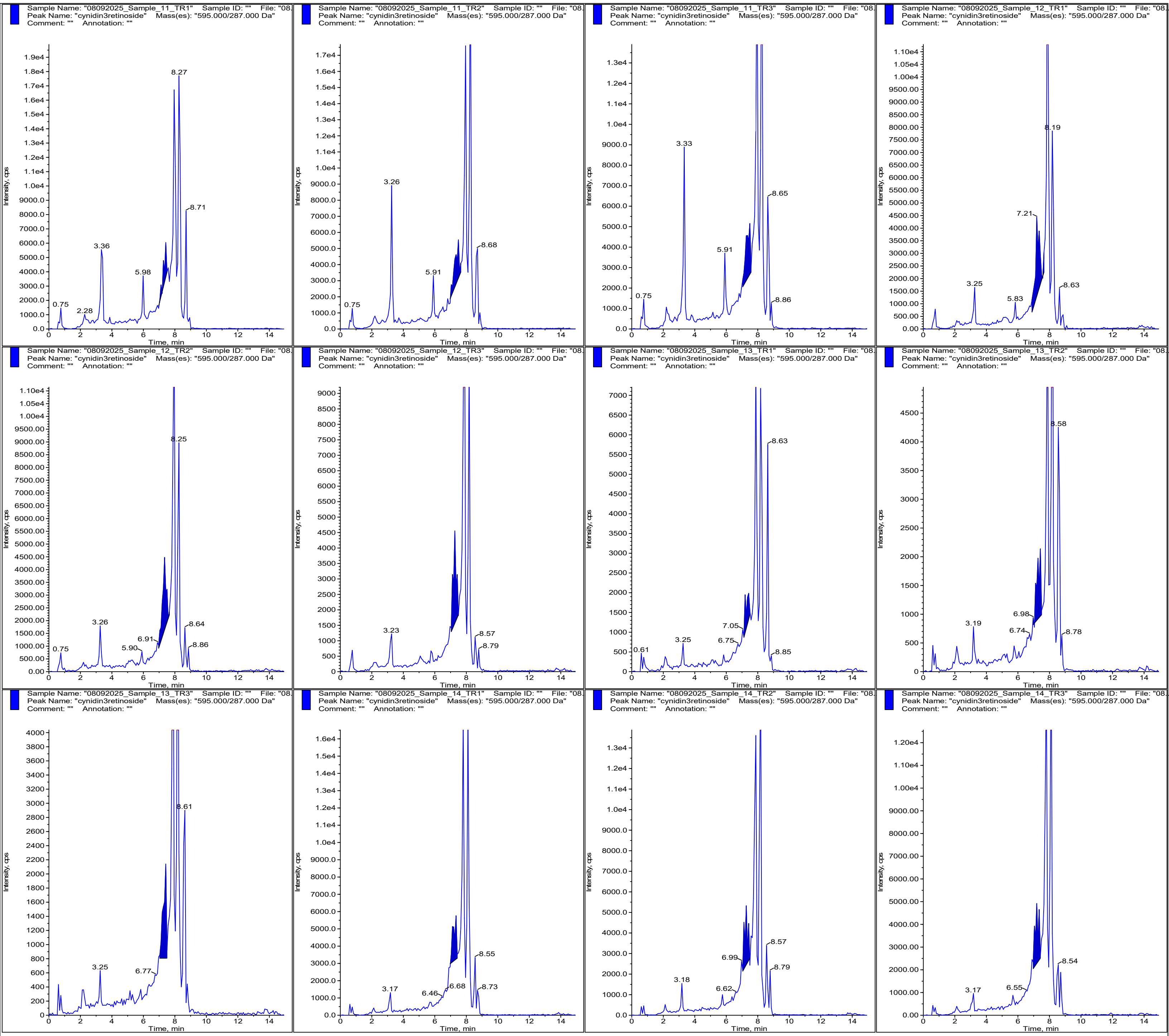

	Sample Name	Sample ID	Sample Type	File Name	Analyte Peak Area (counts)
61	08092025_Sample_11_TR1		Unknown	28072025_Akhil_Dr	2.52e+004
62	08092025_Sample_11_TR2		Unknown	28072025_Akhil_Dr	4.41e+004
63	08092025_Sample_11_TR3		Unknown	28072025_Akhil_Dr	4.79e+004
64	08092025_Sample_12_TR1		Unknown	28072025_Akhil_Dr	5.13e+004
65	08092025_Sample_12_TR2		Unknown	28072025_Akhil_Dr	4.42e+004
66	08092025_Sample_12_TR3		Unknown	28072025_Akhil_Dr	3.91e+004
67	08092025_Sample_13_TR1		Unknown	28072025_Akhil_Dr	1.20e+004

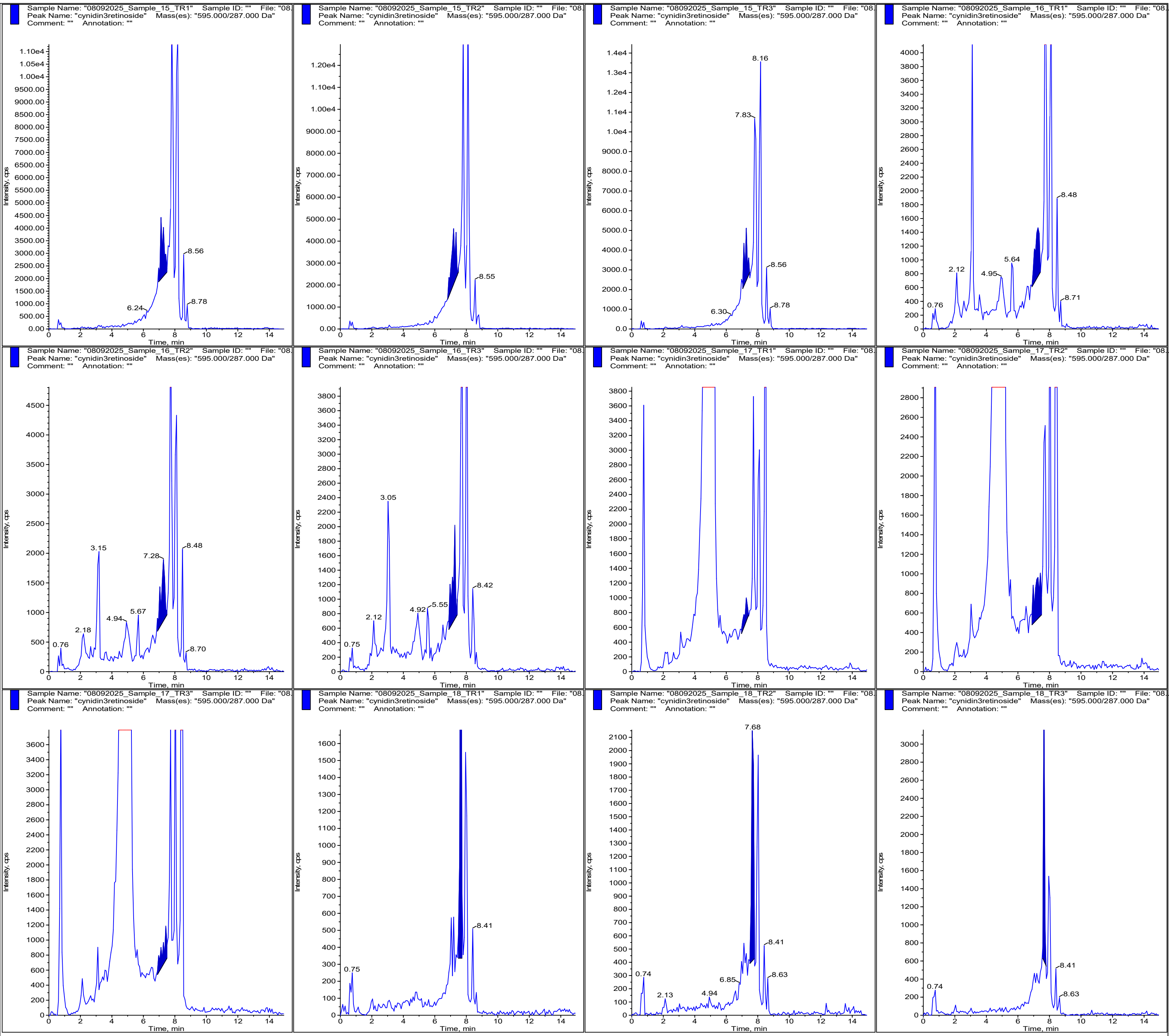

	Sample Name	Sample ID	Sample Type	File Name	Analyte Peak Area (counts)
73	08092025_Sample_15_TR1		Unknown	28072025_Akhil_Dr	3.40e+004
74	08092025_Sample_15_TR2		Unknown	28072025_Akhil_Dr	4.59e+004
75	08092025_Sample_15_TR3		Unknown	28072025_Akhil_Dr	3.05e+004
76	08092025_Sample_16_TR1		Unknown	28072025_Akhil_Dr	1.40e+004
77	08092025_Sample_16_TR2		Unknown	28072025_Akhil_Dr	1.71e+004
78	08092025_Sample_16_TR3		Unknown	28072025_Akhil_Dr	1.56e+004
79	08092025_Sample_17_TR1		Unknown	28072025_Akhil_Dr	4.11e+003

Acq. File:
28072025_Akhil_DrNegi_Pos_Stds.dam,..

Sample Name: Std_4_Dil_1
Sample Number: Sample 1 of 170

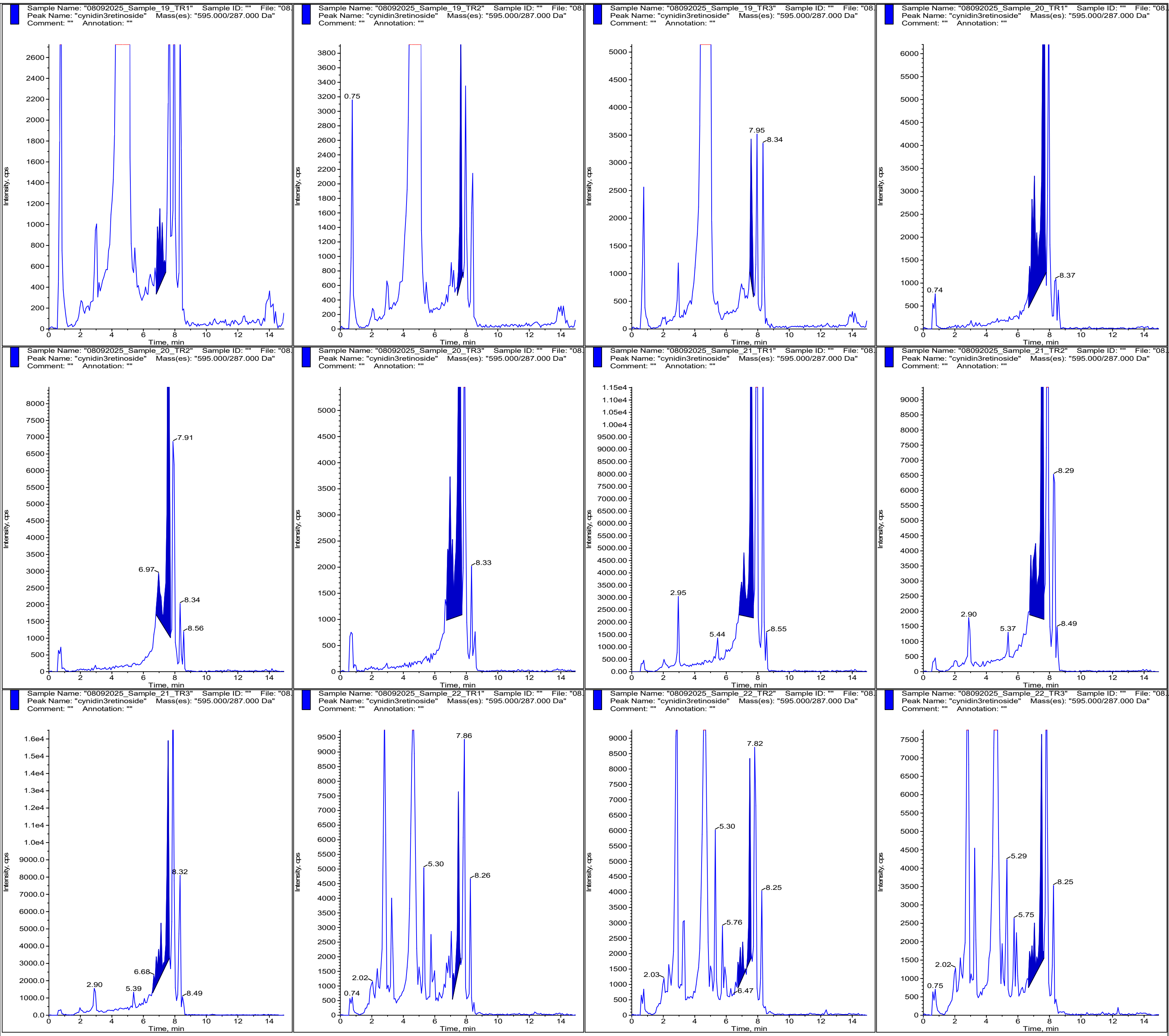

	Sample Name	Sample ID	Sample Type	File Name	Analyte Peak Area (counts)
85	08092025_Sample_19_TR1		Unknown	28072025_Akhil_Dr	1.26e+004
86	08092025_Sample_19_TR2		Unknown	28072025_Akhil_Dr	2.16e+004
87	08092025_Sample_19_TR3		Unknown	28072025_Akhil_Dr	1.45e+004
88	08092025_Sample_20_TR1		Unknown	28072025_Akhil_Dr	1.51e+005
89	08092025_Sample_20_TR2		Unknown	28072025_Akhil_Dr	1.41e+005
90	08092025_Sample_20_TR3		Unknown	28072025_Akhil_Dr	1.61e+005
91	08092025_Sample_21_TR1		Unknown	28072025_Akhil_Dr	1.55e+005

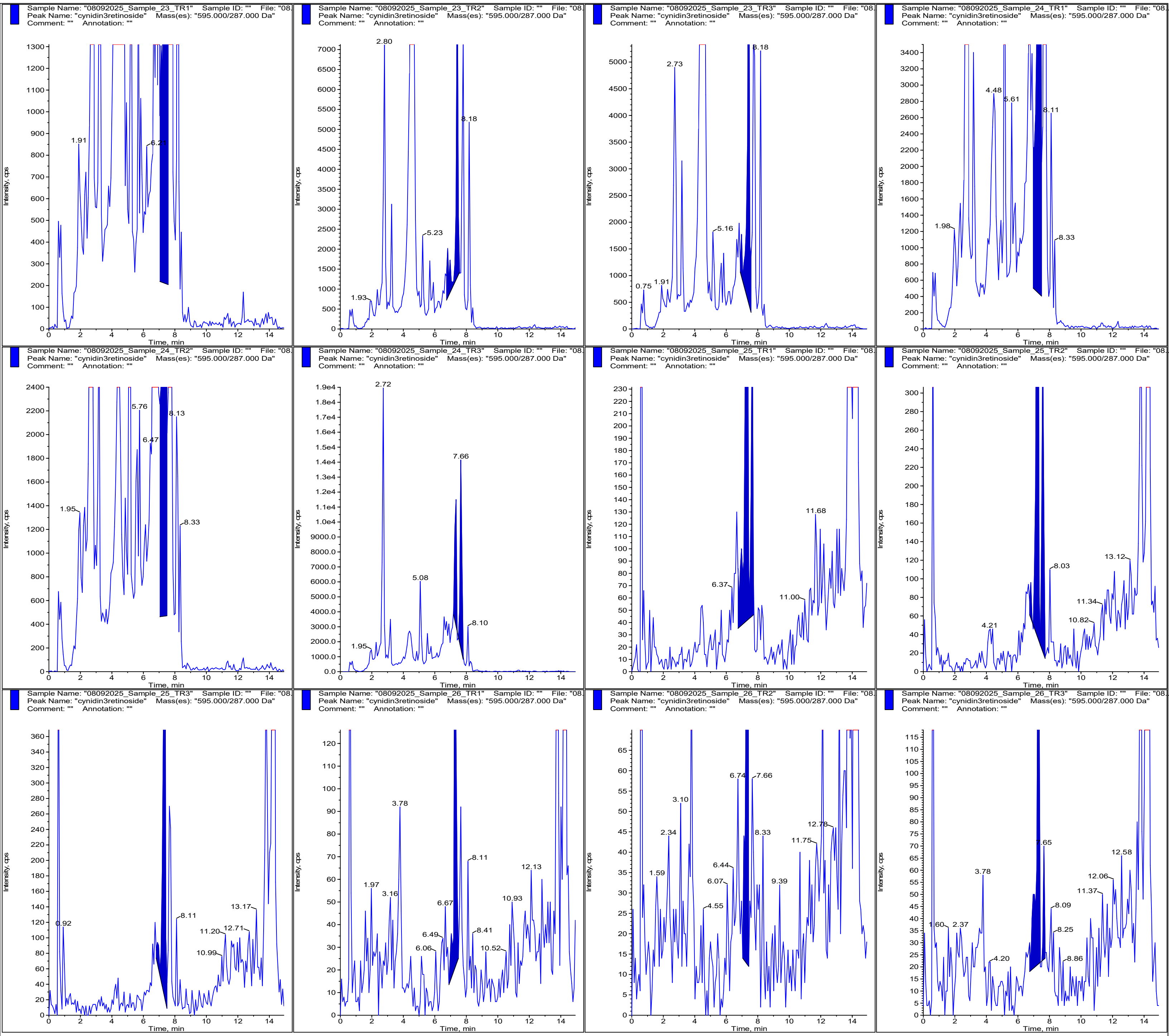

	Sample Name	Sample ID	Sample Type	File Name	Analyte Peak Area (counts)
97	08092025_Sample_23_TR1		Unknown	28072025_Akhil_Dr	8.32e+004
98	08092025_Sample_23_TR2		Unknown	28072025_Akhil_Dr	6.78e+004
99	08092025_Sample_23_TR3		Unknown	28072025_Akhil_Dr	7.37e+004
100	08092025_Sample_24_TR1		Unknown	28072025_Akhil_Dr	1.53e+005
101	08092025_Sample_24_TR2		Unknown	28072025_Akhil_Dr	1.45e+005
102	08092025_Sample_24_TR3		Unknown	28072025_Akhil_Dr	1.46e+005
103	08092025_Sample_25_TR1		Unknown	28072025_Akhil_Dr	9.35e+003

Acq. File: 28072025_Akhil_DrNegi_Pos_Stds.dam,..

Sample Name: Std_4_Dil_1
Sample Number: Sample 1 of 170

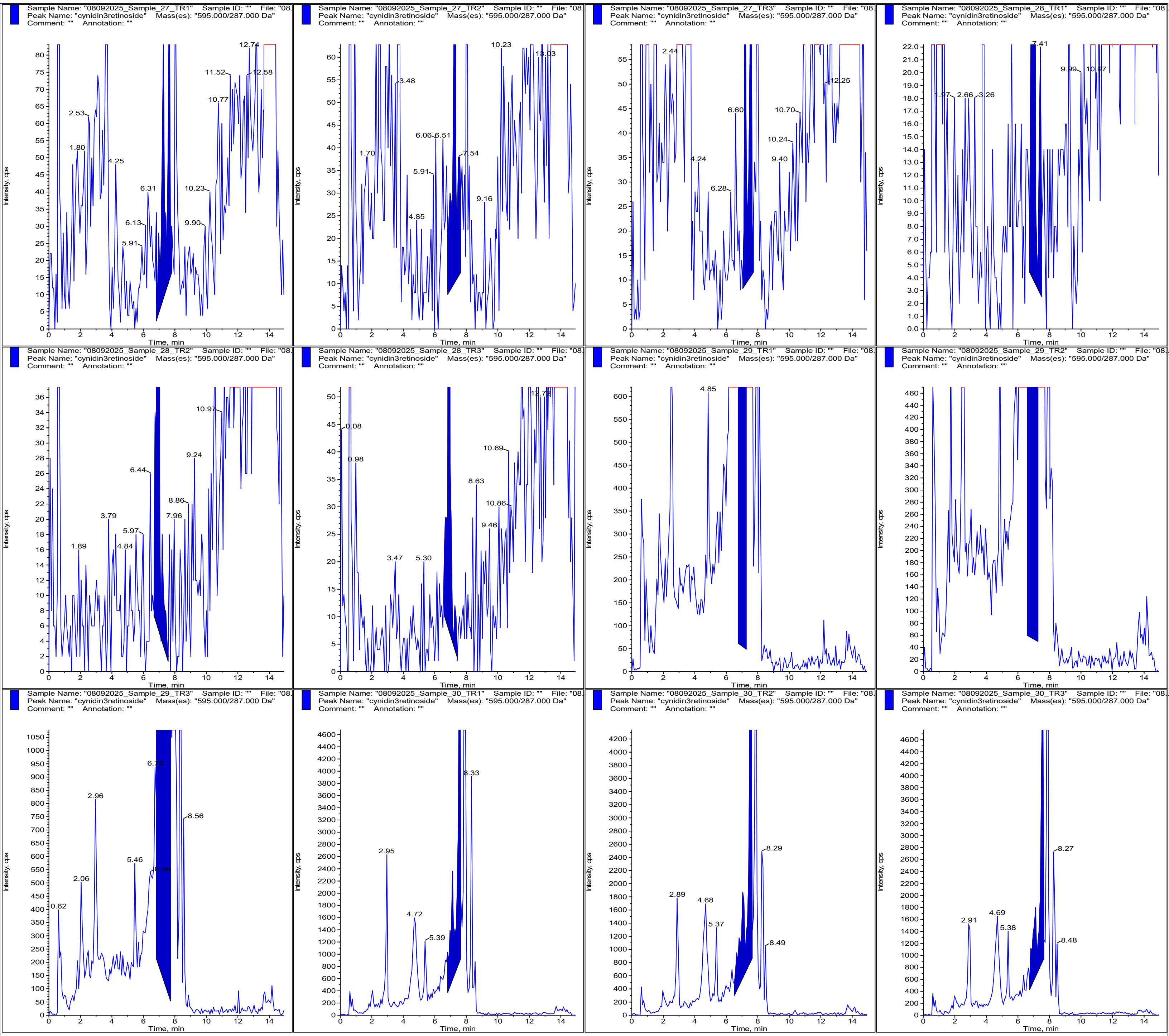

	Sample Name	Sample ID	Sample Type	File Name	Analyte Peak Area (counts)
109	08092025_Sample_27_TR1		Unknown	28072025_Akhil_Dr	1.71e+003
110	08092025_Sample_27_TR2		Unknown	28072025_Akhil_Dr	1.41e+003
111	08092025_Sample_27_TR3		Unknown	28072025_Akhil_Dr	1.26e+003
112	08092025_Sample_28_TR1		Unknown	28072025_Akhil_Dr	2.20e+003
113	08092025_Sample_28_TR2		Unknown	28072025_Akhil_Dr	1.60e+003
114	08092025_Sample_28_TR3		Unknown	28072025_Akhil_Dr	1.97e+003
115	08092025_Sample_29_TR1		Unknown	28072025_Akhil_Dr	9.50e+004

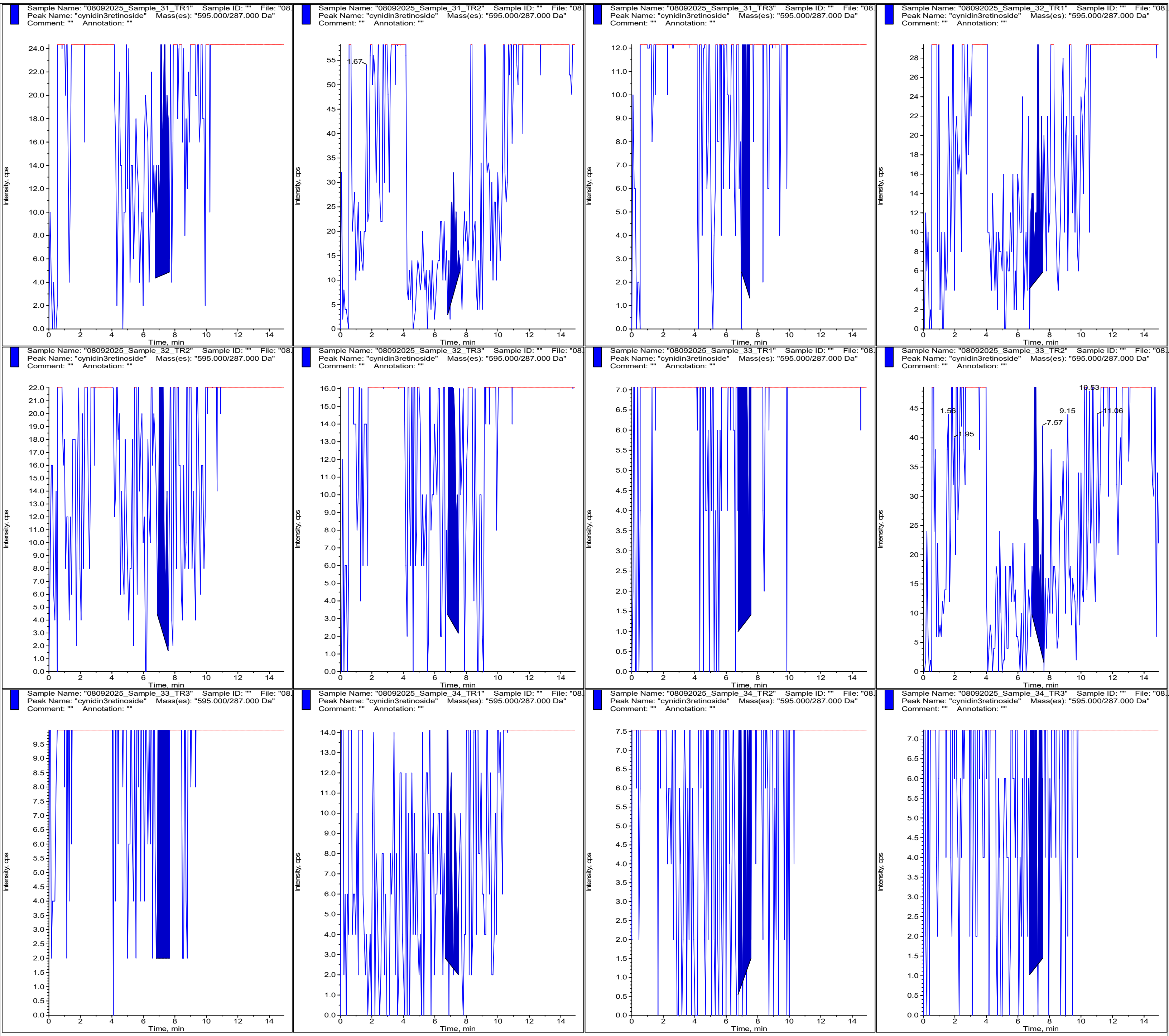

	Sample Name	Sample ID	Sample Type	File Name	Analyte Peak Area (counts)
121	08092025_Sample_31_TR1		Unknown	28072025_Akhil_Dr	6.39e+002
122	08092025_Sample_31_TR2		Unknown	28072025_Akhil_Dr	4.30e+002
123	08092025_Sample_31_TR3		Unknown	28072025_Akhil_Dr	4.27e+002
124	08092025_Sample_32_TR1		Unknown	28072025_Akhil_Dr	4.61e+002
125	08092025_Sample_32_TR2		Unknown	28072025_Akhil_Dr	5.04e+002
126	08092025_Sample_32_TR3		Unknown	28072025_Akhil_Dr	5.26e+002
127	08092025_Sample_33_TR1		Unknown	28072025_Akhil_Dr	8.99e+002

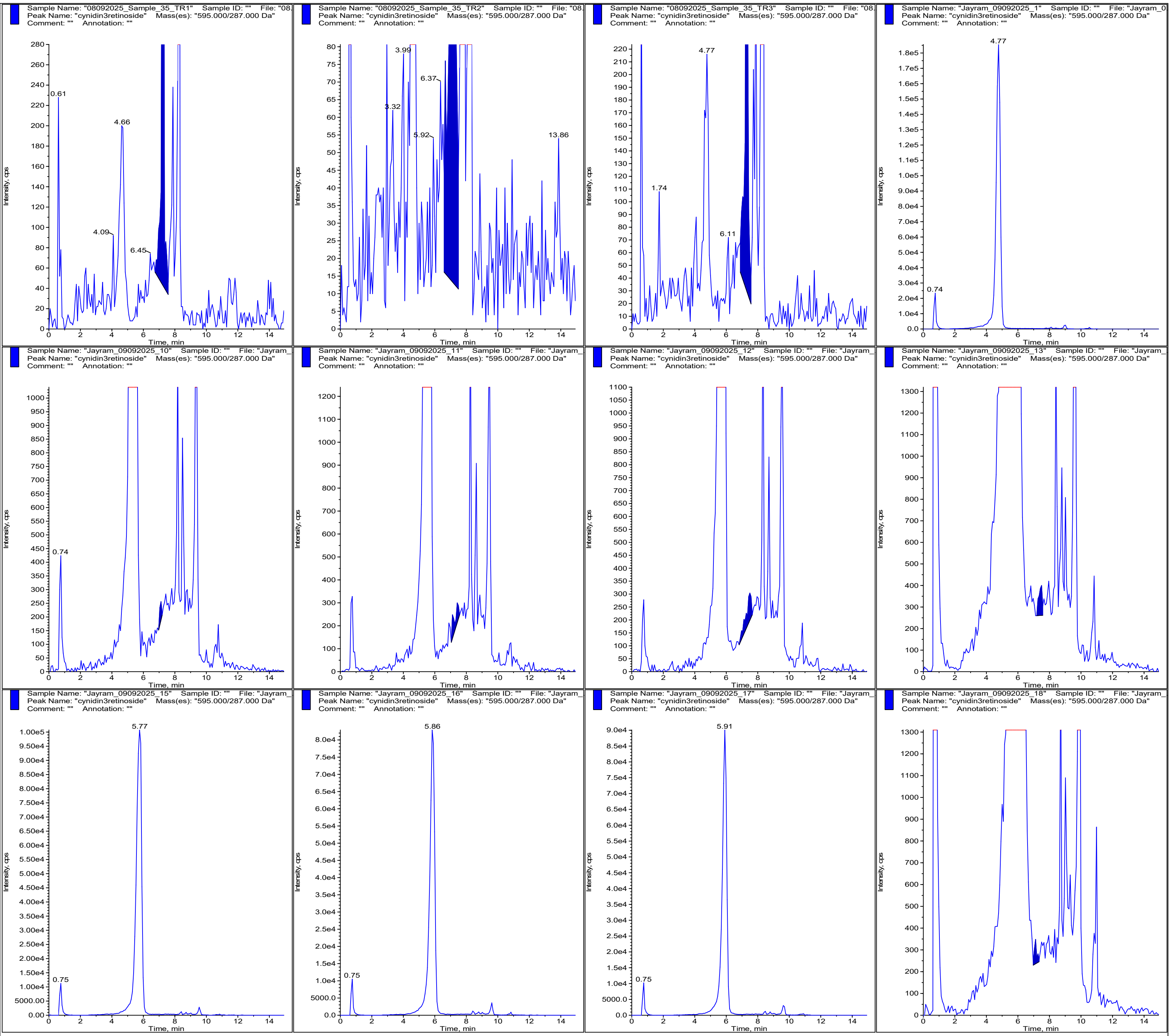

	Sample Name	Sample ID	Sample Type	File Name	Analyte Peak Area (counts)
133	08092025_Sample_35_TR1		Unknown	28072025_Akhil_Dr	6.45e+003
134	08092025_Sample_35_TR2		Unknown	28072025_Akhil_Dr	8.00e+003
135	08092025_Sample_35_TR3		Unknown	28072025_Akhil_Dr	9.10e+003
136	Jayram_09092025_1		Unknown	28072025_Akhil_Dr	0.00e+000
137	Jayram_09092025_10		Unknown	28072025_Akhil_Dr	5.91e+002
138	Jayram_09092025_11		Unknown	28072025_Akhil_Dr	1.71e+003
139	Jayram_09092025_12		Unknown	28072025_Akhil_Dr	2.50e+003

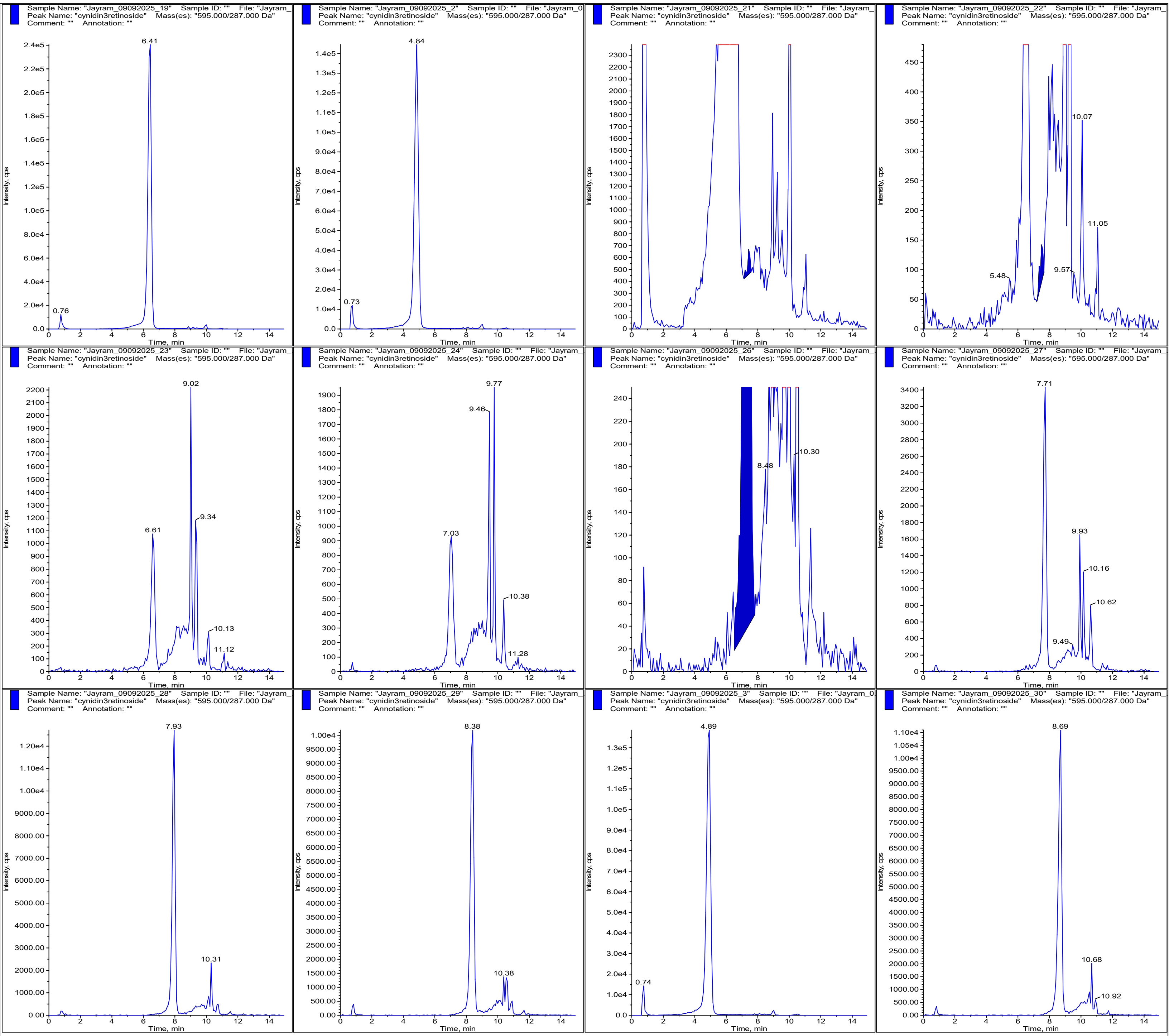

	Sample Name	Sample ID	Sample Type	File Name	Analyte Peak Area (counts)
145	Jayram_09092025_19		Unknown	28072025_Akhil_Dr	0.00e+000
146	Jayram_09092025_2		Unknown	28072025_Akhil_Dr	0.00e+000
147	Jayram_09092025_21		Unknown	28072025_Akhil_Dr	2.23e+003
148	Jayram_09092025_22		Unknown	28072025_Akhil_Dr	8.23e+002
149	Jayram_09092025_23		Unknown	28072025_Akhil_Dr	0.00e+000
150	Jayram_09092025_24		Unknown	28072025_Akhil_Dr	0.00e+000
151	Jayram_09092025_26		Unknown	28072025_Akhil_Dr	5.14e+004

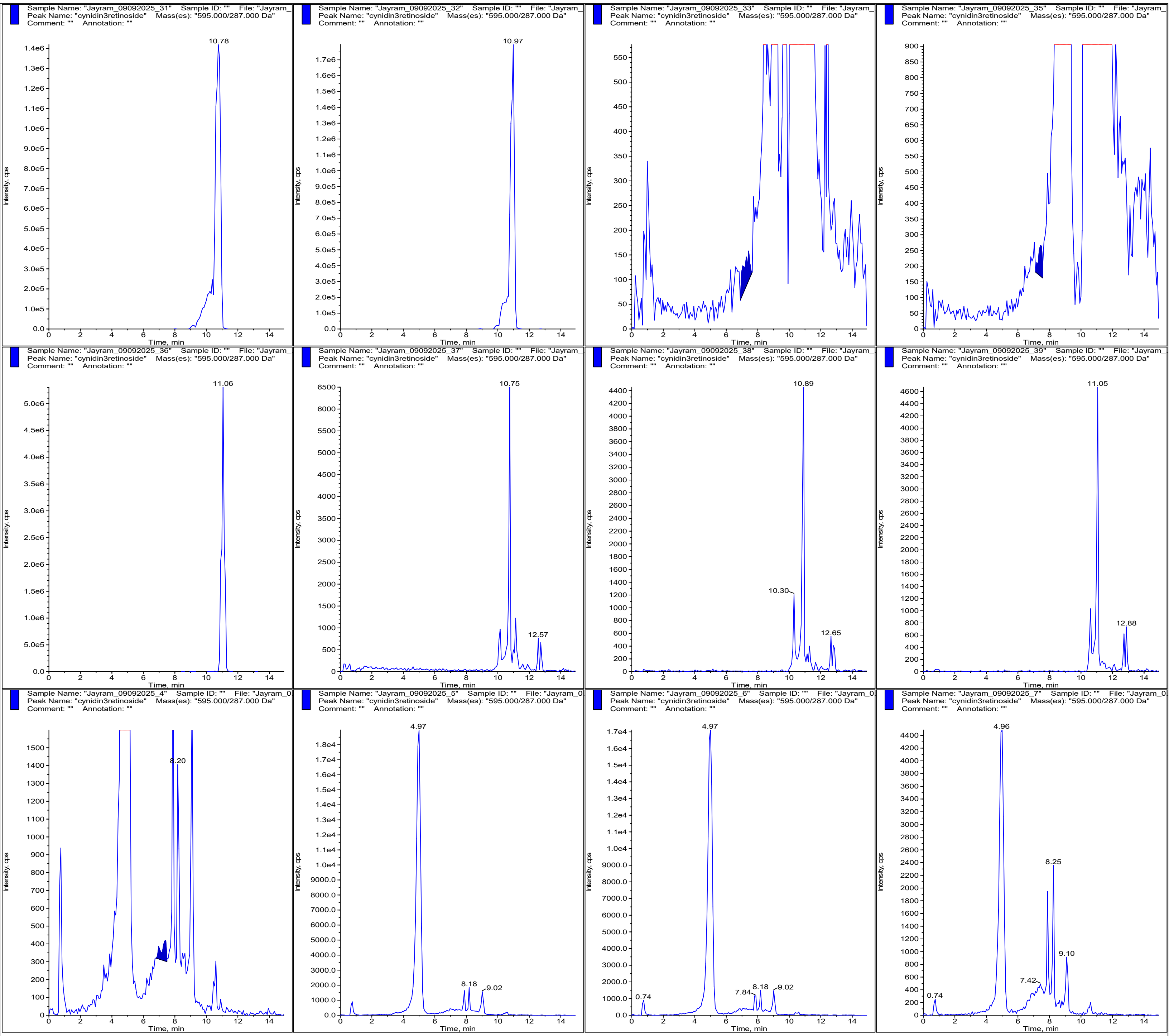

	Sample Name	Sample ID	Sample Type	File Name	Analyte Peak Area (counts)
157	Jayram_09092025_31		Unknown	28072025_Akhil_Dr	0.00e+000
158	Jayram_09092025_32		Unknown	28072025_Akhil_Dr	0.00e+000
159	Jayram_09092025_33		Unknown	28072025_Akhil_Dr	1.79e+003
160	Jayram_09092025_35		Unknown	28072025_Akhil_Dr	1.60e+003
161	Jayram_09092025_36		Unknown	28072025_Akhil_Dr	0.00e+000
162	Jayram_09092025_37		Unknown	28072025_Akhil_Dr	0.00e+000
163	Jayram_09092025_38		Unknown	28072025_Akhil_Dr	0.00e+000

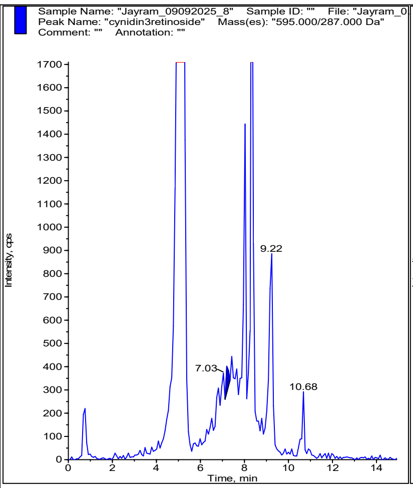

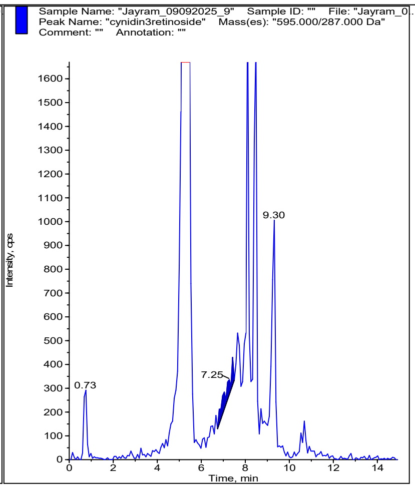

	Sample Name	Sample ID	Sample Type	File Name	Analyte Peak Area (counts)
169	Jayram_09092025_8		Unknown	28072025_Akhil_Dr	8.36e+002
170	Jayram_09092025_9		Unknown	28072025_Akhil_Dr	2.45e+003
