# Supplementary material for: A coordinated network of MYB regulators orchestrates anthocyanin biosynthesis in banana
Source: Hortic Res. 2026 Jan 13;13(6):uhaf361. doi: 10.1093/hr/uhaf361 (PMC13273576; doi:10.1093/hr/uhaf361)
Supplement: Web_Material_uhaf361 [file Web_Material_uhaf361.zip › supplementary file S6 (2).pdf]

Acq. File: 28072025_Akhil_DrNegi_Pos_Stds.dam,..

Sample Name: 20082025_Std_4_Dil_1
Sample Number: Sample 1 of 145

	Sample Name	Sample ID	Sample Type	File Name	Analyte Peak Area (counts)
1	20082025_Std_4_Dil_1		Standard	28072025_Akhil_Dr	4.66e+005
2	20082025_Std_4_Dil_2		Standard	28072025_Akhil_Dr	1.68e+005
3	20082025_Std_4_Dil_3		Standard	28072025_Akhil_Dr	6.03e+004
4	20082025_Std_4_Dil_4		Standard	28072025_Akhil_Dr	1.65e+004
5	20082025_Std_4_Dil_5		Standard	28072025_Akhil_Dr	3.00e+003
6	08092025_Sample_1_TR1		Unknown	28072025_Akhil_Dr	0.00e+000
7	08092025_Sample_1_TR2		Unknown	28072025_Akhil_Dr	0.00e+000
8	08092025_Sample_1_TR3		Unknown	28072025_Akhil_Dr	0.00e+000
9	08092025_Sample_2_TR1		Unknown	28072025_Akhil_Dr	0.00e+000
10	08092025_Sample_2_TR2		Unknown	28072025_Akhil_Dr	0.00e+000
11	08092025_Sample_2_TR3		Unknown	28072025_Akhil_Dr	0.00e+000
12	08092025_Sample_3_TR1		Unknown	28072025_Akhil_Dr	0.00e+000
13	08092025_Sample_3_TR2		Unknown	28072025_Akhil_Dr	0.00e+000
14	08092025_Sample_3_TR3		Unknown	28072025_Akhil_Dr	0.00e+000
15	08092025_Sample_4_TR1		Unknown	28072025_Akhil_Dr	0.00e+000
16	08092025_Sample_4_TR2		Unknown	28072025_Akhil_Dr	0.00e+000
17	08092025_Sample_4_TR3		Unknown	28072025_Akhil_Dr	0.00e+000
18	08092025_Sample_5_TR1		Unknown	28072025_Akhil_Dr	0.00e+000
19	08092025_Sample_5_TR2		Unknown	28072025_Akhil_Dr	2.22e+005
20	08092025_Sample_5_TR3		Unknown	28072025_Akhil_Dr	0.00e+000
21	08092025_Sample_6_TR1		Unknown	28072025_Akhil_Dr	1.28e+005

Sample Name: "20082025_Std_4_Dil_1" Sample ID: "" File: "20082025_Std_4_Dil_1.wiff"
Peak Name: "Catechin" Mass(es): "291.089/123.000 Da"
Comment: "" Annotation: ""

```
Sample Index:       1
Sample Type:      Standard
Concentration:      250.    ng/mL
Calculated Conc:    258.    ng/mL
Acq. Date:        8/20/2025
Acq. Time:        3:09:01 PM

Modified:           No
Proc. Algorithm: Analyst Classic
Bunching Factor:    1
Noise Threshold:  121.31   cps
Area Threshold:  606.56   cps
,Num. Smooths:       0
Sep. Width:         0.20
Sep. Height:        0.01
Exp. Peak Ratio:    5.00
Exp. Adj. Ratio:    4.00
Exp. Val. Ratio:    3.00
RT Window:         30.0    sec
Expected RT:        4.09   min
Use Relative RT:    No

Int. Type:        Base To Base
Retention Time:     4.07   min
Area:          4.66e+005   counts
Height:          4.32e+004  cps
Start Time:         3.11   min
End Time:           4.47   min
```

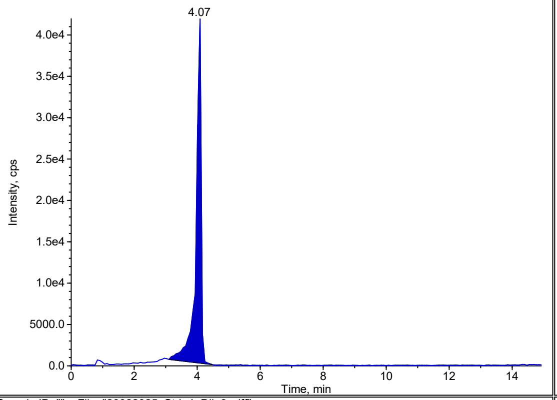

Sample Name: "20082025_Std_4_Dil_2" Sample ID: "" File: "20082025_Std_4_Dil_2.wiff"
Peak Name: "Catechin" Mass(es): "291.089/123.000 Da"
Comment: "" Annotation: ""

```
Sample Index:       1
Sample Type:      Standard
Concentration:      125.    ng/mL
Calculated Conc:    109.    ng/mL
Acq. Date:        8/20/2025
Acq. Time:        3:25:10 PM

Modified:           No
Proc. Algorithm: Analyst Classic
Bunching Factor:    1
Noise Threshold:  121.31   cps
Area Threshold:  606.56   cps
,Num. Smooths:       0
Sep. Width:         0.20
Sep. Height:        0.01
Exp. Peak Ratio:    5.00
Exp. Adj. Ratio:    4.00
Exp. Val. Ratio:    3.00
RT Window:         30.0    sec
Expected RT:        4.09   min
Use Relative RT:    No

Int. Type:        Base To Base
Retention Time:     4.07   min
Area:          1.68e+005   counts
Height:          1.74e+004  cps
Start Time:         3.41   min
End Time:           4.39   min
```

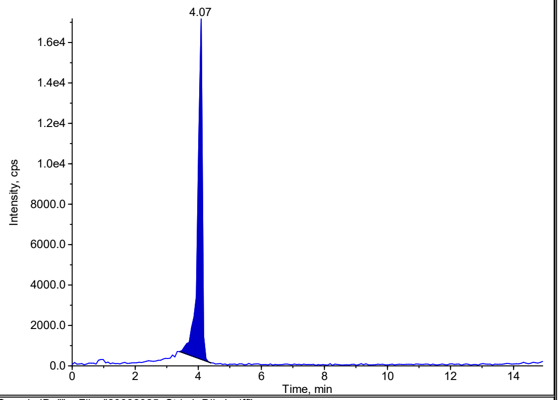

Sample Name: "20082025_Std_4_Dil_3" Sample ID: "" File: "20082025_Std_4_Dil_3.wiff"
Peak Name: "Catechin" Mass(es): "291.089/123.000 Da"
Comment: "" Annotation: ""

```
Sample Index:       1
Sample Type:      Standard
Concentration:      62.5    ng/mL
Calculated Conc:    55.7    ng/mL
Acq. Date:        8/20/2025
Acq. Time:        3:41:20 PM

Modified:           No
Proc. Algorithm: Analyst Classic
Bunching Factor:    1
Noise Threshold:  121.31   cps
Area Threshold:  606.56   cps
,Num. Smooths:       0
Sep. Width:         0.20
Sep. Height:        0.01
Exp. Peak Ratio:    5.00
Exp. Adj. Ratio:    4.00
Exp. Val. Ratio:    3.00
RT Window:         30.0    sec
Expected RT:        4.09   min
Use Relative RT:    No

Int. Type:        Base To Base
Retention Time:     4.02   min
Area:          6.03e+004   counts
Height:          5.87e+003  cps
Start Time:         3.33   min
End Time:           4.32   min
```

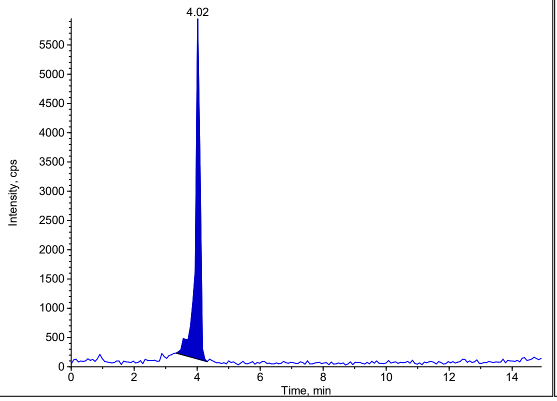

Sample Name: "20082025_Std_4_Dil_4" Sample ID: "" File: "20082025_Std_4_Dil_4.wiff"
Peak Name: "Catechin" Mass(es): "291.089/123.000 Da"
Comment: "" Annotation: ""

```
Sample Index:       1
Sample Type:      Standard
Concentration:      31.3    ng/mL
Calculated Conc:    33.8    ng/mL
Acq. Date:        8/20/2025
Acq. Time:        3:57:29 PM

Modified:           No
Proc. Algorithm: Analyst Classic
Bunching Factor:    1
Noise Threshold:  121.31   cps
Area Threshold:  606.56   cps
,Num. Smooths:       0
Sep. Width:         0.20
Sep. Height:        0.01
Exp. Peak Ratio:    5.00
Exp. Adj. Ratio:    4.00
Exp. Val. Ratio:    3.00
RT Window:         30.0    sec
Expected RT:        4.09   min
Use Relative RT:    No

Int. Type:        Base To Base
Retention Time:     4.02   min
Area:          1.65e+004   counts
Height:          1.92e+003  cps
Start Time:         3.48   min
End Time:           4.39   min
```

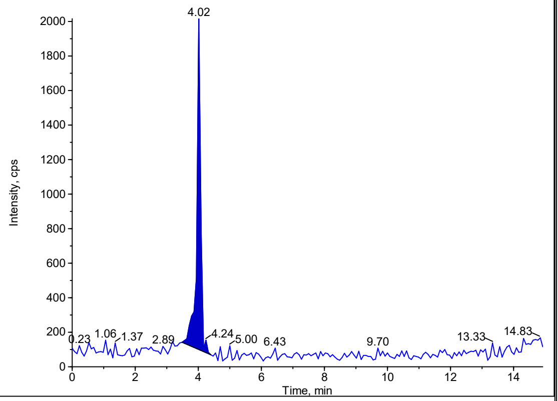

	Sample Name	Sample ID	Sample Type	File Name	Analyte Peak Area (counts)
1	20082025_Std_4_Dil_1		Standard	28072025_Akhil_Dr	4.66e+005
2	20082025_Std_4_Dil_2		Standard	28072025_Akhil_Dr	1.68e+005
3	20082025_Std_4_Dil_3		Standard	28072025_Akhil_Dr	6.03e+004
4	20082025_Std_4_Dil_4		Standard	28072025_Akhil_Dr	1.65e+004
5	20082025_Std_4_Dil_5		Standard	28072025_Akhil_Dr	3.00e+003
6	08092025_Sample_1_TR1		Unknown	28072025_Akhil_Dr	0.00e+000
7	08092025_Sample_1_TR2		Unknown	28072025_Akhil_Dr	0.00e+000
8	08092025_Sample_1_TR3		Unknown	28072025_Akhil_Dr	0.00e+000
9	08092025_Sample_2_TR1		Unknown	28072025_Akhil_Dr	0.00e+000
10	08092025_Sample_2_TR2		Unknown	28072025_Akhil_Dr	0.00e+000
11	08092025_Sample_2_TR3		Unknown	28072025_Akhil_Dr	0.00e+000
12	08092025_Sample_3_TR1		Unknown	28072025_Akhil_Dr	0.00e+000
13	08092025_Sample_3_TR2		Unknown	28072025_Akhil_Dr	0.00e+000
14	08092025_Sample_3_TR3		Unknown	28072025_Akhil_Dr	0.00e+000
15	08092025_Sample_4_TR1		Unknown	28072025_Akhil_Dr	0.00e+000
16	08092025_Sample_4_TR2		Unknown	28072025_Akhil_Dr	0.00e+000
17	08092025_Sample_4_TR3		Unknown	28072025_Akhil_Dr	0.00e+000
18	08092025_Sample_5_TR1		Unknown	28072025_Akhil_Dr	0.00e+000
19	08092025_Sample_5_TR2		Unknown	28072025_Akhil_Dr	2.22e+005
20	08092025_Sample_5_TR3		Unknown	28072025_Akhil_Dr	0.00e+000
21	08092025_Sample_6_TR1		Unknown	28072025_Akhil_Dr	1.28e+005
22	08092025_Sample_6_TR2		Unknown	28072025_Akhil_Dr	1.35e+005
23	08092025_Sample_7_TR1		Unknown	28072025_Akhil_Dr	3.96e+005
24	08092025_Sample_7_TR2		Unknown	28072025_Akhil_Dr	3.98e+005
25	08092025_Sample_7_TR3		Unknown	28072025_Akhil_Dr	4.04e+005
26	08092025_Sample_8_TR1		Unknown	28072025_Akhil_Dr	1.37e+004
27	08092025_Sample_8_TR2		Unknown	28072025_Akhil_Dr	3.01e+004
28	08092025_Sample_8_TR3		Unknown	28072025_Akhil_Dr	2.24e+004
29	08092025_Sample_9_TR1		Unknown	28072025_Akhil_Dr	2.09e+004
30	08092025_Sample_9_TR2		Unknown	28072025_Akhil_Dr	1.96e+004
31	08092025_Sample_9_TR3		Unknown	28072025_Akhil_Dr	2.39e+004
32	08092025_Sample_10_TR1		Unknown	28072025_Akhil_Dr	1.13e+005
33	08092025_Sample_10_TR2		Unknown	28072025_Akhil_Dr	1.39e+005
34	08092025_Sample_10_TR3		Unknown	28072025_Akhil_Dr	9.13e+004
35	08092025_Sample_11_TR2		Unknown	28072025_Akhil_Dr	4.10e+005
36	08092025_Sample_11_TR3		Unknown	28072025_Akhil_Dr	4.61e+005
37	08092025_Sample_12_TR1		Unknown	28072025_Akhil_Dr	1.58e+005
38	08092025_Sample_12_TR2		Unknown	28072025_Akhil_Dr	1.51e+005
39	08092025_Sample_12_TR3		Unknown	28072025_Akhil_Dr	1.54e+005
40	08092025_Sample_13_TR1		Unknown	28072025_Akhil_Dr	1.07e+005
41	08092025_Sample_13_TR2		Unknown	28072025_Akhil_Dr	1.00e+005
42	08092025_Sample_13_TR3		Unknown	28072025_Akhil_Dr	9.60e+004
43	08092025_Sample_14_TR1		Unknown	28072025_Akhil_Dr	1.02e+005
44	08092025_Sample_14_TR2		Unknown	28072025_Akhil_Dr	1.06e+005
45	08092025_Sample_14_TR3		Unknown	28072025_Akhil_Dr	9.53e+004

	Sample Name	Sample ID	Sample Type	File Name	Analyte Peak Area (counts)
46	08092025_Sample_15_TR1		Unknown	28072025_Akhil_Dr	1.15e+004
47	08092025_Sample_15_TR2		Unknown	28072025_Akhil_Dr	1.23e+004
48	08092025_Sample_16_TR1		Unknown	28072025_Akhil_Dr	4.40e+005
49	08092025_Sample_16_TR2		Unknown	28072025_Akhil_Dr	4.49e+005
50	08092025_Sample_16_TR3		Unknown	28072025_Akhil_Dr	3.87e+005
51	08092025_Sample_17_TR2		Unknown	28072025_Akhil_Dr	2.81e+005
52	08092025_Sample_17_TR3		Unknown	28072025_Akhil_Dr	3.14e+005
53	08092025_Sample_18_TR1		Unknown	28072025_Akhil_Dr	2.55e+004
54	08092025_Sample_18_TR2		Unknown	28072025_Akhil_Dr	2.80e+004
55	08092025_Sample_18_TR3		Unknown	28072025_Akhil_Dr	2.57e+004
56	08092025_Sample_19_TR1		Unknown	28072025_Akhil_Dr	3.20e+005
57	08092025_Sample_19_TR2		Unknown	28072025_Akhil_Dr	0.00e+000
58	08092025_Sample_19_TR3		Unknown	28072025_Akhil_Dr	0.00e+000
59	08092025_Sample_20_TR1		Unknown	28072025_Akhil_Dr	0.00e+000
60	08092025_Sample_20_TR2		Unknown	28072025_Akhil_Dr	0.00e+000
61	08092025_Sample_21_TR1		Unknown	28072025_Akhil_Dr	0.00e+000
62	08092025_Sample_21_TR2		Unknown	28072025_Akhil_Dr	0.00e+000
63	08092025_Sample_21_TR3		Unknown	28072025_Akhil_Dr	0.00e+000
64	08092025_Sample_22_TR2		Unknown	28072025_Akhil_Dr	0.00e+000
65	08092025_Sample_22_TR3		Unknown	28072025_Akhil_Dr	0.00e+000
66	08092025_Sample_22_TR1		Unknown	28072025_Akhil_Dr	0.00e+000
67	08092025_Sample_23_TR1		Unknown	28072025_Akhil_Dr	0.00e+000
68	08092025_Sample_23_TR2		Unknown	28072025_Akhil_Dr	0.00e+000
69	08092025_Sample_23_TR3		Unknown	28072025_Akhil_Dr	0.00e+000
70	08092025_Sample_24_TR1		Unknown	28072025_Akhil_Dr	0.00e+000
71	08092025_Sample_24_TR2		Unknown	28072025_Akhil_Dr	0.00e+000
72	08092025_Sample_24_TR3		Unknown	28072025_Akhil_Dr	0.00e+000
73	08092025_Sample_25_TR1		Unknown	28072025_Akhil_Dr	0.00e+000
74	08092025_Sample_25_TR2		Unknown	28072025_Akhil_Dr	0.00e+000
75	08092025_Sample_25_TR3		Unknown	28072025_Akhil_Dr	0.00e+000
76	08092025_Sample_26_TR1		Unknown	28072025_Akhil_Dr	0.00e+000
77	08092025_Sample_26_TR2		Unknown	28072025_Akhil_Dr	1.18e+003
78	08092025_Sample_26_TR3		Unknown	28072025_Akhil_Dr	0.00e+000
79	08092025_Sample_27_TR1		Unknown	28072025_Akhil_Dr	0.00e+000
80	08092025_Sample_27_TR2		Unknown	28072025_Akhil_Dr	0.00e+000
81	08092025_Sample_27_TR3		Unknown	28072025_Akhil_Dr	0.00e+000
82	08092025_Sample_28_TR1		Unknown	28072025_Akhil_Dr	0.00e+000
83	08092025_Sample_28_TR2		Unknown	28072025_Akhil_Dr	0.00e+000
84	08092025_Sample_28_TR3		Unknown	28072025_Akhil_Dr	0.00e+000
85	08092025_Sample_29_TR1		Unknown	28072025_Akhil_Dr	0.00e+000
86	08092025_Sample_29_TR2		Unknown	28072025_Akhil_Dr	0.00e+000
87	08092025_Sample_29_TR3		Unknown	28072025_Akhil_Dr	0.00e+000
88	08092025_Sample_30_TR1		Unknown	28072025_Akhil_Dr	0.00e+000
89	08092025_Sample_30_TR2		Unknown	28072025_Akhil_Dr	0.00e+000
90	08092025_Sample_31_TR1		Unknown	28072025_Akhil_Dr	0.00e+000

	Sample Name	Sample ID	Sample Type	File Name	Analyte Peak Area (counts)
91	08092025_Sample_31_TR2		Unknown	28072025_Akhil_Dr	0.00e+000
92	08092025_Sample_31_TR3		Unknown	28072025_Akhil_Dr	0.00e+000
93	08092025_Sample_32_TR1		Unknown	28072025_Akhil_Dr	0.00e+000
94	08092025_Sample_32_TR2		Unknown	28072025_Akhil_Dr	0.00e+000
95	08092025_Sample_32_TR3		Unknown	28072025_Akhil_Dr	0.00e+000
96	08092025_Sample_33_TR1		Unknown	28072025_Akhil_Dr	0.00e+000
97	08092025_Sample_33_TR3		Unknown	28072025_Akhil_Dr	0.00e+000
98	08092025_Sample_34_TR1		Unknown	28072025_Akhil_Dr	0.00e+000
99	08092025_Sample_34_TR2		Unknown	28072025_Akhil_Dr	0.00e+000
100	08092025_Sample_34_TR3		Unknown	28072025_Akhil_Dr	0.00e+000
101	08092025_Sample_35_TR1		Unknown	28072025_Akhil_Dr	3.21e+003
102	08092025_Sample_35_TR2		Unknown	28072025_Akhil_Dr	0.00e+000
103	08092025_Sample_35_TR3		Unknown	28072025_Akhil_Dr	0.00e+000
104	Jayram_09092025_1		Unknown	28072025_Akhil_Dr	0.00e+000
105	Jayram_09092025_10		Unknown	28072025_Akhil_Dr	0.00e+000
106	Jayram_09092025_11		Unknown	28072025_Akhil_Dr	0.00e+000
107	Jayram_09092025_12		Unknown	28072025_Akhil_Dr	0.00e+000
108	Jayram_09092025_13		Unknown	28072025_Akhil_Dr	0.00e+000
109	Jayram_09092025_14		Unknown	28072025_Akhil_Dr	0.00e+000
110	Jayram_09092025_15		Unknown	28072025_Akhil_Dr	0.00e+000
111	Jayram_09092025_16		Unknown	28072025_Akhil_Dr	0.00e+000
112	Jayram_09092025_17		Unknown	28072025_Akhil_Dr	0.00e+000
113	Jayram_09092025_18		Unknown	28072025_Akhil_Dr	0.00e+000
114	Jayram_09092025_19		Unknown	28072025_Akhil_Dr	0.00e+000
115	Jayram_09092025_2		Unknown	28072025_Akhil_Dr	0.00e+000
116	Jayram_09092025_20		Unknown	28072025_Akhil_Dr	0.00e+000
117	Jayram_09092025_21		Unknown	28072025_Akhil_Dr	0.00e+000
118	Jayram_09092025_22		Unknown	28072025_Akhil_Dr	0.00e+000
119	Jayram_09092025_23		Unknown	28072025_Akhil_Dr	0.00e+000
120	Jayram_09092025_24		Unknown	28072025_Akhil_Dr	0.00e+000
121	Jayram_09092025_25		Unknown	28072025_Akhil_Dr	0.00e+000
122	Jayram_09092025_26		Unknown	28072025_Akhil_Dr	0.00e+000
123	Jayram_09092025_27		Unknown	28072025_Akhil_Dr	0.00e+000
124	Jayram_09092025_28		Unknown	28072025_Akhil_Dr	0.00e+000
125	Jayram_09092025_29		Unknown	28072025_Akhil_Dr	0.00e+000
126	Jayram_09092025_3		Unknown	28072025_Akhil_Dr	0.00e+000
127	Jayram_09092025_30		Unknown	28072025_Akhil_Dr	0.00e+000
128	Jayram_09092025_31		Unknown	28072025_Akhil_Dr	0.00e+000
129	Jayram_09092025_32		Unknown	28072025_Akhil_Dr	0.00e+000
130	Jayram_09092025_33		Unknown	28072025_Akhil_Dr	0.00e+000
131	Jayram_09092025_34		Unknown	28072025_Akhil_Dr	0.00e+000
132	Jayram_09092025_35		Unknown	28072025_Akhil_Dr	0.00e+000
133	Jayram_09092025_36		Unknown	28072025_Akhil_Dr	0.00e+000
134	Jayram_09092025_37		Unknown	28072025_Akhil_Dr	0.00e+000
135	Jayram_09092025_38		Unknown	28072025_Akhil_Dr	0.00e+000

	Sample Name	Sample ID	Sample Type	File Name	Analyte Peak Area (counts)
136	Jayram_09092025_39		Unknown	28072025_Akhil_Dr	0.00e+000
137	Jayram_09092025_4		Unknown	28072025_Akhil_Dr	8.25e+003
138	Jayram_09092025_5		Unknown	28072025_Akhil_Dr	1.09e+004
139	Jayram_09092025_6		Unknown	28072025_Akhil_Dr	1.13e+004
140	Jayram_09092025_7		Unknown	28072025_Akhil_Dr	8.76e+003
141	08092025_Sample_6_TR3		Unknown	28072025_Akhil_Dr	1.60e+005
142	08092025_Sample_11_TR1		Unknown	28072025_Akhil_Dr	4.26e+005
143	08092025_Sample_15_TR3		Unknown	28072025_Akhil_Dr	1.32e+004
144	08092025_Sample_17_TR1		Unknown	28072025_Akhil_Dr	2.78e+005
145	08092025_Sample_20_TR3		Unknown	28072025_Akhil_Dr	0.00e+000

	Sample Name	Analyte Peak Height (cps)	Analyte Concentration (ng/mL)	Standard Query Status	Use Record	Record Modified
1	20082025_Std_4_Dil_1	4.32e+004	250.	Pass	☑	☐
2	20082025_Std_4_Dil_2	1.74e+004	125.	Pass	☑	☐
3	20082025_Std_4_Dil_3	5.87e+003	62.5	Pass	☑	☐
4	20082025_Std_4_Dil_4	1.92e+003	31.3	Pass	☑	☐
5	20082025_Std_4_Dil_5	5.16e+002	15.6	Fail	☑	☑
6	08092025_Sample_1_TR1	0.00e+000	N/A	N/A		☐
7	08092025_Sample_1_TR2	0.00e+000	N/A	N/A		☐
8	08092025_Sample_1_TR3	0.00e+000	N/A	N/A		☐
9	08092025_Sample_2_TR1	0.00e+000	N/A	N/A		☐
10	08092025_Sample_2_TR2	0.00e+000	N/A	N/A		☐
11	08092025_Sample_2_TR3	0.00e+000	N/A	N/A		☐
12	08092025_Sample_3_TR1	0.00e+000	N/A	N/A		☐
13	08092025_Sample_3_TR2	0.00e+000	N/A	N/A		☐
14	08092025_Sample_3_TR3	0.00e+000	N/A	N/A		☐
15	08092025_Sample_4_TR1	0.00e+000	N/A	N/A		☐
16	08092025_Sample_4_TR2	0.00e+000	N/A	N/A		☐
17	08092025_Sample_4_TR3	0.00e+000	N/A	N/A		☐
18	08092025_Sample_5_TR1	0.00e+000	N/A	N/A		☐
19	08092025_Sample_5_TR2	9.34e+003	N/A	N/A		☐
20	08092025_Sample_5_TR3	0.00e+000	N/A	N/A		☐
21	08092025_Sample_6_TR1	9.62e+003	N/A	N/A		☑
22	08092025_Sample_6_TR2	1.24e+004	N/A	N/A		☑
23	08092025_Sample_7_TR1	4.33e+004	N/A	N/A		☑
24	08092025_Sample_7_TR2	4.73e+004	N/A	N/A		☑
25	08092025_Sample_7_TR3	3.73e+004	N/A	N/A		☑
26	08092025_Sample_8_TR1	1.95e+003	N/A	N/A		☑
27	08092025_Sample_8_TR2	2.78e+003	N/A	N/A		☑
28	08092025_Sample_8_TR3	1.71e+003	N/A	N/A		☑
29	08092025_Sample_9_TR1	2.02e+003	N/A	N/A		☑
30	08092025_Sample_9_TR2	2.79e+003	N/A	N/A		☑
31	08092025_Sample_9_TR3	1.84e+003	N/A	N/A		☑
32	08092025_Sample_10_TR1	7.99e+003	N/A	N/A		☑
33	08092025_Sample_10_TR2	9.33e+003	N/A	N/A		☑
34	08092025_Sample_10_TR3	8.84e+003	N/A	N/A		☑
35	08092025_Sample_11_TR2	3.63e+004	N/A	N/A		☑
36	08092025_Sample_11_TR3	5.00e+004	N/A	N/A		☑
37	08092025_Sample_12_TR1	2.33e+004	N/A	N/A		☑
38	08092025_Sample_12_TR2	1.98e+004	N/A	N/A		☑
39	08092025_Sample_12_TR3	1.80e+004	N/A	N/A		☑
40	08092025_Sample_13_TR1	1.48e+004	N/A	N/A		☑
41	08092025_Sample_13_TR2	1.11e+004	N/A	N/A		☑
42	08092025_Sample_13_TR3	1.36e+004	N/A	N/A		☑
43	08092025_Sample_14_TR1	1.00e+004	N/A	N/A		☑
44	08092025_Sample_14_TR2	9.93e+003	N/A	N/A		☑
45	08092025_Sample_14_TR3	1.01e+004	N/A	N/A		☑

	Sample Name	Analyte Peak Height (cps)	Analyte Concentration (ng/mL)	Standard Query Status	Use Record	Record Modified
46	08092025_Sample_15_TR1	1.40e+003	N/A	N/A		☑
47	08092025_Sample_15_TR2	8.85e+002	N/A	N/A		☑
48	08092025_Sample_16_TR1	4.81e+004	N/A	N/A		☑
49	08092025_Sample_16_TR2	3.65e+004	N/A	N/A		☑
50	08092025_Sample_16_TR3	3.77e+004	N/A	N/A		☑
51	08092025_Sample_17_TR2	4.29e+004	N/A	N/A		☑
52	08092025_Sample_17_TR3	3.91e+004	N/A	N/A		☑
53	08092025_Sample_18_TR1	1.82e+003	N/A	N/A		☑
54	08092025_Sample_18_TR2	2.07e+003	N/A	N/A		☑
55	08092025_Sample_18_TR3	2.14e+003	N/A	N/A		☑
56	08092025_Sample_19_TR1	4.20e+004	N/A	N/A		☑
57	08092025_Sample_19_TR2	0.00e+000	N/A	N/A		☐
58	08092025_Sample_19_TR3	0.00e+000	N/A	N/A		☐
59	08092025_Sample_20_TR1	0.00e+000	N/A	N/A		☐
60	08092025_Sample_20_TR2	0.00e+000	N/A	N/A		☐
61	08092025_Sample_21_TR1	0.00e+000	N/A	N/A		☐
62	08092025_Sample_21_TR2	0.00e+000	N/A	N/A		☐
63	08092025_Sample_21_TR3	0.00e+000	N/A	N/A		☐
64	08092025_Sample_22_TR2	0.00e+000	N/A	N/A		☐
65	08092025_Sample_22_TR3	0.00e+000	N/A	N/A		☐
66	08092025_Sample_22_TR1	0.00e+000	N/A	N/A		☐
67	08092025_Sample_23_TR1	0.00e+000	N/A	N/A		☐
68	08092025_Sample_23_TR2	0.00e+000	N/A	N/A		☐
69	08092025_Sample_23_TR3	0.00e+000	N/A	N/A		☐
70	08092025_Sample_24_TR1	0.00e+000	N/A	N/A		☐
71	08092025_Sample_24_TR2	0.00e+000	N/A	N/A		☐
72	08092025_Sample_24_TR3	0.00e+000	N/A	N/A		☐
73	08092025_Sample_25_TR1	0.00e+000	N/A	N/A		☐
74	08092025_Sample_25_TR2	0.00e+000	N/A	N/A		☐
75	08092025_Sample_25_TR3	0.00e+000	N/A	N/A		☐
76	08092025_Sample_26_TR1	0.00e+000	N/A	N/A		☐
77	08092025_Sample_26_TR2	1.76e+002	N/A	N/A		☑
78	08092025_Sample_26_TR3	0.00e+000	N/A	N/A		☐
79	08092025_Sample_27_TR1	0.00e+000	N/A	N/A		☐
80	08092025_Sample_27_TR2	0.00e+000	N/A	N/A		☐
81	08092025_Sample_27_TR3	0.00e+000	N/A	N/A		☐
82	08092025_Sample_28_TR1	0.00e+000	N/A	N/A		☐
83	08092025_Sample_28_TR2	0.00e+000	N/A	N/A		☐
84	08092025_Sample_28_TR3	0.00e+000	N/A	N/A		☐
85	08092025_Sample_29_TR1	0.00e+000	N/A	N/A		☐
86	08092025_Sample_29_TR2	0.00e+000	N/A	N/A		☐
87	08092025_Sample_29_TR3	0.00e+000	N/A	N/A		☐
88	08092025_Sample_30_TR1	0.00e+000	N/A	N/A		☐
89	08092025_Sample_30_TR2	0.00e+000	N/A	N/A		☐
90	08092025_Sample_31_TR1	0.00e+000	N/A	N/A		☐

	Sample Name	Analyte Peak Height (cps)	Analyte Concentration (ng/mL)	Standard Query Status	Use Record	Record Modified
91	08092025_Sample_31_TR2	0.00e+000	N/A	N/A		☐
92	08092025_Sample_31_TR3	0.00e+000	N/A	N/A		☐
93	08092025_Sample_32_TR1	0.00e+000	N/A	N/A		☐
94	08092025_Sample_32_TR2	0.00e+000	N/A	N/A		☐
95	08092025_Sample_32_TR3	0.00e+000	N/A	N/A		☐
96	08092025_Sample_33_TR1	0.00e+000	N/A	N/A		☐
97	08092025_Sample_33_TR3	0.00e+000	N/A	N/A		☐
98	08092025_Sample_34_TR1	0.00e+000	N/A	N/A		☐
99	08092025_Sample_34_TR2	0.00e+000	N/A	N/A		☐
100	08092025_Sample_34_TR3	0.00e+000	N/A	N/A		☐
101	08092025_Sample_35_TR1	3.34e+002	N/A	N/A		☑
102	08092025_Sample_35_TR2	0.00e+000	N/A	N/A		☐
103	08092025_Sample_35_TR3	0.00e+000	N/A	N/A		☐
104	Jayram_09092025_1	0.00e+000	N/A	N/A		☐
105	Jayram_09092025_10	0.00e+000	N/A	N/A		☐
106	Jayram_09092025_11	0.00e+000	N/A	N/A		☐
107	Jayram_09092025_12	0.00e+000	N/A	N/A		☐
108	Jayram_09092025_13	0.00e+000	N/A	N/A		☐
109	Jayram_09092025_14	0.00e+000	N/A	N/A		☐
110	Jayram_09092025_15	0.00e+000	N/A	N/A		☐
111	Jayram_09092025_16	0.00e+000	N/A	N/A		☐
112	Jayram_09092025_17	0.00e+000	N/A	N/A		☐
113	Jayram_09092025_18	0.00e+000	N/A	N/A		☐
114	Jayram_09092025_19	0.00e+000	N/A	N/A		☐
115	Jayram_09092025_2	0.00e+000	N/A	N/A		☐
116	Jayram_09092025_20	0.00e+000	N/A	N/A		☐
117	Jayram_09092025_21	0.00e+000	N/A	N/A		☐
118	Jayram_09092025_22	0.00e+000	N/A	N/A		☐
119	Jayram_09092025_23	0.00e+000	N/A	N/A		☐
120	Jayram_09092025_24	0.00e+000	N/A	N/A		☐
121	Jayram_09092025_25	0.00e+000	N/A	N/A		☐
122	Jayram_09092025_26	0.00e+000	N/A	N/A		☐
123	Jayram_09092025_27	0.00e+000	N/A	N/A		☐
124	Jayram_09092025_28	0.00e+000	N/A	N/A		☐
125	Jayram_09092025_29	0.00e+000	N/A	N/A		☐
126	Jayram_09092025_3	0.00e+000	N/A	N/A		☐
127	Jayram_09092025_30	0.00e+000	N/A	N/A		☐
128	Jayram_09092025_31	0.00e+000	N/A	N/A		☐
129	Jayram_09092025_32	0.00e+000	N/A	N/A		☐
130	Jayram_09092025_33	0.00e+000	N/A	N/A		☐
131	Jayram_09092025_34	0.00e+000	N/A	N/A		☐
132	Jayram_09092025_35	0.00e+000	N/A	N/A		☐
133	Jayram_09092025_36	0.00e+000	N/A	N/A		☐
134	Jayram_09092025_37	0.00e+000	N/A	N/A		☐
135	Jayram_09092025_38	0.00e+000	N/A	N/A		☐

	Sample Name	Analyte Peak Height (cps)	Analyte Concentration (ng/mL)	Standard Query Status	Use Record	Record Modified
136	Jayram_09092025_39	0.00e+000	N/A	N/A		☐
137	Jayram_09092025_4	1.01e+003	N/A	N/A		☐
138	Jayram_09092025_5	1.21e+003	N/A	N/A		☐
139	Jayram_09092025_6	1.36e+003	N/A	N/A		☐
140	Jayram_09092025_7	9.41e+002	N/A	N/A		☐
141	08092025_Sample_6_TR3	1.28e+004	N/A	N/A		☑
142	08092025_Sample_11_TR1	5.28e+004	N/A	N/A		☑
143	08092025_Sample_15_TR3	9.49e+002	N/A	N/A		☑
144	08092025_Sample_17_TR1	3.97e+004	N/A	N/A		☑
145	08092025_Sample_20_TR3	0.00e+000	N/A	N/A		☐

Acq. File: 28072025_Akhil_DrNegi_Pos_Stds.dam,..

Sample Name: 20082025_Std_4_Dil_1
Sample Number: Sample 1 of 145

	Sample Name	Calculated Concentration (ng/mL)	Accuracy (%)
1	20082025_Std_4_Dil_1	258.	103.
2	20082025_Std_4_Dil_2	109.	87.5
3	20082025_Std_4_Dil_3	55.7	89.1
4	20082025_Std_4_Dil_4	33.8	108.
5	20082025_Std_4_Dil_5	27.0	173.
6	08092025_Sample_1_TR1 NR CONTROL	No Peak	N/A
7	08092025_Sample_1_TR2	No Peak	N/A
8	08092025_Sample_1_TR3	No Peak	N/A
9	08092025_Sample_2_TR1	No Peak	N/A
10	08092025_Sample_2_TR2	No Peak	N/A
11	08092025_Sample_2_TR3	No Peak	N/A
12	08092025_Sample_3_TR1	No Peak	N/A
13	08092025_Sample_3_TR2	No Peak	N/A
14	08092025_Sample_3_TR3	No Peak	N/A
15	08092025_Sample_4_TR1	No Peak	N/A
16	08092025_Sample_4_TR2	No Peak	N/A
17	08092025_Sample_4_TR3	No Peak	N/A
18	08092025_Sample_5_TR1	No Peak	N/A
19	08092025_Sample_5_TR2	136.	N/A
20	08092025_Sample_5_TR3	No Peak	N/A
21	08092025_Sample_6_TR1 MA2+TT8I[illegible]	89.6	N/A
22	08092025_Sample_6_TR2	93.2	N/A
23	08092025_Sample_7_TR1 MA2+TT8I[illegible]	223.	N/A
24	08092025_Sample_7_TR2	225.	N/A
25	08092025_Sample_7_TR3	227.	N/A
26	08092025_Sample_8_TR1 MA2+TT8I2	32.4	N/A
27	08092025_Sample_8_TR2	40.6	N/A
28	08092025_Sample_8_TR3	36.8	N/A
29	08092025_Sample_9_TR1 MA4I1	36.0	N/A
30	08092025_Sample_9_TR2	35.3	N/A
31	08092025_Sample_9_TR3	37.5	N/A
32	08092025_Sample_10_TR1 MA4 L6	81.8	N/A
33	08092025_Sample_10_TR2	94.9	N/A
34	08092025_Sample_10_TR3	71.1	N/A
35	08092025_Sample_11_TR2 MA4 L7	230.	N/A
36	08092025_Sample_11_TR3	256.	N/A
37	08092025_Sample_12_TR1 MA4+TT8L3	104.	N/A
38	08092025_Sample_12_TR2	101.	N/A
39	08092025_Sample_12_TR3	103.	N/A
40	08092025_Sample_13_TR1 MA4+TT8L[illegible]	79.2	N/A
41	08092025_Sample_13_TR2	75.8	N/A
42	08092025_Sample_13_TR3	73.5	N/A
43	08092025_Sample_14_TR1 MA4+TT8L1[illegible]	76.5	N/A
44	08092025_Sample_14_TR2	78.4	N/A
45	08092025_Sample_14_TR3	73.2	N/A

	Sample Name	Calculated Concentration (ng/mL)	Accuracy (%)
46	08092025_Sample_15_TR1 MA4+TT8L1[illegible]	31.3	N/A
47	08092025_Sample_15_TR2	31.7	N/A
48	08092025_Sample_16_TR1 MA4+TT8L1	246.	N/A
49	08092025_Sample_16_TR2	250.	N/A
50	08092025_Sample_16_TR3	219.	N/A
51	08092025_Sample_17_TR2	166.	N/A
52	08092025_Sample_17_TR3	183.	N/A
53	08092025_Sample_18_TR1	38.3	N/A
54	08092025_Sample_18_TR2	39.5	N/A
55	08092025_Sample_18_TR3	38.4	N/A
56	08092025_Sample_19_TR1	186.	N/A
57	08092025_Sample_19_TR2	No Peak	N/A
58	08092025_Sample_19_TR3	No Peak	N/A
59	08092025_Sample_20_TR1	No Peak	N/A
60	08092025_Sample_20_TR2	No Peak	N/A
61	08092025_Sample_21_TR1	No Peak	N/A
62	08092025_Sample_21_TR2	No Peak	N/A
63	08092025_Sample_21_TR3	No Peak	N/A
64	08092025_Sample_22_TR2	No Peak	N/A
65	08092025_Sample_22_TR3	No Peak	N/A
66	08092025_Sample_22_TR1	No Peak	N/A
67	08092025_Sample_23_TR1	No Peak	N/A
68	08092025_Sample_23_TR2	No Peak	N/A
69	08092025_Sample_23_TR3	No Peak	N/A
70	08092025_Sample_24_TR1	No Peak	N/A
71	08092025_Sample_24_TR2	No Peak	N/A
72	08092025_Sample_24_TR3	No Peak	N/A
73	08092025_Sample_25_TR1	No Peak	N/A
74	08092025_Sample_25_TR2	No Peak	N/A
75	08092025_Sample_25_TR3	No Peak	N/A
76	08092025_Sample_26_TR1	No Peak	N/A
77	08092025_Sample_26_TR2	26.1	N/A
78	08092025_Sample_26_TR3	No Peak	N/A
79	08092025_Sample_27_TR1	No Peak	N/A
80	08092025_Sample_27_TR2	No Peak	N/A
81	08092025_Sample_27_TR3	No Peak	N/A
82	08092025_Sample_28_TR1	No Peak	N/A
83	08092025_Sample_28_TR2	No Peak	N/A
84	08092025_Sample_28_TR3	No Peak	N/A
85	08092025_Sample_29_TR1	No Peak	N/A
86	08092025_Sample_29_TR2	No Peak	N/A
87	08092025_Sample_29_TR3	No Peak	N/A
88	08092025_Sample_30_TR1	No Peak	N/A
89	08092025_Sample_30_TR2	No Peak	N/A
90	08092025_Sample_31_TR1	No Peak	N/A

	Sample Name	Calculated Concentration (ng/mL)	Accuracy (%)
91	08092025_Sample_31_TR2	No Peak	N/A
92	08092025_Sample_31_TR3	No Peak	N/A
93	08092025_Sample_32_TR1	No Peak	N/A
94	08092025_Sample_32_TR2	No Peak	N/A
95	08092025_Sample_32_TR3	No Peak	N/A
96	08092025_Sample_33_TR1	No Peak	N/A
97	08092025_Sample_33_TR3	No Peak	N/A
98	08092025_Sample_34_TR1	No Peak	N/A
99	08092025_Sample_34_TR2	No Peak	N/A
100	08092025_Sample_34_TR3	No Peak	N/A
101	08092025_Sample_35_TR1	27.1	N/A
102	08092025_Sample_35_TR2	No Peak	N/A
103	08092025_Sample_35_TR3	No Peak	N/A
104	Jayram_09092025_1	No Peak	N/A
105	Jayram_09092025_10	No Peak	N/A
106	Jayram_09092025_11	No Peak	N/A
107	Jayram_09092025_12	No Peak	N/A
108	Jayram_09092025_13	No Peak	N/A
109	Jayram_09092025_14	No Peak	N/A
110	Jayram_09092025_15	No Peak	N/A
111	Jayram_09092025_16	No Peak	N/A
112	Jayram_09092025_17	No Peak	N/A
113	Jayram_09092025_18	No Peak	N/A
114	Jayram_09092025_19	No Peak	N/A
115	Jayram_09092025_2	No Peak	N/A
116	Jayram_09092025_20	No Peak	N/A
117	Jayram_09092025_21	No Peak	N/A
118	Jayram_09092025_22	No Peak	N/A
119	Jayram_09092025_23	No Peak	N/A
120	Jayram_09092025_24	No Peak	N/A
121	Jayram_09092025_25	No Peak	N/A
122	Jayram_09092025_26	No Peak	N/A
123	Jayram_09092025_27	No Peak	N/A
124	Jayram_09092025_28	No Peak	N/A
125	Jayram_09092025_29	No Peak	N/A
126	Jayram_09092025_3	No Peak	N/A
127	Jayram_09092025_30	No Peak	N/A
128	Jayram_09092025_31	No Peak	N/A
129	Jayram_09092025_32	No Peak	N/A
130	Jayram_09092025_33	No Peak	N/A
131	Jayram_09092025_34	No Peak	N/A
132	Jayram_09092025_35	No Peak	N/A
133	Jayram_09092025_36	No Peak	N/A
134	Jayram_09092025_37	No Peak	N/A
135	Jayram_09092025_38	No Peak	N/A

	Sample Name	Calculated Concentration (ng/mL)	Accuracy (%)
136	Jayram_09092025_39	No Peak	N/A
137	Jayram_09092025_4	29.7	N/A
138	Jayram_09092025_5	31.0	N/A
139	Jayram_09092025_6	31.2	N/A
140	Jayram_09092025_7	29.9	N/A
141	08092025_Sample_6_TR3 MA2+TT8l(	106.	N/A
142	08092025_Sample_11_TR1	239.	N/A
143	08092025_Sample_15_TR3	32.1	N/A
144	08092025_Sample_17_TR1	165.	N/A
145	08092025_Sample_20_TR3	No Peak	N/A

Acq. File:
28072025_Akhil_DrNegi_Pos_Stds.dam,..

Sample Name: 20082025_Std_4_Dil_1
Sample Number: Sample 1 of 145

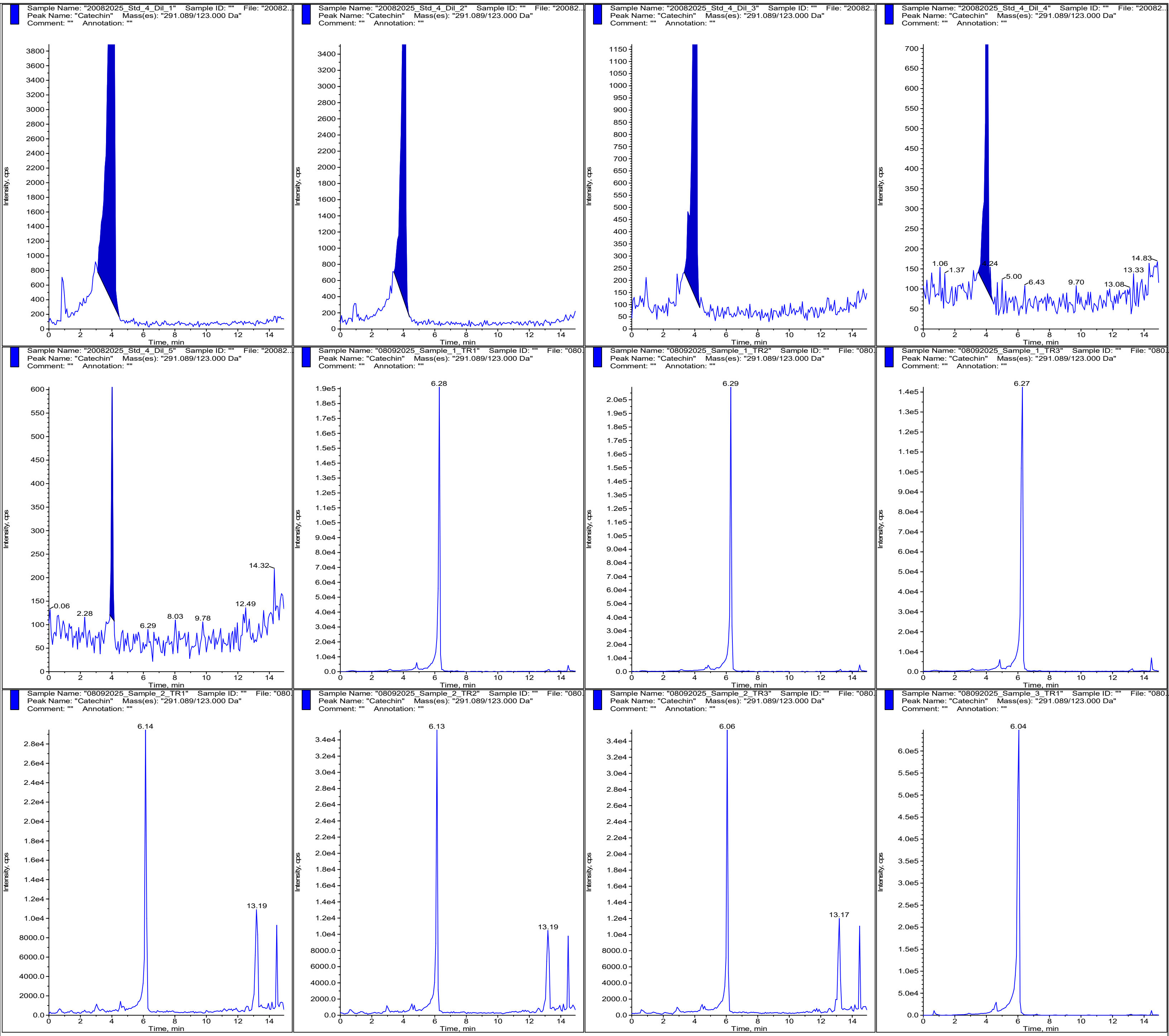

	Sample Name	Sample ID	Sample Type	File Name	Analyte Peak Area (counts)
1	20082025_Std_4_Dil_1		Standard	28072025_Akhil_Dr	4.66e+005
2	20082025_Std_4_Dil_2		Standard	28072025_Akhil_Dr	1.68e+005
3	20082025_Std_4_Dil_3		Standard	28072025_Akhil_Dr	6.03e+004
4	20082025_Std_4_Dil_4		Standard	28072025_Akhil_Dr	1.65e+004
5	20082025_Std_4_Dil_5		Standard	28072025_Akhil_Dr	3.00e+003
6	08092025_Sample_1_TR1		Unknown	28072025_Akhil_Dr	0.00e+000
7	08092025_Sample_1_TR2		Unknown	28072025_Akhil_Dr	0.00e+000

Acq. File: 28072025_Akhil_DrNegi_Pos_Stds.dam,..

Sample Name: 20082025_Std_4_Dil_1

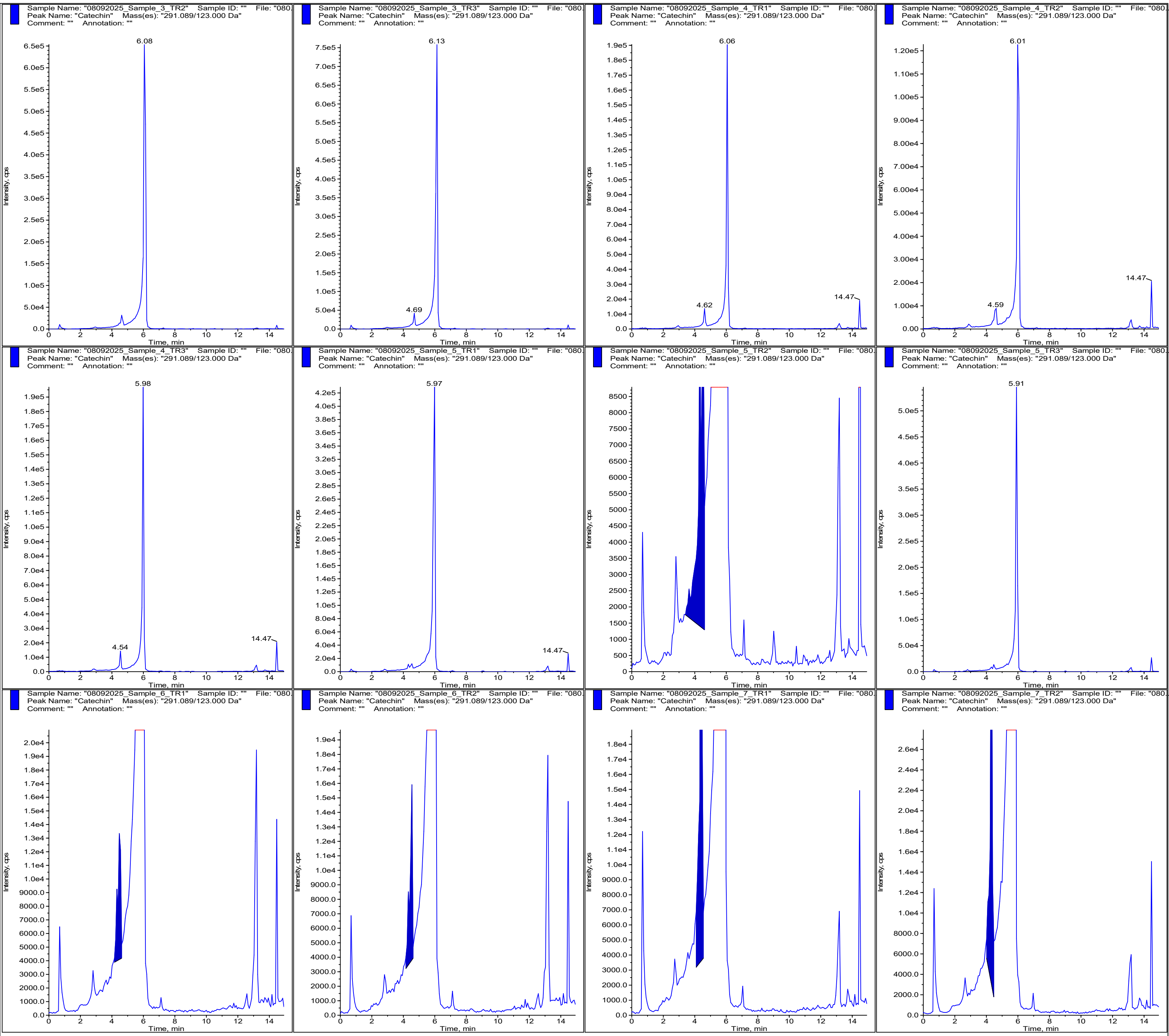

	Sample Name	Sample ID	Sample Type	File Name	Analyte Peak Area (counts)
13	08092025_Sample_3_TR2		Unknown	28072025_Akhil_Dr	0.00e+000
14	08092025_Sample_3_TR3		Unknown	28072025_Akhil_Dr	0.00e+000
15	08092025_Sample_4_TR1		Unknown	28072025_Akhil_Dr	0.00e+000
16	08092025_Sample_4_TR2		Unknown	28072025_Akhil_Dr	0.00e+000
17	08092025_Sample_4_TR3		Unknown	28072025_Akhil_Dr	0.00e+000
18	08092025_Sample_5_TR1		Unknown	28072025_Akhil_Dr	0.00e+000
19	08092025_Sample_5_TR2		Unknown	28072025_Akhil_Dr	2.22e+005

Acq. File: 28072025_Akhil_DrNegi_Pos_Stds.dam,..

Sample Name: 20082025_Std_4_Dil_1
Sample Number: Sample 1 of 145

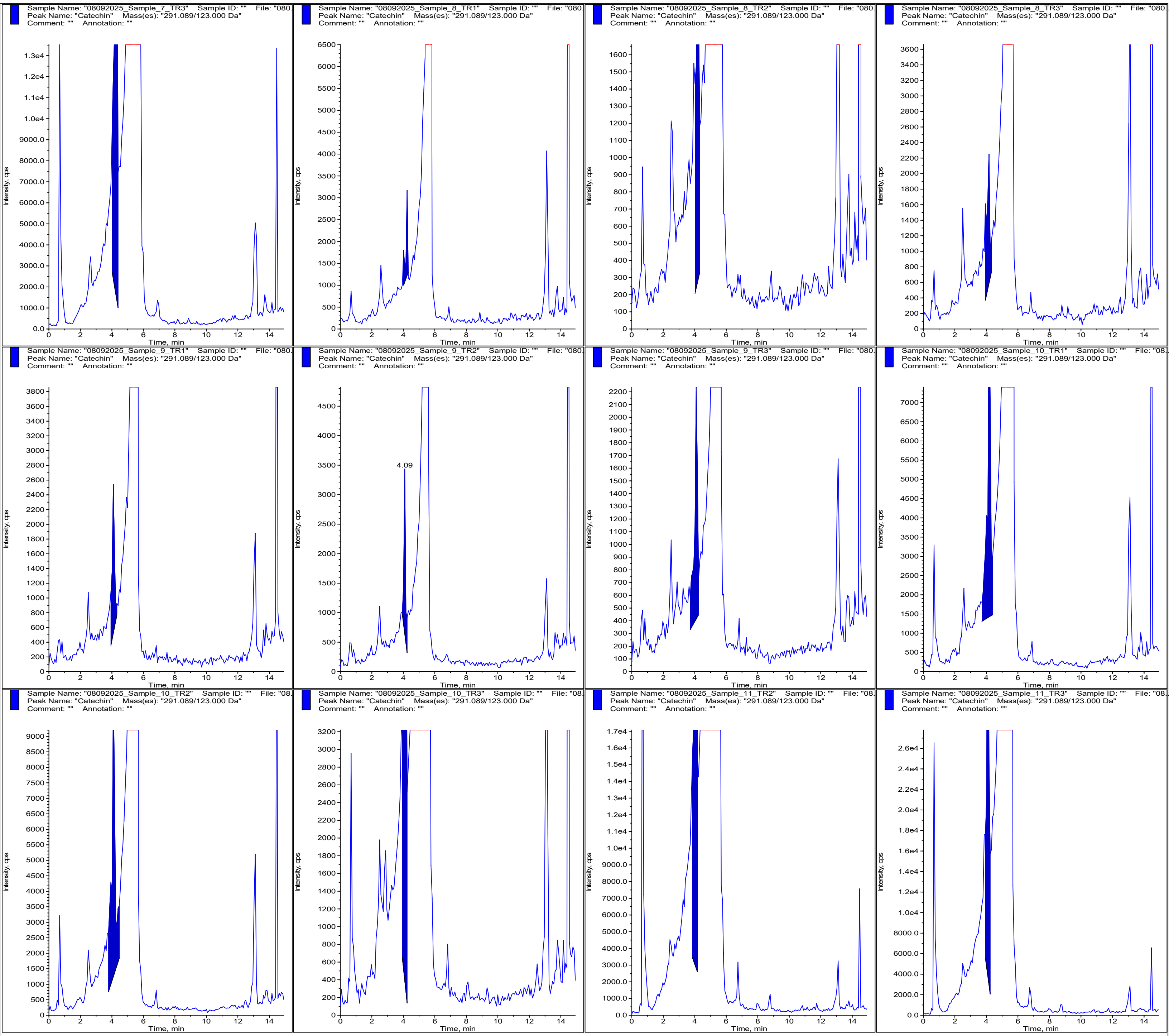

	Sample Name	Sample ID	Sample Type	File Name	Analyte Peak Area (counts)
25	08092025_Sample_7_TR3		Unknown	28072025_Akhil_Dr	4.04e+005
26	08092025_Sample_8_TR1		Unknown	28072025_Akhil_Dr	1.37e+004
27	08092025_Sample_8_TR2		Unknown	28072025_Akhil_Dr	3.01e+004
28	08092025_Sample_8_TR3		Unknown	28072025_Akhil_Dr	2.24e+004
29	08092025_Sample_9_TR1		Unknown	28072025_Akhil_Dr	2.09e+004
30	08092025_Sample_9_TR2		Unknown	28072025_Akhil_Dr	1.96e+004
31	08092025_Sample_9_TR3		Unknown	28072025_Akhil_Dr	2.39e+004

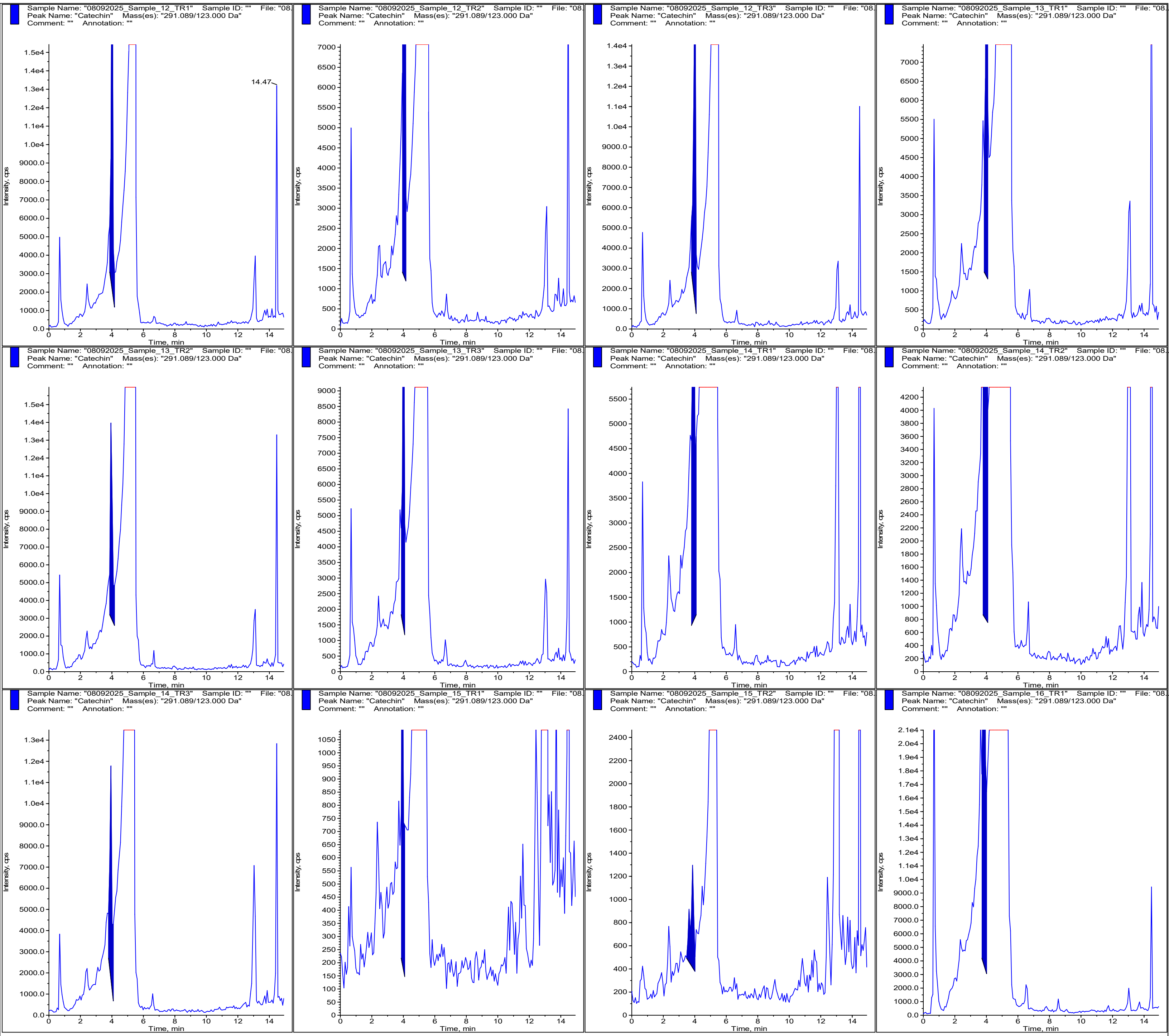

	Sample Name	Sample ID	Sample Type	File Name	Analyte Peak Area (counts)
37	08092025_Sample_12_TR1		Unknown	28072025_Akhil_Dr	1.58e+005
38	08092025_Sample_12_TR2		Unknown	28072025_Akhil_Dr	1.51e+005
39	08092025_Sample_12_TR3		Unknown	28072025_Akhil_Dr	1.54e+005
40	08092025_Sample_13_TR1		Unknown	28072025_Akhil_Dr	1.07e+005
41	08092025_Sample_13_TR2		Unknown	28072025_Akhil_Dr	1.00e+005
42	08092025_Sample_13_TR3		Unknown	28072025_Akhil_Dr	9.60e+004
43	08092025_Sample_14_TR1		Unknown	28072025_Akhil_Dr	1.02e+005

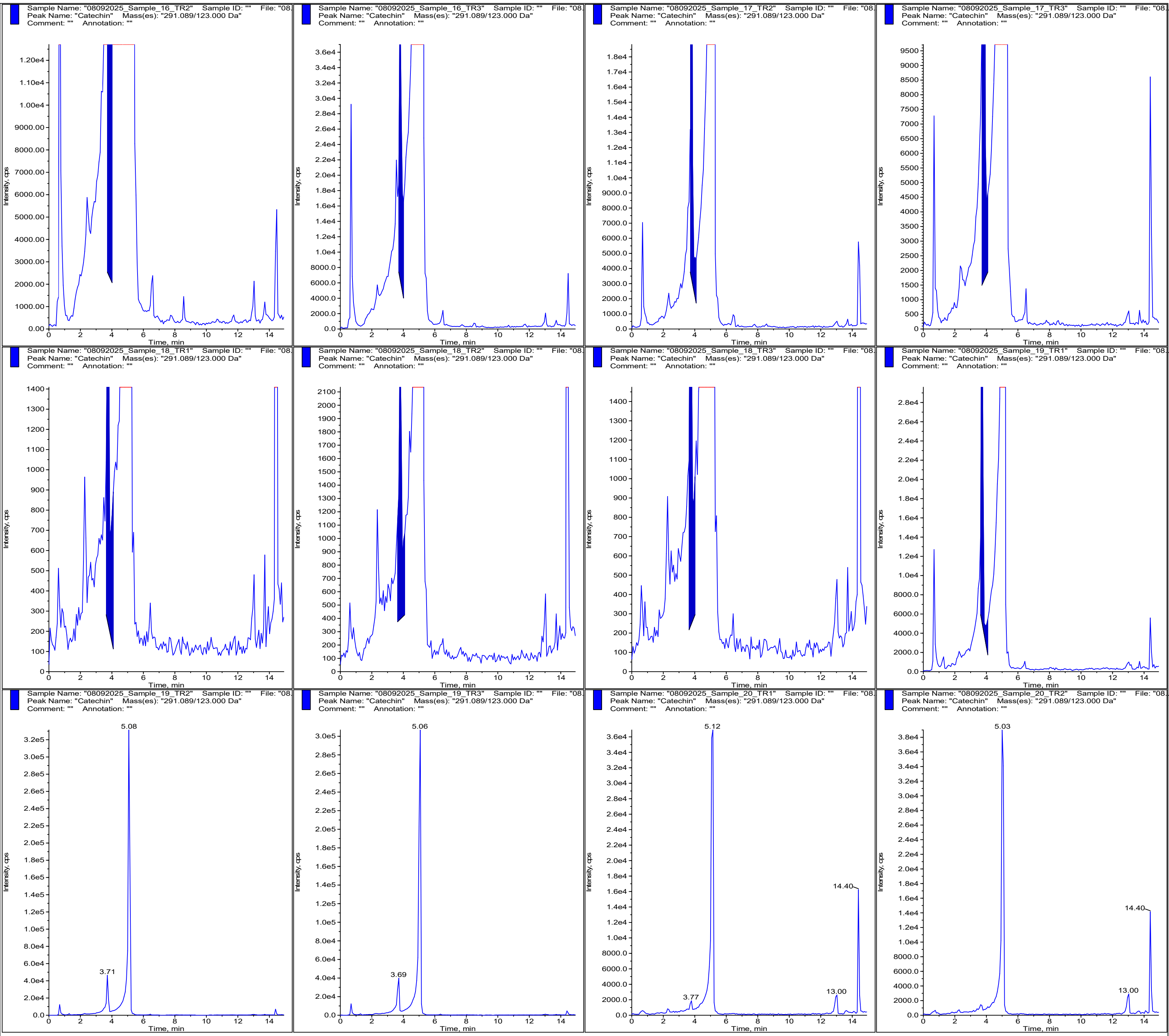

	Sample Name	Sample ID	Sample Type	File Name	Analyte Peak Area (counts)
49	08092025_Sample_16_TR2		Unknown	28072025_Akhil_Dr	4.49e+005
50	08092025_Sample_16_TR3		Unknown	28072025_Akhil_Dr	3.87e+005
51	08092025_Sample_17_TR2		Unknown	28072025_Akhil_Dr	2.81e+005
52	08092025_Sample_17_TR3		Unknown	28072025_Akhil_Dr	3.14e+005
53	08092025_Sample_18_TR1		Unknown	28072025_Akhil_Dr	2.55e+004
54	08092025_Sample_18_TR2		Unknown	28072025_Akhil_Dr	2.80e+004
55	08092025_Sample_18_TR3		Unknown	28072025_Akhil_Dr	2.57e+004

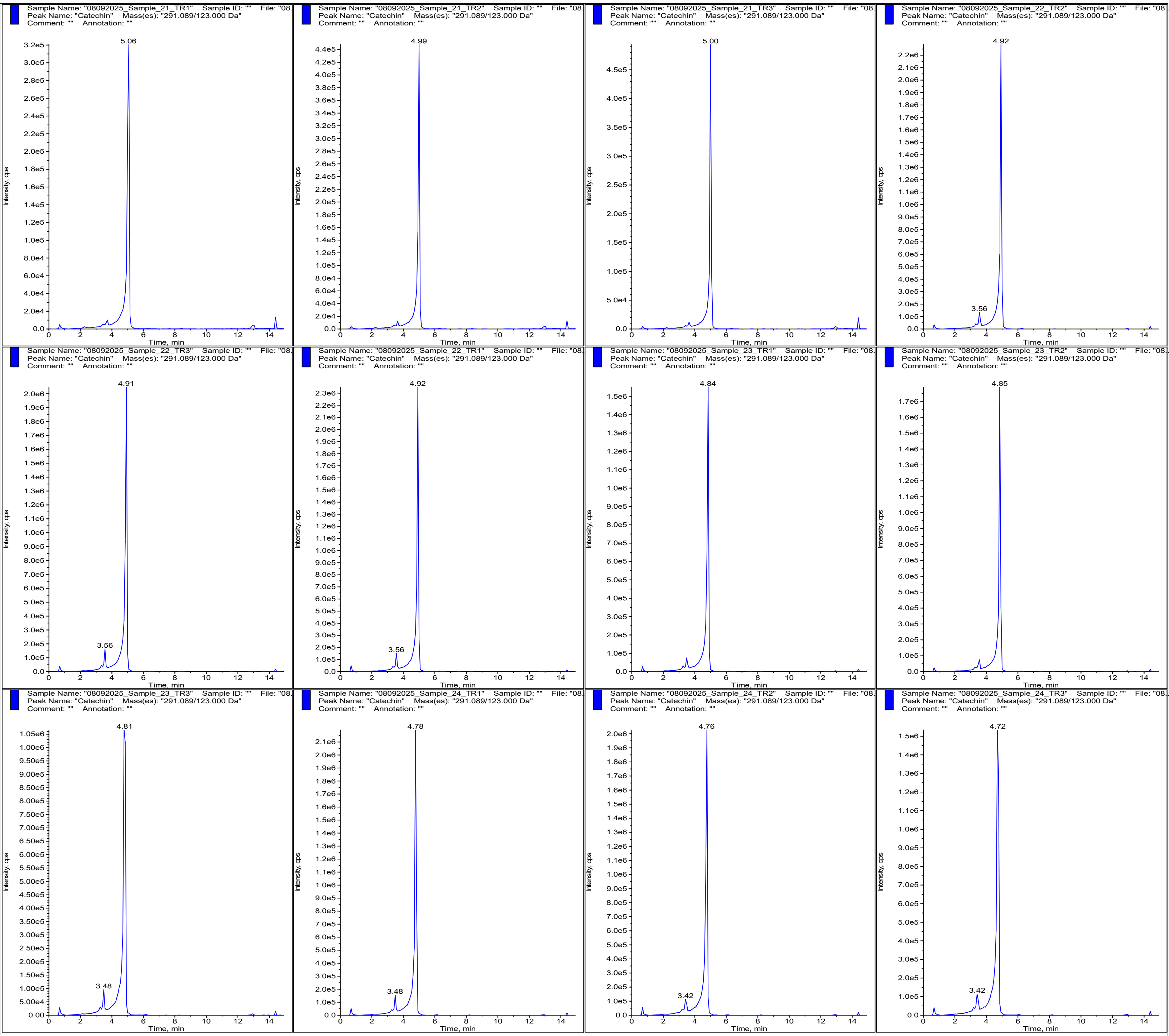

	Sample Name	Sample ID	Sample Type	File Name	Analyte Peak Area (counts)
61	08092025_Sample_21_TR1		Unknown	28072025_Akhil_Dr	0.00e+000
62	08092025_Sample_21_TR2		Unknown	28072025_Akhil_Dr	0.00e+000
63	08092025_Sample_21_TR3		Unknown	28072025_Akhil_Dr	0.00e+000
64	08092025_Sample_22_TR2		Unknown	28072025_Akhil_Dr	0.00e+000
65	08092025_Sample_22_TR3		Unknown	28072025_Akhil_Dr	0.00e+000
66	08092025_Sample_22_TR1		Unknown	28072025_Akhil_Dr	0.00e+000
67	08092025_Sample_23_TR1		Unknown	28072025_Akhil_Dr	0.00e+000

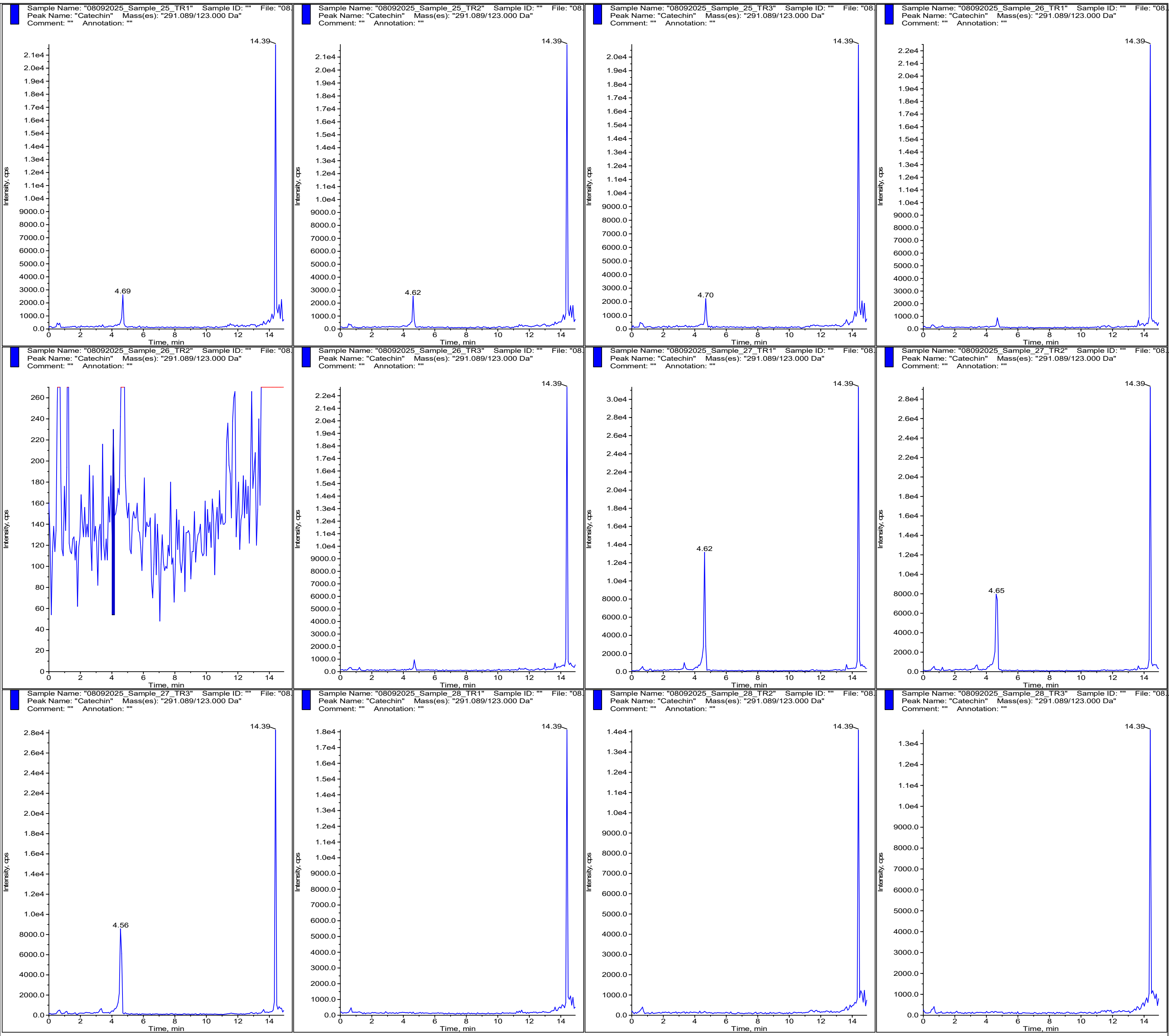

	Sample Name	Sample ID	Sample Type	File Name	Analyte Peak Area (counts)
73	08092025_Sample_25_TR1		Unknown	28072025_Akhil_Dr	0.00e+000
74	08092025_Sample_25_TR2		Unknown	28072025_Akhil_Dr	0.00e+000
75	08092025_Sample_25_TR3		Unknown	28072025_Akhil_Dr	0.00e+000
76	08092025_Sample_26_TR1		Unknown	28072025_Akhil_Dr	0.00e+000
77	08092025_Sample_26_TR2		Unknown	28072025_Akhil_Dr	1.18e+003
78	08092025_Sample_26_TR3		Unknown	28072025_Akhil_Dr	0.00e+000
79	08092025_Sample_27_TR1		Unknown	28072025_Akhil_Dr	0.00e+000

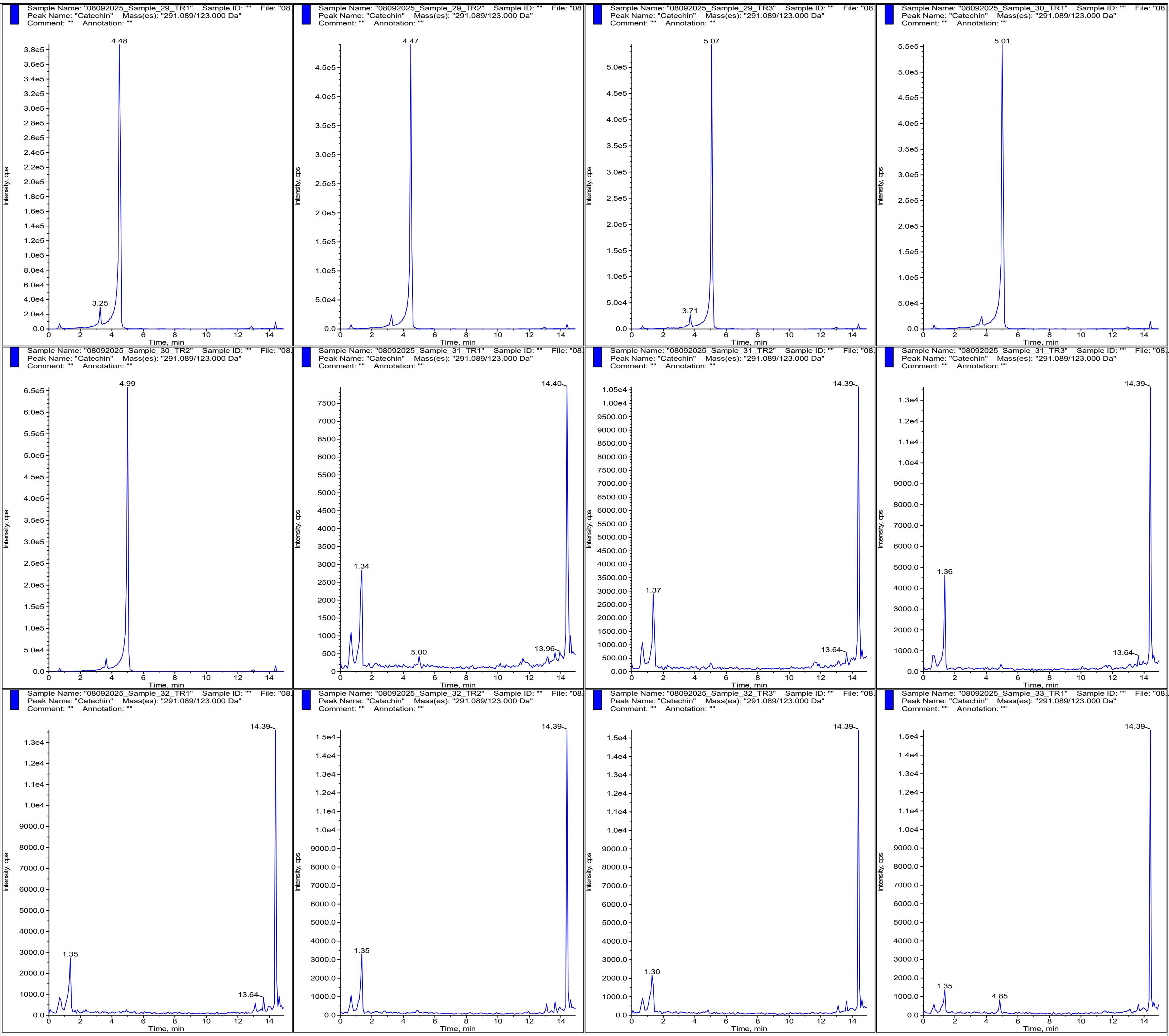

	Sample Name	Sample ID	Sample Type	File Name	Analyte Peak Area (counts)
85	08092025_Sample_29_TR1		Unknown	28072025_Akhil_Dr	0.00e+000
86	08092025_Sample_29_TR2		Unknown	28072025_Akhil_Dr	0.00e+000
87	08092025_Sample_29_TR3		Unknown	28072025_Akhil_Dr	0.00e+000
88	08092025_Sample_30_TR1		Unknown	28072025_Akhil_Dr	0.00e+000
89	08092025_Sample_30_TR2		Unknown	28072025_Akhil_Dr	0.00e+000
90	08092025_Sample_31_TR1		Unknown	28072025_Akhil_Dr	0.00e+000
91	08092025_Sample_31_TR2		Unknown	28072025_Akhil_Dr	0.00e+000

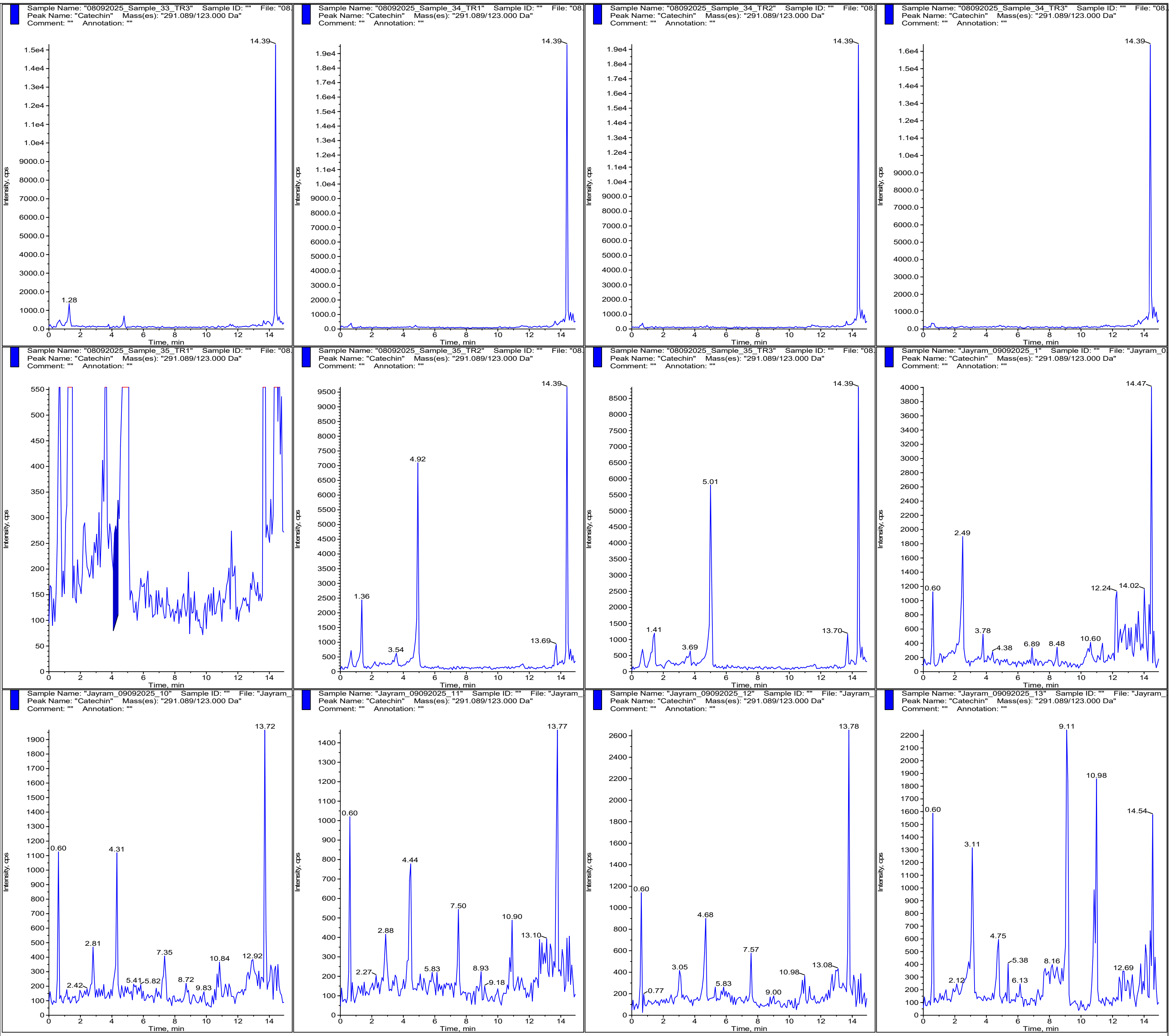

	Sample Name	Sample ID	Sample Type	File Name	Analyte Peak Area (counts)
97	08092025_Sample_33_TR3		Unknown	28072025_Akhil_Dr	0.00e+000
98	08092025_Sample_34_TR1		Unknown	28072025_Akhil_Dr	0.00e+000
99	08092025_Sample_34_TR2		Unknown	28072025_Akhil_Dr	0.00e+000
100	08092025_Sample_34_TR3		Unknown	28072025_Akhil_Dr	0.00e+000
101	08092025_Sample_35_TR1		Unknown	28072025_Akhil_Dr	3.21e+003
102	08092025_Sample_35_TR2		Unknown	28072025_Akhil_Dr	0.00e+000
103	08092025_Sample_35_TR3		Unknown	28072025_Akhil_Dr	0.00e+000

Acq. File: 28072025_Akhil_DrNegi_Pos_Stds.dam,..

Sample Name: 20082025_Std_4_Dil_1
Sample Number: Sample 1 of 145

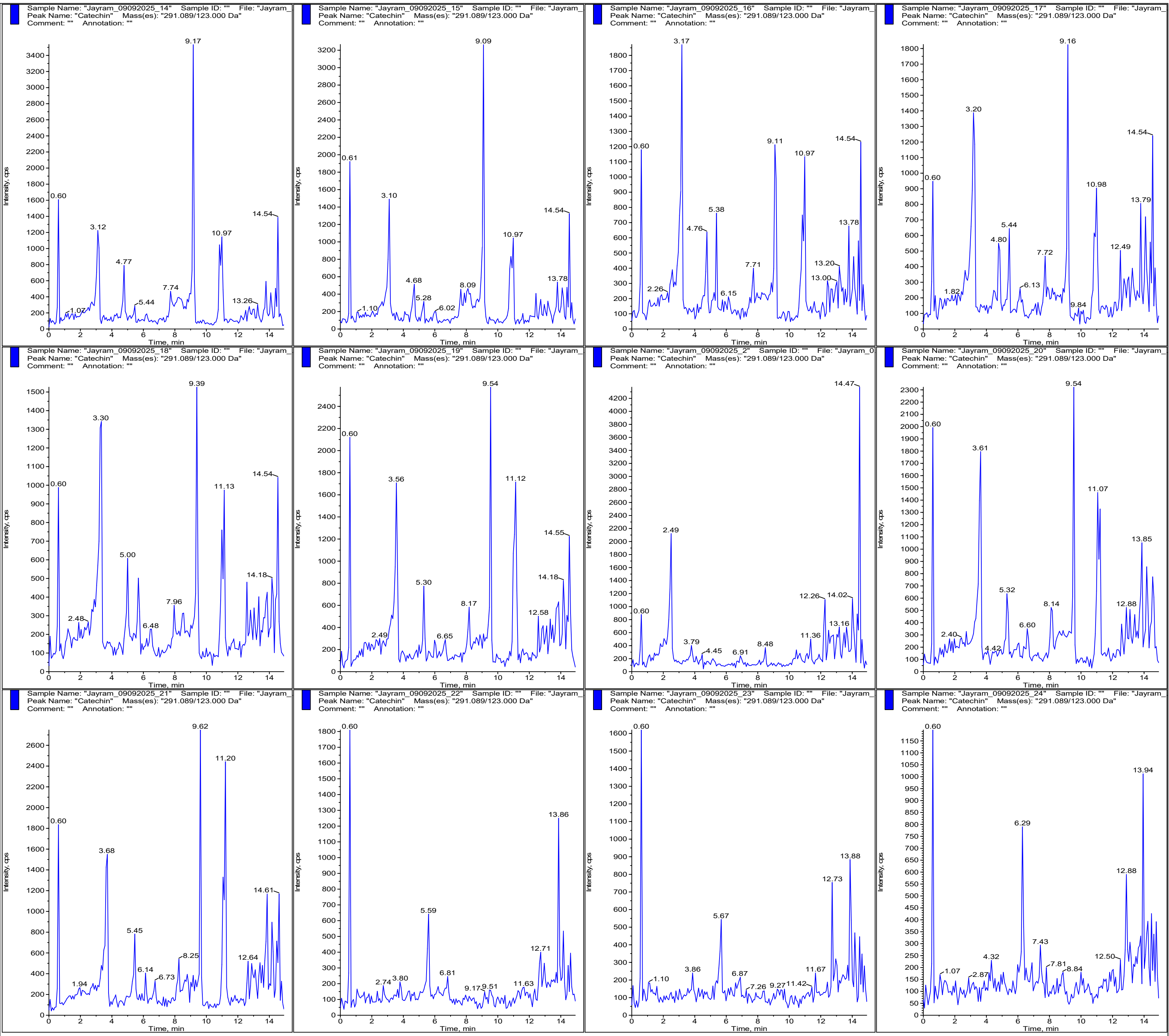

	Sample Name	Sample ID	Sample Type	File Name	Analyte Peak Area (counts)
109	Jayram_09092025_14		Unknown	28072025_Akhil_Dr	0.00e+000
110	Jayram_09092025_15		Unknown	28072025_Akhil_Dr	0.00e+000
111	Jayram_09092025_16		Unknown	28072025_Akhil_Dr	0.00e+000
112	Jayram_09092025_17		Unknown	28072025_Akhil_Dr	0.00e+000
113	Jayram_09092025_18		Unknown	28072025_Akhil_Dr	0.00e+000
114	Jayram_09092025_19		Unknown	28072025_Akhil_Dr	0.00e+000
115	Jayram_09092025_2		Unknown	28072025_Akhil_Dr	0.00e+000

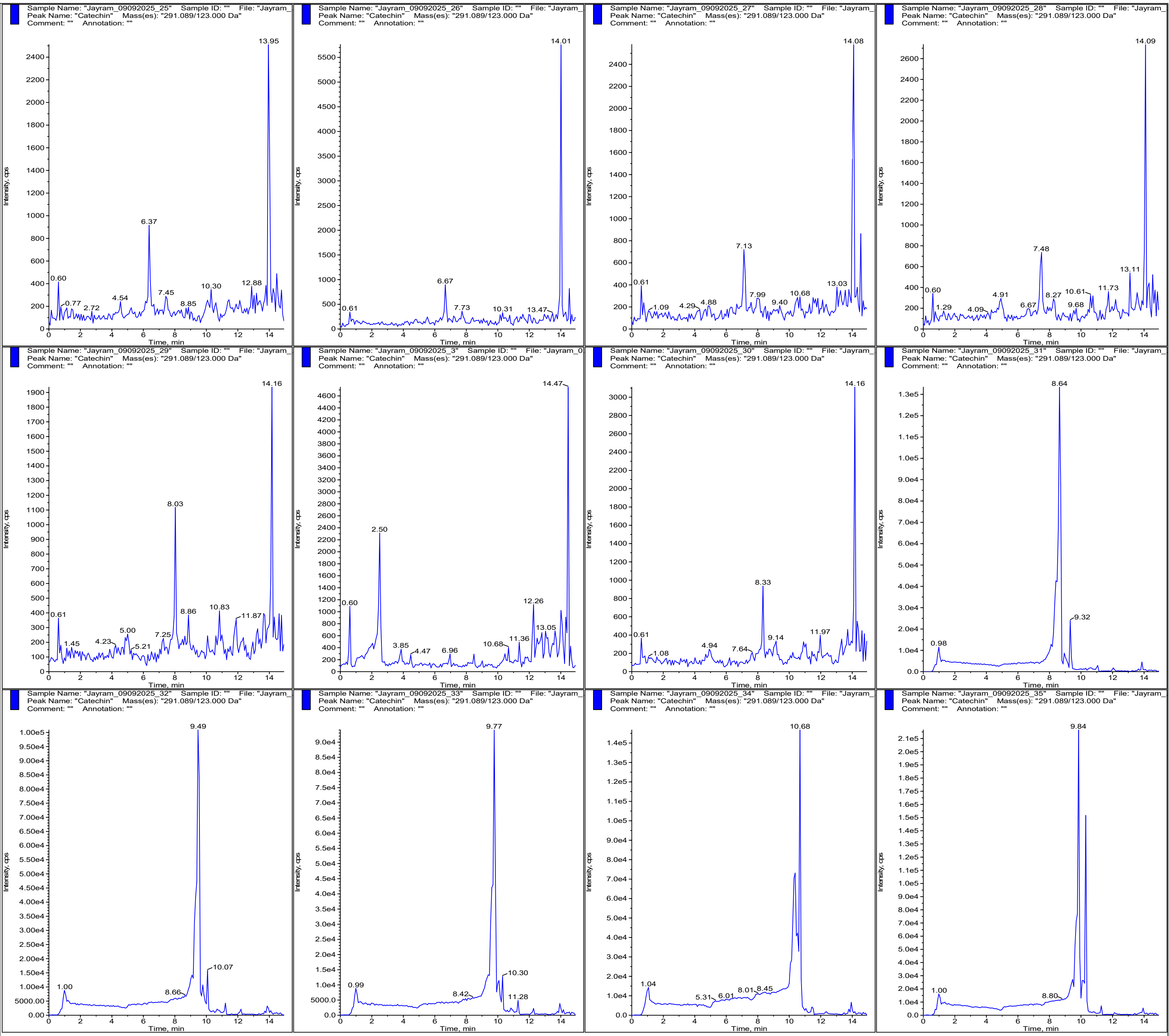

	Sample Name	Sample ID	Sample Type	File Name	Analyte Peak Area (counts)
121	Jayram_09092025_25		Unknown	28072025_Akhil_Dr	0.00e+000
122	Jayram_09092025_26		Unknown	28072025_Akhil_Dr	0.00e+000
123	Jayram_09092025_27		Unknown	28072025_Akhil_Dr	0.00e+000
124	Jayram_09092025_28		Unknown	28072025_Akhil_Dr	0.00e+000
125	Jayram_09092025_29		Unknown	28072025_Akhil_Dr	0.00e+000
126	Jayram_09092025_3		Unknown	28072025_Akhil_Dr	0.00e+000
127	Jayram_09092025_30		Unknown	28072025_Akhil_Dr	0.00e+000

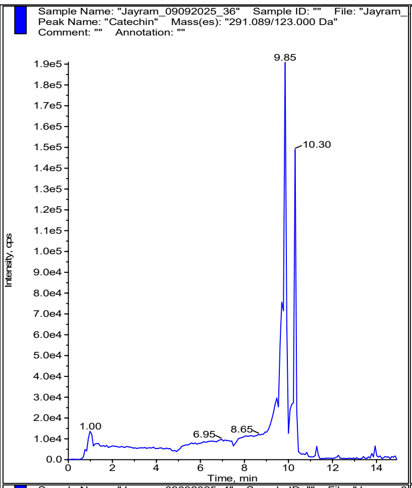

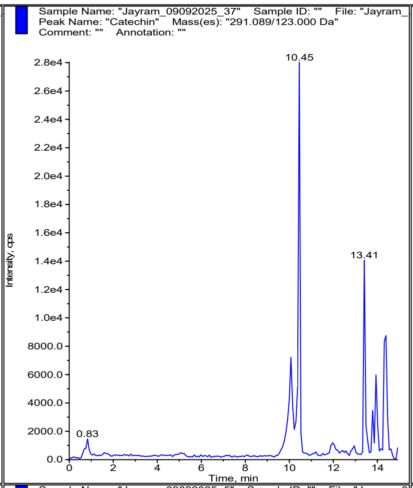

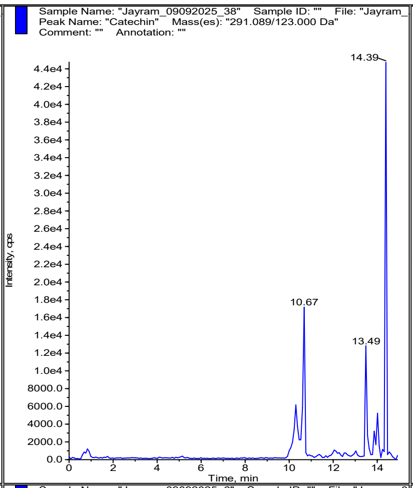

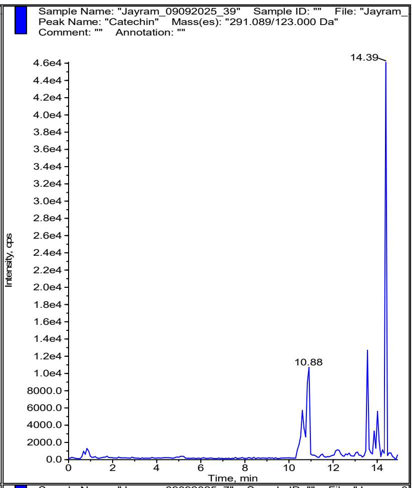

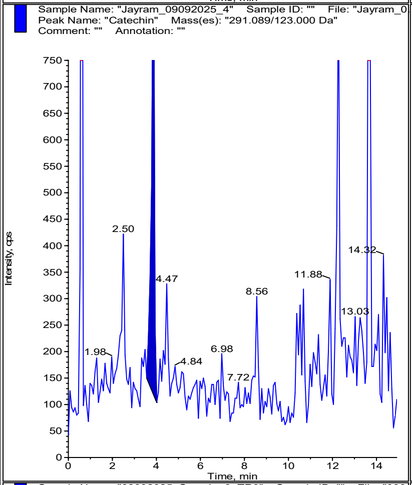

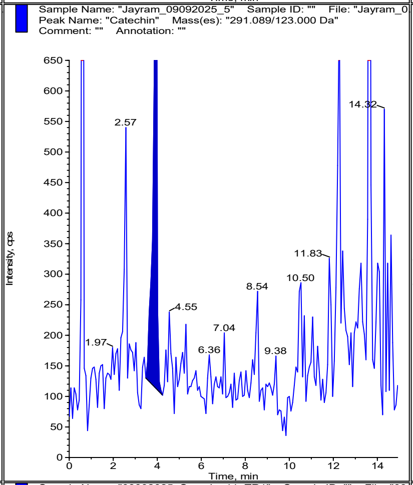

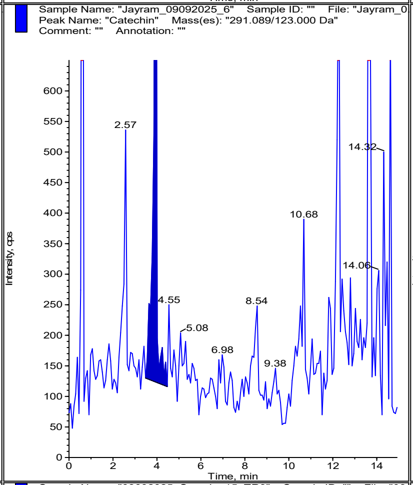

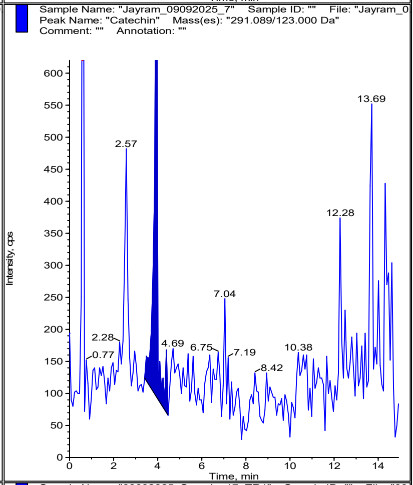

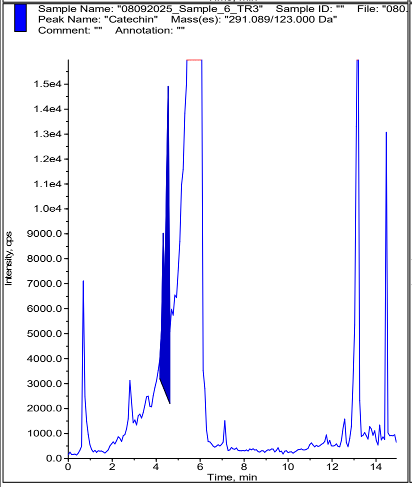

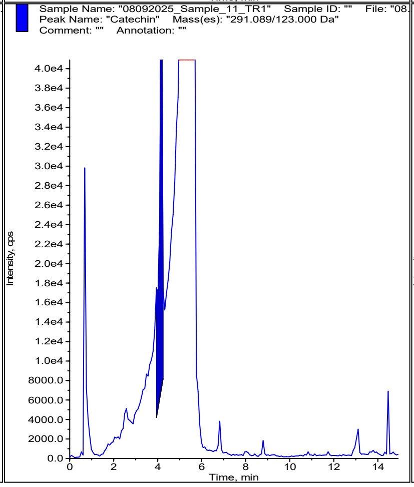

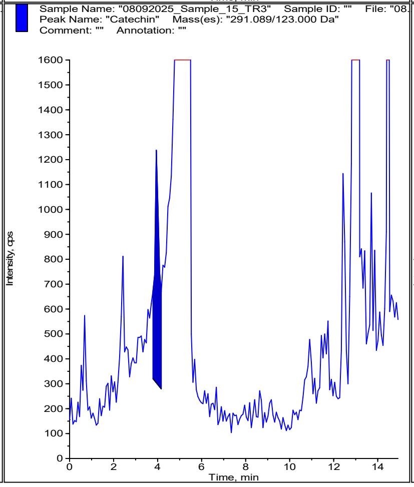

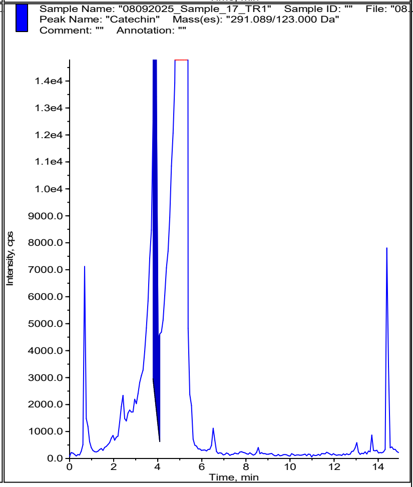

	Sample Name	Sample ID	Sample Type	File Name	Analyte Peak Area (counts)
133	Jayram_09092025_36		Unknown	28072025_Akhil_Dr	0.00e+000
134	Jayram_09092025_37		Unknown	28072025_Akhil_Dr	0.00e+000
135	Jayram_09092025_38		Unknown	28072025_Akhil_Dr	0.00e+000
136	Jayram_09092025_39		Unknown	28072025_Akhil_Dr	0.00e+000
137	Jayram_09092025_4		Unknown	28072025_Akhil_Dr	8.25e+003
138	Jayram_09092025_5		Unknown	28072025_Akhil_Dr	1.09e+004
139	Jayram_09092025_6		Unknown	28072025_Akhil_Dr	1.13e+004

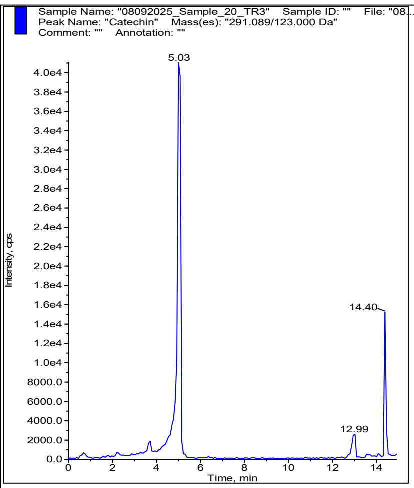

	Sample Name	Sample ID	Sample Type	File Name	Analyte Peak Area (counts)
145	08092025_Sample_20_TR3		Unknown	28072025_Akhil_Dr	0.00e+000
